# The Genus *Specklinia* Lindl. (Orchidaceae: Pleurothallidinae) in Mexico, a Taxonomic Review

**DOI:** 10.3390/plants15152284

**Published:** 2026-07-26

**Authors:** Ethian Licona, Rodolfo Solano, Fabiola Soto-Trejo

**Affiliations:** 1 Unidad de Biotecnología y Prototipos, Facultad de Estudios Superiores Iztacala, Universidad Nacional Autónoma de México, Av. de Los Barrios 1, Tlalnepantla de Baz 54090, Estado de México, Mexico; fabiolasototrejo@gmail.com; 2 Centro Interdisciplinario de Investigación para el Desarrollo Integral Regional, Unidad Oaxaca, Instituto Politécnico Nacional, Hornos 1003, Santa Cruz Xoxocotlán 71230, Oaxaca, Mexico

**Keywords:** conservation status assessment, Mexican orchids, Neotropical orchids, spatial distribution model, *Specklinia* affinity

## Abstract

*Specklinia* is an orchid genus composed of approximately 120 species. Although it has only been recognized recently as a monophyletic group, the presence of several poorly understood species complexes has led in the application of numerous names to taxa not occurring in Mexico and northern Central America. This study presents an updated and comprehensive systematic revision of *Specklinia* in Mexico, based on recent taxonomic and phylogenetic evidence. We examined *Specklinia* specimens housed in Mexican and international herbaria, as well as cultivated specimens and observations recorded on iNaturalist Mexico. For each species, we provided a description, taxonomic history, potential distribution model, conservation status assessment, photographic plate or botanical illustration are provided, along with information on distribution, habitat, phenology and comparisons with morphologically similar species. Twenty-two *Specklinia* species are accepted for Mexico, including five described as new (*S. orbiculata*, *S. perez-garciae*, *S. solanoi, S. wendtii*, and *S. xoconochcana*.) and two newly recorded for the country (*S. acicularis* and *S. juddii*). The identity of Mexican taxa morphologically similar to *S. digitale*, *S. endotrachys*, and *S. grobyi* are re-evaluated here. New combinations in *Specklinia* are proposed for *Pleurothallis choconiana* and *P. hagsateri*, which are recognized as distinct species. Lectotypes are designated for *Epidendrum lanceola*, *E. tribuloides*, *Pleurothallis marginata*, *P. pergarcilis*, *P. microphylla*, *P. lateritia* and *P. spathulata*. An identification key to the Mexican species of *Specklinia* is also provided. This study establishes a robust taxonomic framework for *Specklinia* in Mexico and highlights the need for continued systematic and conservation research, particularly in light of the proposed inclusion of 11 species under a risk category.

## 1. Introduction

The subtribe Pleurothallidinae Lindl. [1] is the most diverse lineage within the family Orchidaceae, comprising 46 genera and almost 5500 accepted species [2,3,4]. In Mexico it is represented by 22 genera and about 230 species [5,6]. The intergeneric relationships within Pleurothallidinae have been well studied and its genera are currently circumscribed as monophyletic groups based on both morphological and molecular evidence [3,4,7,8,9,10,11,12,13,14,15,16,17,18,19,20,21,22]. In contrast, intrageneric relationships remain poorly understood in several genera of the subtribe. Moreover, species delimitation has not been thoroughly assessed within complexes of morphologically similar taxa exhibiting wide geographic distribution.

*Specklinia* Lindl. [1] is a genus of Pleurothallidinae whose phylogenetic position place it in close relationship to *Andreettaea* Luer and to a clade comprising *Scaphosepalum* Pfizer, *Platystele* Schltr. and *Teagueia* (Luer) Luer [18]. *Specklinia* includes just under 120 described species, distributed from southern and central-east of Mexico to Bolivia and southern Brazil, including the Antilles [2]. Species richness is highest in Costa Rica (48 sp.), Panama (34 sp.), Colombia (31 sp.), Ecuador (31 sp.) and Nicaragua (19 sp.) [2,23,24,25,26,27,28]. Plants of *Specklinia* grow as epiphytes or lithophytes and occur across an elevational range from sea level to approximately 2400 m [29,30,31,32,33,34].

The first species currently assigned to *Specklinia* were originally described from plants collected in Jamaica: *Epidendrum lanceola* Sw., *E. corniculatum* Sw. and *E. sertularioides* Sw. [35]. Later, Sprengel [36] transferred these taxa to *Pleurothallis* R.Br. Lindley [1] established the genus *Specklinia*, including those three species together with *S. emarginata* Lindl., *S. floribunda* Lindl. and *S. linearis* Lindl., although he did not designate a nomenclatural type for the genus. At present, only *S. lanceola* (Sw.) Lindl. and *S. corniculata* (Sw.) Mutel (=*S. emarginata*) remain within *Specklinia*.

Over time, *Specklinia* came to be treated as a synonym of *Pleurothallis*, a genus that, at that time, was broadly circumscribed and recognized as heterogeneous and taxonomically problematic. Lindley subsequently included the species of *Specklinia* within *Pleurothallis* sect. *Apodae-caespitosae* Lindl. [37], an arrangement that was later followed by Garay [38] and Luer [39]. It was not until 1972 when Garay & Sweet designated *Epidendrum lanceola* as the lectotype for *Specklinia* [40]. Garay [38] treated *Specklinia* as a subgenus of *Pleurothallis*, while Luer expanded its circumscription to include *Pleurothallis* subg. *Specklinia* sect. *Muscariae* [39], which actually is recognized within *Andreettaea* Luer. Nonetheless, this broad delimitation also included species currently placed in *Anathallis*, *Lankesteriana* Karremans, *Pabstiella* Brieger & Senghas and *Stelis* Sw., resulting in a polyphyletic assemblage.

Molecular studies based on DNA data [7] demonstrated that *Specklinia* and *Pleurothallis* are not closely related, leading Pridgeon and Chase [8] to recognize *Specklinia* as a distinct genus, and circumscribe it with several sections previously recognized by Luer: *Cucumeres* Luer, *Hymenodanthae* Barb.Rodr., *Tribuloides* Luer and *Muscariae*—as well as *Pleurothallis* subgenera *Empusella* Luer and *Pseudoctomeria* (Kraenzl.) Luer—in addition to the genus *Acostaea* Schltr. However, this delimitation resulted in a paraphyletic assemblage.

Subsequently, Luer [41] further subdivided the concept of *Specklinia*, proposing numerous new genera, several of them monospecific. Recently, Karremans et al. [18], based on a phylogenetic analysis, redefined *Specklinia* as a monophyletic genus, which was divided into five subgenera [2,18,42]: *Dresslera* (Luer) Karremans & Vieira-Uribe, *Hymenodanthae* (Barb.Rodr.) Karremans, *Sarcinula* Karremans, *Specklinia s.s.* and *Sylphia* (Luer) Karremans. In this delimitation the authors included many genera previously proposed by Luer [41,43] and also confirmed that *Muscarella* Luer represents a distinct but closely related genus. This delimitation, which recognizes *Specklinia* as a phylogenetically well supported group, is adopted in the study for the treatment of the Mexican species.

In Mexico, the first species currently attributed to *Specklinia* were collected by Henri Galeotti and described as *Pleurothallis microphylla* A.Rich. & Galeotti and *P. spathulata* A.Rich. & Galeotti [44]. Hemsley [45] subsequently registered two species for the country: *P. microphylla* and *P. tribuloides* (Sw.) Lindl. Throughout the 20th century, several species were added to the Mexican Flora for this group, notably throughout the contributions of Lundell [46], Standley [47], Williams [48,49], Conzatti [50] and Oberg [51,52].

Williams [49] listed five species that are now assigned to *Specklinia*. Later, Luer described two additional species: *Pleurothallis digitale* Luer [30] from Veracruz and *P. hagsateri* Luer [32] from Guerrero. Soto-Arenas [53] reported ten species and several new additions, including *P. endotrachys* Rchb.f. and *P. vittariifolia* Schltr. Espejo-Serna and López-Ferrari [54] included nine species within *Pleurothallis* that are currently recognized as members of *Specklinia*.

In the 21st century, Soto-Arenas and Solano [55,56,57,58,59,60,61] proposed some new combinations and records, transferring several species from *Pleurothallis* to *Specklinia*. Subsequently, Soto-Arenas et al. [62] reported 16 Mexican species of *Specklinia* following the circumscription proposed by Pridgeon et al. [63], although some of these taxa are now assigned to *Andreettaea*. Villaseñor [5] also listed 16 species, although there are some nomenclatural differences compared to the previous treatment. More recently, Solano et al. [6] updated the list to 15 species, excluding those currently placed in *Andreettaea*.

Recent taxonomic reviews in other Neotropical regions have clarified the identity of some Mexican species of *Specklinia*. Pupulin et al. [64] determined that *S. endotrachys* (Rchb.f.) Pridgeon & M.W.Chase is endemic to Costa Rica while the specimens previously reported from Mexico with this name correspond instead to *S. spectabilis* (Ames & C.Schweinf.) Pupulin & Karremans. Similarly, Karremans et al. [65] established that *S. glandulosa* (Ames) Pridgeon & M.W.Chase is restricted to Costa Rica and Panama, while the correct name for the Mexican representative of this complex is *S. vittariifolia* (Schltr.) Pridgeon & M.W.Chase.

In this context, the present study provides an updated and comprehensive systematic revision of the genus *Specklinia* in Mexico, based on the most recent taxonomic and phylogenetic evidence. This work aims to establish a robust framework for future floristic and evolutionary studies within this complex group of miniature orchids.

## 2. Materials and Methods

### 2.1. Information Sources

Specimens of the Mexican species of *Specklinia* deposited at the national herbaria AMO, CH, CHAPA, CHIP, CICY, CST, ECO-TA-H, HEM, HUAP, HUMO, IEB, MEXU, QMEX, SERBO, UJAT and XAL were reviewed (acronyms according to Thiers [66]). In addition, digitized specimens from foreign herbaria deposited at AMES, ARIZ, BM, BR, CAS, F, GH, GOET, J.RENZ, K, LD, LL, MICH, MO, NYBG, P, S, SEL, UC, US, USJ and W were consulted throughout the respective websites. The JSTOR Global Plant Library (https://www.plants.jstor.org/) was also consulted for type specimens, as well as the microfilms collection housed at AMO herbarium.

Living and spirit-preserved specimens, as well as photographs, illustrations and diapositives housed in AMO herbarium were examined. Additional living specimens were studied from M.A.S. Orchidarium of the Faculty of Sciences, Universidad Nacional Autónoma de México (Mexico City), Ecological Reserve and Orchidarium Moxviquil (San Cristobal de las Casas, Chiapas), Orchidarium and Botanical Garden (Comitán, Chiapas) and private collections. Records deposited in the citizen science platform iNaturalist (https://mexico.inaturalist.org/) were also consulted and all the observations were curated to validate their taxonomic determination. In total, 762 specimens from 38 different herbaria, along with 240 iNaturalist observations were studied.

The protologues of all the binomials (both accepted and synonyms) corresponding to species reported from Mexico were consulted from their original sources through the Biodiversity Heritage Library (https://www.biodiversitylibrary.org/) and the library at AMO herbarium.

A database was constructed using the information obtained from the label of the herbarium specimens, including geographical data (country, state, municipality, locality, geographical coordinates and elevation), ecological information (climate, vegetation type, phenology, phorophytes and associated species) and collection data (collector, collection number and date). Geographical coordinates were verified and, when necessary, corrected using Google Earth (https://earth.google.com/web/) accessed on 14 September 2025.

### 2.2. Morphological Analysis

For the morphological description of the species treated here, the shape and measures of vegetative and floral structures were recorded in detail from herborized, spirit specimens and, when available, living flowering specimens. In some cases, with the kind permission of the collection curators, a dried flower was dissected to complete the morphological record. High resolution photographs were also taken using a Nikon^®^ D850 camera equipped with a Nikon^®^ AF S Micro Nikkor 105 mm 1:2.8 G lens and a set of three Nikon^®^ speedlight SB-R200 flashes (Nikon, Tokyo, Japan). These photographs were used to prepare a digital plate from each available species following Karremans [67]; the plates were edited using Adobe Photoshop^®^ CC. When no living material was available, a line or charcoal drawing was produced from a herborized specimen with the aid of a Wild Heerbrugg stereoscopic microscope (Type 308700; Wild Heerbrugg, Heerbrugg, Switzerland), attached with a lucid camera.

When the Mexican specimens were scarce, specimens collected near the borders with Guatemala and Belize were reviewed. Specimens from El Salvador, Honduras, Nicaragua, Costa Rica, Panama, Colombia, Venezuela, Ecuador, Peru, Brazil, Guyana, French Guiana, Cuba and Jamaica were also examined for species belonging to complexes of morphologically very similar taxa, with the aim of identifying differences in their morphology and their distribution patterns.

A morphological character matrix was compiled, and the most contrasting and informative characters were selected to construct the determination key.

### 2.3. Modeling of Potential Distribution

Subsequently, occurrence localities of each species were overlaid on a digital elevation model (DEM) map of Mexico using QGIS 3.22.9 [68]. To generate a potential distribution model for each species, a vector layer defining the “area M” was created. This region was delimited by combining the areas corresponding to species’ localities intersected with layers of Mexico’s political division [69], floristic provinces [70] and potential vegetation [71]. Raster layers comprising 19 bioclimatic variables and the DEM were obtained from WorldClim [72] at spatial resolution of 30 arc-seconds. The DEM of Mexico was additionally cropped to match the extent of area M for each species. Because these environmental variables may exhibit high levels of correlation, a preliminary model was run in MaxEnt 3.4.1 [73] to select a subset of the least correlated variables. This subset was subsequently used to build the final model, which incorporated between 4 and 12 variables per model.

In MaxEnt, the default settings were used, with “Hinge features” deactivated, and the options “Create response curves” and “Do jackknife to measure variable importance” activated. The logistic output format was selected. Under Settings/basic, the “Replicated run type” Bootstrap was selected, the “Random seed” option was activated, and 30% was assigned as the “Random test percentage”, with 100 replicates per model and one run per Bootstrap. In the Settings/advanced section, the “Write plot data” option was activated, whereas “Extrapolate” and “Do clamping” were deactivated. The “Apply threshold rule” was set to the 10-percentile training presence. However, when the number of occurrence localities were fewer than 11 but greater than 5, the “Replicated run type” Crossvalidate was used instead. This approach relies on comparative subsampling and uses the environmental values shared among the limited occurrences to generate a rigorously validated distribution model [74,75].

The model was then executed. To select the best model for each species, the model performance was evaluated using the area under the curve (AUC), applying the following criteria: 0.9–1.0 (excellent), 0.8–0.9 (good), 0.7–0.8 (acceptable), 0.6–0.7 (poor), and 0.5–0.6 (failed). Model selection also considered those models that incorporated the greatest number of environmental variables while minimizing the omission of known occurrence localities. Habitat suitability within each model was classified into four categories: not suitable (≤0.10), low suitability (0.11–0.30), moderate suitability (0.31–0.70), and high suitability (≥0.71) [75,76,77,78].

When the number of known occurrence localities were less than five, it was not possible to generate a distribution model. For these species only maps displaying the occurrence localities were presented.

### 2.4. Evaluation of Conservation Status

Finally, the conservation status for each species was assessed using both the Extinction Risk Assessment Method (MER) for plant species in Mexico, as defined in NOM-059-SEMARNAT-2010 [78], and criterion B for the IUCN Red List, regardless of whether the species was already included in any category under Mexican normativity.

For the MER assessment, the four conforming criteria (geographic distribution, habitat status, biological vulnerability, and anthropogenic impact) were evaluated for each species. These partial values were summarized to obtain a final score, which was then compared with the corresponding risk assessment scale to each species to a category.

For the IUCN assessment, extinction risk was determined based on estimates of geographic range following the criterion B, specifically B1 (extent of occurrence, EOO) and B2 (area of occupancy, AOO), in accordance with the guidelines of the IUCN Standards and Petition Committee (https://www.iucnredlist.org/documents/RedListGuidelines.pdf, accessed 22 June 2026) [79]. To this end, all occurrence records for each species were georeferenced. The Geospatial Conservation Assessment Tool (GeoCAT), an open-source web-based application was used to calculate EOO and AOO (https://geocat.iucnredlist.org/editor, accessed 22 June 2026) based on the georeferenced occurrence records.

## 3. Results

### 3.1. Richness, Distribution, Habitat and Reproductive Biology of Specklinia in Mexico

A total of 22 species of *Specklinia* are recognized in Mexico. Five species are described as new: *S. orbiculata*, *S. perez-garciae*, *S. solanoi*, *S. wendtii*, and *S. xoconochcana*. Meanwhile, two are newly recorded for the Mexican Flora: *S. acicularis* and *S. juddii*. Other two taxa previously treated as synonyms, *S. choconiana* and *S. hagsateri*, are reinstated as distinct species with new combinations proposed. Five species are endemisms from Mexico (*S. digitale*, *S. hagsateri*, *S. microphylla*, *S. perez-garciae*, and *S. wendtii*).

**Figure 1:**
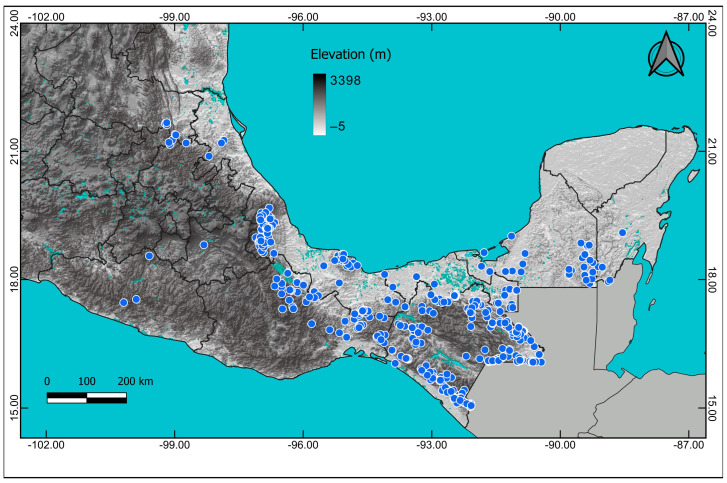
Distribution of currently known occurrence records (blue dots) for the genus *Specklinia* in Mexico according to the elevation model of Mexico.

In Mexico *Specklinia* is distributed between 0 and 2400 m elevation, extending from southeastern San Luis Potosi and northeastern Queretaro to the south of the Yucatan peninsula, and from Guerrero to Chiapas (Figure 1). Chiapas harbors the highest richness, with 19 of the 22 species, followed by Oaxaca (13 species), Veracruz (11 species) and Tabasco (eight species). According to the Floristic Provinces of Mexico [70], *Specklinia* occurs primarily in the Costa del Golfo de México Province, with additional representation in Soconusco, Serranías Transístmicas, Serranías Meridionales, Valle de Tehuacán, Sierra Madre Oriental, and Peninsula de Yucatán Provinces.

*Specklinia* plants prefer warm, humid to sub-humid environments. In Mexico, they occur mainly in the evergreen tropical forest, semi-deciduous tropical forest, tropical mountain cloud forest, riparian vegetation and mangrove forest. Nevertheless, some species prefer more temperate environments, such as *Quercus* and *Pinus-Quercus* forest. Several species show an affinity for habitats with a marked seasonality, like tropical deciduous forest, thorn forest and xerophytic rupicolous vegetation such as *S. marginata*, *S. perez-garciae*, *S. solanoi* and *S. tribuloides*. *Specklinia perez-garciae*, a microendemic of the Tehuantepec Isthmus, grows as a lithophyte in a xerophytic scrub surrounded by tropical deciduous forest, in a highly windy zone frequently covered by morning mist that helps to retain humidity and reduce direct solar radiation [80].

Some species appear to benefit by disturbance. For example, *S. blancoi*, *S. brighamii*, *S. choconiana*, *S. marginata*, *S. solanoi* and *S. tribuloides* are commonly found in secondary vegetation, on isolated trees in pastures, along roadsides and even on fences or poles near settlements. Others, like *S. brighamii*, *S. choconiana*, *S. digitale*, *S. lanceola* and *S. marginata* occur in orange and guava orchards, while *S. digitale*, *S. marginata*, *S. microphylla* and *S. tribuloides* are also found in coffee plantations. Some species like *S. digitale*, *S. marginata* and *S. tribuloides* persist on trees in urban parks.

Mexican species of *Specklinia* may flower throughout the year, but a pronounced flowering peak occurs at the end of the dry season, from April to June. Generally, flowers open successively over several months, resulting in extended flowering periods. However, in some species of subgenus *Hymenodanthae* the flowers open simultaneously and blooming events tend to be gregarious. Individual flowers remain open for one to two weeks in optimal conditions. Fruit matures within three to four months, although in species closely related to *S. spectabilis*, fruit development may take up to six months. Species belonging to the *S. endotrachys* complex exhibit a floral arrest following successful pollination of a single flower, stopping further flower production and initiating the development of a single fruit per inflorescence [81].

Very little is known about the reproductive biology of *Specklinia*, with only a few observational records and studies documenting pollination by small Diptera. Some species have been reported as being pollinated by Ceratopogonidae flies in Costa Rica [82], while in Chiapas, Mexico, *S. xoconochcana* (previously identified as *S. marginata*) has been observed to be pollinated by flies of the families Cecidomyiidae and Phoridae [83]. *Specklinia calyptrostele* produces nectar-like droplets on the inner surface of the sepals, which may function as attractants for pollinators [2]. Flowers of *Specklinia vierlingii* Baumbach are visited by Ulidiidae flies [82], and photographic evidence of *S.* aff. *corniculata* from Costa Rica shows visitation by a fly that appears to belong to the family Sciaridae (https://www.facebook.com/share/p/1AJ5TxgD4h/ Accessed 2 May 2025). Additionally, *Drosophila* species have been reported visiting the flowers of *S. alajuelensis* Karremans & Pupulin [65] and *S. fulgens* (Rchb.f.) Pridgeon & M.W. Chase [81] in Costa Rica. Reports on capsule production in *Specklinia* are scarce. Available data indicate a fruit set of 1.9% in *S. xoconochcana* (then identified as *S. marginata*) [82], and 40% in *S. pfavii* (Rchb.f.) Pupulin & Karremans, although only 8% of the flowers produced were pollinated.

### 3.2. Conservation Status

Among the 22 species of *Specklinia* treated in this study, four have been previously included in a risk category in Mexico, according to NOM-059-SEMARNAT-2010 [78]. Based on the conservation status assessment conducted using the four MER criteria, we propose the inclusion of 11 *Specklinia* species in this normativity list of threatened Mexican plants. Of these, five species are assigned to the category of Under Special Protection (Pr), two to Threatened (A) and four to Endangered (P) (Table 1).

At the same time, these species were assessed using the Criterion B of the IUCN red list of endangered species. This evaluation considered only the presence of the species within Mexican territory and does not reflect their conservation status across its entire distribution range. Based on this assessment, we propose the inclusion of 14 *Specklinia* species in the Mexican red list of threatened species. Of these, two are categorized as Near Threatened (NT), four as Vulnerable (VU), six as Endangered (EN) and two as Critically Endangered (CR) (Table 1).

### 3.3. Taxonomic Treatment

***Specklinia*** Lindl., Gen. Sp. Orch. PI., 8. 1830. Lectotype (designated by Garay and Sweet [40]): *Epidendrum lanceola* Sw.

*Pleurothallis* subg. *Specklinia* (Lindl.) Garay, Orquideología, 9 (2): 121. 1974.

*Pleurothallis* sect. *Apodae-caespitosae* Lindl., Fol. Orchid. 35. 1859. *Pleurothallis* subg. *Specklinia* sect. *Hymenodanthae* subsect. *Apodae-caespitosae* (Lindl.) Luer, Monogr. Syst. Bot. Missouri Bot. Gard., 20: 84. 1986. Lectotype: *Epidendrum corniculatum* Sw.

*Pleurothallis* sect. *Hymenodanthae* Barb.Rodr., Gen. et Sp. Orch. Nov., 2: 9. 1882. *Specklinia* subg. *Hymenodanthae* (Barb.Rodr.) Karremans, Phytotaxa, 272 (1): 22. 2016. Lectotype: *Pleurothallis grobyi* Bateman ex. Lindl.

*Lepanthes* sect. *Longicaulae* Barb.Rodr., Gen. Sp. Orchid. Nov., 2: 40. 1882. *Pleurothallis* subg. *Specklinia* sect. *Hymenodanthae* subsect. *Longicaule* (Barb.Rodr.) Luer, Monogr. Syst. Bot. Missouri Bot. Gard., 20: 86. 1986. Lectotype: *Pleurothallis trilineata* Barb.Rodr., non *Pleurothallis trilineata* Lindl. (=*Stelis velaticaulis* (Rchb.f.) Pridgeon & M.W.Chase).

*Acostaea* Schltr., Repert. Spec. Nov. Regni Veg., Beih., 19: 283. 1923. *Specklinia* subg. *Acostaea* (Schltr.) Karremans, Phytotaxa, 272 (1): 21. 2016, *nom. illegit*. Lectotype: *Acostaea costaricensis* Schltr.

*Pleurothallis* subg. *Dresslera* Luer, Monogr. Syst. Bot. Missouri Bot. Gard., 20: 38. 1986. *Areldia* Luer, Monogr. Syst. Bot. Missouri Bot. Gard., 95: 255. 2004, replace name. *Specklinia* subg. *Dresslera* (Luer) Karremans & S.Vieira-Uribe, Orquideología, 35 (2): 168–171. 2018. Type: *Pleurothallis dressleri* Luer.

*Pleurothallis* subg. *Specklinia* sect. *Cucumeres* Luer, Monogr. Syst. Bot. Missouri Bot. Gard., 20: 81. 1986. *Cucumeria* Luer, Monogr. Syst. Bot. Missouri Bot. Gard., 95: 257. 2004. Type: *Pleurothallis cucumeris* Luer.

*Pleurothallis* subg. *Empusella* Luer, Monogr. Syst. Bot. Missouri Bot. Gard., 20: 41. 1986. *Empusella* Luer, Monogr. Syst. Bot. Missouri Bot. Gard., 95: 258. 2004. Type: *Pleurothallis endotrachys* Rchb.f.

*Gerardoa* Luer, Monogr. Syst. Bot. Missouri Bot. Gard., 105: 86. 2006. Type: *Pleurothallis montezumae* Luer.

*Pseudoctomeria* Kräenzl., Bull. Misc. Inform. Kew 1925, (3): 116. 1925. *Pleurothallis* subg. *Pseudoctomeria* (Kräenzl.) Luer, Monogr. Syst. Bot. Missouri Bot. Gard., 20: 67. 1986. Type: *Pleurothallis lentiginosa* F.Lehm. & Kräenzl.

*Sarcinula* Luer, Syst. Bot. Missouri Bot. Gard., 105: 201. 2006. Type: *Pleurothallis acicularis* Ames & C.Schweinf.

*Specklinia* subg. *Sarcinula* Karremans, Phytotaxa, 272 (1): 24. 2016. Type: *Pleurothallis condylata* Luer.

*Sylphia* Luer, Syst. Bot. Missouri Bot. Gard., 105: 227. 2006. *Specklinia* subg. *Sylphia* (Luer) Karremans, Phytotaxa, 272 (1): 26. 2016. Type: *Pleurothallis turrialbae* Luer.

*Pleurothallis* sect. *Tribuloides* Luer, Syst. Bot. Missouri Bot. Gard., 20: 91. 1986. *Tribulago* Luer, Syst. Bot. Missouri Bot. Gard., 95: 265. 2004. Type: *Epidendrum tribuloides* Sw.

*Tridelta* Luer, Syst. Bot. Missouri Bot. Gard., 105: 232. 2006. Type: *Cryptophoranthus aurantiacus* Dod.

Epiphytic or rupicolous herbs, sympodial, cespitose or rarely rhizomatous; erect, ascending or decumbent. **Roots** terete, flexuous, whitish, sometimes orange or purplish. **Rhizome** abbreviated between adjacent stems, covered by papyraceous sheaths. **Stem** shorter than the leaf, terete, formed by 2–3 internodes, with an annulus near the base of the upper internode, each internode enclosed by a tubular, narrow, scarious or papyraceous sheath. **Leaf** fleshy or coriaceous, minutely bilobate and mucronate at the apex, longitudinally sulcate along the mid vein, attenuated at the base into a channeled petiole. **Inflorescence** erect or ascending, from the annulus, a lax or subfasciculate raceme; peduncle terete or rarely ancipitous, enclosed at the base by a scarious, triangular, conduplicate spathaceous bract; with 2–3 tubular-infundibuliform, oblique, acute, membranaceous, glabrous, hyaline bracts; sometimes fleshy, ancipitous. **Flowers** bilabiate (with the dorsal sepal forming an acute or obtuse angle with the lateral sepals) or rarely sub-campanulate, successive or simultaneous. **Sepals** membranaceous, acute or obtuse, glabrous, verrucose or glandulous, sometimes caudate or the apex of the dorsal sepal calyptrate; marginally entire to minutely erose; dorsal sepal free; lateral sepals connate in a variable length. **Petals** shorter than the sepals, membranaceous or fleshy, porrect, obtuse or rarely mucronate, marginally entire. **Lip** vibratile, fleshy, oblong, pandurate or shortly 3-lobulate, sometimes 2-auriculate at the base; articulated to the column foot by a hyaline claw; with a pair of submarginal and longitudinal calli, channeled between them, sometimes with a glenion-like depression near the apex. **Column** slender, elongated, broadened towards the apex, semi-terete, ventrally channeled, straight or arched, winged, with a pair of triangular teeth at the apex; foot stout, fleshy, shorter than or as long as the body, sometimes with a pair of globose or columnar calli; clinandrium cucullate, entire or denticulate. **Stigmatic cavity** ventral, sticky; rostellum ventral, galeate. **Anther** ventral, unilocular, partially divided by an incomplete septum inside. **Pollinia** two, pyriform or obovoid, yellow, similar, laterally compressed, without caudicles nor viscidium. **Ovary** unilocular, tricarpelar, trigonous, 3-keeled, glabrous, glandulous or echinate, articulated to a terete pedicel. **Capsule** usually glabrous, glandulous or echinate, trigonous, with persistent perianth.

Determination key for the subgenera of *Specklinia* in Mexico.
1. Leaves linear to oblanceolate; raceme subfasciculate; flowers reddish to purple, foul scented; lip with two auricled basal lobes…………………… *Specklinia* subg. *Sarcinula*
1. Leaves obovate to orbicular, rarely linear; raceme elongated or rarely subfasciculate; flowers white, pink, yellow or orange, normally unscented; lip without auricled basal lobes……………………………………………………………………………………. 2
2. Leaves biconvex, adaxially verrucose; flowers white, pink or yellow; lip oblong; column with two globose calli…………………………………. *Specklinia* subg. *Hymenodanthae*
2. Leaves flattened, glabrous; flowers yellow to orange; lip 3-lobulate, rarely entire; column generally without calli………………………………………………………………. 3
3. Raceme elongated; flowers caudate, yellowish, glabrous; petals obovate, obtuse; lobes of the lip obtuse and deflex; foot as long as the column body; ovary papillose–verrucose.………………………………………………………………. *Specklinia* subg. *Sylphia*
3. Raceme subfasciculate, sometimes elongated; flowers acute, yellow to orange, verrucose, glabrous or glandulous; petals lanceolate, acute; lobes of the lip acute and erect; foot shorter than the column body; ovary glabrous, glandulous or echinate……………………………………………………………. *Specklinia* subg. *Specklinia*

Key for the species of the subgenus *Sarcinula*.

1. Leaves acicular, triquetrous; raceme congested but not subfasciculate; flowers purple with yellow in the base; sepals adaxially spiculate; lip truncate–emarginate……………………………………………………………………………. *S. acicularis*
1. Leaves oblanceolate, flattened; raceme subfasciculate; flowers yellow with purple in the base; sepals glabrous; lip rounded…………………………………………… *S. brighamii*

Key for the species of the subgenus *Hymenodanthae*.

1. Raceme with simultaneous flowers; dorsal sepal thickened at the apex………………. 2
1. Raceme with successive flowers; dorsal sepal calyptrate at the apex…………………… 6
2. Plants > 5 cm tall without the inflorescence; leaves erect; peduncle as long as the leaf; flowers white; sepals recurved at the apex; petals lanceolate, slightly falcate; lip as long as the petals…………………………………………………………. *S. xoconochcana*
2. Plants < 5 cm tall without the inflorescence; leaves ascending to prostrate; peduncle longer than the leaf; sepals straight; petals variable in shape; lip longer than the petals…………………………………………………………………………………………… 3
3. Leaves obovate; inflorescence > 12 cm long; flowers yellowish; petals obovate; lip narrowly obovate……………………………………………………………………. *S. solanoi*
3. Leaves elliptic, oblanceolate or orbicular; inflorescence < 12 cm long; petals variable in shape; lip oblong.…………………………………………………………………………. 4
4. Leaves elliptic; raceme secund; flowers white to yellowish, sometimes with bright pink lines; petals obliquely lanceolate; lip magenta with the apex white…………………………………………………………………………… *S. marginata*
4. Leaves oblanceolate to orbicular; raceme distichous; flowers yellow with red lines; petals lanceolate; lip yellow with red lines………………………………………………… 5
5. Plants >3 cm tall without the inflorescence; leaves oblanceolate to obovate, with a petiole up to 8 mm long; dorsal sepal acuminate; synsepal lanceolate; petals acuminate……………………………………………………………………………… *S. choconiana*
5. Plants < 3 cm tall without the inflorescence; leaves orbicular–elliptic, with a petiole up to 2 mm long; dorsal sepal acute; synsepal elliptic–lanceolate; petals obtuse–mucronate……………………………………………………………………………. *S. orbiculata*
6. Leaves > 1.5 cm long; inflorescence > 5 cm long; peduncle up to 5 cm long; flowers >5 mm long…………………………………………………………………………. *S. digitale*
6. Leaves < 1.5 cm long; inflorescence <5 cm long; peduncle up to 2 cm long; flowers < 5 mm long……………………………………………………………………………………. 7
7. Flowers white with magenta lines; dorsal sepal with a poorly defined calyptra; petals lanceolate; lip magenta with two thickened calli……………………………… *S. pisinna*
7. Flowers yellowish-white, concolorous; dorsal sepal with a well-defined calyptra; petals obovate; lip yellowish, with two thin calli……………………………………………… 8
8. Leaves < 1 cm long; raceme up to 6 flowers; flower < 4.5 mm long; peduncle as long as the leaf; plants from the evergreen tropical forest………………………. *S. microphylla*
8. Leaves > 1 cm long; raceme up to 10 flowers; flower > 4.5 mm long; peduncle shorter than the leaf; plants from the tropical deciduous forest………………… *S. perez-garciae*

Key for the species of the subgenus *Sylphia*.

1. Leaves lanceolate, acute; inflorescence congested; peduncle as long as the leaf; lateral sepals sub-parallel towards the apex……………………………………………… *S. fuegi*
1. Leaves obovate, obtuse; inflorescence lax; peduncle longer than the leaf; lateral sepals divergent towards the apex………………………………………………………………. 2
2. Flowers yellowish suffused with purple, up to 7.8 mm long; lip purple, mid lobe as long as the lateral ones; plants from Chiapas and Veracruz……………………… *S. echinata*
2. Flowers yellowish, concolorous, up to 5.0 mm long; lip greenish-yellow, mid lobe longer than the lateral ones; plants from Guerrero…………………………………. *S. hagsateri*

Key for the species of the subgenus *Specklinia*.

1. Plants with subfasciculate inflorescences…………………………………………………. 2
1. Plants with elongated inflorescences………………………………………………………. 5
2. Peduncle abbreviated, <1 cm long; flowers verrucose–papillose.………………………. 3
2. Peduncle elongated, >2 cm long; flowers glabrous or glandulous……………………… 4
3. Plants cespitose; leaves linear, truncate; sepals obtuse, free in their apices; petals obliquely obovate………………………………………………………………… *S. blancoi*
3. Plants spiral-ascending; leaves obovate, obtuse; sepals acuminate, united in their apices; petals obliquely lanceolate…………………………………………………. *S. tribuloides*
4. Leaves linear, triquetrous; raceme glandulous, multiflorous; flowers chasmogamous, orange; sepals glandulous; synsepal oblanceolate, acute, bifid………… *S. vittariifolia*
4. Leaves oblanceolate, succulent; raceme glabrous, with one functional flower; flowers usually cleistogamous, yellow; sepals glabrous; synsepal lanceolate, obtuse, emarginate…………………………………………………………………………. *S. corniculata*
5. Plants < 10 cm tall without the inflorescence; raceme terete, <10 cm long; flowers up to 6, simultaneous, up to 7 mm long; sepals obtuse, glabrous; petals obovate, obtuse; lip elliptic……………………………………………………………………………. *S. lanceola*
5. Plants > 15 cm tall without the inflorescence; raceme ancipitous, >20 cm long; flowers up to 40, successive, >11 mm long; sepals acute to acuminate, verrucose; petals linear–lanceolate, acuminate; lip lanceolate–pandurate………………………………………. 6
6. Plants shortly rhizomatose; leaves narrowly obovate, up to 2.8 cm width; floral internodes shortening their length towards the apex; flowers up to 1.6 cm long; petals with the apex geniculate………………………………………………………… *S. wendtii*
6. Plants cespitose; leaves narrowly oblanceolate, up to 2.1 cm width; floral internodes equidistant from each other; flowers >1.6 cm long; petals with the apex cucullate or with a filamentous appendage…………………………………………………………… 7
7. Leaves up to 2.1 cm width; raceme up to 40 cm long; flowers up to 30, externally yellow, internally orange; lateral sepals straight and parallel from each other; petals cucullate; column yellow………………………………………………………………. *S. juddii*
7. Leaves up to 1.5 cm width; raceme up to 60 cm long; flowers up to 40, orange; lateral sepals recurved, divergent towards their apices; petals with a filamentous appendage in the apex; column orange……………………………………………… *S. spectabilis*

***Specklinia* subg. *Sarcinula*** Karremans, Phytotaxa, 272 (1): 22. 2016.

1. *Specklinia acicularis* (Ames & C.Schweinf.) Pridgeon & M.W.Chase, Lindleyana, 16 (4): 256. 2001.

**Figure 2:**
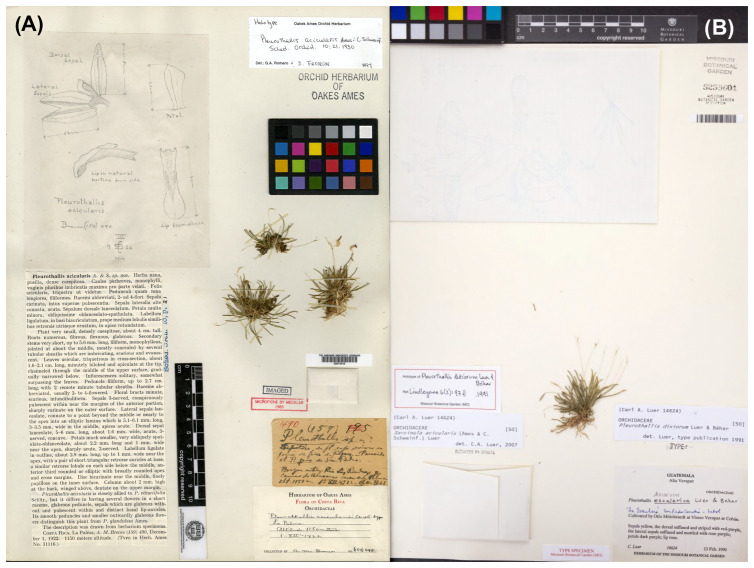
(**A**) Holotype of *Pleurothallis acicularis*, *A.M. Brenes (159) 490* (AMES-74018), from The Orchid Herbarium of Oakes Ames of Harvard University. (**B**) Holotype of *Pleurothallis dixiorum*, *C. Luer 14624* (MO-3148601, https://www.tropicos.org/image/101215815. Accesses 15 August 2023), from the Herbarium of Missouri Botanical Garden.

Basionym: *Pleurothallis acicularis* Ames & C.Schweinf., Schedul. Orchid., 10: 21. 1930. Type: COSTA RICA. Guanacaste: La Palma, forest between río Las Piedras and road to Alacena, near La Palma, 1150 m, 1 December 1922, A.M. Brenes (159) 490 (holotype: AMES-31116! (Figure 2A)).

Homotypic synonym: *Sarcinula acicularis* (Ames & C.Schweinf.) Luer, Monogr. Syst. Bot. Missouri Bot. Gard. 105: 202–203. 2006.

Etymology: From the Latin *acicula*, “needle”, referring to the acicular leaves of the species, unique in this subgenus.

Heterotypic synonym: *Pleurothallis dixiorum* Luer & Béhar, Lindleyana 6(2): 97. 1991. Type: GUATEMALA. Alta Verapaz: [San Pedro Carchá] Epiphytic in forest at “La Escalera,” between San Pedro Carchá and Sebol, ca. 1500 m, collected and cultivated by O. Mittelstaedt at Vivero Verapaz at Cobán, 13 Feb 1990. *C. Luer 14624* (holotype: MO-3148601! (Figure 2B)).

Etymology: Named after the doctors Margaret and Michael Dix, from the Universidad del Valle, Guatemala, who collected the type.

**Figure 3:**
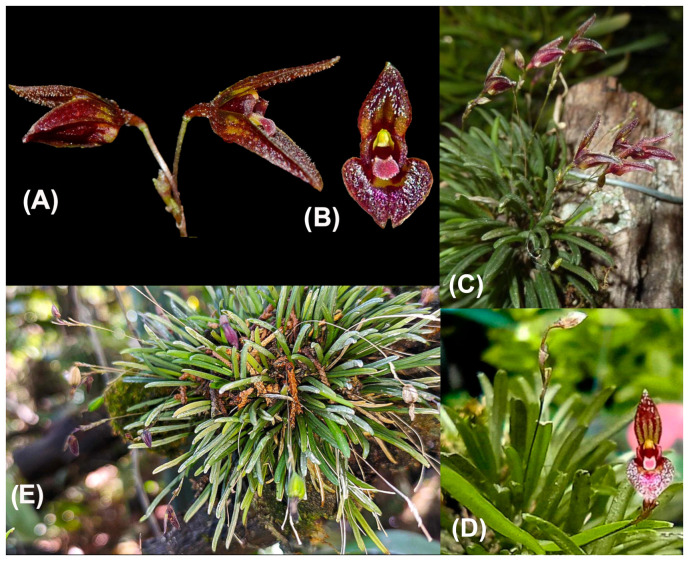
*Specklinia acicularis*. (**A**) Cultivated specimen from Alta Verapaz, Guatemala. Photograph by José Monzón and Edgar Mó. (**B**,**C**) Cultivated specimen in Atlixco, Puebla. Photograph by Azucena Briones. (**D**) Cultivated specimen in Coscomatepec, Veracruz. Photograph by Miguel Castillo. (**E**) Cultivated specimen in San Andrés Huayapam, Oaxaca. Photograph by C. Tomás-López.

**Figure 4:**
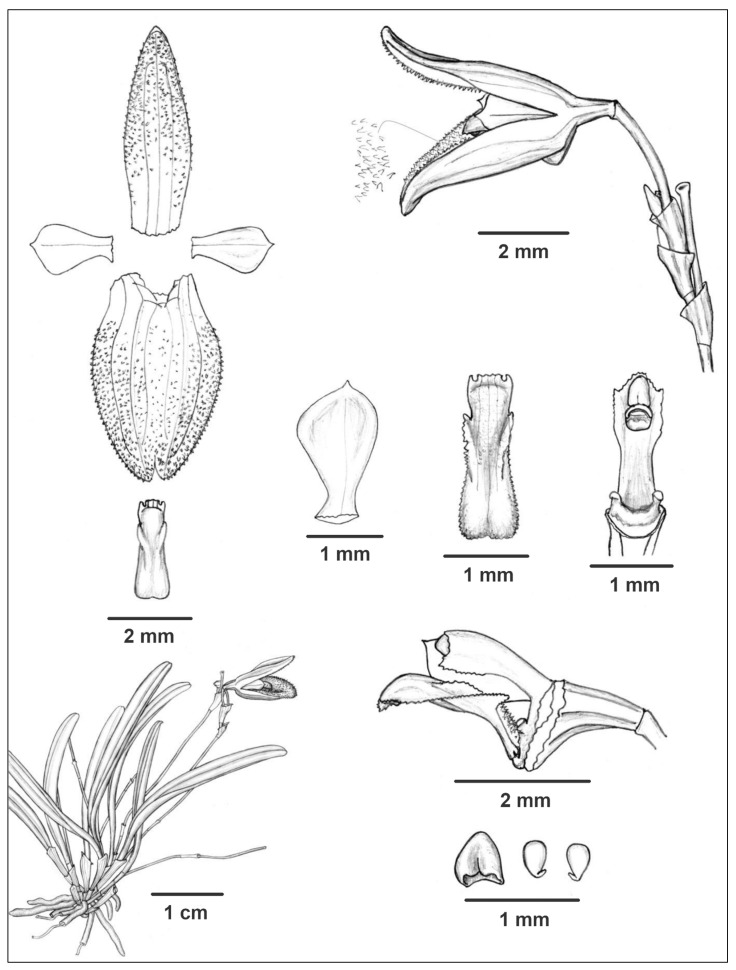
Illustration of *Specklinia acicularis*. Drawing by Rodolfo Solano, based on *R. Solano s.n.* (OAX).

**Herb** cespitose, erect, up to 4.3 cm tall without the inflorescence. **Roots** 0.8 mm gross. **Rhizome** 0.3–1.0 mm long between adjacent stems. **Stem** 1.6–8.0 mm long, 0.5–0.8 gross, the sheaths 1.2–3.5 mm long. **Leaf** 8.0–30.0 mm × 0.6–2.0 mm, succulent, acicular, triquetrous, obtuse to truncate, glabrous, the apical mucro 0.2–0.3 mm long, green with white dots on the abaxial surface, gradually attenuated into a petiole 1.2–5.0 mm long, 0.3–0.5 mm thick. **Inflorescence** 4.8–6.9 cm long; congested, erect, glabrous; spathaceous bract 1.0–1.2 mm long; peduncle longer than the leaf, 2.5–5.4 cm long, 0.1–0.25 mm gross, with 2 bracts, 1.0–1.8 mm long, membranaceous, glabrous, hyaline; rachis 0.3–1.5 cm long, with 2–8 flowers, 1–2 open at a time. **Floral bracts** 1.4–4.0 mm long, obliquely tubular-infundibuliform, obtuse, shortly apiculate, membranaceous, glabrous, hyaline, suffused with purple. **Flowers** 5.1–9.5 mm × 2.8–3.5 mm; sepals dark purple with yellow towards the base; petals dark purple; lip yellow with pink-magenta at the apex; column yellow, the foot suffused with purple; anther yellow. **Sepals** concave, axially carinate, adaxially papillar-spiculate, abaxially glabrous; dorsal sepal 5.0–9.5 mm × 1.6–3.0 mm, lanceolate–oblong to ovate–oblong, obtuse, 3-veined; lateral sepals 5.1–8.7 mm × 3.3–4.3 mm, connate into a widely elliptic to obovate, shortly bifid synsepal, 6-veined, gibbose in the base. **Petals** 2.2–3.0 mm × 1.0–1.7 mm, obliquely obovate to spathulate, apiculate, 2-veined. **Lip** 2.3–3.5 mm × 0.8–1.5 mm, fleshy, minutely 3-lobed, 3-veined; oblong to obovate, truncate, emarginate, minutely papillar-glandulous, marginally erose, convex towards the apex, with a pair of submarginal calli, erose–verrucose, channeled between them; lateral lobes obliquely triangular, erect, erose. **Column** 2.0–2.8 mm long, 0.7–1.1 mm width; wings obliquely triangular, obtuse, erose, 0.2–0.4 mm long; clinandrium obtuse, erose; foot 1.0–1.5 mm long, 0.3–0.5 mm width, slightly concave, obtuse, with two columnar calli at the base. **Stigmatic cavity** 0.3 mm × 0.4 mm, elliptic; rostellum laminar, oblong. **Anther** 0.4–0.5 mm long and width, ovoid. **Pollinia** 0.3–0.4 mm × 0.2–0.25 mm, obovoid. **Ovary** 1.0–1.9 mm long, 0.5–0.8 mm gross, obconical, recurved, glabrous, purple; pedicel 1.8–6.2 mm long, 0.2–0.3 mm gross, glabrous. **Capsule** 4.0–5.5 mm long, 2.0–2.5 mm gross, elliptic–obovoid, glabrous, green suffused with purple (Figure 3 and Figure 4).

**Figure 5:**
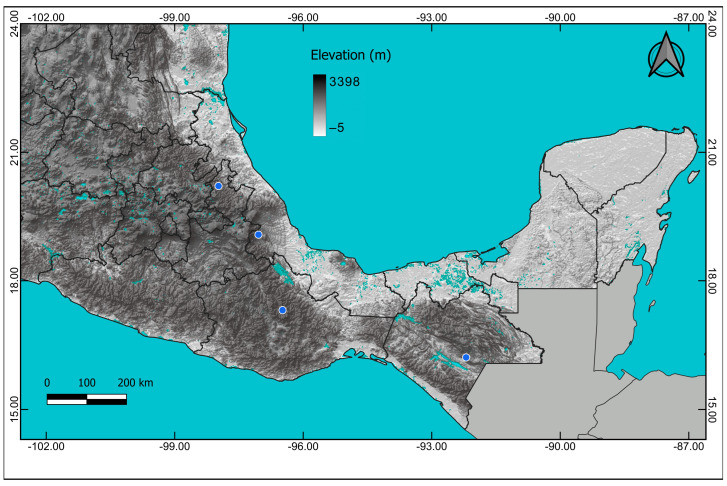
Probable localities of occurrence (blue dots) of *Specklinia acicularis* from Mexican origin. Map by Ethian Licona.

Distribution: Costa Rica, Guatemala and Mexico. This species represents a new record for the Mexican flora. It is known from specimens traded in traditional markets of Chiapas, Puebla and Veracruz and some cultivated in Oaxaca. However, in Guatemala it grows in humid forests in the Sierra de los Cuchumatanes, which has its northernmost limit in the Montebello Lakes, Chiapas [84] (Figure 5).

Habitat: Probably the tropical mountain cloud forest like that one from Montebello region in Chiapas.

Phenology: Flowering occurs in cultivation from April to December. Mature fruits have been observed from September to November.

Conservation status: It was not possible to assess its conservation status due to the lack of information about their wild populations and habitat.

Additional commentaries: *Specklinia acicularis* can be recognized by its acicular leaves; congested raceme, longer than the leaf; dark purple flowers; adaxially spiculate sepals; elliptic, shortly bifid synsepal; spathulate, apiculate petals; papillar-glandulous, oblong, bright yellow lip with the apex magenta, erose, with triangular and acute lateral lobes.

In Mexico this species may be confused in its habit with *S. vittariifolia*, but it differs by its wider leaves (up to 2.6 mm vs. up to 2.0 mm width), glandulous inflorescence (vs. glabrous), flowers up to 7.0 mm long, abaxially glandulous, bright orange (vs. up to 9.5 mm long, adaxially papillar-spiculate, dark purple), oblong–lanceolate synsepal, deeply bifid (vs. elliptic, shortly bifid), lip orange with the lateral lobes sub-pandurate (vs. yellow, with the apex magenta, with the lateral lobes narrowly triangular), column with long, obliquely triangular–subulate, dentate wings (vs. with short, triangular–obtuse, erose wings).

Examined specimens: MEXICO. Puebla: Atlixco, specimen cultivated by Mrs. Azucena Briones, 01.11.2015, *R. Solano s/n* (OAX!).

Additional records: MEXICO. Chiapas: Comitán de Domínguez, specimen cultivated by Leonel Gonzalez, 07.08.2023, *Leonel Gonzalez s/n* (Photograph). Oaxaca: Ixtlán de Juárez, specimen cultivated at Universidad de la Sierra Juárez, 15.06.2023, *C. Tomás-López s/n* (Photograph). San Andrés Huayapam, specimen cultivated by Octavio Suárez in Orquidario La Encantada, 18.11.2023, *C. Tomás-López s/n* (Photograph). Veracruz: Coscomatepec de Bravo, specimen cultivated by Miguel Castillo, 09.07.2023, *Miguel Castillo s/n* (Photograph).

2. *Specklinia brighamii* (S.Watson) Pridgeon & M.W.Chase, Lindleyana, 16 (4): 256. 2001.

**Figure 6:**
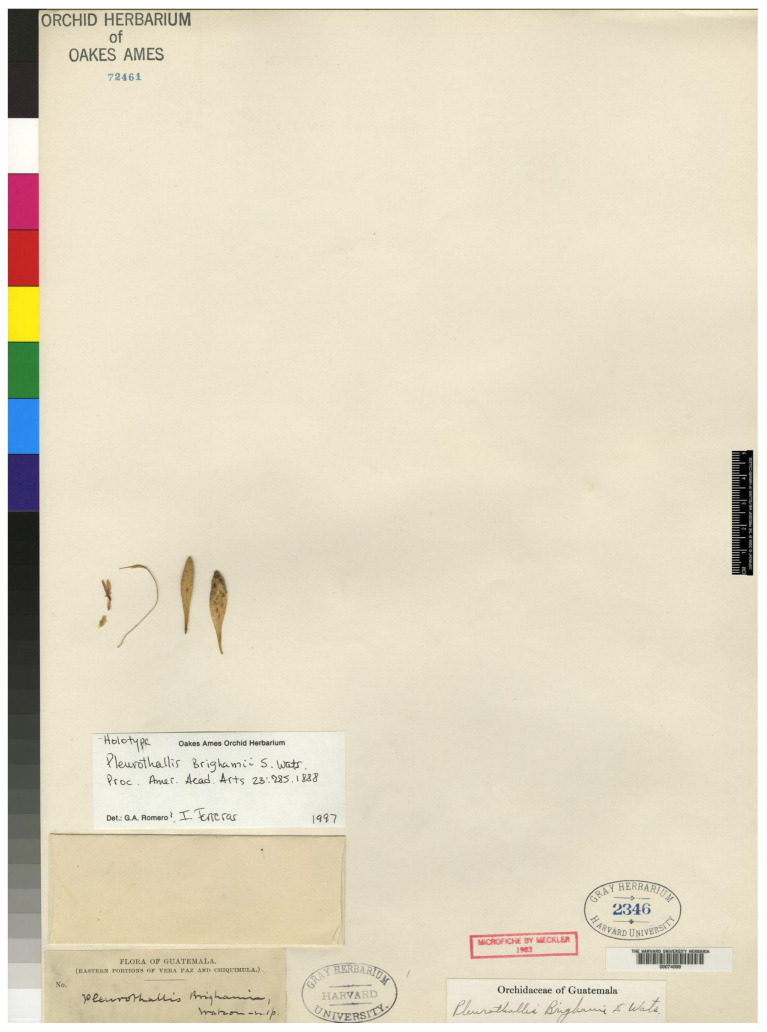
Holotype of *Pleurothallis brighamii*, *S. Watson s/n* (AMES-74099), from The Orchid Herbarium of Oakes Ames of Harvard University.

Basionym: *Pleurothallis brighamii* S.Watson, Proc. Amer. Acad. Arts 23 (2): 285–286. 1888. Type: GUATEMALA. [Izabal: Livingston] Eastern portions of Vera Paz and Chiquimula, on trees in the forests of Chocon, flowered in cultivation at Cambridge, August 1887. *S. Watson s.n.* (holotype: AMES-74099! (Figure 6), drawing based on the type AMES-74100!).

Homotypic synonym: *Sarcinula brighamii* (S.Watson) Luer, Monogr. Syst. Bot. Missouri Bot. Gard., 15: 206–207. 2006.

Etymology: Named after W.T. Brigham, American geologist, botanist and ethnologist.

**Figure 7:**
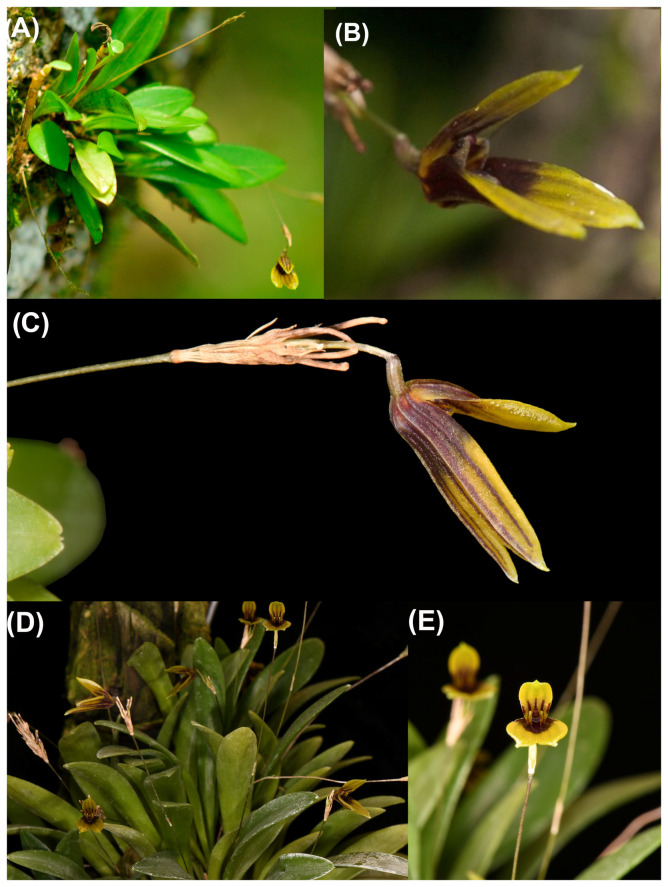
Photographs of *Specklinia brighamii*. (**A**) Specimen in its habitat, Las Margaritas, Chiapas. Photograph by José Espinoza. (**B**) Specimen in its habitat, Maravilla Tenejapa, Chiapas. Photograph by Jesús Moreno. (**C**–**E**) Specimen cultivated in Mexico City. Photographs by Ethian Licona, based on *M.A. Soto Arenas et al. 9573* (AMO).

**Figure 8:**
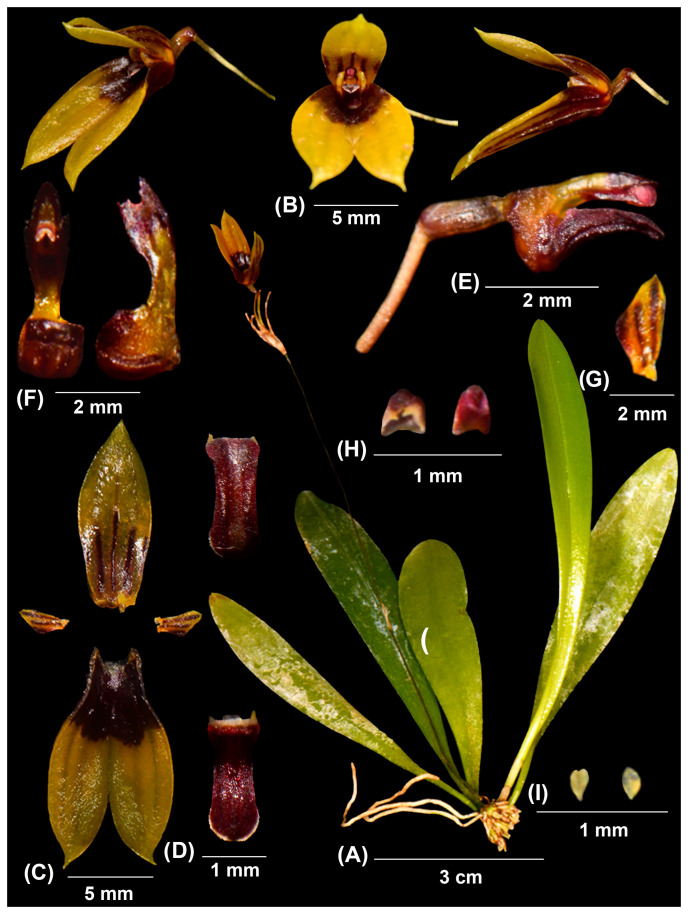
LCDP of *Specklinia brighamii*. (**A**) Habit. (**B**) Flower, ¾, frontal and lateral views. (**C**) Dissected flower. (**D**) Lip, dorsal and ventral views. (**E**) Column, lip and pedicelate ovary, lateral view. (**F**) Column, lateral and ventral views. (**G**) Petal. (**H**) Anther, dorsal and ventral views. (**I**) Pollinia, lateral and frontal views. Plate by Ethian Licona, based on *M.A. Soto Arenas et al. 9573* (AMO).

**Herb** cespitose, erect, up to 12.8 cm tall without the inflorescence. **Roots** 0.5–0.8 mm gross. **Rhizome** 1.0–3.0 mm long between adjacent stems. **Stem** 2.5–11.0 mm long, 0.6–0.8 mm gross, the sheaths 2.5–8.0 mm. **Leaf** 3.5–8.2 cm × 0.3–1.2 cm, coriaceous, flattened, linear–oblanceolate to narrowly oblanceolate, obtuse, glabrous, the apical mucro 0.2–0.4 mm long, light green, gradually attenuated into a petiole 0.3–3.5 mm long, 1.4–1.8 mm gross. **Inflorescence** 2.3–13.1 cm long, subfasciculate, erect, glabrous; spathaceous bract 1.8–2.0 mm long; peduncle as long as the leaf, 2.0–11.0 cm long, 0.4–0.5 mm gross, with 3 bracts 2.0–2.4 mm long, glabrous, papyraceous, whitish; rachis 0.3–2.1 cm long, with 5–20 flowers, 1 open at a time. **Floral bracts** 2.0–6.0 mm long, obliquely tubular-infundibuliform, acuminate, papyraceous, glabrous, whitish. **Flowers** 6.7–12.7 mm × 5.0–6.0 mm; sepals and petals bright yellow with purplish-red lines, purplish-red at the synsepal base; lip dark purple; column and foot bright yellow, turning purplish-red towards the apex; anther purplish-red. **Sepals** concave, axially carinate, glabrous, apiculate, marginally papillose; dorsal sepal 6.0–9.2 mm × 1.8–3.7 mm, elliptic–lanceolate to lanceolate, acute, 3-veined; lateral sepals 6.7–12.7 mm × 2.8–5.6 mm, connate in the basal ⅔ into an obovate to elliptic–obovate, deeply bifid synsepal, 6-veined, gibbose in the base, divergent in the apex. **Petals** 2.0–3.7 mm × 0.8–1.2 mm, obliquely oblanceolate, apiculate, 2-veined. **Lip** 2.1–3.0 mm × 0.7–1.4 mm, fleshy, minutely 3-lobed, 3-veined; oblong–ligulate, minutely papillar-glandulous, rounded, erose in the apex, with a pair of papillose submarginal calli, channeled between them; lateral lobes oblong, erect, rounded. **Column** 2.0–2.8 mm long, 0.7–1.0 mm width; wings obliquely trapezoidal, acute, dentate, 0.5–0.6 mm long; clinandrium acute, dentate; foot 1.2–1.5 mm long, 0.8–1.2 mm width, obtuse, with two conical calli in the base. **Stigmatic cavity** 0.25 mm × 0.3 mm, elliptic–oblong; rostellum laminar, oblong. **Anther** 0.4–0.5 mm long, 0.3–0.4 mm width, ovoid, with a mucro at the apex. **Pollinia** 0.3–0.4 mm × 0.2–0.25 mm, obovoid. **Ovary** 1.1–2.5 mm long, 0.4–1.0 mm gross, cylindrical–oblong, recurved, glabrous, yellowish-purple; pedicel 2.5–6.0 mm long, 0.2–0.3 mm gross, glabrous. **Capsule** 7.5–8.0 mm long, 3.0–3.7 mm gross, elliptic–obovoid, glabrous, purple to green (Figure 7 and Figure 8).

Distribution: Mexico, Guatemala, Belice, Honduras, Nicaragua, Costa Rica, Panama, Colombia, Ecuador, Cuba, Jamaica and Haiti. In Mexico, the species is distributed in the states of Campeche, Chiapas, Tabasco, Oaxaca and probably Quintana Roo. It occurs in the Floristic Provinces of Costa del Golfo de México, Peninsula de Yucatán, Serranías Meridionales and Serranías Transístmicas.

**Figure 9:**
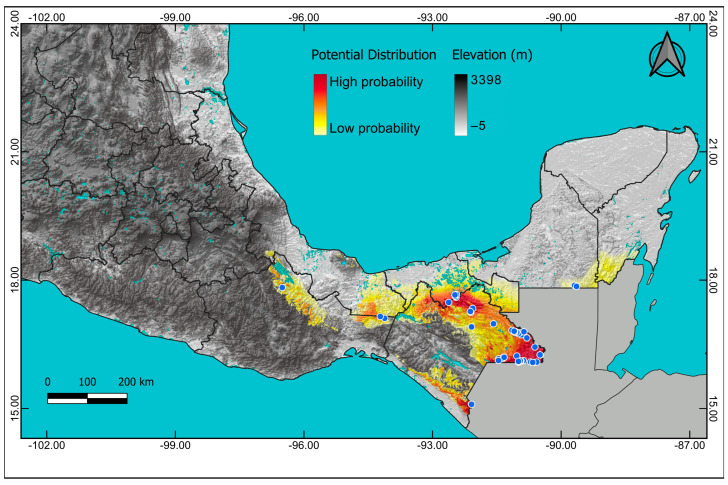
Potential distribution of *Specklinia brighamii* in Mexico and the location of currently known occurrence records (blue dots). Map by Ethian Licona.

The accepted potential distribution model for *S. brighamii* yields an AUC = 0.895, where the most influential environmental variables were temperature seasonality (22.7%), annual precipitation (21.8%) and precipitation of the warmest quarter (16.3%). The potential distribution area was estimated at 69,989.46 km^2^, representing approximately 3.56% of Mexico’s total territory (Figure 9).

Habitat: Epiphyte on *Brosimum alicastrum* Sw., *Ceiba pentandra* (L.) Gaertn., *Crescentia* sp., *Cynometra retusa* Britton & Rose, *Ficus* sp., *Terminalia amazonia* (J.F.Gmel.) Exell and *Coffea arabica* L. The species grows in the evergreen tropical forest, semi-deciduous tropical forest, secondary vegetation derived from these forest types and coffee plantations, at elevations between 10 and 1000 m. An anomalous record is known from Tacaná volcano, Chiapas, where it was collected in the tropical mountain cloud forest at 2052 m.

Phenology: Flowering occurs throughout the year, with a peak from January to September. Mature fruits have been observed from March to November. The flowers emit a diurnal scent similar to rotten fish.

Conservation status: In the assessment of its conservation status following the MER criteria [78], a score of 0.97 was obtained, therefore it is not necessary to include it in any Mexican risk category. In the evaluation under IUCN criterion B, it achieved an EOO of 123,943.998 km^2^ and an AOO of 184.00 km^2^, suggesting that the species should be classified as least concerned (LC).

Additional commentaries: *Specklinia brighamii* can be recognized by its narrowly oblanceolate leaves; subfasciculate raceme, as long as the leaf; bright yellow flowers with purplish-red lines; obovate, deeply bifid synsepal; oblanceolate, apiculate petals; oblong–ligulate, dark purple lip, with minute, oblong, rounded, erect lateral lobes.

*Specklinia brighamii* belongs to a morphologically variable complex, widely distributed and centered in Central America. This complex includes *Specklinia acrisepala* (Ames & C.Schweinf.) Pridgeon & M.W.Chase, *S. alexii* (A.H.Heller) Pridgeon & M.W.Chase, *S. barbelifera* Karremans, Salguero & Bogarín, *S. brighamii*, *S. simmleriana* (Randle) Luer and *S. tirimbina* Karremans, Salguero & M.Cedeño [85], together with the recently described *S. luluae* D.Gut.del Pozo, M.M.Jiménez & Iturralde [86].

No species occurring in Mexico is likely confused with *S. brighamii*. The most morphologically similar species are *S. simmleriana* and *S. luluae*. From *S. simmleriana* differs by its smaller leaves (2.5–5.0 × 0.4–0.7 cm vs. 3.5–8.2 × 0.3–1.2 cm), shorter inflorescence (up to 6.2 cm vs. up to 13.1 cm long), smaller flowers (up to 7.3 mm long vs. up to 12.7 mm long), lanceolate synsepal, with parallel apices (vs. elliptic–obovate, with divergent apices), obovate, yellow lip, half as long as the lateral sepals (vs. oblong–ligulate, dark purple, one third as long as the lateral sepals).

From *S. luluae* differs by its smaller, elliptic–lanceolate leaves (1.9–4.1 × 0.4–0.6 cm vs. linear–oblanceolate, 3.5–8.2 × 0.3–1.2 cm), inflorescence longer than the leaf (up to 8.0 cm long vs. as long as the leaf, up to 13.1 cm long), shorter flowers (up to 8.0 mm long vs. up to 12.7 mm long), lanceolate dorsal sepal (vs. elliptic–lanceolate), lanceolate–elliptic, narrow synsepal, with parallel apices (vs. elliptic–obovate, wide synsepal, with divergent apices), ruby red lip (vs. dark purple).

Examined specimens: MEXICO. Campeche: Calakmul, in a ruin discovered south of Villa Hermosa, 07.01.1932, *C. Lundell 1158* (AMES!). Chiapas: Benemérito de las Américas, 3 km al W de Nueva Veracruz, camino a Ixcán, sobre la carretera fronteriza del sur, 09.09.1985, *E. Martínez 13488* (GH!); 8 km al S de Benemérito de las Américas, camino a Flor de Cacao, 08.04.1985, *E. Martínez 11677* (IEB!); Nuevo Orizaba, 25 km al W del vértice del río Chixoy, camino a Chajul, 22.06.1986, *E. Martínez 18875* (CICY!, MEXU!); 6 km al E de Nuevo Orizaba, camino al vértice del río Chixoy, 17.05.1987, *E. Martínez 21037, G. Aguilar, G. Urquijo & A. Márquez* (MEXU!); Nueva Veracruz, 33 km al W del vértice del Río Chixoy, camino a Chajul, 22.06.1986, *E. Martínez 18862* (MEXU!); Nueva Chihuahua, 70 km al S de Boca Lacantún, 22.06.1986, *E. Martínez 18937* (MEXU!); Nueva Chihuahua, 70 km al S de Boca Lacantún, 23.06.1986, *E. Martínez 18974* (MEXU!). Chilón, cascadas de Agua Azul, 06.08.1985, *T. Chehaibar 121, A. Espejo & M. Palacios-Ríos* (MEXU!); cascadas de Agua Azul, 01.12.1974, *C. Lamas s/n* (MEXU!). Las Margaritas, km 41.7 del camino Montebello-Marqués de Comillas, 17.07.1999, *M.A. Soto-Arenas 9054 & L. López* (AMO!); Río Jabalí, 45 km al E de Tziscao, camino a Ixcán, sobre la carretera fronteriza del sur, 11.05.1984, *E. Martínez 6258 & A. Shilon* (IEB!, MEXU! MO!, UJAT!). Marqués de Comillas, Boca de Chajul, 20.02.2006, *G. López 353* (CHIP!). Ocosingo, 5 km al E de Crucero Corozal, camino a Frontera Corozal, 16.08.1984, *E. Martínez 7273* (ARIZ!, MEXU!, MO!, XAL!); 10 km al SE de Crucero Corozal camino a Boca Lacantún 18.08.1984, *E. Martínez 7455* (ARIZ!, XAL!); Zona urbana de Frontera Corozal, 2.64 km al SE de Frontera Corozal, 07.06.2004, *G. Aguilar 10516*, *F. Cruz & M. Meéz* (MEXU!); 2 km antes de San Andrés, sobre la carretera de terracería que llega del W, 05.03.2005, *B. Chiu 3* (HEM!); 0.4 km al N del banco de grava de San Javier, 23.11.2002, *D. Álvarez 2475, A. Aguilar & E. Martínez* (K!, MEXU!); 2.47 km al NE de San Javier, 20.09.2003, *G. Aguilar 7914, A. Chambor & C. Chancayun* (MEXU!); 1.38 km al NE del Crucero de San Javier, 25.10.2003, *G. Aguilar 8224, A. Chambor & C. Chancayun* (MEXU!); 200 m al N de la Estación Chajul, 02.05.1991, *E. Martínez 24536-A, C.H. Ramos, J. Larson, D. Benavides, C. Carrillo & C. Eufrosio* (MEXU!); Estación Chajul, 20.07.1992, *E. Martínez 25040, C.H. Ramos, R. Lombera & R. Guerrero* (AMO!); Estación Chajul, 01.04.2000, *M.A. Soto Arenas 9573, S. Maldonado, L. López & P. Schlutter* (AMO!); 20 km arriba del arroyo Miranda, por canoa, entrando por el río Chajul, 19.02.1985, *G. Castillo 3979, R. Acosta, R. Acevedo & F. Vásquez* (XAL!); 4 km al W de Crucero Corozal, camino Palenque-Boca Lacantún, 10.08.1984, *E. Martínez 6895* (MEXU!); 2 km al S de Crucero Corozal, camino Palenque-Boca Lacantún, 21.09.1984, *E. Martínez 7697* (MEXU!); Lacanjá Chansayab, 07.09.1985, *E. Martínez 13435* (MEXU!); Nahá, *A. Durán 580, 581 & S. Levy* (MEXU!); Arroyo José, camino a La Motobomba, 10.01.1999, *S. Sinaca 2160* (MEXU!); en la ribera del río Lacantún, 13.02.1999, *S. Sinaca 2278, E. Chankin & J. Vargas* (MEXU!); Campamento COFOLASA, 24 km al SE de Crucero Corozal, camino a Boca Lacantum, 13.08.1983, *E. Martínez 7082* (MO!); Sierra de La Cojolita, 20.09.1989, *M. González 724, P. Quintana, M. Martínez & N. Ramírez* (ECOSUR-CH!). Salto del Agua, camino a La Boquilla-Cascada de Agua Azul, 25.10.2011, *D. Torres s/n* (CHIP!). Tumbalá, Parque Nacional Cascadas de Agua Azul, 25.10.2011, *M. Jonapá 586* (CHIP!). Unión Juárez, volcán Tacaná, entre Talquian y la cima del volcán, 19.06.1985, *E. Martínez 13347 & O. Téllez* (MEXU!, MO!). Oaxaca: San Felipe Usila, Senda para la parcela de Inocente Joaquín Miguel, al NE de Santiago Tlatepusco, 18.05.1990, *J. Calzada 15225* (MO!). Santa María Chimalapa, vereda hacía el cerro Loma Larga, 29.01.1996, *M. Cerón 259, S. Salas & H. Morales* (AMO!); en las tierras bajas del norte, cerca de San Isidro La Gringa, undated, *T. Wendt* et al. *s/n* (AMO!). Tabasco: Macuspana, Centro Recreativo Agua Blanca, 7 km de la carretera Villahermosa-Escárcega, junto al río, 04.05.1980, *M.R. Niño 2975 & C. Cowan* (CST!); Centro Recreativo Agua Blanca, 27.05.1985, *S. Zamudio 1465* (ECOSUR-CH!, IEB!, XAL!); Balneario Agua Blanca, 23.10.1985, *Á. Aldarete 58* (MEXU!); Las Palomas, cerca de Agua Blanca, 15.05.1982, *S. Zamudio 314* (IEB!, XAL!). Tacotalpa, Selva Agua Blanca, 25.04.2005, *N. García 96* (UJAT!).

Additional records: MEXICO. Campeche: Calakmul, al SE de Villa Hermosa, 02.05.2024, *I. Arellano s/n* (https://www.inaturalist.org/observations/214067845. Accessed 3 July 2025). Chiapas: Las Margaritas, cerca de Nuevo San Cristobalito, 06.06.2017, *J. Espinoza s/n* (https://www.inaturalist.org/observations/6616812. Accessed 3 July 2025); al E del Parque Nacional Lagunas de Montebello, cerca del río Santo Domingo, undated, *M. A. Soto Arenas s/n* (Photograph). Maravilla Tenejapa, cerca de Gallo Giro, 02.08.2012, *J. Moreno s/n* (https://www.inaturalist.org/observations/2880866. Accessed 3 July 2025); Las Nubes, 01.05.2013, *A. Jiménez s/n* (Photograph). Marqués de Comillas, Boca de Chajul, 17.03.2015, *I. Martínez s/n* (https://www.inaturalist.org/observations/1305108). Accessed 3 July 2025.

***Specklinia* subg. *Hymenodanthae*** (Barb.Rodr.) Karremans, Phytotaxa, 272 (1): 22. 2016.

3. *Specklinia choconiana* (S.Watson) E.Licona, Solano & Soto-Trejo, *comb. nov.*

**Figure 10:**
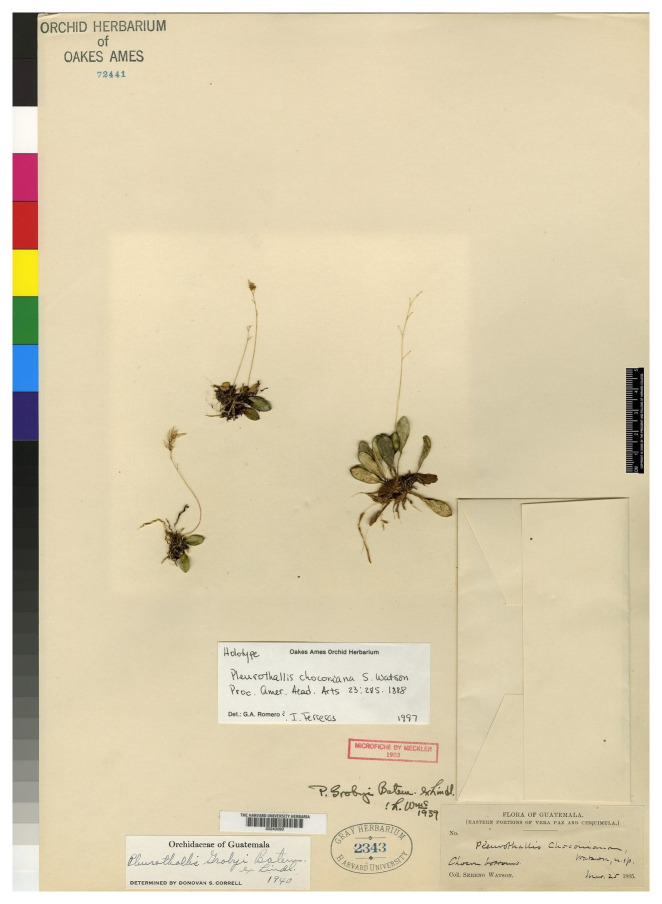
Holotype of *Pleurothallis choconiana*, *S. Watson s/n* (AMES-243090), from The Orchid Herbarium of Oakes Ames of Harvard University.

Basionym: *Pleurothallis choconiana* S.Watson, Proc. Amer. Acad. Arts, 23: 285. 1888. TYPE: GUATEMALA. [Izabal: Livingston] in the Chocón forests and at the ruins of Quirigua, 25 Mar 1885. Described from plants in bloom at Cambridge, Jul 1887, *S. Watson s.n.* (holotype: AMES-243090! (Figure 10)).

Etymology: Named after the Chocón forest, now known as the Chocón-Machacas Protected Biotope, in Izabal, Guatemala; where the type specimen was collected.

**Figure 11:**
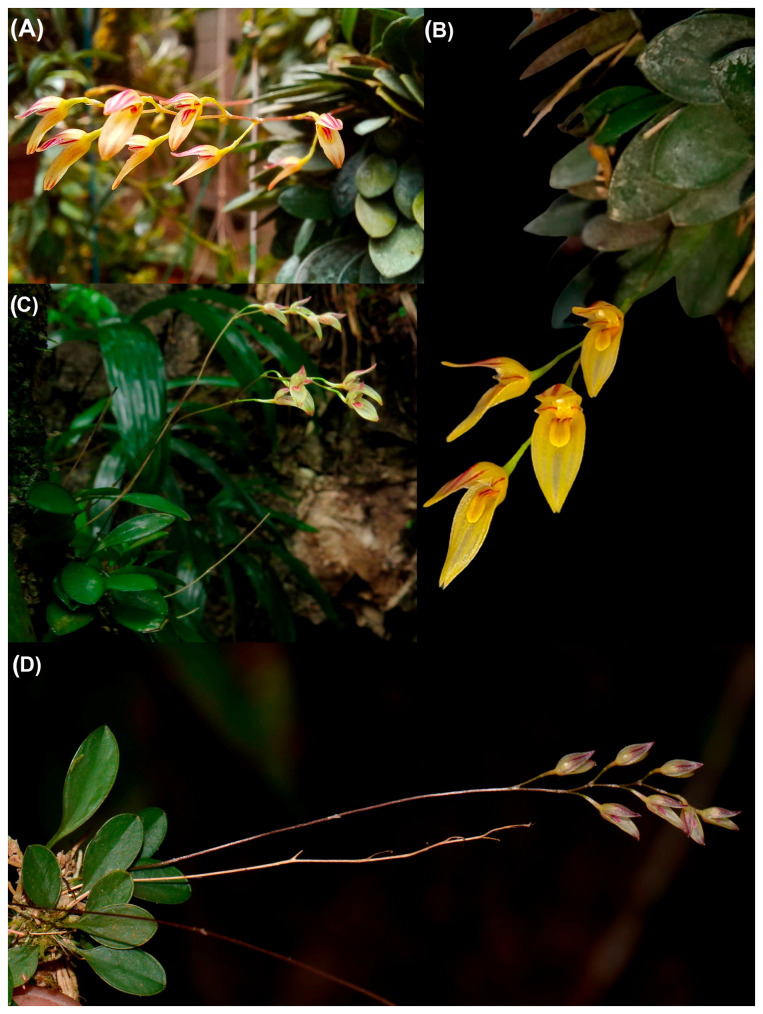
Photographs of *Specklinia choconiana*. (**A**,**B**) Specimen cultivated in Mexico City. Photographs by Ethian Licona, based on *M.A. Soto-Arenas et al., 9612* (AMO). (**C**) Specimen in its habitat, Maravilla Tenejapa, Chiapas. Photograph by Marilyn Castillo. (**D**) Specimen in its habitat, Ocosingo, Chiapas. Photograph by Gerardo Salazar, based on *G. Salazar et al. 8675* (MEXU).

**Figure 12:**
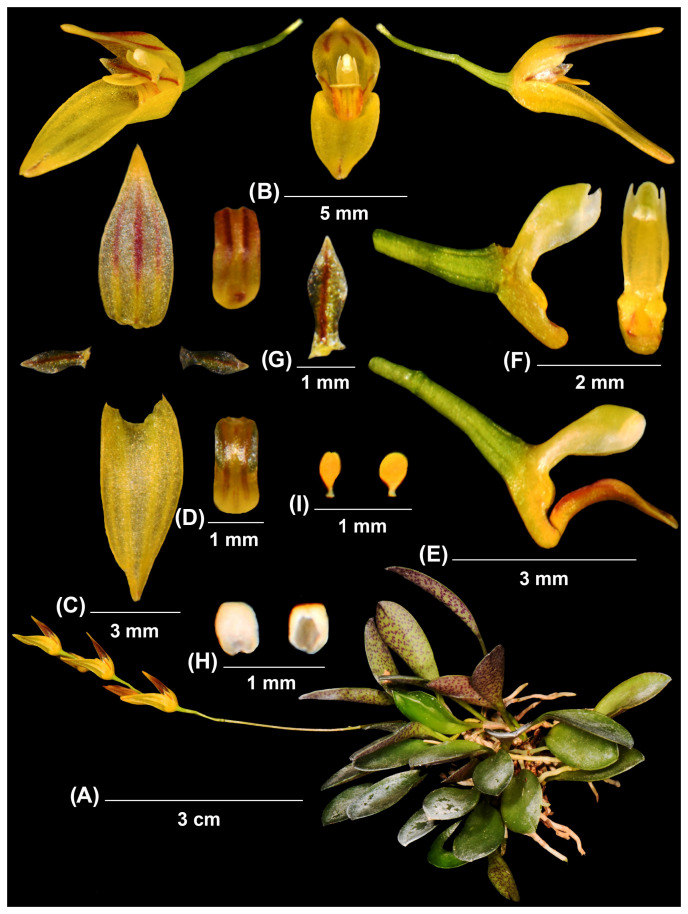
LCDP of *Specklinia choconiana*. (**A**) Habit. (**B**) Flower, ¾, frontal and lateral views. (**C**) Dissected flower. (**D**) Lip, dorsal and ventral views. (**E**) Column, lip and pedicelate ovary, lateral view. (**F**) Column, lateral and ventral views. (**G**) Petal. (**H**) Anther, dorsal and ventral views. (**I**) Pollinia, lateral and frontal views. Plate by Ethian Licona, based on *M.A. Soto Arenas et al. 9700* (AMO).

**Figure 13:**
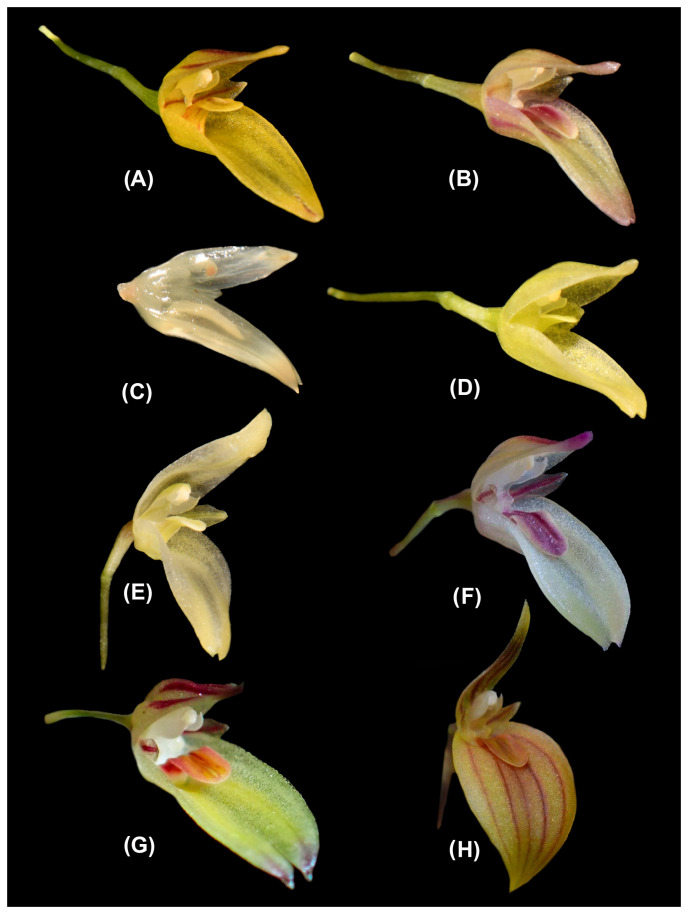
Comparison of flowers of some representatives of the *Specklinia grobyi-picta* complex, including the Mexican species. (**A**) *S. choconiana* from Ocosingo, Chiapas. (**B**) *S. marginata* from Ocosingo, Chiapas. (**C**) *S. orbiculata* from Calakmul, Campeche (in spirit). (**D**) *S. solanoi* from Ocosingo, Chiapas. (**E**) *S. xoconochcana* from Chiapas. (**F**) *S.* aff. *grobyi* from Puntarenas, Costa Rica. (**G**) *S. barbosae* (Schltr.) Luer & Toscano from São Paulo, Brazil. (**H**) *S. kakabadsei* H.Medina & J.Portilla from Esmeraldas, Ecuador. (**A**–**E**) Photographs by Ethian Licona. (**F**) Photograph by Adam Karremans. (**G**) Photographs by Rudy Gelis. (**H**) Photographs by Karol Olszowski. Images not a scale.

**Herb** cespitose, prostrate to ascending, up to 3.0 cm tall without the inflorescence. **Roots** 0.6–0.8 mm gross. **Rhizome** 1.0–3.7 mm long between adjacent stems. **Stem** 2.0–8.0 mm long, 0.6–0.8 mm gross, sheaths 1.0–6.0 mm long. **Leaf** 5.0–20.0 mm × 3.0–7.6 mm, obovate to oblanceolate, succulent, biconvex, obtuse, marginate, adaxially verrucose, abaxially glabrous, the apical mucro 0.2–0.3 mm long, light green, sometimes suffused with purple, with purple dots abaxially, abruptly attenuated into a petiole 1.0–8.0 mm long, 0.4–0.6 mm gross. **Inflorescence** 1.9–11.8 cm long, ascending, elongated, lax, glabrous; spathaceous bract 0.5–0.8 mm long; peduncle longer than the leaf, 1.3–6.5 cm long, 0.2–0.4 mm gross, with 3 bracts 0.8–1.8 mm long, glabrous, membranaceous, hyaline; rachis 0.6–5.3 cm long, with 3–12 flowers, simultaneous, distichous, sub-parallel to the rachis axis. **Floral bracts** 0.7–2.5 mm long, obliquely tubular-infundibuliform, obtuse, shortly apiculate, membranaceous, glabrous, hyaline, suffused with purple. **Flowers** 3.4–7.9 mm × 1.8–3.0 mm; sepals bright yellow to yellowish, the dorsal sepal with dark red lines, the apex of the synsepal sometimes suffused with red; petals bright yellow with a dark red line; lip bright yellow, reddish along the calli and the glenion-like depression dark red; column bright yellow, the foot suffused with red; anther white. **Sepals** concave, axially carinate, glabrous; dorsal sepal 3.0–7.0 mm × 1.0–2.9 mm, lanceolate to narrowly ovate, acuminate, 3-veined, with an apical triangular thickening, 0.8–1.0 mm × 0.2–0.3 mm; lateral sepals 3.4–7.9 mm × 1.5–3.4 mm, connate into a lanceolate to oblong–lanceolate, acuminate, shortly bifid synsepal, 4-veined, gibbose at the base, with two obliquely triangular apical thickenings, 1.0–1.3 mm × 0.2–0.3 mm. **Petals** 1.6–2.6 mm × 0.4–1.0 mm, oblong–lanceolate, acuminate, attenuated towards the base, 1-veined. **Lip** 1.8–3.0 mm × 0.6–1.3 mm, fleshy, oblong–ligulate, obtuse, 3-veined, minutely papillar-glandulous, with a pair of submarginal calli, channeled between them, with a glenion-like depression near the apex. **Column** 1.6–2.6 mm long, 0.5–1.0 mm width; wings obliquely triangular, falcate, acute, entire, 0.8–1.0 mm × 0.3–0.4 mm; clinandrium obtuse, entire; foot 0.8–1.5 mm long, 0.3–0.6 mm width, obtuse, with two elliptic, low calli in the base. **Stigmatic cavity** 0.2 mm × 0.3 mm, elliptic–oblong; rostellum laminar, oblong. **Anther** 0.4–0.6 mm long and width, globose. **Pollinia** 0.3–0.45 mm × 0.2–0.3 mm, obovoid. **Ovary** 1.0–3.1 mm long, 0.3–0.9 mm gross, obconical, straight, glabrous, yellowish-green; pedicel 1.0–6.4 mm long, 0.1–0.2 mm gross, glabrous. **Capsule** 3.6–6.4 mm long, 2.2–3.7 mm gross, elliptic–obovoid, yellowish-green suffused with purple (Figure 11, Figure 12 and Figure 13).

Distribution: Mexico, Guatemala, Belize, Honduras, El Salvador, Nicaragua and Costa Rica. In Mexico, the species is distributed in the states of Campeche, Chiapas, Oaxaca, Tabasco, Quintana Roo and Veracruz. It occurs in the Floristic Provinces of Costa del Golfo de México, Costa Pacífica, Peninsula de Yucatán, Serranías Meridionales, Serranías Transístmicas, Valle de Tehuacán and El Soconusco.

**Figure 14:**
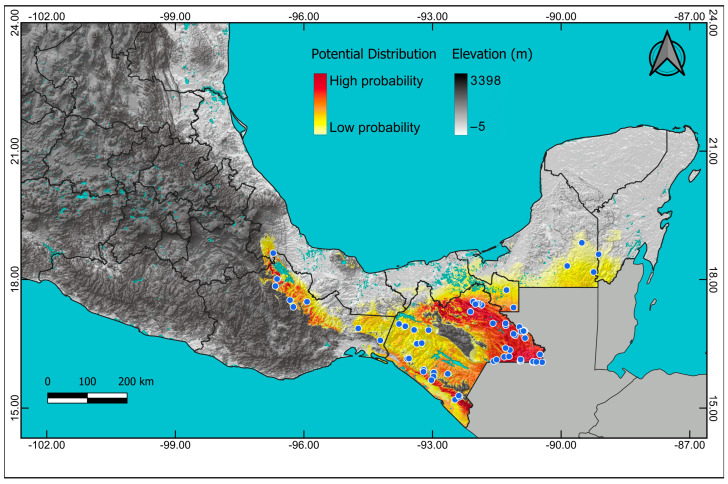
Potential distribution of *Specklinia choconiana* in Mexico and the location of currently known occurrence records (blue dots). Map by Ethian Licona.

The accepted potential distribution model for *S. choconiana* yields an AUC = 0.840, where the most influential environmental variables were temperature seasonality (33.1%), annual precipitation (15.0%) and the Digital Elevation Model of Mexico (13.7%). The potential distribution area was estimated at 114,006.78 km^2^, representing approximately 5.80% of Mexico’s total territory (Figure 14).

Habitat: Epiphyte on *Brosimum alicastrum*, *Cryosophila stauracantha* (Heynh.) R.J.Evans, *Cynometra retusa*, *Ficus* sp., *Haematoxylum campechianum* L., *Metopium brownei* (Jacq.) Urb., *Plumeria rubra* L., *Quercus* sp., *Terminalia amazonia* and *Coffea arabica* and occasionally as a lithophyte. The species grows in the evergreen tropical forest, semi-deciduous tropical forest, *Quercus* forest, thorn forest, savanna grassland, secondary vegetation derived from these forest types and coffee plantations, at elevations between 30 and 1630 m.

Phenology: Flowering occurs from October to July. Mature fruits have been observed from December to September.

Conservation status: In the assessment of its conservation status following the MER criteria [78], a score of 0.57 was obtained, therefore it is not necessary to include it in any risk category. In the evaluation under IUCN criterion B, it achieved an EOO of 181,895.063 km^2^ and an AOO of 224.00 km^2^, suggesting that the species should be classified as least concerned (LC).

Additional commentaries: *Specklinia choconiana* can be recognized by its obovate, obtuse, marginate leaves; ascending raceme, longer than the leaf, bearing distichous flowers arranged sub-parallel to the rachis axis; bright yellow flowers with red lines on the dorsal sepal; acuminate sepals with thickened apices; lanceolate dorsal sepal and synsepal; lanceolate, acuminate petals; oblong–ligulate, obtuse lip, bright yellow with red lines.

This species belongs to the *Specklinia grobyi-picta* complex [2,26,87], most of whose members have been treated as synonyms of *S. grobyi* (Bateman ex Lindl.) F.Barros, a species originally described by Lindley from Guyana. Nonetheless, *S. grobyi* is confidently known only from the northern and western Amazon forest and the Guyana shield. The morphological differences between *S. choconiana*, *S. grobyi* and the other Mexican species of this complex are summarized in Table 2.

Examined specimens: MEXICO. Chiapas: Benemérito de las Américas, Nueva Veracruz, 33 km al W del vértice del Río Chixoy, camino a Chajul, 10.01.1986, *E. Martínez 15895* (AMES!, AMO!); Nueva Veracruz, 33 km al W del vértice del Río Chixoy, camino a Chajul, 10.01.1986, *E. Martínez 15897* (MEXU!); 27 km al W del vértice del río Chixoy, camino a Chajul, 10.09.1985, *E. Martínez 13571* (AMO!, MEXU!); Nueva Orizaba, 25 km al W del vértice del río Chixoy, camino a Chajul, 22.06.1986, *E. Martínez 18880* (MEXU!); Nueva Orizaba, 25 km al W del vértice del río Chixoy, camino a Chajul, 22.06.1986. *E. Martínez 18894* (MEXU!); vértice del río Chixoy, por el camino Boca Lacantún-Chajul, 12.01.1986, *E. Martínez 16153, 16454* (MEXU!); Nueva Chihuahua, 70 km al S de Boca Lacantún, 23.06.1986, *E. Martínez 18972* (MEXU!); 6 km al E de Nueva Orizaba, camino al vértice del río Chixoy, 17.05.1987, *E. Martínez 21035* (MEXU!). Cintalapa, cruce de los ríos Negro y Venta, 1.5 km al S del río Negro, 04.07.1992, *S. Ochoa 3942 & M. Martínez* (ECOSUR-CH!). La Concordia, ejido El Rosario, 26 km al N de Puente Margaritas, sobre la carretera a Pijijiapan, polígono zona de amortiguamiento El Triunfo, 20.04.2006, *N. Martínez-Meléndez 1521* (HEM!); Finca Custepec, camino NW hacía la finca, 12.07.1990, *R. Hampshire 1245 & A. Reyes-García* (MEXU!). La Trinitaria: 21–23 km on the road between Lake Tziscao to Santa Elena, 08.11.1988, *D. Breedlove 71286 & T. Daniel* (MEXU!, MO!). Las Margaritas, km 36.5 Tziscao-Bonampak, 08.11.1993, *B. Thurston 1475 & W. Thurston* (AMO!). Motozintla: Monte Boquerón, Finca Hamburgo, above Huixtla, 12.04.1935, *O. Nagel 4364* (AMES!, MO!); Belisario Domínguez, 06.02.1999, *A. Damon s/n* (ECO-TA-H!). Ocosingo, 4 km al S de Frontera Corozal, sobre el río Usumacinta, 29.05.1985, *E. Martínez 12294 & G. Aguilar* (IEB!, MEXU!); 1.18 km al E de Frontera Corozal, 13.10.2004, *G. Aguilar 11552 & R. Arcos* (MEXU!); al NW de Frontera Corozal, 28.04.2004, *G. Aguilar 9899 & E. Martínez* (MEXU!); Monumento Natural Yaxchilán, porción N de la Omega, 22.06.1998, *M. Romero 3441* (MEXU!); entre Bonampak y el río Lacanjá, 01.04.2000, *M.A. Soto-Arenas 9612, S. Maldonado, L. López & P. Schlutter* (AMO!); 8 km antes de llegar a Lacanjá, saliendo de Chancalá, 19.07.1984, *A. Espejo 1064, M. Martínez, L. Pacheco & S. Hernández* (MEXU!); 2.5 km al SE de la colonia Benito Juárez Miramar, 22.08.1993, *A. Reyes-García 2173 & M. Sousa* (MEXU!); Nahá, 15 km al N de Monte Líbano, camino a Chancalá, 13.04.1986, *E. Martínez 18042* (MEXU!); a 600 m de Nuevo Guerrero, sobre el arroyo, 10.06.1982, *G. Aguilar 1338* (MEXU!); Nuevo Guerrero, 27.06.1986, *E. Martínez 19093* (MEXU!); Chajul, borde de la selva baja, en el área de la sabana, 30.03.2014, *G. Salazar 8675, L. Cabrera, A. Barceinas & S. Akira* (MEXU!); Estación Biológica de Chajul, junto al Río Lacantún, 15.04.2000, *M.A. Soto-Arenas 9700, S. Maldonado, L. López & P. Schluetter* (AMO!); Estación Chajul, 28.10.1992, *E. Martínez 25540, C.H. Ramos, R. Lombera & G. Domínguez* (AMO!); Estación Chajul, 08.09.1992, *E. Martínez 25279, R. Lombera, G. Domínguez & C.H. Ramos* (AMO!); 2.8 km al N de la Estación Chajul, 26.06.1999, *S. Sinaca 2722* (MEXU!); Restauración Ecológica El Cartón, 5.6 km al SW de Frontera Corozal, 22.05.2004, *G. Aguilar 10165, F. Cruz & M. Méndez* (MEXU!); Restauración Ecológica El Cartón, 5.6 km al SW de Frontera Corozal, 25.05.2004, *G. Aguilar 10234, M. Méndez & F. Cruz* (MEXU!); 1.53 km al SE de Frontera Corozal, 14.05.2004, *G. Aguilar 10037, M. Méndez & F. Cruz* (MEXU!); Reserva Zona Urbana, a 1.74 km al SE de Frontera Corozal, 29.06.2004, *G. Aguilar 10826, M. Méndez & F. Cruz* (MEXU!); 2.25 km al SE del Crucero de San Javier, 09.10.2003, *G. Aguilar 8123, A. Chambor & C. Chancayun* (MEXU!). Ocozocoautla, Emilio Rabasa, 23.05.2009, *F. Borraz 34* (HEM!); Cerro Santo, ribera Piedra Parada, 27.05.2005, *A. Gálvez 9* (HEM!). Palenque, 20 km al SE de Palenque, camino a Chancalá, 06.09.1985, *E. Martínez 13346* (MEXU!); en las cercanías de la Zona Arqueológica de Palenque, 11.05.1982, *G. Davidse 20350, M. Sousa, A. Chater & E. Cabrera* (MEXU!); 17 km al SE de Palenque, por el camino a Bonampak, ejido Doctor Samuel León Brindis, 12.05.1982, *G. Davidse 20368, M. Sousa, A. Chater & E. Cabrera* (MEXU!). Salto del Agua, 6–12 km from Palenque, along the road to Ocosingo, 23.11.1972, *D. Breedlove 29770 & R. Dressler* (MEXU!, MO!); on the top of a ridge, 15 km S from Palenque, along the road to Ocosingo, 13.12.1986, *D. Breedlove 66317, D. Mally & H. Mally* (MEXU!). Tuxtla Gutiérrez, El Sumidero, 04.07.1954, *R. Dressler 1387* (AMES!, MEXU!). Villa Corzo, Plan de Ayala, cerro Miramar, Poligono 5 El Triunfo, 28.05.1999, *F. Hernández 183* (HUAP!); ejido Nuevo Edén, polígono 5 El Triunfo, 30.03.2005, *J. Martínez 752* (HEM!, MEXU!); ejido Sierra Morena, al W del poblado, 06.08.2002, *L. Alvarado 330, A. Reyes-García, J. Galdamez & J. Aguilar* (MEXU!); ejido Sierra Morena, 31.05.2002, *A. Reyes-García 4893, D. Gómez & E. Montejo* (MEXU!). Villaflores, Roblada Grande, 07.06.1972, *T. Macdougall 485* (NYBG!). Oaxaca: San Miguel Chimalapa, en la zona del Baúl, 22.04.1967, *G. Pollard s/n* (MEXU!). San Pedro Sochiápam, San Juan Zautla, cerca del río Caracol, en la base del cerro Machín, 29.06.1939, *R. Schultes 739* (AMES!). San Pedro Teutila, sobre la brecha a Piedra Ancha, a 1.6 km en línea recta al NE del faro, 03.07.2019, *G. Juárez-García 6120, S. Salas, T. Gregory & J. Chemnick* (MEXU!). Tabasco: Balancán, Reforma, 26.05.1939, *E. Matuda 3150* (AMES!, MEXU!, MICH!). Tenosique, Rancho Atotonilco, al E del ejido El Tigre, 27.05.1988, *V. Ramón 56* (MEXU!).

Additional records: MEXICO. Campeche: Calakmul, 4.5 km al SW de El Ramonal, 08.09.2023, *B. Steven s/n* (https://www.inaturalist.org/observations/185065731. Accessed 4 August 2025). Chiapas: Ángel Albino Corzo, Cascada El Pajal, 02.04.2024, *I. Merchan s/n* (https://www.inaturalist.org/observations/213208984. Accessed 4 August 2025). Chilón, cascadas de Agua Azul, 28.05.2022, *S. Søndergaard s/n* (https://www.inaturalist.org/observations/119422650. Accessed 4 August 2025). Huixtla, Piedra de Huixtla, 29.12.2022, *A. López s/n* (https://www.inaturalist.org/observations/155716485. Accessed 4 August 2025). Maravilla Tenejapa, cerca de Gallo Giro, 02.06.2019, *M. Castillo s/n* (https://www.inaturalist.org/observations/26901414. Accessed 4 August 2025). Ocozocoautla, Cerro Brujo, 19.01.2020, *E. Martínez Ovando s/n* (Photograph); El Cerebro, al N de La Ventosa, 30.06.2024, *L. Rivera s/n* (https://www.inaturalist.org/observations/226344671. Accessed 4 August 2025). Palenque, cerca del museo de sitio de la Zona Arqueológica de Palenque, 10.09.2018, *M. Hernández s/n* (https://www.inaturalist.org/observations/16398468. Accessed 4 August 2025; Sendero Motiepá, 17.03.2021, *S. van Belle s/n* (https://www.inaturalist.org/observations/72811458). Pijijiapan, al E de Plan de Ayala, 23.01.2022, *G. Martínez s/n* (https://www.inaturalist.org/observations/105679571. Accessed 4 August 2025). Tonalá, al W de Sierra Morena, 19.04.2025, *M. Lombard s/n* (https://www.inaturalist.org/observations/316519297. Accessed 4 August 2025). Oaxaca: Ixtlán de Juárez, San Miguel Tiltepec, 03.06.2023, *C. Tomás-López s/n* (Photograph). San Juan Lalana, Asunción Lacova, 01.01.2022, *M. González s/n* (Photograph). Villa Talea de Castro, al NE de Talea, 21.02.2022, *C. Tomás-López s/n* (Photograph). San Miguel Chimalapa, al E de San Antonio, 06.07.2025, *F. Wildtruth s/n* (https://www.inaturalist.org/observations/296956607. Accessed 4 August 2025). Quintana Roo: Othón P. Blanco, ejido Hermenegildo Galeana, 17.04.2016, *L. Ibarra s/n* (Photograph). Veracruz: Tezonapa, cerca de Tezonapa, *Á. Andrade s/n* (Photograph).

4. *Specklinia digitale* (Luer) Pridgeon & M.W.Chase, Lindleyana, 16 (4): 257. 2001.

**Figure 15:**
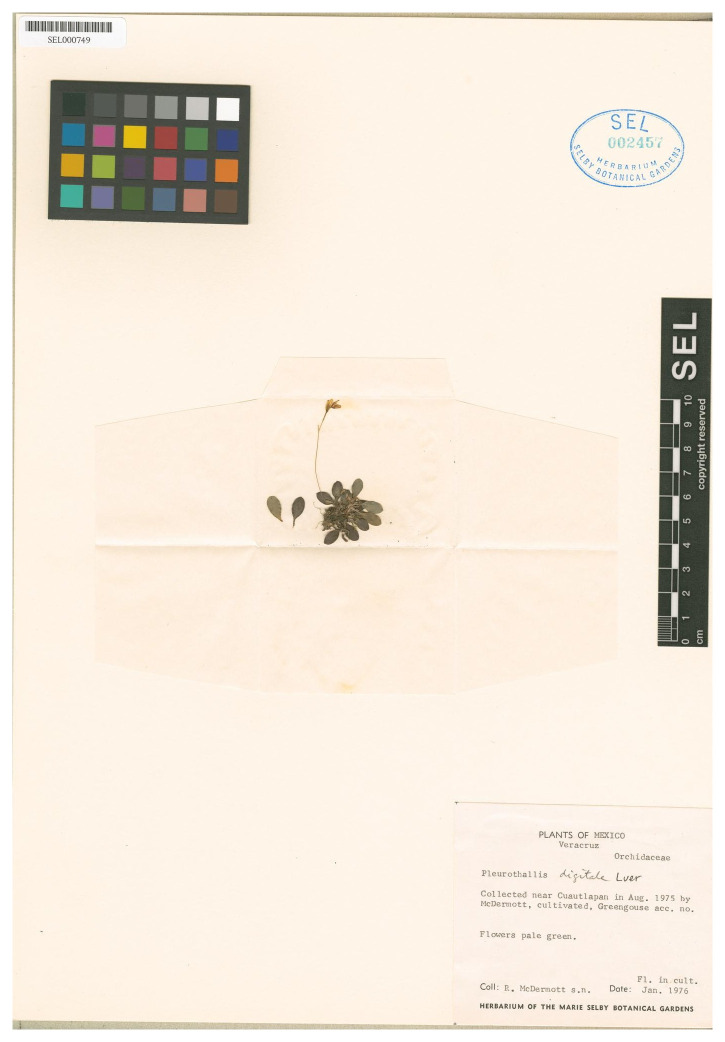
Holotype of *Pleurothallis digitale*, *R. McDermott s/n* (SEL-749), courtesy of the Marie Selby Botanical Gardens.

Basionym: *Pleurothallis digitale* Luer, Orquídea (Mexico City) n.s., 6 (1): 3–4. 1976. TYPE: MEXICO. Veracruz: [Totutla] Cuautlalpan, Aug. 1975, *R. McDermott s.n.* (holotype: SEL-749! (Figure 15); isotype: MEXU-198820!).

Etymology: From the Latin *digitalis*, “with the shape of a finger”, referring to the calyptra in the apex of the dorsal sepal.

**Figure 16:**
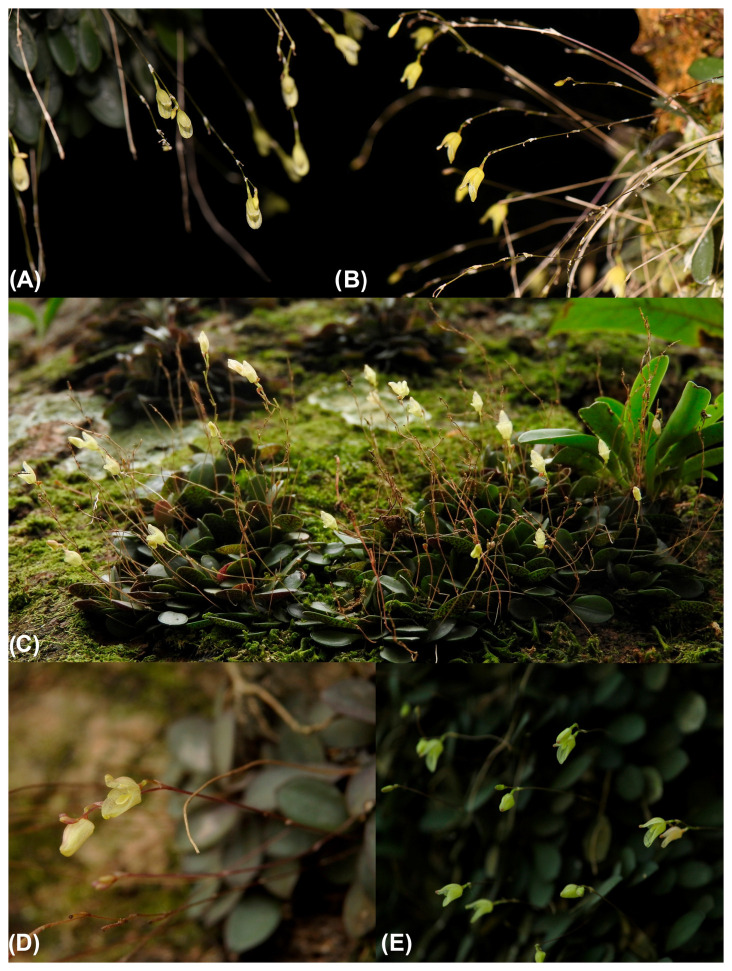
Photographs of *Specklinia digitale*. (**A**,**B**) Specimen cultivated in Mexico City. Photographs by Ethian Licona, based on *L. Sánchez et al., 311* (AMO). (**C**) Specimen in its habitat, Atoyác, Veracruz. (**D**) Specimen in its habitat, Córdoba, Veracruz. (**C**,**D**) Photographs by Victor de la Cruz. (**E**) Specimen in its habitat, Zongolica, Veracruz. Photograph by Gerardo Torres.

**Figure 17:**
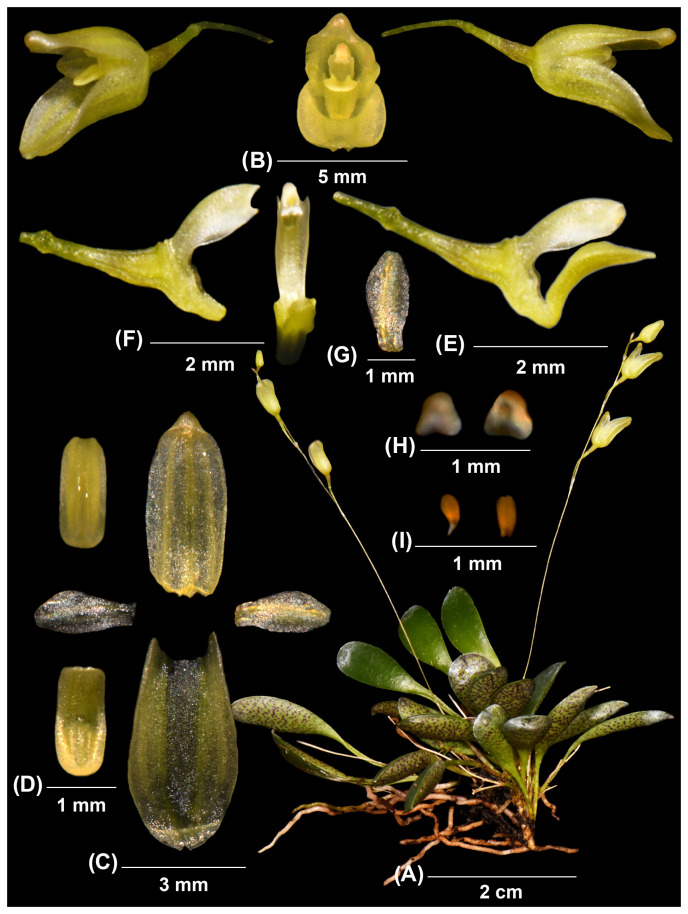
LCDP of *Specklinia digitale*. (**A**) Habit. (**B**) Flower, ¾, frontal and lateral views. (**C**) Dissected flower. (**D**) Lip, dorsal and ventral views. (**E**) Column, lip and pedicelate ovary in lateral view. (**F**) Column, lateral and ventral views. (**G**) Petal. (**H**) Anther, dorsal and ventral views. (**I**) Pollinia, lateral and frontal views. Plate by Ethian Licona, based on *L. Sánchez et al., 311* (AMO).

**Figure 18:**
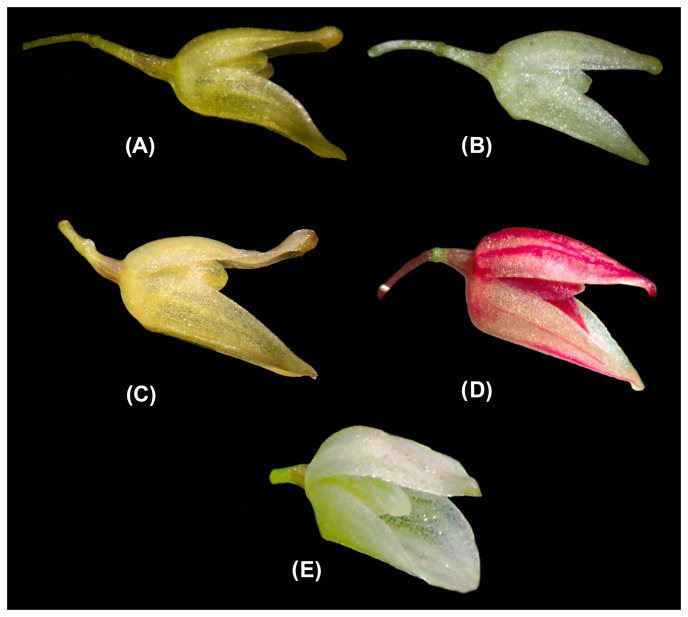
Comparison of flowers among the species belonging to the *Specklinia digitale* complex. (**A**) *S. digitale* from Totutla, Veracruz. (**B**) *S. microphylla* from Santa María Chimalapa, Oaxaca. (**C**) *S. perez-garciae* from Asunción Ixtaltepec, Oaxaca. (**D**) *S. pisinna* from Ocosingo, Chiapas. (**E**) *S. lugduno-batavae* from Río San Juan, Nicaragua. (**A**,**C**) Photographs by Ethian Licona. (**B**) Photograph by Daniel Cornú. (**D**) Photograph by Rodolfo Solano. (**E**) Photograph by Sune Holt. Images not a scale.

**Herb** cespitose, prostrate to ascending, up to 2.8 cm tall without the inflorescence. **Roots** 0.4–0.7 mm gross. **Rhizome** 0.7–3.0 mm long between adjacent stems. **Stem** 1.5–10.0 mm long, 0.3–0.5 mm gross, sheaths 1.7–5.0 mm long. **Leaf** 5.0–19.0 mm × 3.4–7.0 mm, elliptic–obovate to obovate, obtuse, succulent, biconvex, marginate, adaxially verrucose, abaxially glabrous, the apical mucro 0.2–0.3 mm long, light green suffused with purple, with purple dots abaxially, abruptly attenuated into a petiole 1.0–7.5 mm long, 0.3–0.5 mm gross. **Inflorescence** 2.5–10.8 cm long, elongated, lax, flexuous, glabrous; spathaceous bract 0.3–0.6 mm long; peduncle longer than the leaf, 1.3–4.8 cm long, 0.1–0.2 mm gross, with 2 bracts 2.0–2.6 mm long, glabrous, membranaceous, hyaline; rachis 1.2–6.0 cm long, with 3–10 flowers, successive, secund, 1–3 open at a time. **Floral bracts** 1.0–2.5 mm long, obliquely tubular-infundibuliform, obtuse, shortly apiculate, membranaceous, glabrous, hyaline suffused with purple. **Flowers** 3.8–5.9 mm × 2.0–3.0 mm; sepals and petals cream, the apex of the dorsal sepal pale pink; lip and column yellowish; anther cream suffused with pale pink. **Sepals** concave, axially carinate, glabrous; dorsal sepal 4.0–5.5 mm × 1.9–2.5 mm, oblong–elliptic to oblong–ovate, acute, 3-veined, the apex forming a calyptra, papillar-glandulous inside, 0.5–0.7 mm × 0.5–0.8 mm; lateral sepals 4.1–5.9 mm × 2.0–3.3 mm, connate into an ovate to oblong–elliptic, obtuse, shortly bifid synsepal, 4-veined, gibbose in the base. **Petals** 2.0–2.4 mm × 0.8–1.1 mm, obliquely obovate, obtuse, slightly falcate, 1-veined. **Lip** 1.7–2.2 mm × 0.6–1.1 mm, fleshy, oblong–ligulate, obtuse, 3-veined, minutely papillar-glandulous, with a pair of submarginal calli, channeled between them. **Column** 1.7–2.5 mm long, 0.6–1.0 mm width; wings narrowly triangular, acute, entire, 0.3–0.5 mm × 0.2–0.4 mm; clinandrium obtuse, entire; foot 0.9–1.7 mm long, 0.3–0.6 mm width, obtuse, with two globular, high calli at the base. **Stigmatic cavity** 0.25 mm × 0.3 mm, elliptic–oblong; rostellum laminar, oblong. **Anther** 0.3–0.5 mm long and width, ovoid. **Pollinia** 0.25–0.35 mm × 0.2–0.3 mm, obovoid. **Ovary** 1.5–2.4 mm long, 0.4–0.6 mm gross, obconical, slightly recurved, glabrous, yellowish-green, suffused with magenta; pedicel 1.5–4.3 mm long, 0.1–0.2 mm gross, glabrous. **Capsule** 3.0–4.0 mm long, 1.7–2.3 mm gross, elliptic–obovoid, yellowish-green, suffused with purple (Figure 16, Figure 17 and Figure 18).

Distribution: Endemic. The species is known only from the mountains in central Veracruz. It occurs in the Floristic Provinces of Serranías Meridionales and Valle de Tehuacán.

**Figure 19:**
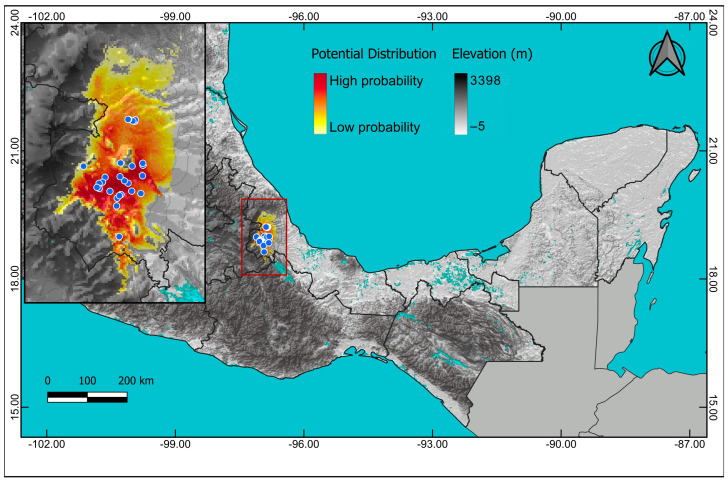
Potential distribution of *Specklinia digitale* in Mexico and the location of currently known occurrence records (blue dots). Map by Ethian Licona.

The accepted potential distribution model for *S. digitale* yields an AUC = 0.943, where the most influential environmental variables were the Digital Elevation Model of Mexico (42.5%), isothermality (31.8%), and precipitation of the wettest month (9.5%). The potential distribution area was estimated at 4085.60 km^2^, representing approximately 0.21% of Mexico’s total territory (Figure 19).

A specimen apparently collected in Ocosingo, Chiapas (*R. Dressler 1432*, AMES!) resembles *S. digitale* but is larger than the plants known from Veracruz. No other specimens with these characteristics have been documented from that region; therefore, this record is likely the result of a labeling error.

Habitat: Epiphyte on *Liquidambar styraciflua* L. and *Quercus* sp. It is also commonly found growing on *Citrus × sinensis* (L.) Osbeck, *Coffea arabica* and *Mangifera indica* L., and occasionally as a lithophyte. The species grows in the evergreen tropical forest, semi-deciduous tropical forest, *Quercus* forest, secondary vegetation derived from these types of forest, coffee plantations and citrus, banana, mango and tamarind orchards as well as in urban parks, at elevations between 560 and 1500 m.

Phenology: Flowering occurs from September to July. Mature fruits have been observed from December to October.

Conservation status: This species is currently listed as Threatened (A) under NOM-059-SEMARNAT-2010 [78]. However, a reassessment of its conservation status following the MER criteria [78], yielded a score of 1.63, indicating that it should be instead classified as a species Under Special Protection (Pr). In the evaluation under IUCN criterion B, it achieved an EOO of 1141.913 km^2^ and an AOO of 96.00 km^2^, which suggests that this species should be classified as Endangered (EN).

Additional commentaries: *Specklinia digitale* can be recognized by its prostrate habit; obovate, obtuse, marginate leaves; inflorescence longer than the leaf, lax and flexuous, with cream-colored, successive flowers, 1–3 open at a time; ovate dorsal sepal with a deeply calyptrate apex; oblanceolate, obtuse and slightly bifid synsepal; obliquely obovate, obtuse petals; and an oblong–ligulate lip.

This species gives its name to a distinct species complex, that, in addition to *S. digitale*, includes *S. lugduno-batavae* Karremans, Bogarín & Gravend., *S. microphylla*, *S. perez-garciae* and *S. pisinna*. Most members of this complex are known only from Mexico, although two extended their distribution to Honduras and Panama. The morphological differences among *S. digitale* and the remaining species of this complex are summarized in Table 3.

Examined specimens: MEXICO. Veracruz: without locality, 01.01.1907, *C. Purpus s/n* (AMES!). Amatlán de los Reyes, La Caldera del Diablo, cerca del poblado de Santa Ana, 23.01.1993, *R. Solano 604, R. Jiménez, L. Sánchez & J. Arguijo* (AMO!). Atoyac, Sierra de Atoyac, 1–4 km al NW de Atoyac, 07.12.1992, *P. Hietz 399* (XAL!). Ixtaczoquitlán, Barranca de Metlác, 13.05.1992, *M. López 125* (AMO!); 3 km W of Fortín, on the side of a deep canyon along the San Antonio river, 03.08.1977, *E. Lehto 22022* (ASU!). Totutla, Zacuapan, Barranca de Zacuapan, 17.05.1994, *L. Sánchez 311, A.R. López-Ferrari, A. Espejo & J. García-Cruz* (AMO!); Zacuapan, Barranca de Zacuapan, 17.05.1994, *L. Sánchez 312, A.R. López-Ferrari, A. Espejo & J. García-Cruz* (AMO!); Virgin forest near Zacuapan, 12.02.1932, *O. Nagel sub Oestlund 2660* (AMES!); near Zacuapan, 07.08.1937, *C. Purpus 5071* (AMES!); Zacuapan, 01.03.1938, *C. Purpus 16258* (AMES!); Hacienda de Zacuapan, near Huatusco, 01.01.1913, *C. Purpus 6387* (AMES!, GH!, NYBG!, UC!); Rancho Zacuapan, a 27 km de Huatusco, hacía Conejos, a 2 km de Mata Obscura, 05.07.1987, *I. Aguirre 1263 & G. Salazar* (AMO!); El Mirador, *F. Ventura 8218* (AMO!, MEXU!). El Mirador, 03.07.1979, *F. Ventura 16286* (AMO!).

Additional records: MEXICO. Veracruz: Amatlán de los Reyes, Aquiojapa, 01.05.2021, *M. Murillo s/n* (https://www.inaturalist.org/observations/80996451. Accessed 3 July 2025); Chilpanapa, 01.05.2020, *D. Osorno s/n* (https://www.inaturalist.org/observations/45095240. Accessed 3 July 2025); Manuel León, 01.07.2020, *V. de la Cruz s/n* (https://www.inaturalist.org/observations/52944078. Accessed 3 July 2025); al NW de La Joya, 15.03.2024, *O. Morgado s/n* (https://www.inaturalist.org/observations/202835134. Accessed 3 July 2025). Atoyac, al W de Colonia Cabrera, 01.12.2020, *V. de la Cruz s/n* (https://www.inaturalist.org/observations/66897335. Accessed 3 July 2025). Chocamán, al W de Tepexilotla, 28.12.2020, *V. de la Cruz s/n* (https://www.inaturalist.org/observations/67236554. Accessed 3 July 2025). Córdoba, al N de El Porvenir, 01.04.2021, *V. de la Cruz s/n* (https://www.inaturalist.org/observations/74671058. Accessed 3 July 2025); al W de Rancho Limón, 01.03.2021, *V. de la Cruz s/n* (https://www.inaturalist.org/observations/72338720. Accessed 3 July 2025); El Bajío, al O de Matlaquiahuitl, 01.12.2022, *A. Lara s/n* (https://www.inaturalist.org/observations/143618321. Accessed 3 July 2025). Fortín, al E de Villa Unión, 01.04.2022, *I. Aguirre s/n* (https://www.inaturalist.org/observations/112565588. Accessed 3 July 2025); La Huerta, 01.10.2022, *A. Flores s/n* (https://www.inaturalist.org/observations/141562438. Accessed 3 July 2025). Ixhuatlán del Café, al N de San José de Los Naranjos, 01.12.2020, *V. de la Cruz s/n* (https://www.inaturalist.org/observations/66951210. Accessed 3 July 2025). Ixtaczoquitlán, Nahuapan, 01.06.2021, *V. de la Cruz s/n* (https://www.inaturalist.org/observations/82172971. Accessed 3 July 2025). Naranjal, El Rincón de Axalpa, 30.08.2015, *A. Fernández-Díaz s/n & C. Huerta-Alvizar* (Photograph). Paso del Macho, al W de La Unión de los Reyes, 01.03.2023, *P. Molina s/n* (https://www.inaturalist.org/observations/152873326. Accessed 3 July 2025). Yanga, al NW de El Pochote, cruzando el río Jamapa, 22.12.2020, *V. de la Cruz s/n* (https://www.inaturalist.org/observations/66951126. Accessed 3 July 2025). Zongolica, al S de Ameyalco, 01.02.2021, *G. Torres s/n* (https://www.inaturalist.org/observations/70974560. Accessed 3 July 2025); al NW de Coyametla, 01.03.2025, *D. Sevilla s/n* (https://www.inaturalist.org/observations/265862572. Accessed 3 July 2025).

5. *Specklinia marginata* (Lindl.) Pridgeon & M.W.Chase, Lindleyana, 16 (4): 258. 2001.

**Figure 20:**
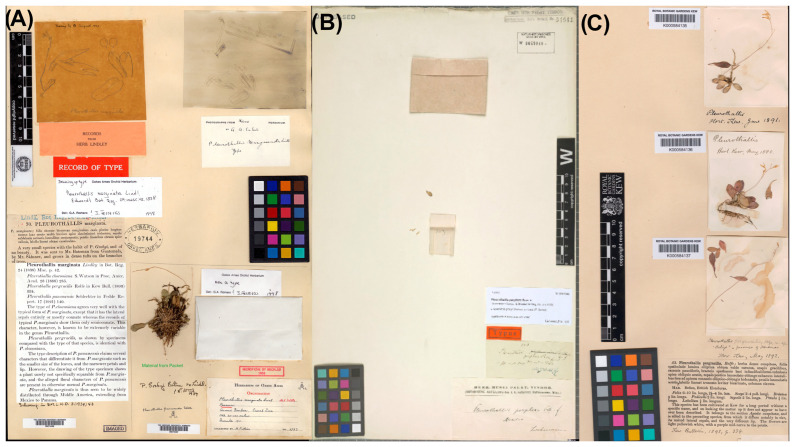
(**A**) Specimen selected as lectotype of *Pleurothallis marginata*, *U.R. Skinner s/n* (AMES-74466), from The Orchid Herbarium of Oakes Ames of Harvard University. (**B**) Holotype of *Pleurothallis perplexa*, *F. Liebmann 133* (W-49046), courtesy of the Herbarium of the Naturhistorisches Museum Wien. (**C**) Specimen selected as lectotype of *Pleurothallis pergracilis*, *Anon. s/n* (K-584135, http://specimens.kew.org/herbarium/K000584135. Accessed 11 June 2025), © The Board of Trustees-RBG, Kew.

Basionym: *Pleurothallis marginata* Lindl., Edward’s Bot. Reg., 24: Misc. 42. 1838, non *P. marginata* (Rich.) Cogn., *nom. illeg.* (=*Andreettaea marginata* (Rich.) A.Doucette). TYPE: GUATEMALA. “*It was sent to Mr. Bateman from Guatemala, by Mr. Skinner*”. *U.R. Skinner s.n.* (holotype: K (not found), lectotype here designated: Photographs and illustration by A.A. Eaton based on the type, AMES-74466! (Figure 20A)).

Homotypic synonym: Humboldtia marginata (Lindl.) Kuntze, Revis. Gen. Plant. 2: 668. 1891.

Etymology: From the Latin *marginatus*, “margined”; probably referring to the margined leaves or the lines on the sepals.

Heterotypic synonyms: *Pleurothallis perplexa* Rchb.f., in Hamb. Garten. 16: 15. 1860. TYPE: MEXICO. Veracruz: [Totutla] Mirador. Herrn Consul Schiller eingeführt, cultivirt von Herrn Stange. *F. Liebmann 133* (holotype: W-49046! (Figure 20B)).

Etymology: From the Latin *perplexus*; “perplexed or confused”; although there is no explanation in the protologue, probably refers to the unawareness of the author about similar species to the one he described.

*Pleurothallis pergracilis* Rolfe, Bull. Misc. Inform. Kew 1893 (82–83): 334. 1893. TYPE: BELIZE. “*Cultivated at Kew since 1887, when it was received from the Belize Estate and Produce Company*”. *Anon s.n.* (lectotype here designated: K-584135! (Figure 20C); isolectotype; drawing of the type by Rolfe, AMES-74607!).

Etymology: From the Latin *pergracilis*, “very thin”, referring to the appearance of the inflorescence.

**Figure 21:**
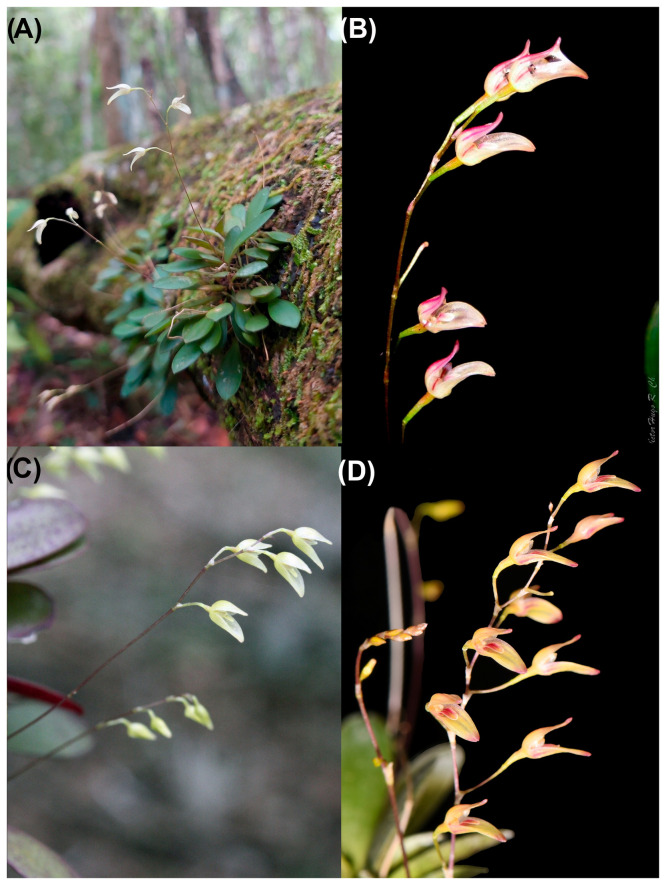
Photographs of *Specklinia marginata*. (**A**) Specimen in its habitat, Calakmul, Campeche. Photograph by Carlos Delgado. (**B**) Specimen in its habitat, Palenque, Chiapas. Photograph by Victor Chaparro. (**C**) Specimen in its habitat, Othón P. Blanco, Quintana Roo. Photograph by León Ibarra. (**D**) Specimen cultivated in Mexico City, from Ocosingo, Chiapas. Photograph by Ethian Licona.

**Figure 22:**
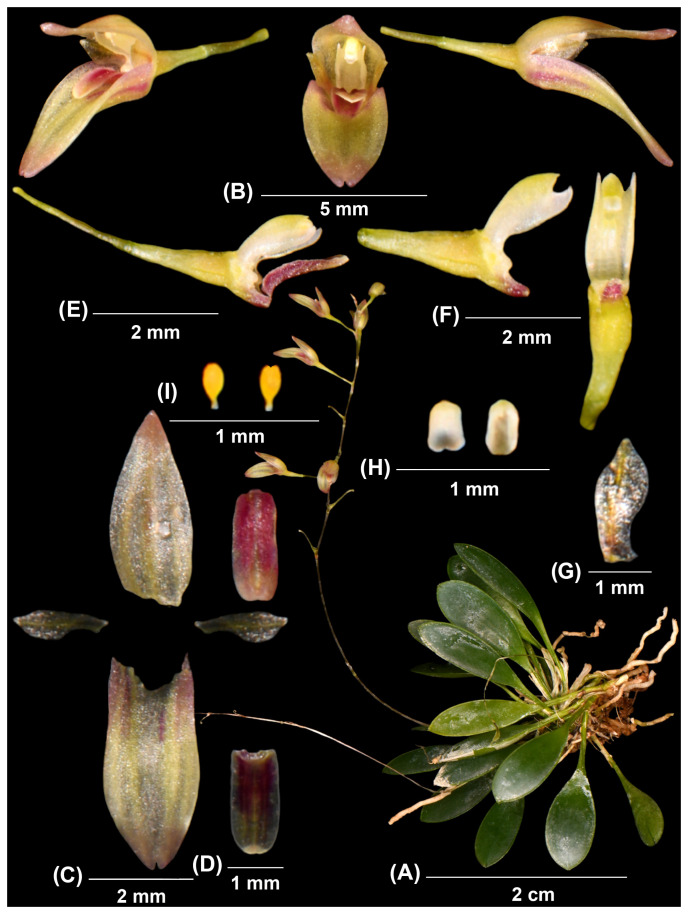
LCDP of *Specklinia marginata*. (**A**) Habit. (**B**) Flower, ¾, frontal and lateral views. (**C**) Dissected flower. (**D**) Lip, dorsal and ventral views. (**E**) Column, lip and pedicelate ovary in lateral view. (**F**) Column, lateral and ventral views. (**G**) Petal. (**H**) Anther, dorsal and ventral views. (**I**) Pollinia, lateral and frontal views. Plate by Ethian Licona, based on a specimen from Ocosingo, Chiapas, cultivated in the AMO herbarium.

**Herb** shortly rhizomatous, prostrate to ascending, up to 3.0 cm tall without the inflorescence. **Roots** 0.5–0.8 mm gross. **Rhizome** 1.0–3.5 mm long between adjacent stems. **Stem** 2.0–8.8 mm long, 0.5–0.7 mm gross, sheaths 1.4–5.0 mm long. **Leaf** 5.0–18.0 mm × 3.0–7.6 mm, elliptic to elliptic–oblanceolate, acute, succulent, biconvex, marginate, adaxially verrucose, abaxially glabrous, the apical mucro 0.3–0.4 mm long, light green, sometimes suffused with purple, with purple dots abaxially, abruptly attenuated into a petiole 1.5–9.0 mm long, 0.3–0.5 mm gross. **Inflorescence** 2.6–12.8 cm long, erect, elongated, lax, glabrous; spathaceous bract 0.4–0.8 mm long; peduncle longer than the leaf, 1.7–8.0 cm long, 0.2–0.3 mm gross, with two bracts 1.4–2.0 mm long, glabrous, membranaceous, hyaline; rachis 0.9–4.8 cm long, with 3–12, simultaneous, secund flowers, perpendicular to the rachis axis. **Floral bracts** 0.7–2.0 mm long, obliquely tubular-infundibuliform, obtuse, shortly apiculate, membranaceous, glabrous, hyaline suffused with purple. **Flowers** 3.2–5.5 mm × 1.5–2.6 mm; sepals white to dull yellow, sometimes with pink to magenta lines, the apices suffused with pink; petals white to dull yellow, sometimes with a pink line; lip magenta with the apex turning white, the calli darker and the glenion-like depression yellowish; column yellowish-white, foot magenta; anther white. **Sepals** concave, axially carinate, glabrous; dorsal sepal 3.0–4.8 mm × 1.0–2.0 mm, lanceolate to narrowly ovate, acute, 3-veined, with a triangular to lanceolate apical thickening, 0.8–1.0 mm × 0.3–0.4 mm; lateral sepals 3.2–5.5 mm × 1.6–2.7 mm, connate into an oblong–elliptic to oblong–lanceolate, acute, shortly bifid synsepal, 4-veined, gibbose in the base, with two obliquely oblanceolate to oblong–elliptic apical thickenings, 0.8–1.1 mm × 0.3–0.4 mm. **Petals** 1.6–2.2 mm × 0.5–0.7 mm, obliquely obovate, acuminate, falcate, attenuated towards the base, 1-veined. **Lip** 1.7–2.1 mm × 0.5–0.9 mm, fleshy, oblong–ligulate, obtuse, 3-veined, minutely papillar-glandulous, with a pair of submarginal calli, channeled between them, with a glenion-like depression near the apex. **Column** 1.7–2.0 mm long, 0.5–0.9 mm width; wings obliquely triangular, falcate, acute, entire, 0.5–0.7 mm × 0.3–0.4 mm; clinandrium obtuse, entire; foot 0.8–1.0 mm long, 0.3–0.5 mm width, acute, with two globular, high calli at the base. **Stigmatic cavity** 0.2 mm × 0.3 mm, elliptic–oblong; rostellum laminar, oblong. **Anther** 0.4–0.5 mm long, 0.3–0.4 mm width, elliptic–ovoid. **Pollinia** 0.25–0.3 mm × 0.2–0.25 mm, elliptic–obovoid. **Ovary** 0.8–2.8 mm long, 0.3–1.0 mm gross, obconical, straight, glabrous, yellowish-green suffused with purple; pedicel 1.0–4.4 mm long, 0.1–0.2 mm gross, glabrous. **Capsule** 4.5–5.4 mm long, 2.0–3.0 mm gross, elliptic–obovoid, yellowish-green suffused with purple (Figure 21 and Figure 22).

Distribution: Mexico, Guatemala, Belize, Honduras, El Salvador, Nicaragua and Costa Rica. In Mexico, the species is distributed in the states of Campeche, Chiapas, Oaxaca, Tabasco, Quintana Roo and Veracruz. It occurs in the Floristic Provinces of Costa del Golfo de México, Costa Pacífica, Península de Yucatán, Serranías Meridionales, Sierra Madre Oriental, Valle de Tehuacán and El Soconusco.

**Figure 23:**
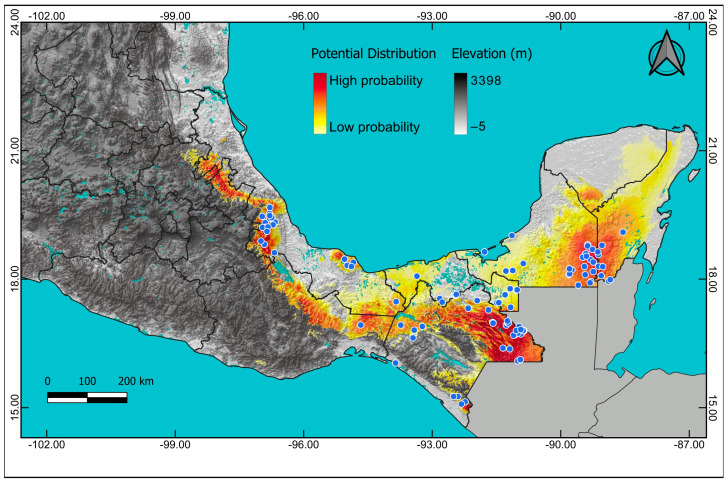
Potential distribution of *Specklinia marginata* in Mexico and the location of currently known occurrence records (blue dots). Map by Ethian Licona.

The accepted potential distribution model for *S. marginata* yields an AUC = 0.849, where the most influential variables were precipitation seasonality (21.9%), the Digital Elevation Model of Mexico (21.5%) and temperature seasonality (13.9%). The potential distribution area was estimated at 159,241.98 km^2^, representing approximately 8.11% of Mexico’s total territory (Figure 23).

Habitat: Epiphye on *Byrsonima spicata* (Cav.) Rich. ex Kunth, *Cordia megalantha* UNDATEDBlake, *Cornutia pyramidata* L., *Crescentia* sp., *Cryosophila stauracantha*, *Haematoxylum campechianum*, *Manilkara zapota* (L.) P.Royen, *Metopium brownei*, *Pachira aquatica* Aubl., *Platymiscium* sp., *Plumeria rubra*, *Quercus* sp., *Rhizophora mangle* L., *Spondias mombin* L. and *Yucca* sp. also frequently found on *Coffea arabica*, *Citrus x sinensis* and *Theobroma cacao* L., and occasionally as a lithophyte. The species grows in the evergreen tropical forest, semi-deciduous tropical forest, *Quercus* forest, thorn forest, mangrove forest, riparian vegetation, savanna grassland, secondary vegetation derived from these forest types, coffee plantations and citrus and cacao orchards, at elevations between 1 and 1600 m elev.

Phenology: Flowering occurs throughout the year. Mature fruits have been observed year-round.

Conservation status: In the assessment of its conservation status following the MER criteria [78], a score of 0.46 was obtained, therefore it is not necessary to include it in any risk category. While in the assessment for the B criterion of the IUCN, it achieved an EOO of 285,290.042 km^2^ and an AOO of 392.00 km^2^, which suggests that this species should be considered Least Concerned (LC).

Additional commentaries: *Specklinia marginata* can be recognized by its elliptic, acute, marginate leaves; raceme erect, longer than the leaf, bearing secund, yellowish-white flowers, that are concolorous or marked with pink-magentas lines, arranged perpendicular to the rachis; acute sepals, dorsal sepal ovate, synsepal oblong–lanceolate; obliquely obovate, falcate, acuminate petals; oblong–ligulate, obtuse, magenta lip with the apex white; and white column with a magenta foot.

This species has been long confused with *Specklinia grobyi*, having been treated as one of its multiple synonyms. In Mexico it was traditionally considered as the sole representative of the *S. grobyi* complex. Here, it is recognized that this complex in Mexico encompasses several morphologically similar species, including *S. choconina*, *S. marginata*, *S. orbiculata*, *S. solanoi* and *S. xoconochcana*. The diagnostic morphological differences among these species are summarized in Table 2.

The specimen corresponding to *H. Wendland 258*, deposited in GOET, is commonly cited as the type material of *Pleurothallis marginata*. However, this specimen does not correspond to the original material upon which the species description was based.

Examined specimens: MEXICO. Campeche: Calakmul, Ejido Nueva Vida, al N de Zoh Laguna, 17.04.1995, *M.A. Soto Arenas 7700, E. Martínez & G. Tavera* (AMO!); Jardín Botánico de Zoh-Laguna, 05.05.1997, *E. Martínez 27096, P. Álvaro & F. Trejo* (MEXU!); 25 km al S de Xpujil, por la carretera que va a la laguna Alvarado y Justo Sierra, al S de Ingeniero Ricardo Payro Jene, 05.05.1998, *G. Carnevali 5339 & I. Ramírez* (CICY!); 7 km al SE de Dos Naciones, 03.04.1998, *E. Martínez 30504, D. Álvarez & B. Sanders* (MEXU!); 10 km al SE de Dos Naciones, camino a El Cibalito, 17.05.1997, *E. Martínez 27195, D. Álvarez & P. Álvaro* (MEXU!); 10 km al SE de Dos Naciones, camino a El Cibalito, 17.05.1997, *E. Martínez 27210, D. Álvarez & P. Álvaro* (MEXU!, XAL!); 4 km al S de Ejido Nueva Vida, camino a Xpujil, 13.05.1997, *E. Martínez 27163 & P. Álvaro* (MEXU!); 1.2 km al NE del poblado San Miguel, 08.07.2003, *D. Álvarez 5977* (MEXU!). Carmen, km 84 carretera Champotón-Ciudad del Carmen, 10.10.2004, *J. Viana 4732, J. Meza, F. Salazar & J. Bonilla* (HUMO!); brazo del río Candelaria, Conquista Campesina, 12.08.2015, *A. de la Cruz 1215, A. Anacleto & F. Izquierdo* (UJAT!). Chiapas: Cintalapa, Reserva El Ocote, 14.04.1993, *T. Cabrera 198 & C. Beutelspacher* (ECOSUR-CH!). Las Margaritas, near the confluence of the Perlas and Jataté rivers, in San Quintín, near laguna Miramar, 21.03.1955, *E. Sohns 1675* (MICH!). Motozintla, km 20 Huixtla-Motozintla, 01.01.1973, *G.E. Pollard s/n* (AMO!). Ocosingo, Bonampak, in the road to the river, W extreme of the ruins, 18.03.1975, *W. Hoover 286* (MO!); 4 km al SE de Frontera Corozal, sobre el río Usumacinta, 29.05.1985, *E. Martínez 12294 & C. Aguilar* (IEB!, MEXU!); Crucero Corozal, 03.05.2004, *G. Aguilar 9931 & E. Martínez* (IEB!); Crucero Corozal, sobre el camino Palenque-Boca Lacantún, 25.04.1986, *E. Martínez 17891* (MEXU!); 10 km al SE de Crucero Corozal, sobre la carretera fronteriza del sur, 22.01.1985, *E. Martínez 10036 & C. Aguilar* (MEXU!); 2 km al S de Crucero Corozal, sobre el camino Palenque-Boca Lacantún, 21.09.1984, *E. Martínez 7682* (MEXU!); Reserva Zona Urbana, a 1.74 km al SE de Frontera Corozal, 29.06.2004, *G. Aguilar 10854, F. Cruz & M. Mendéz* (MEXU!); Parque Natural Yaxchilán, porción N de la Omega, 23.06.1998, *A. Rincón 988* (MEXU!); ruinas de Loma Bonita, 24.05.1999, *S. Sinaca 2604* (MEXU!); 2 km al N de Nahá, camino a Palenque, 23.09.1988, *E. Martínez 23980 & W. Stevens* (MEXU!); Chajul, en el borde de la selva, área de la sabana, 30.03.2014, *G. Salazar 8636, L. Cabrera, A. Barceinas & S. Akira* (MEXU!); Chajul, vereda de la sabana al arroyo Miranda, 31.03.2014, *G. Salazar 8675, L. Cabrera, A. Barceinas & S. Akira* (MEXU!); 1.5 km al SE de Frontera Corozal, 14.05.2004, *G. Aguilar 10038, M. Méndez & F. Cruz* (MEXU!); 6.5 km al S de Nuevo Guerrero, por el camino a Santo Domingo, 06.05.2002, *D. Álvarez 993, J. Calónico, G. Aguilar, S. Aguilar, F. Aguilar & E. Martínez* (MEXU!); 4.1 km al E de Nuevo Guerrero, 05.05.2002, *J. Calónico 23173, E. Martínez, G. Aguilar & D. Álvarez* (MEXU!); 8.1 km al SE de Lacanjá Chansayab, 19.04.2003, *G. Aguilar 6435 & C. Chancayun* (MEXU!); 16 km al W de Frontera Echeverría, camino a Crucero Corozal, 19.06.1984, *E. Martínez 6607* (MEXU!); Arroyo Nuevo Tila, camino a Lacanjá, 6.4 km al S de Nuevo Guerrero, 27.05.2002, *G. Aguilar 1111, D. Álvarez, J.P. Abascal & E. Martínez* (MEXU!, MO!); Benito Juárez Miramar, en la orilla E de la colonia, 26.08.1993, *A. Reyes-García 2337 & M. Sousa* (MEXU!); 200 m de Nuevo Jerusalén, camino a Nuevo Francisco León, 04.05.2002, *G. Aguilar 694, J. Calónico, D. Álvarez, S. Aguilar & E. Martínez* (MEXU!). Ocozocoautla, barrancos cerca de Piedra Parada, en las cercanías de Ocozocoautla, 11.04.1949, *H. Moore 2549* (AMES!); ranchito Peña de la Flor, camino para la reina, Reserva del Ocote, 03.05.1983, *J. Calzada 9821* (XAL!). Tapachula, near Las Chicharras, 12.02.1896, *E. Nelson 3810* (AMES!). Tonalá, Paredón, *E. Matuda 16330* (MEXU!, SEL!). Villa Comaltitlán, cerca de Colonia Morelos, 06.02.1999, *A. Damon s/n* (ECO-TA-H!). Oaxaca: Santa María Chimalapa, afloramientos de roca en la cresta S del cañón del río del Corte, 4 km al NE de S.M.C., cerca de la vereda al Paso de la Cueva, 08.07.1985, *H. Hernández 1303* (MEXU!). Quintana Roo: Bacalar, 0.5 km al N de Nuevo Jerusalén, 11 km al Oeste de Graciano Sánchez (La Pantera), 03.12.1997, *G. Carnevali 4785, F. May, M. Gómez & J.C. Chab* (AMO!, CICY!). Othón P. Blanco, al NW de Estero Franco, sobre la carretera Ucum-La Unión, cerca de la frontera con Belice, 09.05.1981, *E. Ucan 955 & J. Flores* (CICY!, XAL!); Ejido Caobas, 13.5 km al SW de San José de la Montaña, en los límites con Campeche, 16.05.1984, *E. Ucan 3369, J. Calzada & C. Chan* (CICY!); 9 km al S de San José de la Montaña, en el camino a Tomás Garrida, al W de Chetumal, al S de la autopista 186, *G. Davidse 20260, M. Sousa, A. Chater & E. Cabrera* (MO!). Tabasco: Balancán, carretera N. 25, 3 m sobre el E.W.O. a la carretera N. 20, 07.12.1975, *P. Valdivia 2081* (XAL!); carretera N. 20, 1 km de la E.W.O., hacia el poblado Cuauhtémoc, 08.12.1975, *P. Valdivia 2092* (XAL!); Rancho Los Hermanos González, Río San Pedro, 29.03.2015, *C. Burelo 499, M. González, A. Pérez & C. Córdova* (UJAT!); Ejido Pan Duro, cerca del balneario El Tortuguero, 13.06.2015, *C. Burelo 536, M. González, A. Pérez & C. Córdova* (UJAT!); carretera San Miguelito-San Pedro, 28.06.2014, *C. Burelo 236, M. González & S. Rodríguez* (UJAT!). Macuspana, Centro Recreativo Agua Blanca, 17.06.1999, *J. Cruz 21 & G. Ortíz* (UJAT!); Centro Recreativo Agua Blanca, 7 km de la carretera Villahermosa-Escárcega, junto al río, 04.05.1980, *M.R. Niño 2974 & C. Cowan* (CST!, IEB!, MEXU!); Agua Blanca, 30.03.2014, *D. López 2 & S. Zúñiga* (UJAT!). Tacotalpa, 0.4 km al E de la estación Tacotalpa, potrero a la orilla del camino de terracería, rumbo a Tapijulapa, 27.05.1982, *C. Cowan 3424 & S. Zamudio* (CST!); Tapijulapa, Estación Biológica La Florida, 07.05.2012, *S. Hernández 11 & N. Jiménez* (UJAT!). Tenosique, Boca del Cerro, 23.03.2002, *N. Jiménez 519* (UJAT!); ejido El Faisán 2da Sección, en las ruinas de Chinikil-há, 15.05.1990, *S. Sol 545* (UJAT!). Veracruz: Amatlán de los Reyes, a orillas del río Negro, en el ejido Guadalupe, 09.01.1991, *C. Huerta 69* (AMO!). Apazapan, al SE de Chahuapan, 25.04.1971, *R. Hernández 1200* (F!). Catemaco, Parque de la Flora y Fauna Silvestre Tropical, cerro Pipiapan, 29.06.1991, *G. Carmona 20* (AMO!); Bastonal, 18 km al NE de Tebanca, *R. Cedillo 3923* (MEXU!). Emiliano Zapata, Barranca de Dos Ríos, 23.01.1982, *F. Herrador 3* (XAL!). Fortín, en la Barranca del Metlác, 16.07.1983, *S. Zamudio 963 B-C y A. Guadarrama* (UJAT!). Jalcomulco, Barranca de Atipa, ejido Santa María Tatetla, 03.05.1983, *L. Robles 124* (MICH!, XAL!). Las Choapas, Las Cruces, 14.07.1970, *L. Nevling 1508 & A. Gómez-Pompa* (MEXU!). Soteapan, El Bastonal, Cañada La Platanera, 15 km al SE de Tebanca, 18.09.1985, *R. Cedillo 3414* (AMO!); Arroyo Claro, 12.05.1981, *J. Palma 626* (XAL!). Tezonapa, Monte Almalonga, NE of Tezonapa, 12.11.1934, *O. Nagel sub Oestlund 4113* (AMES!). Totutla, Zacuapan, 01.02.1913, *C. Purpus 6387* (UC!); El Mirador, 18.05.1985, *A. Espíritu 42 & P. Martínez* (XAL!).

Additional records: MEXICO. Campeche: Calakmul, Zoh Laguna, 28.04.2012, *M. Méndez s/n* (https://www.inaturalist.org/observations/474084. Accessed 29 May 2025); en las ruinas de Calakmul, 05.08.2014, *J. Moreno s/n* (https://www.inaturalist.org/observations/1208663. Accessed 29 May 2025); al NE de Calakmul, 24.10.2021, *C. Delgado s/n* (https://www.inaturalist.org/observations/99414050. Accessed 29 May 2025); sobre la carretera a la Zona Arqueológica, al NE de Calakmul, 25.10.2021, *C. Delgado s/n* (https://www.inaturalist.org/observations/99414049. Accessed 29 May 2025); 32.5 km al NW de Felipe Ángeles, 19.06.2022, *C. Delgado s/n* (https://www.inaturalist.org/observations/123833688. Accessed 29 May 2025); al SW de Chacanbacab, cerca de la frontera con Quintana Roo, 24.04.2023, *I. Arellano s/n* (https://www.inaturalist.org/observations/166312636. Accessed 29 May 2025); Chicanná, 12.05.2022, *N. Damián s/n* (https://www.inaturalist.org/observations/137600715. Accessed 29 May 2025); 4.5 km al SW de Noshayab, 28.07.2023, *Biologalatriste s/n* (https://www.inaturalist.org/observations/176025825. Accessed 29 May 2025); al SE de Villa Hermosa, 02.05.2024, *I. Arellano s/n* (https://www.inaturalist.org/observations/214067842. Accessed 29 May 2025); al SE de El Sacrificio, 01.06.2025, *M. Riestra s/n* (https://www.inaturalist.org/observations/286356568. Accessed 29 May 2025). Carmen, Ciudad del Carmen, jardín botánico de la UNACAR, 01.01.2022, *M. González s/n* (Photograph). Chiapas: Maravilla Tenejapa, sobre el río Lacantún, 06.06.2017, *J. Espinoza s/n* (https://www.inaturalist.org/observations/6617141. Accessed 29 May 2025). Palenque, en el río Nututún (Chacamax), 30.09.2008, *V. Chaparro s/n* (Photograph). Quintana Roo: Othón P. Blanco, ejido Hermenegildo Galeana, 08.06.2017, *L. Ibarra s/n* (Photograph); al S de Tres Garantías, cerca del Río Hondo 16.10.2024, *E. Mendoza s/n* (https://www.inaturalist.org/observations/331277040. Accessed 29 May 2025). Veracruz: Emiliano Zapata, El Terrero, 06.04.2021, *G. Aguilar-Castillo s/n* (Photograph). Teocelo, al S de Santa Rosa, 01.03.2025, *H. Bojórquez s/n* (https://www.inaturalist.org/observations/269496578. Accessed 29 May 2025). Zentla, Colonia Manuel González, 16.02.2021, *R. Carral s/n* (https://www.inaturalist.org/observations/70221780. Accessed 29 May 2025).

6. *Specklinia microphylla* (A.Rich. & Galeotti) Pridgeon & M.W.Chase, Lindleyana, 16 (4): 258. 2001.

**Figure 24:**
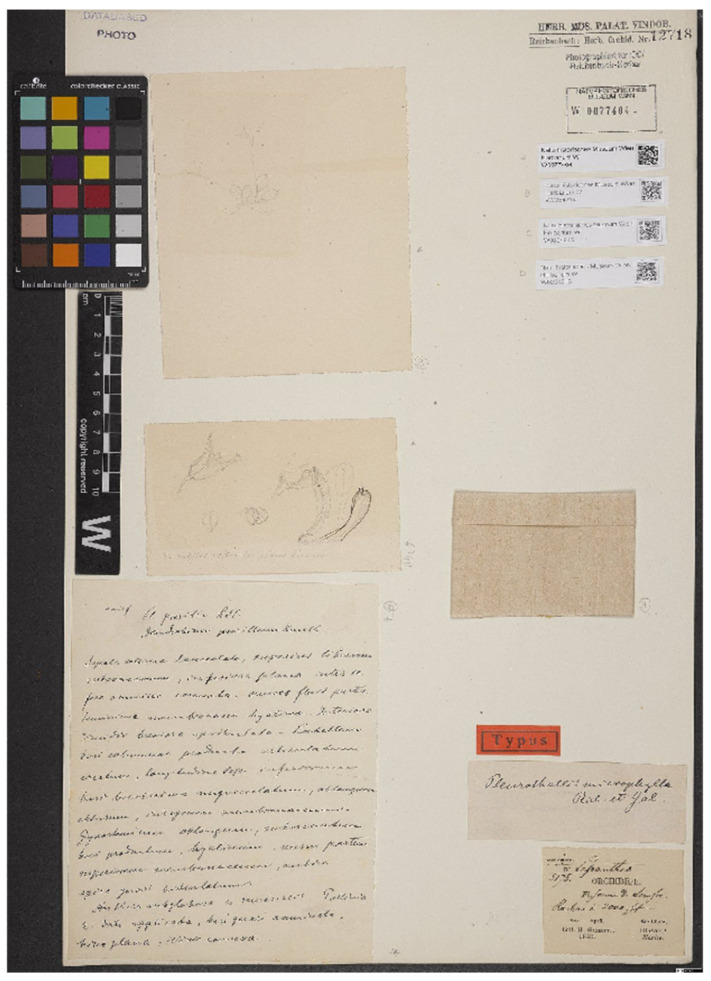
Specimen selected as lectotype of *Pleurothallis microphylla*, *H. Galeotti 5175* (W-77404), courtesy of the Herbarium of the Naturhistorisches Museum Wien.

Basionym: *Pleurothallis microphylla* A.Rich & Galeotti, Ann. Sci. Nat. Bot., sér. 3, 3: 17. 1845. TYPE: MEXICO. Oaxaca: cordillera, “rochers”, 3000 ft. *H. Galeotti 5175* (lectotype here designated: W-77404! (Figure 24); drawings based on the type: AMES-74435!, AMES-74437!, AMES-74438!).

Homotypic synonym: *Humboltia microphylla* (A.Rich. & Galeotti) Kuntze, Revis. Gen. Plant. 2: 668. 1891.

Etymology: From the Greek *μικρός (micros)*, “small or minute” and the Greek *φύλλον (phyllon)*, “leaf”; “with minute leaves”, referring to the minute size of the leaves.

**Figure 25:**
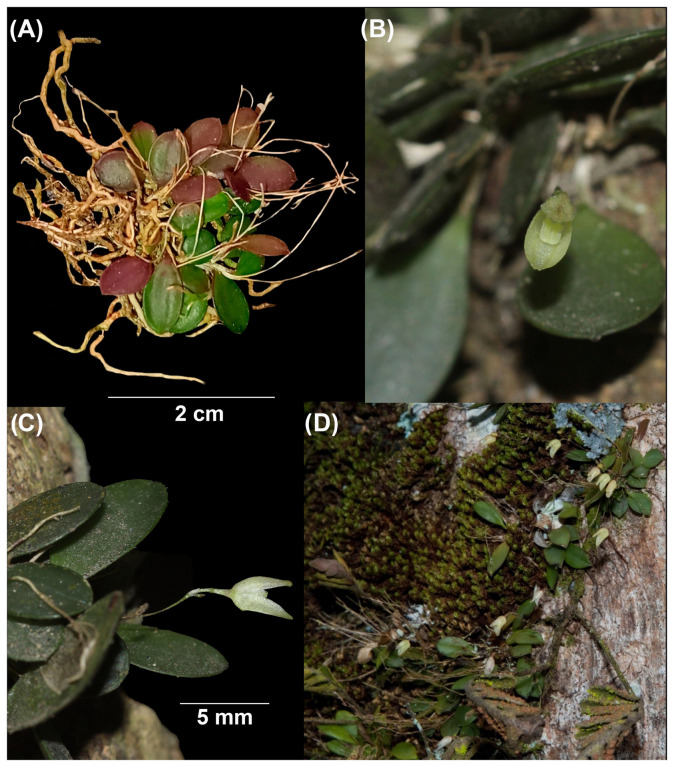
Photographs of *Specklinia microphylla*. (**A**) Specimen cultivated in Mexico City, from Chiapas. Photograph by Ethian Licona, culture by César Rivera. (**B**,**C**) Specimen cultivated in the Orchidarium of Universidad Veracruzana, Xalapa, Veracruz, from Santa María Chimalapa, Oaxaca. Photographs by Daniel Cornú. (**D**) Specimen in its habitat, Teapa, Tabasco. Photograph by Eduardo Pérez García.

**Figure 26:**
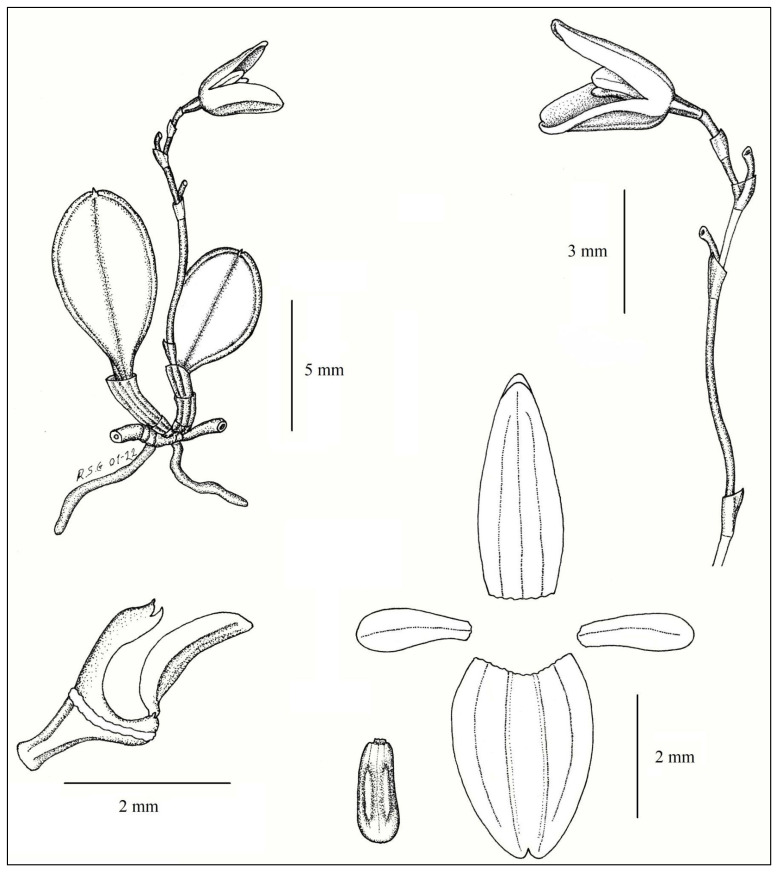
Illustration of *Specklinia microphylla*. Drawing by Rodolfo Solano, based on *P. Valdivia 199* (MEXU).

**Herb** cespitose, prostrate, up to 1.9 cm tall without the inflorescence. **Roots** 0.5–0.8 mm gross. **Rhizome** 1.0–3.5 mm long between adjacent stems. **Stem** 1.5–4.0 mm long, 0.3–0.4 mm gross, sheaths 1.1–3.5 mm long. **Leaf** 4.0–10.8 mm × 2.5–5.4 mm, elliptic–orbicular to oblong–elliptic, obtuse, succulent, biconvex, marginate, adaxially verrucose, abaxially glabrous, the apical mucro 0.2–0.3 mm long, light green, suffused with purple, with purple dots abaxially, abruptly attenuated into a petiole 1.0–4.0 mm long, 0.4–0.5 mm gross. **Inflorescence** 0.8–2.7 cm long, elongated, lax, erect, glabrous; spathaceous bract 0.2–0.5 mm long; peduncle as long or slightly longer than the leaf, 0.5–1.6 cm long, 0.1–0.2 mm gross, with two bracts 0.4–1.0 mm long, glabrous, membranaceous, hyaline; rachis 0.3–1.1 cm long, with 2–6, successive, secund flowers, 1–3 open at a time. **Floral bracts** 0.5–1.5 mm long, obliquely tubular-infundibuliform, obtuse, shortly apiculate, membranaceous, glabrous, hyaline suffused with purple. **Flowers** 2.7–4.3 mm × 1.2–2.1 mm; sepals, petals and lip white; column and foot yellowish-white; anther cream. **Sepals** concave, axially carinated, glabrous; dorsal sepal 2.5–3.7 mm × 1.3–1.6 mm, lanceolate to oblong–ovate, acute, 3-veined, the apex forming a calyptra, papillar-glandulous inside, 0.4–0.5 mm × 0.3–0.4 mm; lateral sepals 3.2–4.3 mm × 1.4–2.2 mm, connate into an ovate to elliptic, obtuse, shortly bifid synsepal, 4-veined, gibbose in the base. **Petals** 1.4–2.3 mm × 0.4–0.6 mm, obliquely oblong–obovate to narrowly oblanceolate, obtuse, falcate, 1-veined. **Lip** 1.5–1.8 mm × 0.5–0.6 mm, fleshy, oblong–obovate, rounded, 3-veined, minutely papillar-glandulous, with a pair of submarginal calli, channeled between them. **Column** 1.3–1.6 mm long, 0.4–0.6 mm width; wings narrowly triangular, acute, entire, 0.3–0.4 mm × 0.1–0.3 mm; clinandrium obtuse, entire; foot 0.8–1.2 mm long, 0.3–0.5 mm width, obtuse, with two globular, low calli at the base. **Stigmatic cavity** 0.15 mm × 0.25 mm, elliptic–oblong; rostellum laminar, oblong. **Anther** 0.2–0.4 mm long and width, ovoid. **Pollinia** 0.15–0.3 mm × 0.15–0.25 mm, obovoid. **Ovary** 0.5–1.3 mm long, 0.2–0.4 mm gross, obconical, straight, glabrous, greenish-white suffused with purple; pedicel 0.7–2.2 mm long, 0.1–0.2 mm gross, glabrous. **Capsule** 2.6–3.2 mm long, 1.0–2.4 mm gross, elliptic–obovoid, yellowish-green suffused with purple (Figure 25 and Figure 26).

Distribution: Endemic. The species is known only from the states of Oaxaca, Tabasco and Veracruz, probably also present in northwestern Chiapas. It occurs in the Floristic Provinces of Costa del Golfo de México, Serranías Meridionales and Valle de Tehuacán.

**Figure 27:**
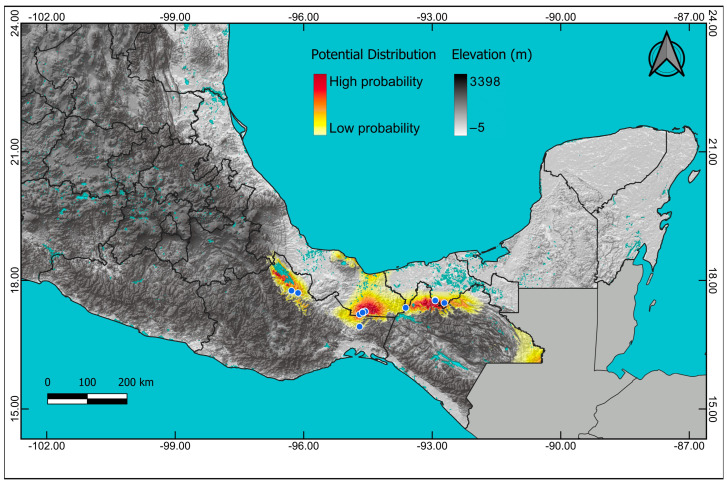
Potential distribution of *Specklinia microphylla* in Mexico and the location of currently known occurrence records (blue dots). Map by Ethian Licona.

The accepted potential distribution model for *S. microphylla* yields an AUC = 0.933, where the most influential environmental variables were precipitation of the warmest quarter (37.3%), annual precipitation (37.2%) and isothermality (10.0%). The potential distribution area was estimated at 31,847.41 km^2^, representing approximately 1.62% of Mexico’s total territory (Figure 27).

Habitat: Epiphyte on *Brosimum alicastrum*, *B. costaricanum* Liebm., *Cornutia pyramidata*, *Guazuma ulmifolia* Lam., *Lonchocarpus guatemalensis* Benth., *Plumeria rubra*, *Pouteria sapota* (Jacq.) H.E.Moore & Stearn, *Pseudobombax ellipticum* (Kunth) Dugand and *Tabebuia rosea* (Bertol.) DC. It has also been recorded on *Citrus × sinensis* and occasionally as a lithophyte. The species grows in the evergreen tropical forest, *Quercus* forest, riparian vegetation, secondary vegetation derived from these forest types, and orange orchards, at elevations between 60 and 920 m.

Phenology: Flowering occurs from July to April. Mature fruits have been observed from October to June.

Conservation status: In the assessment of its conservation status following the MER criteria [78], a score of 1.53 was obtained, which suggests it should be considered as a species Under Special Protection (Pr). In the evaluation under IUCN criterion B, it achieved an EOO of 19,015.418 km^2^ and an AOO of 60.00 km^2^, suggesting that the species should be classified as Vulnerable (VU).

Additional commentaries: *Specklinia microphylla* can be recognized by its prostrate habit; elliptic–orbicular, obtuse, marginate leaves; inflorescence with a peduncle as long as the leaf; successive, white flowers, 1–3 open at a time; lanceolate, shortly calyptrate dorsal sepal; ovate, obtuse, shortly bifid synsepal; obliquely oblanceolate, obtuse, falcate petals; oblong–obovate, rounded lip. The morphological differences among *S. microphylla* and the remaining species of the *S. digitale* complex are summarized in Table 3.

This species has long been confused [18,39,41] with members of the *S. grobyi-picta* complex, which are characterized by its rhizomatous habit with orbicular, prostrate, leaves and acuminate, bright yellow flowers with red lines on the dorsal sepal. This former concept of *S. microphylla* was previously reported for Mexico and Central America; however, the Central American populations can be segregated into two clearly distinct entities. The first is distributed along the Pacific coast from Costa Rica and Panama, it was recently recognized as a separate species, as *Specklinia panamensis* (Schltr.) Bogarín & Pupulin [88]. The second is distributed along the Caribbean coast from Nicaragua through Costa Rica and Panama, extending inland towards the limits of Honduras and El Salvador. However, this entity does not correspond to *S. microphylla* either and likewise represent an undescribed species requiring taxonomic recognition.

Examined specimens: MEXICO. Oaxaca: Ayotzintepec, 3.7 km al NE de Ayotzintepec, en el cerro Cueva de los Duendes, 06.04.2011, *G. Juárez 3759* (MEXU!). San Juan Bautista Valle Nacional, cerro de La Loma, 3.3 km al E de Santa Fé y La Mar, 05.09.2009, *K. Velasco 4272, L. Vásquez & E. Enriquez* (MEXU!). Tabasco: Tacotalpa, Cerro La Pila, Carretera Mexiquito km 16, 30.10.2002, *F. Hernández 618* (CHIP!). Teapa, Puyacatengo, Jardín Agrícola Tropical, URUSSE-UACH, 20.02.1996, *M. Mendieta 10* (UJAT!); 2 km al E de Teapa, camino a Puyacatengo, 12.11.1984, *E. Martínez 8555, O. Tellez & M. Sousa* (AMO!, MEXU!); Sierra El Madrigal, 1200 m al NE del edificio principal del Centro Regional Tropical Puyacatengo, 16.10.1991, *A. Hanan 573* (UJAT!); San José Puyacatengo, 13.11.1988, *V. Ramón 153* (MEXU!). Veracruz: Uxpanapa, 7 km del campamento Hermanos Cedillo, al SW, a mano derecha, 07.03.1974, *P. Valdivia 35* (XAL!); al E del campamento Hermanos Cedillo, 1 km hacía La Laguna, 10.04.1974, *P. Valdivia 199* (MEXU!, XAL!); hacía Álvaro Obregón, 800 m del campamento Hermanos Cedillo, rumbo al W, 24.04.1974, *P. Valdivia 473* (MEXU!, XAL!); en el lindero Hermanos Cedillo hacia La Laguna (Congregación Piedra de Azúcar), a mano derecha 200 m, 24.05.1974, *P. Valdivia 696* (XAL!); 800 m del campamento Hermanos Cedillo, hacía La Escuadra, 26.07.1974, *P. Valdivia 1121* (MEXU!, XAL!).

Additional records: MEXICO. Chiapas: Without known locality, cultivated in Mexico City, 20.06.2021, *C. Rivera s/n* (Photograph). Oaxaca: Santa María Chimalapa, camino al río del Corte, empezando a bajar al cañón, aprox. 2 km de S. M. C., 01.01.2014, *D. Moreno s/n* (Photograph). Tabasco: Huimanguillo, cerca de Villa de Guadalupe, en la sierra, 01.01.2022, *M. González s/n* (Photograph). Teapa, Puyacatengo, undated, *E.A. Pérez-García s/n* (Photograph).

7. *Specklinia orbiculata* E.Licona & Solano *sp. nov.*

**Figure 28:**
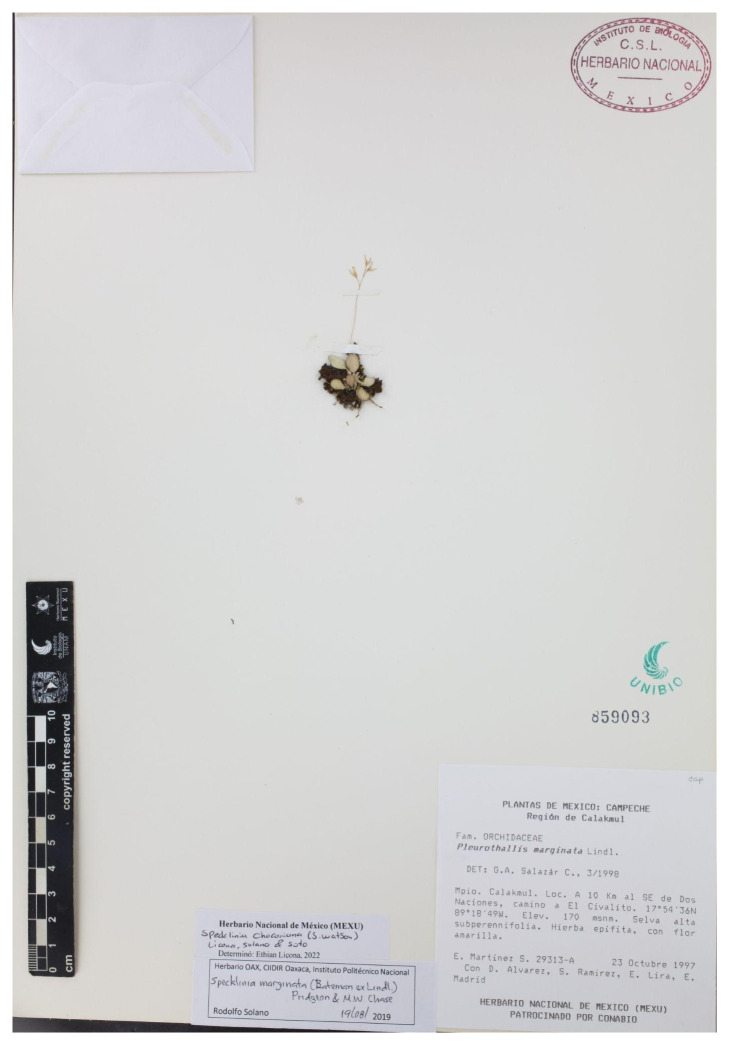
Holotype of *Specklinia orbiculata*, *E. Martínez et al. 29313-A* (MEXU-859093), courtesy of the National Herbarium of the Universidad Nacional Autonoma de Mexico.

HOLOTYPE: MEXICO. Campeche: Calakmul, 10 km al SE de Dos Naciones, camino a El Cibalito, 170 m, 23 Octubre 1997, *E. Martínez 29313-A, D. Álvarez, S. Ramírez, E. Lira & E. Madrid* (MEXU-859093! (Figure 28)).

Etymology: From the Latin *orbiculatus*, “orbicular shape”, referring to the shape of the leaves.

*Diagnosis: Similar to Specklinia grobyi from which it differs by its smaller, prostrate habit (up to 1.8 cm tall without the inflorescence vs. up to 4.0 cm tall, ascending), orbicular–elliptic, 5.0–9.0 mm long, shortly petiolate leaves (vs. obovate, 7.4–22.0 mm long, long petiolate), raceme up to 8.2 cm long with flowers arranged sub-parallel to the rachis, (vs. raceme up to 12.0 cm long with flowers arranged perpendicular to the rachis), dorsal sepal elliptic–lanceolate (vs. ovate), synsepal elliptic–lanceolate, acute (vs. oblong–ovate, obtuse), petals obovate, obtuse–apiculate (vs. lanceolate, acuminate).*

**Figure 29:**
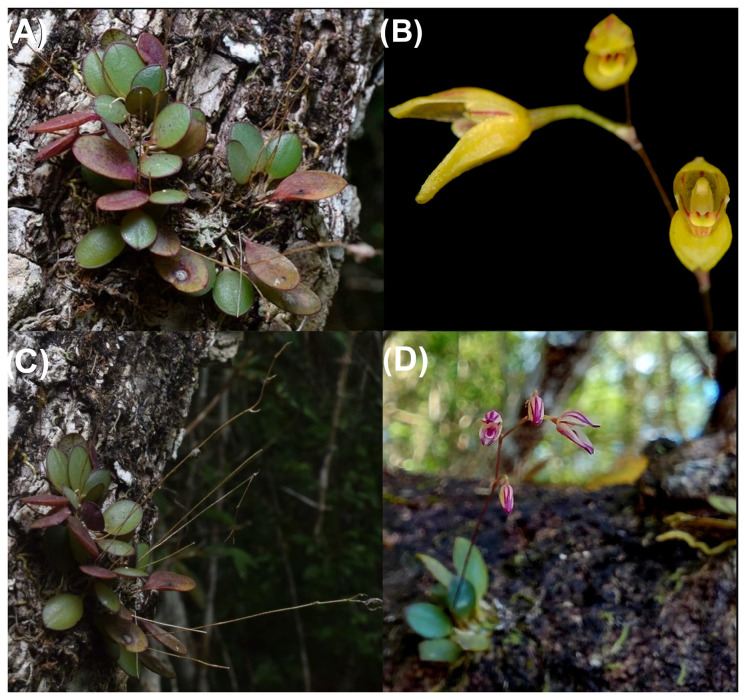
Photographs of *Specklinia orbiculata*. (**A**,**C**) Specimen in its habitat, Calakmul, Campeche. Photographs by René Vela. (**B**) Specimen cultivated in Cobán, Guatemala. Photograph by José Monzón and Edgar Mó. (**D**) Specimen in its habitat, Petén, Guatemala. Photograph by Wilfredo Chub.

**Figure 30:**
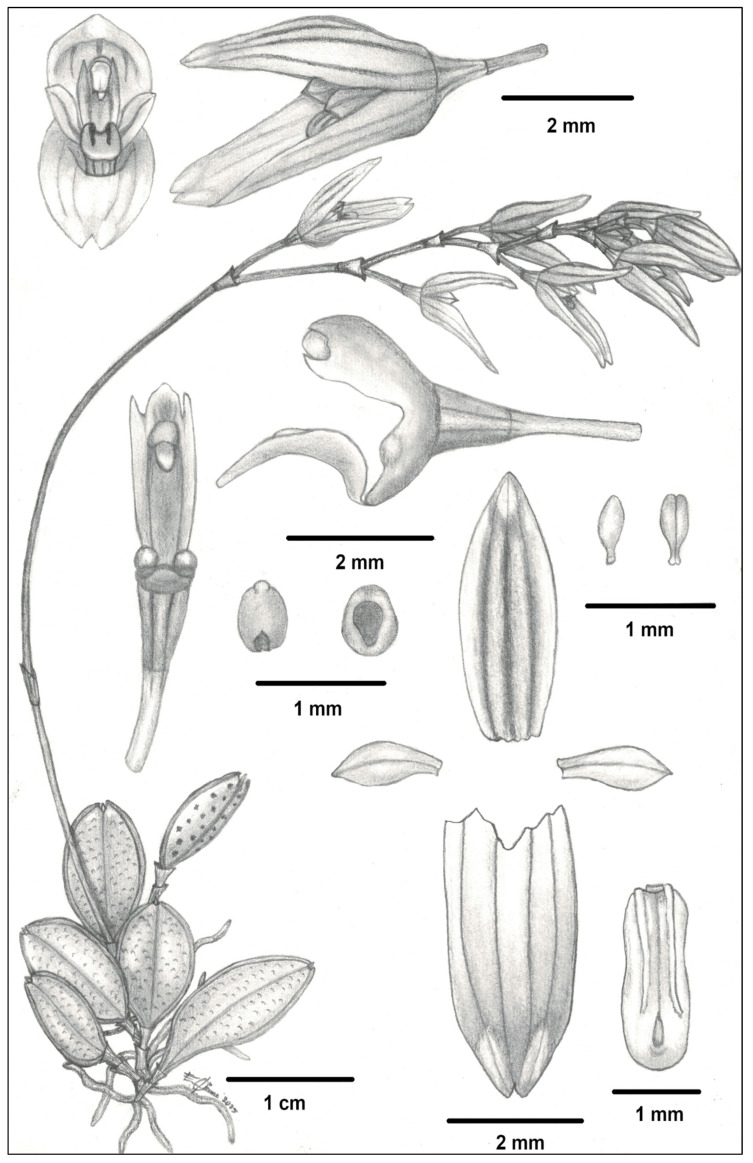
Illustration of *Specklinia orbiculata*. Drawing by Ethian Licona, based on without data specimen *66467* (AMO, in spirit).

**Herb** shortly rhizomatous, prostrate, up to 1.8 cm tall without the inflorescence. **Roots** 0.5–0.8 mm gross. **Rhizome** 0.5–2.0 mm long between adjacent stems. **Stem** 2.0–4.5 mm long, 0.5–0.7 mm gross, sheaths 1.2–3.5 mm long. **Leaf** 5.0–9.0 mm × 3.0–6.5 mm, orbicular–elliptic to elliptic–obovate, rounded to obtuse, succulent, biconvex, marginate, adaxially verrucose, abaxially glabrous, the apical mucro 0.2–0.3 mm long, light green, sometimes suffused with purple, with purple dots abaxially, abruptly attenuated into a petiole 1.0–1.7 mm long, 0.4–0.5 mm gross. **Inflorescence** 3.0–8.2 cm long, erect, elongated, lax, glabrous; spathaceous bract 0.8–1.0 mm long; peduncle longer than the leaf, 2.2–5.7 cm long, 0.2–0.4 mm gross, with 2–3 bracts 1.3–1.6 mm long, glabrous, hyaline, membranaceous; rachis 0.8–2.8 cm long, with 3–8, simultaneous, distichous, flowers, sub-parallel to the rachis axis. **Floral bracts** 0.5–1.8 mm long, obliquely tubular-infundibuliform, obtuse, shortly apiculate, membranaceous, glabrous, hyaline suffused with purple. **Flowers** 3.5–5.0 mm × 1.5–2.3 mm; sepals bright to pale yellow with red lines on both sepals; petals bright yellow with a red line; lip bright yellow to red with red calli and the glenion-like depression yellow; column and foot bright yellow; anther yellowish-white. **Sepals** concave, axially carinate, glabrous; dorsal sepal 2.8–4.7 mm × 1.0–1.8 mm, elliptic–lanceolate to elliptic–ovate, acute, 3-veined, with an ovate–lanceolate apical thickening, 1.0–1.2 mm × 0.3–0.4 mm; lateral sepals 3.5–5.0 mm × 1.6–2.5 mm, connate into an elliptic–lanceolate to oblong–lanceolate, acute, shortly bifid synsepal, 4-veined, gibbose at the base, with two obliquely triangular to oblong–lanceolate apical thickenings, 1.0–1.5 mm × 0.4–0.6 mm. **Petals** 1.4–2.1 mm × 0.4–0.6 mm, obliquely oblong–obovate, obtuse, minutely apiculate, slightly falcate, attenuated towards the base, 1-veined. **Lip** 1.6–2.4 mm × 0.5–0.8 mm, fleshy, oblong–ligulate, obtuse, 3-veined, minutely papillar-glandulous, with a pair of submarginal calli, channeled between them, with a glenion-like depression near the apex. **Column** 1.5–2.4 mm long, 0.4–0.6 mm width, wings obliquely triangular, acute, entire, 0.4–0.6 mm × 0.3–0.4 mm; clinandrium obtuse, entire; foot 0.7–1.5 mm long, 0.3–0.5 mm width, obtuse, with two globular, high calli at the base. **Stigmatic cavity** 0.2 mm × 0.3 mm, elliptic–oblong; rostellum laminar, oblong. **Anther** 0.4–0.5 mm long, 0.3–0.4 mm width, elliptic–ovoid. **Pollinia** 0.25–0.3 mm × 0.2–0.25 mm, obovoid. **Ovary** 0.8–1.8 mm long, 0.3–0.6 mm gross, obconical, straight, glabrous, yellowish-green suffused with purple; pedicel 1.2–4.5 mm long, 0.2–0.3 mm gross, glabrous. **Capsule** 5.0–7.0 mm long, 2.5–3.6 mm gross, elliptic–obovoid, yellowish-green suffused with purple (Figure 29 and Figure 30).

Distribution: Mexico, Guatemala and Belize. In Mexico, the species is distributed in the states of Campeche, Chiapas and Quintana Roo. Occurring mainly in the Floristic Province of Península de Yucatán, with few localities in Costa del Golfo de México and Costa Pacífica.

**Figure 31:**
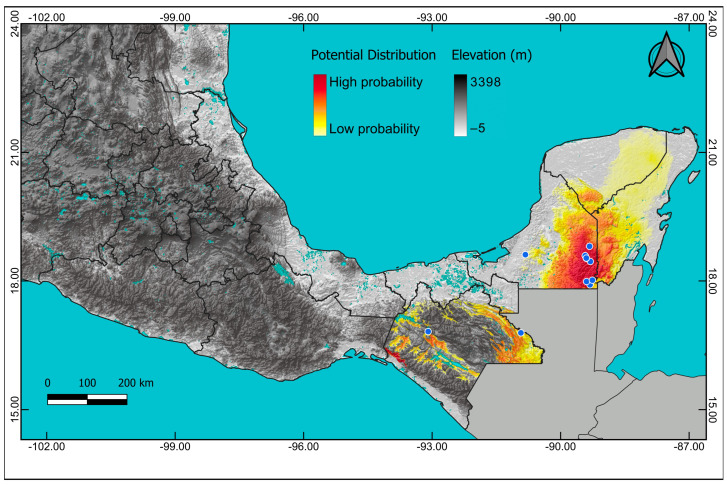
Potential distribution of *Specklinia orbiculata* in Mexico and the location of currently known occurrence records (blue dots). Map by Ethian Licona.

The accepted potential distribution model for *S. orbiculata* yields an AUC = 0.902, where the most influential environmental variables were the Digital Elevation Model of Mexico (77.4%), isothermality (21.1%) and mean diurnal range (1.1%). The potential distribution area was estimated at 84,555.77 km^2^, representing approximately 4.3% of Mexico’s total territory (Figure 31).

Habitat: Epiphyte on *Brosimum alicastrum*, *Haematoxylum campechianum*, *Manilkara zapota*, *Metopium brownei*, *Pouteria sapota*, *Quercus* sp., *Terminalia amazonia* and *T. buceras* (L.) C.Wright. The species grows in the semi-deciduous tropical forest and thorn forest, at elevations between 60 and 300 m. In the Cañón del Sumidero National Park, Chiapas, it has been recorded in the *Quercus* forest, at 1260 m.

Phenology: Flowering occurs from April to October. Mature fruits have been observed from June to December.

Conservation status: In the assessment of its conservation status following the MER criteria [78], a score of 1.47 was obtained, therefore it is not necessary to include it in any risk category. However, this score is close to the threshold for classify as Under Special Protection (Pr). In the evaluation under IUCN criterion B, it achieved an EOO of 47,955.140 km^2^ and an AOO of 40.00 km^2^, suggesting that this species should be classified as Vulnerable (VU). Most known populations are concentrated in the southern Yucatán Peninsula, a region undergoing a rapid habitat transformation, particularly due to the construction of the Tren Maya and the associated expansion of tourism development. These factors represent potential threats to the species.

Additional commentaries: *Specklinia orbiculata* is recognized by its prostrate habit; orbicular–elliptic, obtuse, marginate leaves, with a very short petiole; raceme erect, longer than the leaf, bearing distichous, bright to pale yellow flowers with red lines, arranged sub-parallel to the rachis axis; sepals acute, dorsal sepal elliptic–ovate, synsepal elliptic–lanceolate; petals obovate, falcate, obtuse, minutely apiculate; oblong–ligulate, obtuse lip, yellow with red calli.

This species has also been confused with *Specklinia grobyi*. Table 2 presents a comparative summary that allows the recognition of *S. orbiculata* from the other Mexican species of the *S. grobyi-picta* complex.

Examined specimens: MEXICO. Campeche: Without known locality, without date, *Anon., specimen 66467* (AMO! In spirit). Calakmul, 6 km al NW de Dos Naciones, camino a Ranchería El Sacrificio, 27.10.1997, *E. Martínez 29480, D. Álvarez & S. Ramírez* (MEXU!); 1 km al E de 16 de septiembre, camino a Santa Rosa, 26.04.1998, *E. Martínez 30672, D. Álvarez & C. Galindo* (MEXU!); 3 km al E de Nueva Vida, camino a La Mancolona, 07.12.1998, *E. Martínez 31619, D. Álvarez & S. Ramírez* (MEXU!). Escárcega, Campo experimental Ing. Eduardo Sangrí Serrano, 23.07.1997, *M. Sarmiento 37* (AMO!); Campo experimental Ing. Eduardo Sangrí Serrano, 23.07.1997, *M. Sarmiento 38* (AMO!). Chiapas: Ocosingo, Restauración Ecológica El Cartón, 5.6 km al SW de Frontera Corozal, 25.05.2004, *G. Aguilar 10234, M. Méndez & F. Cruz* (MEXU!). Tuxtla Gutiérrez, El Sumidero, 04.07.1954, *R. Dressler 1387* (MEXU!).

Additional records: GUATEMALA. Petén: San Andrés, Parque Nacional Laguna del Tigre, Estación Biológica Las Guacamayas, 29.10.2025, W. Chub s/n (https://www.inaturalist.org/observations/324425605. Accessed 30 May 2025). MEXICO. Campeche: Calakmul, Ejido 20 de Noviembre, 01.09.2021, *R. Vela s/n* (https://www.inaturalist.org/observations/105759467. Accessed 30 May 2025); cerca a CFE SE Xpujil, 10.05.2014, *M. Barba s/n* (https://www.inaturalist.org/observations/194066408. Accessed 30 May 2025); Universidad Tecnológica de Calakmul, al N de Xpujil, 02.10.2019, *K. Uc s/n* (https://www.inaturalist.org/observations/33952290. Accessed 30 May 2025); al W de El Refugio, 19.08.2018, *S.J. Calva s/n* (https://www.inaturalist.org/observations/51901398. Accessed 30 May 2025).

8. *Specklinia perez-garciae* E.Licona & Solano *sp. nov.*

**Figure 32:**
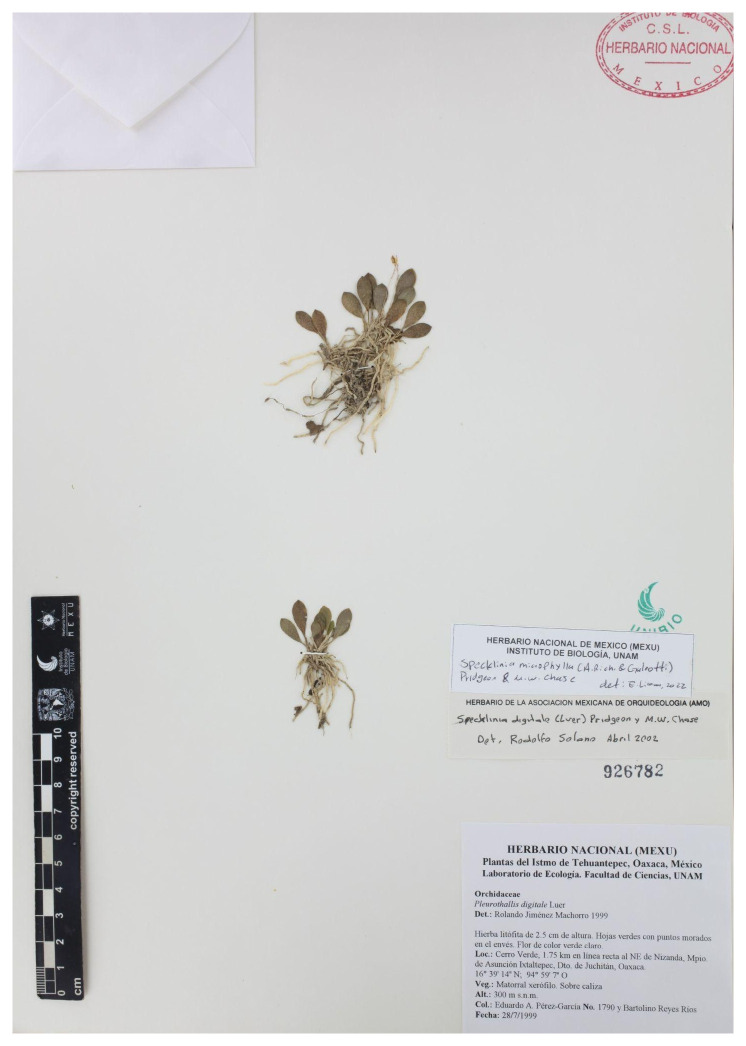
Holotype of *Specklinia perez-garciae, E.A. Pérez-García 1790 & B. Reyes* (MEXU-926782), courtesy of the National Herbarium of the Universidad Nacional Autonoma de Mexico.

HOLOTYPE: MEXICO. Oaxaca: Asunción Ixtaltepec, Cerro Verde, 1.75 km en LR al NE de Nizanda, 304 m, 28 July 1999, *E.A. Pérez-García 1790 & B. Reyes* (MEXU-926782! (Figure 32)).

Etymology: Named after Eduardo Alberto Pérez-García, researcher committed to the conservation of Mexican orchids.

*Diagnosis: Similar to Specklinia digitale, from which it differs by its elliptic–oblanceolate leaves (vs. obovate); inflorescence up to 4.5 cm long with a peduncle shorter or as long as the leaf (vs. up to 10.8 cm long with a peduncle longer than the leaf); dorsal sepal oblong–ovate with a calyptra 1.0–1.4 mm long (vs. oblong–elliptic with a calyptra 0.5–0.7 mm long); synsepal oblong–elliptic (vs. ovate) and petals oblanceolate (vs. obovate).*

**Figure 33:**
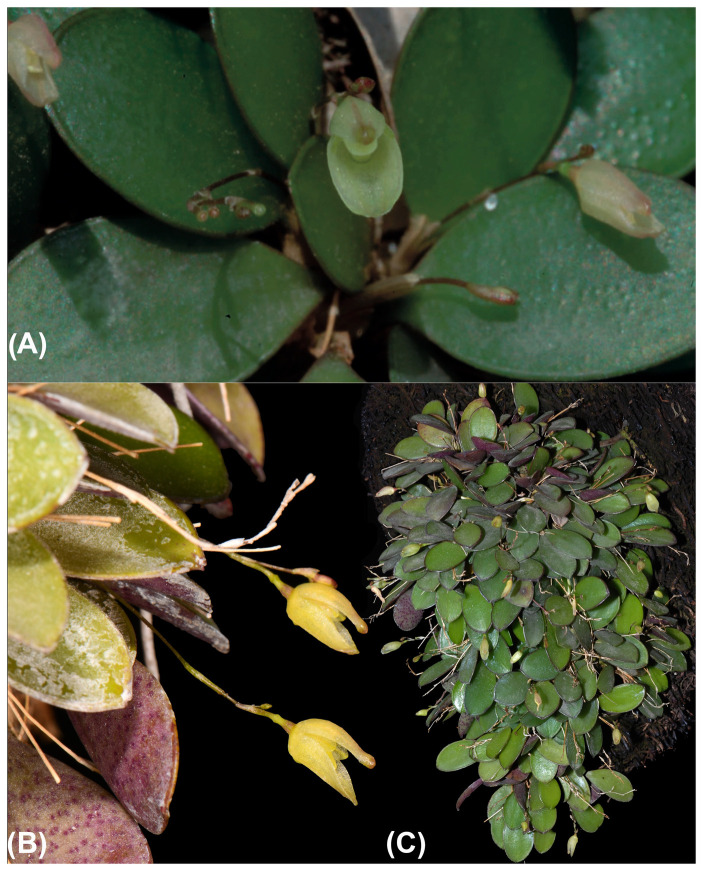
Photographs of *Specklinia perez-garciae*. (**A**) Specimen in its habitat, Asunción Ixtaltepec, Oaxaca. Photograph by Eduardo Pérez-García. (**B**,**C**) Specimen cultivated in Orchidarium M. A. S., in Mexico City. Photographs by Ethian Licona (**B**) and Gerardo Salazar (**C**), from the type.

**Figure 34:**
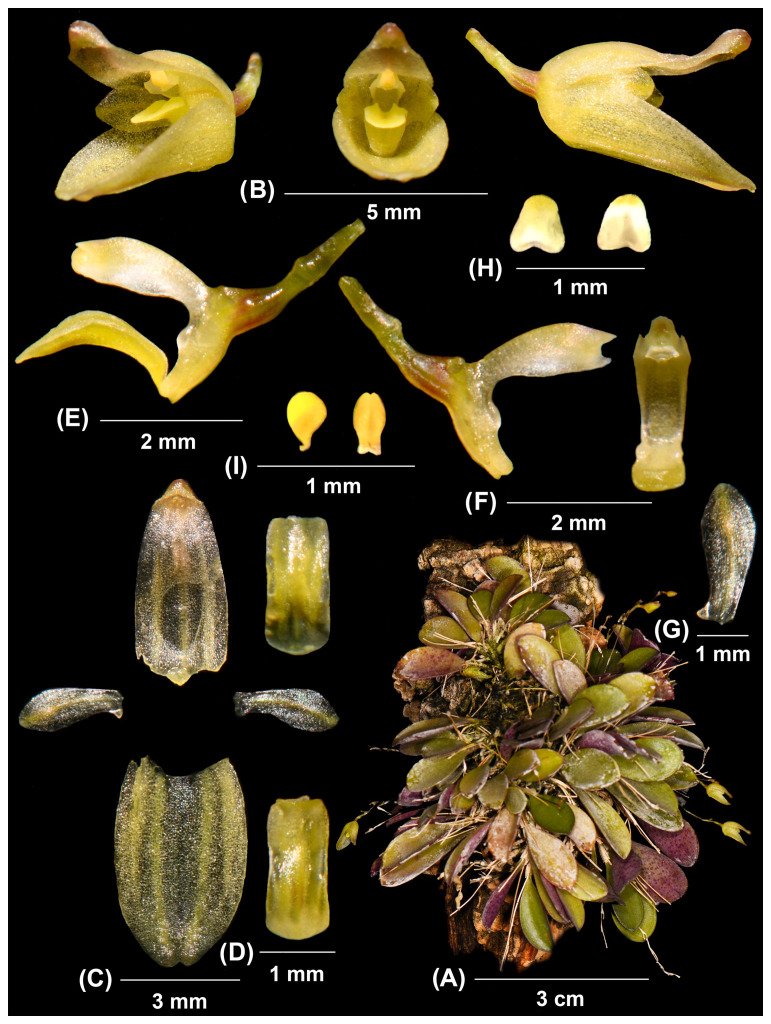
LCDP of *Specklinia perez-garciae*. (**A**) Habit. (**B**) Flower, ¾, frontal and lateral views. (**C**) Dissected flower. (**D**) Lip, dorsal and ventral views. (**E**) Column, lip and pedicelate ovary in lateral view. (**F**) Column, lateral and ventral views. (**G**) Petal. (**H**) Anther, dorsal and ventral views. (**I**) Pollinia, lateral and frontal views. Plate by Ethian Licona, based on the type.

**Herb** cespitose, prostrate to ascending, up to 2.5 cm tall without the inflorescence. **Roots** 0.5–0.9 mm gross. **Rhizome** 1.0–2.0 mm long between adjacent stems. **Stem** 2.5–5.5 mm long, 0.4–0.6 mm gross, sheaths 2.0–4.0 mm long. **Leaf** 6.7–20.0 mm × 3.0–10.0 mm, elliptic–oblanceolate to oblong–oblanceolate, obtuse, succulent, biconvex, marginate, adaxially verrucose, abaxially glabrous, the apical mucro 0.2–0.4 mm long, yellowish-green suffused with purple, with purple dots abaxially, abruptly attenuated into a petiole 1.0–3.0 mm long, 0.4–0.6 mm gross. **Inflorescence** 1.2–4.5 cm long, elongated, lax, flexuous, glabrous; spathaceous bract 0.5–0.9 mm long; peduncle shorter or as long as the leaf, 0.4–2.5 cm long, 0.1–0.2 mm gross, with 2 bracts 1.0–1.3 mm long, glabrous, membranaceous, hyaline; rachis 0.5–2.0 cm long, with 3–10, successive, secund flowers, 1–3 open at a time. **Floral bracts** 1.0–1.2 mm long, obliquely tubular-infundibuliform, obtuse, shortly apiculate, membranaceous, glabrous, hyaline suffused with purple. **Flowers** 3.6–5.5 mm × 2.3–2.8 mm; sepals and petals yellowish with the apex of the dorsal sepal pale pink; lip light yellow; column yellowish-white, foot light yellow; anther yellowish-white. **Sepals** concave, axially carinate, glabrous; dorsal sepal 3.3–5.1 mm × 1.4–2.2 mm, oblong–ovate to elliptic, acute, 3-veined, the apex forming a calyptra papiliar-glandulous inside, 1.0–1.4 mm × 0.6–0.8 mm; lateral sepals 3.6–5.5 mm × 2.0–3.2 mm, connate into an oblong–elliptic, obtuse, shortly bifid synsepal, 4-veined, gibbose in the base. **Petals** 2.1–2.5 mm × 0.6–1.0 mm, obliquely oblanceolate, obtuse, falcate, 1-veined. **Lip** 2.8–3.4 mm × 1.3–1.6 mm, fleshy, oblong–ligulate, obtuse–rounded, 3-veined, minutely papillar-glandulous, with a pair of submarginal calli, channeled between them. **Column** 2.2–2.4 mm long, 0.6–0.8 mm width, wings narrowly triangular, acute, entire, 0.4–0.6 mm × 0.1–0.3 mm; clinandrium obtuse, slightly undulate; foot 1.4–1.6 mm long, 0.5–0.7 mm width, obtuse, with two globular, high calli at the base. **Stigmatic cavity** 0.2 mm × 0.3 mm, elliptic–oblong; rostellum laminar, oblong. **Anther** 0.4–0.5 mm long and width, ovoid. **Pollinia** 0.3–0.4 mm × 0.2–0.25 mm, obovoid. **Ovary** 1.0–1.7 mm long, 0.3–0.7 mm gross, obconical, slightly recurved, glabrous, yellowish-green suffused with pink; pedicel 1.0–2.0 mm long, 0.2–0.3 mm gross, glabrous. **Capsule** not seen (Figure 33 and Figure 34).

**Figure 35:**
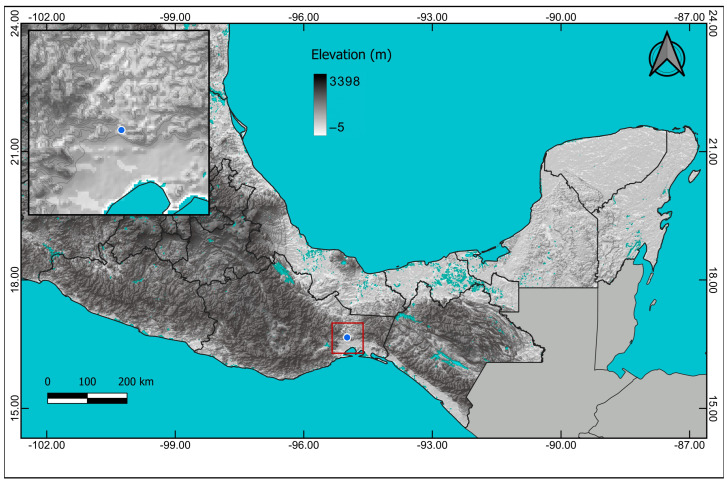
Distribution map of *Specklinia perez-garciae* in Mexico (blue dots). Map by Ethian Licona.

Distribution: Endemic. Currently this species is known only from the type locality in the state of Oaxaca. It occurs between the limits of the Floristic Provinces Costa Pacífica and Serranías Meridionales (Figure 35).

Habitat: The species has only been recorded as a lithophyte, but it may also occur as an epiphyte. It grows in a xerophytic scrub surrounded by tropical deciduous forest and thorn forest, at elevations between 300 and 400 m.

Phenology: Flowering occurs from February to August. Mature fruits have not been observed.

Conservation status: In the assessment of its conservation status following the MER criteria [78], a score of 2.48 was obtained, which suggests that it should be considered as Endangered (P). In the evaluation under IUCN criterion B, it was not possible to perform the EOO since it is known only from a single locality, while its AOO is only 2.00 km^2^; this combination of factors, together with the rarity of both the species and its habitat, suggests that it should be classified as Critically Endangered (CR). Due to its extremely restricted distribution, this species faces a high risk of extinction as a result of major landscape transformations currently occurring in the Isthmus of Tehuantepec region, in addition to the habitat damage caused by climate change.

Additional commentaries: *Specklinia perez-garciae* can be recognized by its prostrate habit; elliptic–oblanceolate, obtuse, marginate leaves; peduncle shorter or as long as the leaf; rachis with successive, secund, yellowish-white flowers; dorsal sepal oblong–ovate, with the apex deeply calyptrate; synsepal oblong–elliptic, obtuse; petals oblanceolate, obtuse, falcate; lip oblong–ligulate, widely obtuse. This species is part of the *S. digitale* complex, the morphological differences among its members are summarized in Table 3.

9. *Specklinia pisinna* (Luer) Solano & Soto-Arenas, Icon. Orchid., 5–6: xi. 2003.

**Figure 36:**
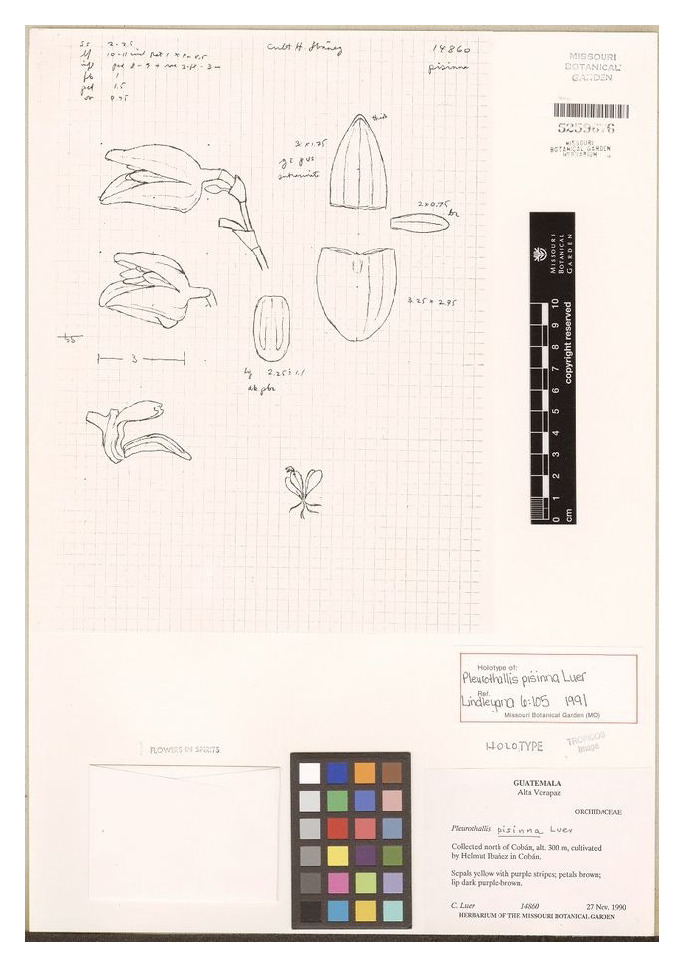
Drawing by C. Luer based on the type (holotype inside the envelope) of *Pleurothallis pisinna*, *C. Luer 14860* (MO-149977, https://www.tropicos.org/image/37055. Accessed 31 May 2025), courtesy of the Herbarium of Missouri Botanical Garden.

Basionym: *Pleurothallis pisinna* Luer, Lindleyana, 6 (2): 105. 1991. TYPE: GUATEMALA. Alta Verapaz: Cobán, N of Cobán, 300 m, collected and cultivated by H. Ibañez in Cobán, 27 November 1990, *C. Luer 14860* (holotype: MO-149977! (Figure 36)).

Homotypic synonym: *Specklinia pisinna* (Luer) Luer, Monogr. Syst. Bot. Missouri Bot. Gard., 95: 263. 2004, *nom. superfl.*

Etymology: From the Latin *pisinnus*, “very small”; referring to its small habit and flowers.

**Figure 37:**
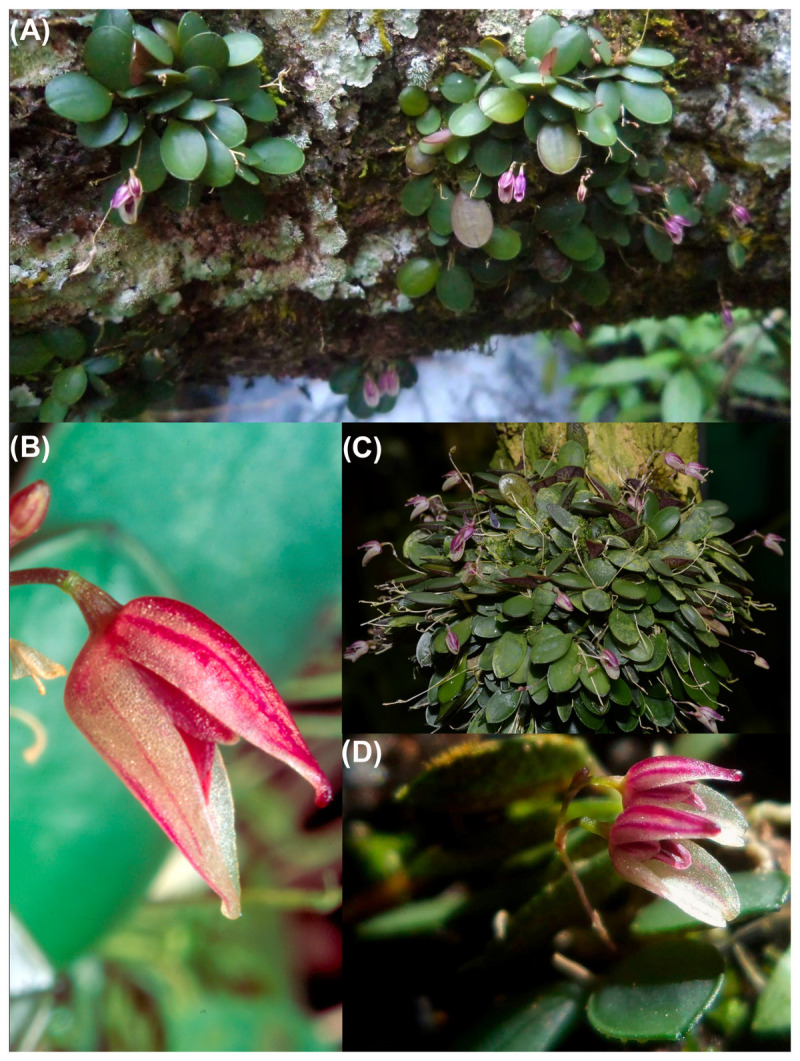
Photographs of *Specklinia pisinna*. (**A**,**D**) Specimen in its habitat, Ocosingo, Chiapas. Photographs by Matus Hernández. (**B**) Specimen cultivated in Mexico City. Photograph by Rodolfo Solano, based on *M.A. Soto-Arenas 5657* (AMO). (**C**) Specimen cultivated in Mexico City. Photograph by Gerardo Salazar, based on *G. Salazar & S. Sinaca 487* (AMO).

**Figure 38:**
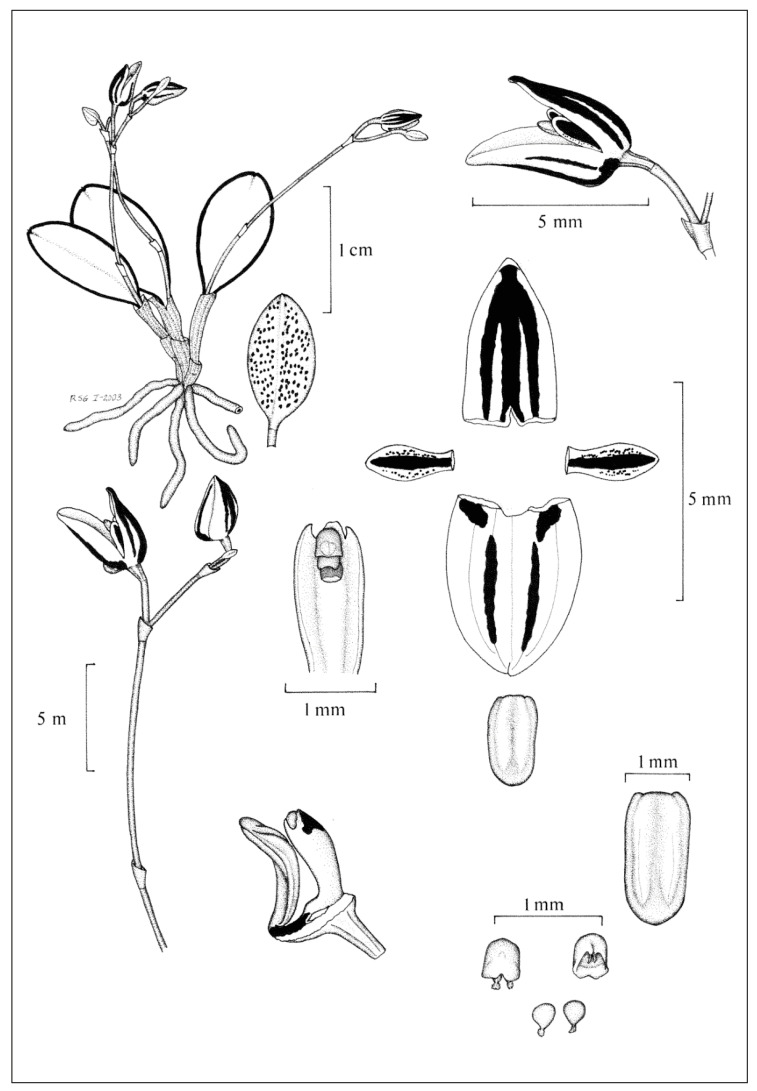
Illustration of *Specklinia pisinna*. Drawing by Rodolfo Solano, based on *M.A. Soto-Arenas 5657* (AMO).

**Herb** cespitose, prostrate, up to 2.3 cm tall without the inflorescence. **Roots** 0.3–0.6 mm gross. **Rhizome** 0.5–2.0 mm long between adjacent stems. **Stem** 2.0–6.0 mm long, 0.4–0.5 mm gross, sheaths 1.2–3.7 mm long. **Leaf** 4.0–14.0 mm × 3.0–6.5 mm, elliptic–obovate to oblong–obovate, obtuse to rounded, succulent, biconvex, marginate, adaxially verrucose, abaxially glabrous, the apical mucro 0.2–0.4 mm long, light green with a darker margin, suffused with purple, with purple dots abaxially, abruptly attenuated into a petiole 0.7–3.0 mm long, 0.3–0.5 mm gross. **Inflorescence** 0.7–3.6 cm long, erect, elongated, lax, glabrous; spathaceous bract 0.4–0.7 mm long; peduncle longer than the leaf, 0.4–2.5 cm long, 0.1–0.2 mm gross, with 2 bracts 0.7–1.6 mm long, glabrous, membranaceous, hyaline; rachis 0.3–1.1 cm long, with 2–6, successive, secund flowers, 1–3 open at a time. **Floral bracts** 0.6–1.4 mm long, obliquely tubular-infundibuliform, obtuse, shortly apiculate, membranaceous, glabrous, hyaline suffused with purple. **Flowers** 3.2–4.7 mm × 1.8–2.9 mm; sepals and petals white-translucent with magenta lines, the apex of the dorsal sepal dark magenta to purple; lip magenta to purple; column white, dorsally suffused with magenta, foot magenta; anther white. **Sepals** concave, axially carinate, glabrous; dorsal sepal 2.8–4.5 mm × 1.4–2.2 mm, oblong–elliptic to oblong–ovate, acute, 3-veined, the apex forming a calyptra, papillar-glandulous inside, 0.5–0.7 mm × 0.5–0.8 mm; lateral sepals 3.2–4.7 mm × 1.9–3.0 mm, connate into an elliptic to ovate, obtuse, shortly bifid synsepal, 4-veined, gibbose in the base. **Petals** 1.5–2.8 mm × 0.5–0.9 mm, lanceolate, acute, straight, 1-veined. **Lip** 1.8–2.5 mm × 0.7–1.1 mm, fleshy, elliptic to elliptic–oblong, rounded, 3-veined, minutely papillar-glandulous, with a pair of submarginal calli, channeled between them. **Column** 1.5–1.7 mm long, 0.5–0.7 mm width, wings narrowly triangular, acute, entire, 0.4–0.6 mm × 0.2–0.3 mm; clinandrium obtuse, entire; foot 0.9–1.1 mm long, 0.3–0.5 mm width, obtuse, with two globular, high calli at the base. **Stigmatic cavity** 0.2 mm × 0.3 mm, elliptic–oblong; rostellum laminar, oblong. **Anther** 0.4–0.5 mm long, 0.3–0.4 mm width, ovoid. **Pollinia** 0.2–0.3 mm × 0.2–0.25 mm, obovoid. **Ovary** 0.6–1.5 mm long, 0.3–0.5 mm gross, obconical, straight, glabrous, green suffused with magenta; pedicel 0.8–2.8 mm long, 0.1–0.2 mm gross, glabrous. **Capsule** 2.8–4.0 mm long, 2.0–2.3 mm gross, elliptic–obovoid, yellowish-green suffused with purple (Figure 37 and Figure 38).

Distribution: Mexico, Guatemala, Belize and Honduras. In Mexico, the species is distributed in the states of Chiapas, Tabasco, Oaxaca and Veracruz. It occurs in the Floristic Provinces of Costa del Golfo de México, Costa Pacífica, Serranías Meridionales and Valle de Tehuacán.

**Figure 39:**
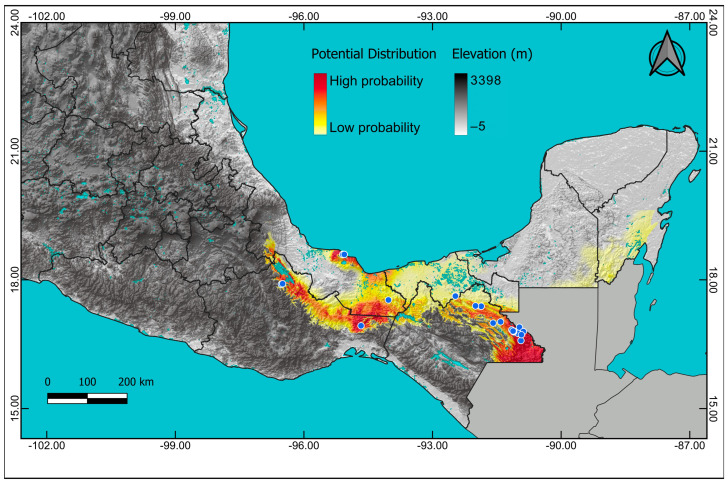
Potential distribution of *Specklinia pisinna* in Mexico and the location of currently known occurrence records (blue dots). Map by Ethian Licona.

The accepted potential distribution model for *S. pisinna* yields an AUC = 0.948, where the most influential environmental variables were annual precipitation (43.4%), the Digital Elevation Model of Mexico (15.1%) and temperature annual range (13.9%). The potential distribution area was estimated at 60,749.50 km^2^, representing approximately 3.09% of Mexico’s total territory (Figure 39).

Habitat: Epiphyte on *Brosimum alicastrum*, *Cynometra retusa*, *Dussia mexicana* (Standl.) Harms, *Rhizophora mangle* and *Terminalia amazonia*; it has also been recorded on *Mangifera indica* and *Tamarindus indica* L. The species grows in the evergreen tropical forest, semi-deciduous tropical forest, savanna grassland, mangrove forest, riparian vegetation and secondary vegetation derived from these forest types, at elevations between 90 and 860 m.

Phenology: Flowering occurs throughout the year. Mature fruits have been observed year-round.

Conservation status: In the assessment of its conservation status following the MER criteria [78], a score of 1.25 was obtained, therefore it is not necessary to include it in any Mexican risk category. In the evaluation under IUCN criterion B, it achieved an EOO of 23,634.983 km^2^ and an AOO of 60.00 km^2^, suggesting that this species should be classified as Near Threatened (NT).

Additional commentaries: *Specklinia pisinna* can be recognized by its prostrate habit; elliptic–obovate, obtuse to rounded, marginate leaves; short, lax, erect inflorescences with the peduncle longer than the leaf; successive, white-translucent flowers with magenta lines; dorsal sepal oblong–elliptic with an apical calyptra; synsepal elliptic, obtuse; petals lanceolate, acute; lip elliptic, rounded, magenta to purple.

This species is part of the *S. digitale* complex, the morphological differences among the species of this complex are summarized in Table 3.

Examined specimens: MEXICO. Chiapas: Ocosingo, 10 km al SE de Crucero Corozal, camino a Boca Lacantún, 18.08.1984, *E. Martínez 7431* (MEXU!); Lacanjá-Chanzayab, 25 km al SW de Crucero Corozal, camino Palenque-Boca Lacantum, 07.09.1985, *E. Martínez 13423* (MEXU!); Crucero Corozal, 06.11.1985, *E. Martínez 14949* (MEXU!); Lacanjá-Chanzayab, 25 km al SW de Crucero Corozal, camino Palenque-Boca Lacantum, 07.11.1985, *E. Martínez 15057* (MEXU!, MO!); cañada húmeda entre Nahá y Lacandón, 55 km al SE del Crucero de Chancalá, 27.04.1989, *M.A. Soto Arenas 5657* (AMO!); Monumento Natural Yaxchilán, porción N de la Omega, 23.07.1998, *A. Rincón 989* (MEXU!); Monumento Natural Yaxchilán, porción N de la Omega, 25.08.1998, *A. Rincón 1031* (MEXU!); al S del Crucero San Javier, 24.08.2002, *G. Aguilar 2271, A. Chambor, C. Chancayun, C.H. Ramos, E. Martínez & J. Abascal* (MEXU!); 0.4 km al N del banco de grava de San Javier, 23.11.2002, *D. Álvarez 2472, A. Aguilar & E. Martínez* (MEXU!); 1.3 km al SE de San Javier, 27.02.2003, *G. Aguilar 5912, A. Chambor & C. Chancayun* (MEXU!); 1.1 km al NE de San Javier, 08.10.2003, *G. Aguilar 8104, A. Chambor & C. Chancayun* (MEXU!); 1.1 km al S de San Javier, 24.11.2002, *D. Álvarez 2636, C. Chancayun, E. Martínez & S. Aguilar* (MEXU!); 1.1 km al E de San Javier, 15.11.2003, *D. Álvarez 6992, A. Aguilar & C. Jiménez* (MEXU!); 2.03 km al NW de San Javier, 07.09.2003, *G. Aguilar 7833 & C. Chancayun* (MEXU!); 1.47 km del Crucero de San Javier, 27.10.2003, *G. Aguilar 8272, A. Chambor & C. Chancayun* (MEXU!); al SW de Frontera Corozal, 16.04.2004, *G. Aguilar 9784 & M. Méndez* (MEXU!). Palenque, km 21 Palenque-Chancalá, undated, *E. Hágsater 4885* (AMO!). Salto de Agua, S of Palenque, near cascada Misol-Há, 15.01.1982, *D. Breedlove 57322* (CAS!). Oaxaca: San Felipe Usila, sin localidad, 03.10.1967, *G. Pollard p-211* (AMO!); cerca de Usila, por la vereda a la Presa Cerro de Oro, en la orilla del río Usila, 24.04.1992, *M.A. Soto Arenas 6618 & M. Hernández* (AMO!). Santa María Chimalapa, cerca del afloramiento cárstico en la cresta S del cañón del río del Corte, 4 km al N de S.M.C., 21.07.1996, *O. Rocha 311, G. Salazar, L. Cabrera & H. Hernandez* (AMO!). Tabasco: Macuspana, Agua Blanca, 30.03.2014, *D. López 2 & S. Zúñiga* (UJAT!); Agua Blanca, 30.03.2014, *D. López 3 & S. Zúñiga* (UJAT!). Veracruz: Las Choapas, rancho El Milagro, 5 km en línea recta al SW de la colonia Nueva Tabasqueña, 05.11.2003, *A. Franco 105* (XAL!). San Andrés Tuxtla, Estación Biológica Tropical Los Tuxtlas, cerro El Vigía, 16.11.1984, *G. Ibarra 2152, N. Pérez & A. Valiente* (MEXU!); Estación Biológica Tropical Los Tuxtlas, camino a la Laguna Escondida, 08.12.1984, *G. Salazar 224* (MEXU!); Laguna Azul, cerca del límite N de la Estación Biológica Tropical Los Tuxtlas, 09.02.1985, *G. Salazar 487 & S. Sinaca* (MEXU!); Laguna Zacatal, 2.5 km al NW de la Estación Biológica Tropical Los Tuxtlas, 28.03.1985, *S. Sinaca 26* (AMO!, MEXU!, XAL!); Estación Biológica Tropical Los Tuxtlas, Sendero Vigía 4, 21.12.2005, *T. Kromer 2733, A. Acebey & A. Pérez* (MEXU!, SEL!).

Additional records: MEXICO. Chiapas: Ocosingo, al N de Lacanjá, 12.04.2023, *J. Gallegos s/n* (https://www.inaturalist.org/observations/174823330. Accessed 1 Jun 2025); Santo Domingo, 07.01.2014, *M. Hernández s/n* (Photograph); Lacanjá-Chansayab, 27.03.2026, *J. Ojascastro s/n* (https://www.inaturalist.org/observations/346367966. Accessed 1 Jun 2025).

**10. *Specklinia solanoi*** E.Licona *sp. nov*.

**Figure 40:**
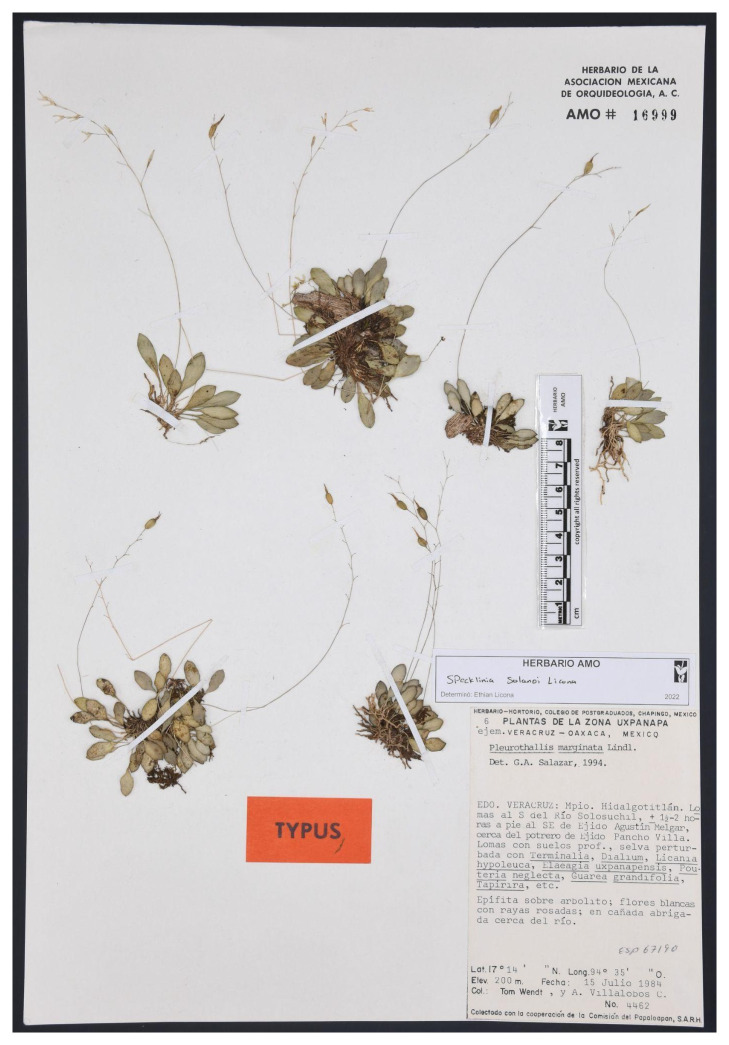
Holotype of *Specklinia solanoi, T. Wendt 4462 & A. Villalobos* (AMO-16999), courtesy of the Eric Hágsater AMO Herbarium.

HOLOTYPE: MEXICO. Veracruz: Uxpanapa, lomas al S del río Solosuchil, 1.5 horas a pie al SE del ejido Agustín Melgar, cerca del potrero del ejido Pancho Villa, 153 m, 15 July 1984, *T. Wendt 4462 & A. Villalobos* (AMO-16999! (Figure 40); isotypes: LL-111548!, MEXU-627539!, MO-5021484!).

Etymology: Named after Rodolfo Solano, a dedicated researcher of the orchids of the subtribe Pleurothallidinae in Mexico.

*Diagnosis: Similar to Specklinia grobyi, but differing by its longer inflorescence (up to 17.6 cm long vs. up to 12.0 cm long), with flowers initially distant from each other and approaching towards the apex (vs. flowers equidistant along the rachis); flowers yellowish-white, concolorous (vs. bright yellow with red lines on the dorsal sepal); dorsal sepal oblong–ovate (vs. lanceolate); synsepal oblong–lanceolate, acute (vs. oblong–obovate, obtuse); petals oblong–obovate, obtuse (vs. lanceolate, acuminate) and a lip oblong–obovate (vs. oblong–ligulate).*

**Figure 41:**
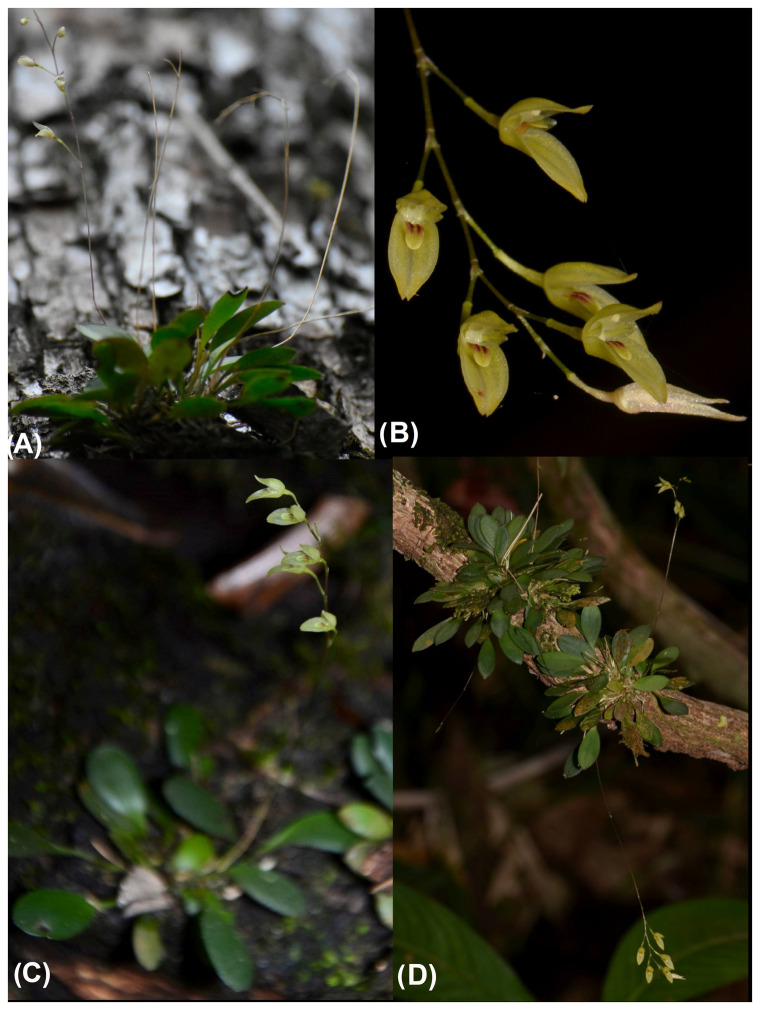
Photographs of *Specklinia solanoi*. (**A**) Specimen in its habitat, Carmen, Campeche. Photograph by Juvenal1804 (iNaturalist). (**B**,**D**) Specimen in its habitat, Ocosingo, Chiapas. Photographs by Gerardo Salazar, based on *G. Salazar et al., 8616* (MEXU). (**C**) Specimen in its habitat, Othón P. Blanco, Quintana Roo. Photograph by León Ibarra.

**Figure 42:**
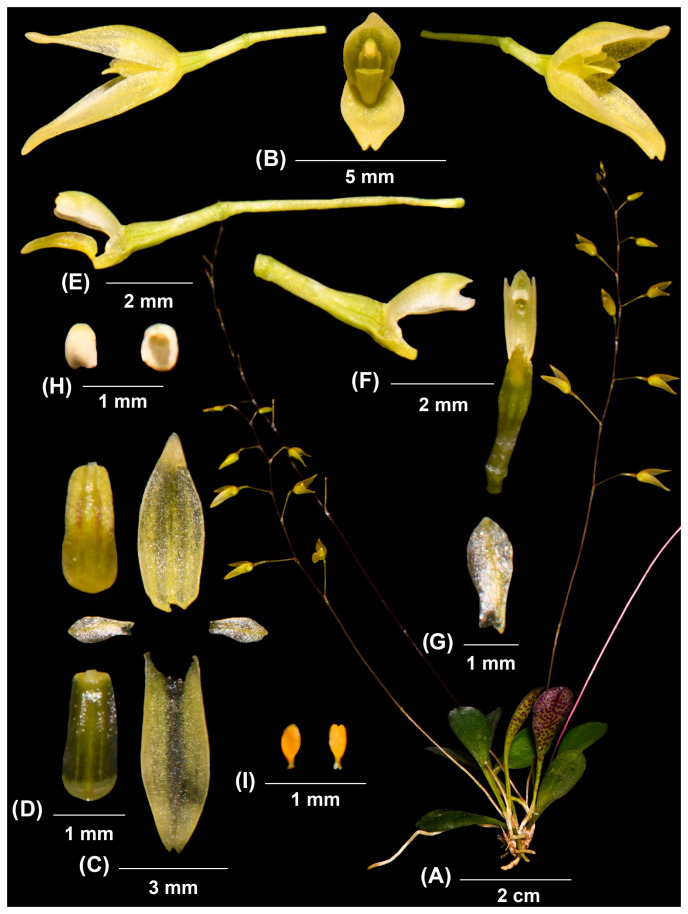
LCDP of *Specklinia solanoi*. (**A**) Habit. (**B**) Flower, ¾, frontal and lateral views. (**C**) Dissected flower. (**D**) Lip, dorsal and ventral views. (**E**) Column, lip and pedicelate ovary in lateral view. (**F**) Column, lateral and ventral views. (**G**) Petal. (**H**) Anther, dorsal and ventral views. (**I**) Pollinia, lateral and frontal views. Plate by Ethian Licona, based on *R. Solano & M.A. Soto-Arenas 817* (AMO).

**Herb** cespitose, ascending to prostrate, up to 4.0 cm tall without the inflorescence. **Roots** 0.4–0.8 mm gross. **Rhizome** 1.5–4.0 mm long between adjacent stems. **Stem** 3.0–12.5 mm long, 0.3–0.6 mm gross, sheaths 1.9–7.2 mm long. **Leaf** 6.0–25.0 mm × 3.4–9.2 mm, obovate to elliptic–oblanceolate, obtuse, succulent, biconvex, marginate, adaxially verrucose, abaxially glabrous, the apical mucro 0.3–0.4 mm long, light green, sometimes suffused with purple, with purple dots abaxially, abruptly attenuated into a petiole 1.5–12.4 mm long, 0.3–0.5 mm gross. **Inflorescence** 5.2–17.6 cm long, erect, elongated, lax, glabrous; spathaceous bract 0.8–1.5 mm long; peduncle longer than the leaf, 3.8–12.2 cm long, 0.4–0.5 mm gross, with 3 bracts 1.6–3.0 mm long, glabrous, membranaceous, hyaline; rachis 1.4–5.4 cm long, with 3–15, simultaneous, distichous flowers, perpendicular to the rachis axis, initially distant in the base of the rachis, then approaching towards the apex. **Floral bracts** 0.5–3.0 mm long, obliquely tubular-infundibuliform, obtuse, shortly apiculate, membranaceous, glabrous, hyaline suffused with purple. **Flowers** 3.3–6.0 mm × 1.4–2.3 mm; sepals and petals yellowish-white to greenish-yellow, the petals sometimes with a reddish line; lip greenish-yellow, with the calli red and the glenion-like depression yellow; column yellowish-white, foot yellow; anther white. **Sepals** concave, axially carinate, glabrous; dorsal sepal 2.9–5.4 mm × 1.2–2.2 mm, oblong–ovate to oblong–lanceolate, acuminate, 3-veined, with a triangular–ovate apical thickening, 0.9–1.1 mm × 0.4–0.5 mm; lateral sepals 3.3–6.0 mm × 1.5–2.6 mm, connate into an oblong–lanceolate, acuminate, shortly bifid synsepal, 4-veined, gibbose in the base, with two obliquely triangular–lanceolate apical thickenings, 1.0–1.5 mm × 0.4–0.5 mm. **Petals** 1.4–2.2 mm × 0.4–0.8 mm, obliquely oblong–obovate, obtuse, minutely apiculate, slightly falcate, attenuated in the base, 1-veined. **Lip** 1.5–2.4 mm × 0.6–1.1 mm, fleshy, oblong–obovate, obtuse, 3-veined, minutely papillar-glandulous, with a pair of submarginal calli, channeled between them, and a glenion-like depression near the apex. **Column** 1.5–2.2 mm long, 0.4–0.8 mm width; wings obliquely triangular, obtuse, entire, slightly falcate, 0.5–0.7 mm × 0.3–0.4 mm; clinandrium obtuse, entire; foot 0.8–1.2 mm long, 0.3–0.5 mm width, acute, with two globular, high calli at the base. **Stigmatic cavity** 0.2 mm × 0.3 mm, elliptic–oblong; rostellum laminar, oblong. **Anther** 0.4–0.6 mm long, 0.4–0.5 mm width, elliptic–ovoid. **Pollinia** 0.3–0.4 mm × 0.2–0.3 mm, obovoid. **Ovary** 1.0–4.0 mm long, 0.3–0.7 mm gross, obconical, straight, glabrous, yellowish-green suffused with purple; pedicel 1.3–7.5 mm long, 0.2–0.3 mm gross, glabrous. **Capsule** 4.2–7.5 mm long, 1.7–3.5 mm gross, elliptic–obovoid, yellowish-green suffused with purple (Figure 41 and Figure 42).

Distribution: Mexico, Guatemala and Belize, probably also Honduras. In Mexico, the species is distributed in the states of Campeche, Chiapas, Oaxaca, Tabasco, Quintana Roo and Veracruz. It occurs in the Floristic Provinces of Costa del Golfo de México, Costa Pacífica and Peninsula de Yucatán.

**Figure 43:**
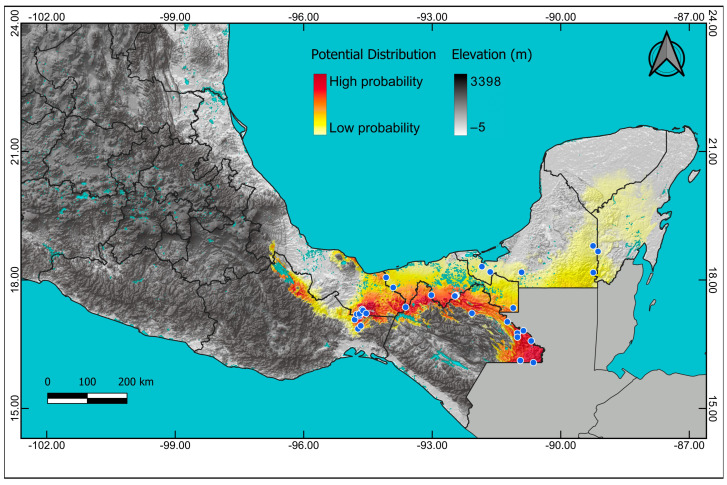
Potential distribution of *Specklinia solanoi* in Mexico and the location of currently known occurrence records (blue dots). Map by Ethian Licona.

The accepted potential distribution model for *S. solanoi* yields an AUC = 0.908, where the most influential environmental variables were precipitation of the warmest quarter (26.2%), the Digital Elevation Model of Mexico (15.4%) and precipitation of the coldest quarter (11.2%). The potential distribution area was estimated at 73,691.09 km^2^, representing approximately 3.75% of Mexico’s total territory (Figure 43).

Habitat: Epiphyte on *Brosimum alicastrum*, *B. costaricanum*, *Cojoba arborea* (L.) Britton & Rose, *Cryosophila stauracantha*, *Dialium guianense* (Aubl.) Sandwith, *Haematoxylum campechianum*, *Inga vera* Willd., *Lonchocarpus guatemalensis*, *Metopium brownei*, *Rhizophora mangle*, *Terminalia amazonia*, *T. buceras*, *Zanthoxylum kellermanii* P.Wilson and *Zygia* sp. The species grows in the evergreen tropical forest, semi-deciduous tropical forest, thorn forest, lithophytic xerophilous scrub, riparian vegetation, mangrove forest, savanna grassland and secondary vegetation derived from these forest types, at elevations between 1 and 670 m.

Phenology: Flowering occurs from March to December. Mature fruits have been observed from May to February.

Conservation status: In the assessment of its conservation status following the MER criteria [78], a score of 1.17 was obtained, therefore it is not necessary to include it in any risk category. In the evaluation under IUCN criterion B, it achieved an EOO of 113,342.097 km^2^ and an AOO of 124.00 km^2^, suggesting that this species should be classified as Least Concerned (LC).

Additional commentaries: *Specklinia solanoi* can be recognized by its obovate, obtuse, marginate leaves; inflorescence three or more times longer than the leaf, with flowers initially widely spaced from each other at the base and progressively close towards the apex, bearing yellowish-white, concolorous flowers, arranged perpendicular to the rachis; dorsal sepal oblong–ovate, acuminate; synsepal oblong–lanceolate, acute; petals oblong–obovate, obtuse, falcate; lip oblong–obovate, obtuse, greenish-yellow with red calli.

This species has also been confused with *S. grobyi*. The morphological differences between the Mexican species belonging to this complex are summarized in Table 2.

Examined specimens: MEXICO. Campeche: Candelaria, río Candelaria, Dos Arroyos, aprox. 30 km al SE de Candelaria, 17.05.2000, *G. Carnevali 6110, J. L. Tapia, F. May, D. Mondragón, C. Carrillo, P. Álvarez & L. Martínez* (CICY!). Palizada, El Vapor, 28.07.1939, *E. Matuda 3895* (AMES!, MICH!). Chiapas: Benemérito de las Américas, en Boca Lacantún, sobre la carretera fronteriza del sur, 26.10.1984, *E. Martínez 8731 & G. Aguilar* (MEXU!); Nuevo Orizaba, 25 km al W del vértice del río Chixoy, camino a Chajul, 22.06.1986, *E. Martínez 18874* (MEXU!). Chilón, 56 km al S de Palenque, sobre la carretera a Ocosingo, 01.05.1985, *A. Espejo 1570, R. Riba & B. Pérez* (MEXU!, UJAT!). Ocosingo, Estación Biológica de Chajul, vereda de la Estación a la Sabana, 18.06.1996, *R. Solano 817 & M.A. Soto Arenas* (AMO!); Chajul, vereda del puente hamaca a la sabana, 30.03.2014, *G. Salazar 8616, L. Cabrera, A. Barcenas & S. Akira* (MEXU!); Crucero Corozal, 170 km al SE de Palenque, camino a Boca Lacantún, 10.12.1984, *E. Martínez 10011 & G. Aguilar* (MEXU!); Crucero Corozal, 03.05.2004, *G. Aguilar 9931 & E. Martínez* (MEXU!); Zona Urbana, 1.33 km al NE de Frontera Corozal, 08.06.2004, *G. Aguilar 10517, F. Cruz & M. Mendéz* (MEXU!); 600 m al SW de Nuevo Jerusalén, 31.05.2002, *G. Aguilar 1268, D. Álvarez, J. Aguilar, P. Abascal & E. Martínez* (MEXU!); 7 km al SE de Bonampak, 16.05.1984, *E. Martínez 6358* (MEXU!). Oaxaca: Matías Romero Avendaño, 3.2 km al S del aserradero La Floresta, 16 km al S de La Esmeralda, lomas al N del río Verde, 28.05.1981, *T. Wendt 3339, A. Villalobos, J. García, I. Navarrete & J. Anguiano* (CHAPA!). Santa María Chimalapa, arroyo Chocolia, poblado Nicolás Bravo, cerca del rancho de Agustín Montero, aprox. 3–4 km al S de Río Alegre, Veracruz, 21.10.1983, *T. Wendt 4242, A. Montero & I. Almaráz* (CHAPA!); afloramientos rocosos en la cresta S del cañón del río del Corte, 4 km al NE de S.M.C., cerca de la vereda al Paso de la Cueva, 08.07.1985, *H. Hernández 1303* (MEXU!, XAL!); camino que va hacía el Río del Corte, 20.11.2005, *M. Martínez 131* (MEXU!); en el camino que va a Santa María Chimalapa, 20.11.2005, *M. Martínez 133* (MEXU!). Tabasco: Huimanguillo, Villa de Guadalupe, undated, *M. González 5* (UJAT!). Macuspana, Centro Recreativo Agua Blanca, 7 km del camino Villahermosa-Escárcega, junto al río, 04.05.1980, *M.R. Niño 2974 & C. Cowan* (CST!, NYBG!); Centro Recreativo Agua Blanca, 15.05.1982, *S. Zamudio 315* (IEB!, MEXU!, UJAT!); Centro Recreativo Agua Blanca, 27.05.1985, *S. Zamudio 1466* (IEB!, MEXU!); Centro Recreativo Agua Blanca, 25.09.1985, *A. Guadarrama 607 & M. Magaña* (MEXU!). Teapa, ejido Benito Juárez-Colorado, 09.05.1996, *A. Guadarrama 4152* (UJAT!). Tenosique, Rancho Atotonilco, al E del ejido El Tigre, 27.05.1988, *V. Ramón 56* (MEXU!, UJAT!); El Chotal, en el Tigre, de La Palma hacía el S por el río San Pedro, 02.06.1986, *A. Guadarrama 822* (UJAT!). Veracruz: Agua Dulce, límites entre Veracruz y Tabasco, 10.05.1980, *J. Calzada 6098* (IEB!, UC!, XAL!). Jesús Carranza, 41 km sobre la carretera a Hermanos Cedillo, a partir de la carretera Acayucan-Matías Romero, 17.07.2007, *A. Espejo 7004* (IEB!, XAL!). Uxpanapa, entre La Esmeralda y Poblado Cinco, 16.11.2005, *T. Kromer 2694, A. Acebey & M. Kessler* (MEXU!, SEL!, XAL!); 7 km del campamento Hermanos Cedillo, al SW, a mano derecha, 07.03.1974, *P. Valdivia 36* (XAL!); al NW del campamento Hermanos Cedillo, rumbo a La Escuadra, 4 km del campamento, sobre la brecha a mano izquierda 100 m, 15.03.1974, *P. Valdivia 136* (XAL!); 2.5 km del Campamento Hermanos Cedillo, frente a Paso Moral, 19.05.1974, *P. Valdivia 352* (MEXU!, XAL!); 1.5 km del Campamento Hnos. Cedillo, hacía La Escuadra, 24.06.1974, *P. Valdivia 971* (MEXU!, XAL!); brecha Campamentos Hermanos Cedillo-La Escuadra, 04.06.1974, *M. Vásquez 806* (MEXU!); al N del campamento Hermanos Cedillo, por el camino que va hacia Álvaro Obregón, 22.04.1975, *P. Valdivia 4016* (XAL!); al N del campamento Hermanos Cedillo, por el camino que va hacia Álvaro Obregón, 22.04.1975, *P. Valdivia 4018* (XAL!).

Additional records: MEXICO. Campeche: Calakmul, 6.7 km al SE de Unión 20 de julio, 28.07.2023, *I. Arellano s/n* (https://www.inaturalist.org/observations/166312636. Accessed 1 June 2025). Carmen, al N de San Antonio de la Sabana, 05.08.2017, *Juvenal1804 s/n* (https://www.inaturalist.org/observations/75695814. Accessed 1 June 2025). Chiapas: Ocozocoautla, Selva El Ocote, 01.07.2024, *R. García s/n* (https://www.inaturalist.org/observations/228132141. Accessed 1 June 2025). Quintana Roo: Othón P. Blanco, ejido Hermenegildo Galeana, 08.06.2017, *L. Ibarra s/n* (Photograph); 8 km al S de Nuevo Becar, por el camino a Caobas, 09.11.2023, *I. Arellano s/n* (https://www.inaturalist.org/observations/190543785. Accessed 1 June 2025). Tabasco: Huimanguillo, camino a Francisco Rueda, 01.01.2022, *M. González s/n* (Photograph).

**11. *Specklinia xoconochcana*** E.Licona, Solano & Soto-Trejo *sp. nov.*

**Figure 44:**
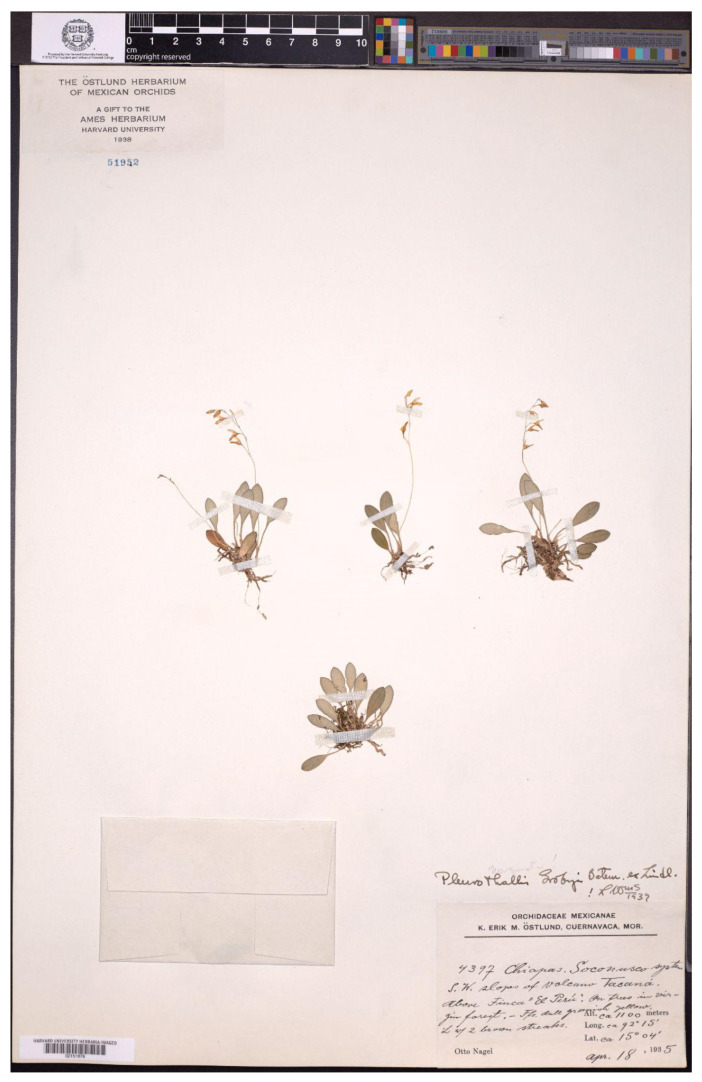
Holotype of *Specklinia xoconochcana, O. Nagel sub Östlund 4397* (AMES-2151878), courtesy of The Orchid Herbarium of Oakes Ames of Harvard University.

HOLOTYPE: MEXICO. Chiapas: Cacahoatán, Soconusco System, SW slopes of volcano Tacaná, above finca El Perico, 1141 m, 18 April 1935, *O. Nagel sub Östlund 4397* (AMES-2151878! (Figure 44)).

Etymology: From the Nahuatl *Xoconochco*, “place of the sour prickly pears”, referring to the region of El Soconusco, Chiapas, where this species inhabits.

*Diagnosis: Similar to Specklinia grobyi, but differing by its oblanceolate leaves (vs. obovate); inflorescence with a peduncle as long as the leaf (vs. peduncle longer than the leaf); flowers secund, white, concolorous (vs. flowers distichous, bright yellow with red lines); dorsal sepal lanceolate, recurved (vs. ovate, straight); synsepal oblong–oblanceolate, recurved (vs. oblong–lanceolate, straight); and a lip oblong–obovate, as long as the petals (vs. oblong–ligulate, longer than the petals).*

**Figure 45:**
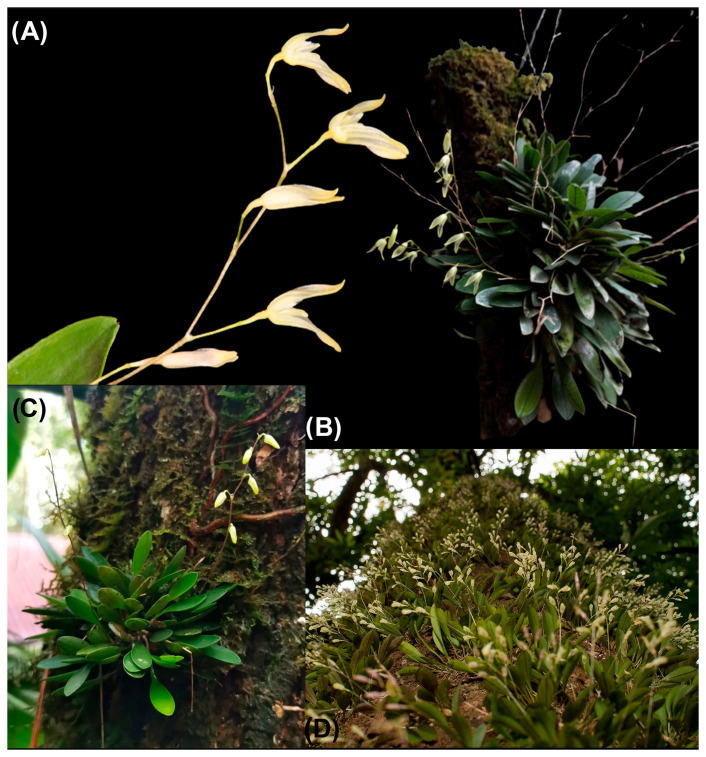
Photographs of *Specklinia xoconochcana*. (**A**,**B**) Specimen cultivated in Mexico City, from Chiapas. Photographs by Ethian Licona. (**C**) Specimen in its habitat, San Pedro Carchá, Guatemala. Photograph by Edgar Mó. (**D**) Specimen in its habitat, Tuzantán, Chiapas. Photograph by Eduardo Chamé.

**Figure 46:**
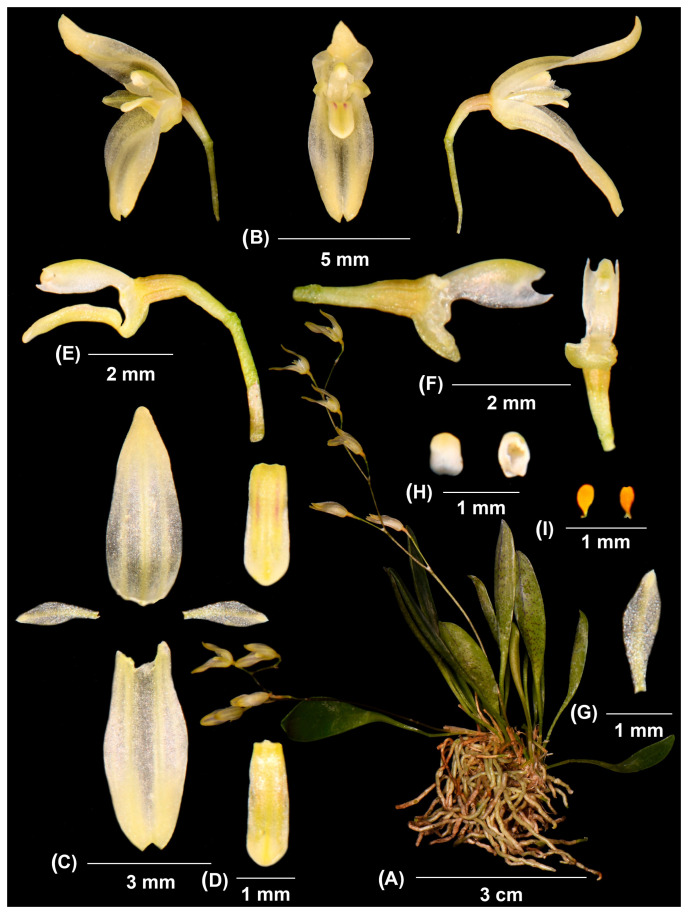
LCDP of *Specklinia xoconochcana*. (**A**) Habit. (**B**) Flower, ¾, frontal and lateral views. (**C**) Dissected flower. (**D**) Lip, dorsal and ventral views. (**E**) Column, lip and pedicelate ovary in lateral view. (**F**) Column, lateral and ventral views. (**G**) Petal. (**H**) Anther, dorsal and ventral views. (**I**) Pollinia, lateral and frontal views. Plate by Ethian Licona, based on a specimen from Chiapas, cultivated in the AMO Herbarium.

**Herb** shortly rhizomatous, ascending to erect, up to 4.6 cm tall without the inflorescence. **Roots** 0.4–0.8 mm gross. **Rhizome** 1.5–4.0 mm long between adjacent stems. **Stem** 2.5–16.0 mm long, 0.3–0.6 mm gross, sheaths 2.5–7.5 mm long. **Leaf** 8.2–30.0 mm × 4.0–9.0 mm, oblanceolate to elliptic–oblanceolate, obtuse, succulent, biconvex, marginate, adaxially verrucose, abaxially glabrous, the apical mucro 0.3–0.5 mm long, light green, sometimes suffused with purple, sometimes with purple dots abaxially, abruptly attenuated into a petiole 1.5–10.0 mm long, 0.3–0.6 mm gross. **Inflorescence** 2.5–9.9 cm long, erect, elongated, lax, glabrous; spathaceous bract 0.5–0.8 mm long; peduncle as long or shorter than the leaf, 2.0–4.9 cm long, 0.2–0.5 mm gross, with 2–3 bracts 1.6–2.0 mm long, glabrous, membranaceous, hyaline; rachis 0.5–5.0 cm long, with 3–8, simultaneous, secund flowers, perpendicular to the rachis axis. **Floral bracts** 0.8–3.0 mm long, obliquely tubular-infundibuliform, obtuse, shortly apiculate, membranaceous, glabrous, hyaline suffused with purple. **Flowers** 3.0–6.4 mm × 1.1–2.6 mm; sepals, petals, lip, column and anther white to yellowish-white, concolorous; the lip with magenta calli and the glenion-like depression yellow. **Sepals** concave, axially carinate, glabrous, recurved at the apices; dorsal sepal 3.0–5.8 mm × 1.2–2.5 mm, lanceolate to ovate, acuminate, 3-veined, with a triangular–ovate apical thickening, 1.4–2.6 mm × 0.4–0.5 mm; lateral sepals 3.0–6.4 mm × 1.3–2.9 mm, connate into an oblong–oblanceolate to elliptic–oblanceolate, obtuse, shortly bifid synsepal, 4-veined, gibbose in the base, with two obliquely oblanceolate apical thickenings, 1.8–2.5 mm × 0.4–0.6 mm. **Petals** 1.8–2.9 mm × 0.5–0.7 mm, obliquely lanceolate, acute, minutely mucronate, slightly falcate, attenuated in the base, 1-veined. **Lip** 1.8–2.9 mm × 0.5–1.2 mm, fleshy, oblong–obovate, obtuse, 3-veined, minutely papillar-glandulous, with a pair of submarginal calli, channeled between them, and a glenion-like depression near the apex. **Column** 2.0–2.3 mm long, 0.7–1.2 mm width, wings obliquely triangular, acuminate, falcate, entire, 0.6–1.4 mm × 0.3–0.4 mm; clinandrium obtuse, entire; foot 1.0–1.3 mm long, 0.4–0.7 mm width, obtuse, with two globular, high calli at the base. **Stigmatic cavity** 0.2 mm × 0.4 mm, elliptic–oblong; rostellum laminar, oblong. **Anther** 0.5–0.6 mm long, 0.4–0.5 mm width, elliptic–ovoid. **Pollinia** 0.3–0.4 mm × 0.2–0.3 mm, obovoid. **Ovary** 1.7–3.0 mm long, 0.3–1.0 mm gross, obconical, straight to recurved, glabrous, yellowish-green suffused with purple; pedicel 1.0–6.0 mm long, 0.2–0.3 mm gross, glabrous. **Capsule** 4.0–6.0 mm long, 1.1–2.5 mm gross, elliptic–obovoid, yellowish-green suffused with purple (Figure 45 and Figure 46).

Distribution: Mexico, Guatemala and probably El Salvador. In Mexico, the species is distributed only in the state of Chiapas. It occurs in the Floristic Provinces of Serranías Transístmicas and El Soconusco.

**Figure 47:**
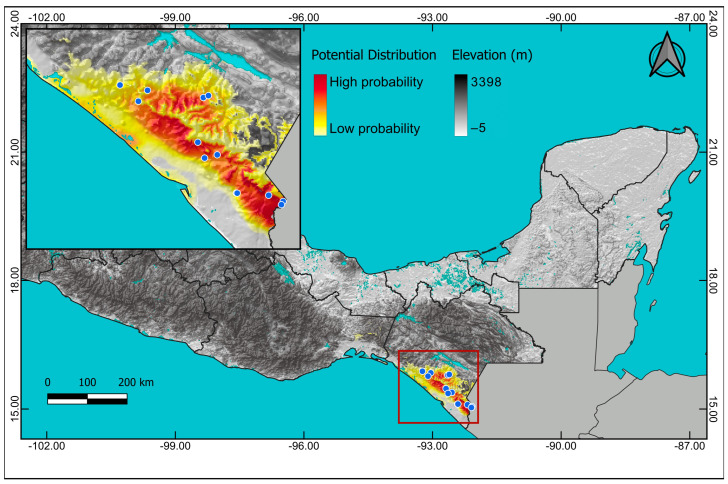
Potential distribution of *Specklinia xoconochcana* in Mexico and the location of currently known occurrence records (blue dots). Map by Ethian Licona.

The accepted potential distribution model for *S. xoconochcana* yields an AUC = 0.926, where the most influential environmental variables were precipitation of the wettest quarter (66.8%), precipitation of the warmest quarter (16.4%) and annual precipitation (7.7%). The potential distribution area was estimated at 9864.60 km^2^, representing approximately 0.50% of Mexico’s total territory (Figure 47).

Habitat: Epiphyte on *Brosimum costaricanum*, *Cojoba arborea*, *Dussia cuscatlanica* (Standl.) Standl. & Steyerm., *Liquidambar styraciflua*, *Quercus* sp., *Terminalia oblonga* (Ruiz & Pav.) Steud., and occasionally on *Coffea arabica*. The species grows in the evergreen tropical forest, semi-deciduous tropical forest, tropical mountain cloud forest, *Quercus* forest, riparian vegetation and coffee plantations, at elevations between 80 and 1560 m.

Phenology: Flowering occurs from April to December. Mature fruits have been observed from June to February.

Conservation status: In the assessment of its conservation status following the MER criteria [78], a score of 1.65 was obtained, which suggests that this species should be considered as a species Under Special Protection (Pr). In the evaluation under IUCN criterion B, it achieved an EOO of 3783.727 km^2^ and an AOO of 44.00 km^2^, suggesting that this species should be classified as Endangered (EN).

Additional commentaries: *Specklinia xoconochcana* can be recognized by its erect to ascending habit; oblanceolate, obtuse, marginate, long peciolate leaves; raceme with a peduncle as long as the leaf, with secund, white, concolorous flowers; sepals recurved at their apices; dorsal sepal lanceolate, acuminate; synsepal oblong–oblanceolate, obtuse; petals obliquely lanceolate, acute, falcate; and lip oblong–obovate, obtuse, yellowish-white with magenta calli, as long as the petals.

This species has long been confused with *Specklinia grobyi* or *S. marginata* by their morphological similarities. The morphological differences among the Mexican species of this complex are summarized in Table 2.

Examined specimens: MEXICO. Chiapas: Acacoyagua, 5 km del camino de ejido Las Golondrinas-Rosario Zacatonal, 09.03.2006, *R. Martínez 917* (HEM!). Escuintla, sin localidad precisa, 01.07.1938, *E. Matuda 2626* (MEXU!, MICH!); Reserva de la Biósfera El Triunfo, 02.03.1998, *A. Damon s/n* (ECO-TA-H!). La Concordia, Finca Arroyo Negro, 10.07.2014, *M. Díaz 1383* (CHIP!). Pijijiapan, El Cerrón, polígono área de amortiguamiento El Triunfo, 19.05.2005, *H. Reyes 20* (HEM!). Unión Juárez, al S de Unión Juárez, 01.07.1997, *A. Damon s/n* (ECO-TA-H!). Villa Corzo, Plan de Ayala, 28.05.1999, *F. Pérez 210* (HEM!).

Additional records: GUATEMALA. Alta Verapaz: Cobán, Aldea Cubilguitz, 30.08.2022, *E. Mó s/n* (Photograph). San Pedro Carchá, al NE de San Pedro Carchá, en la orilla del río Cahabón, 01.12.2022, *E. Mó s/n* (Photograph). MEXICO. Chiapas: Ángel Albino Corzo, al NE de Libertad del Pajal, cerca de la cascada El Pajal, 01.06.2020, *D. Manzano s/n* (https://www.inaturalist.org/observations/48842950. Accessed 28 May 2025); 6 km al NE de Pajal, 14.06.2023, *D. Manzano s/n* (https://www.inaturalist.org/observations/167558982. Accessed 28 May 2025). Huixtla, Piedra de Huixtla, 02.07.2024, *A. López s/n* (https://www.inaturalist.org/observations/227034042. Accessed 28 May 2025). Tuzantán, Jardín Botánico Regional El Soconusco, 03.04.2021, *S. López s/n* (Photograph); Guardiania, Jardín Etnobiológico de las Selvas del Soconusco, 26.05.2022, *A. López s/n* (https://www.inaturalist.org/observations/118876858. Accessed 28 May 2025). Unión Juárez, Orquideario Santo Domingo, undated, *A. Damon s/n* (Photograph).

***Specklinia* subgen. *Sylphia*** (Luer) Karremans, Phytotaxa, 272 (1): 26. 2016.

**12. *Specklinia echinata*** (L.O.Williams) Soto Arenas & Solano, Icon. Orchid., 5–6: t. 670. 2003.

**Figure 48:**
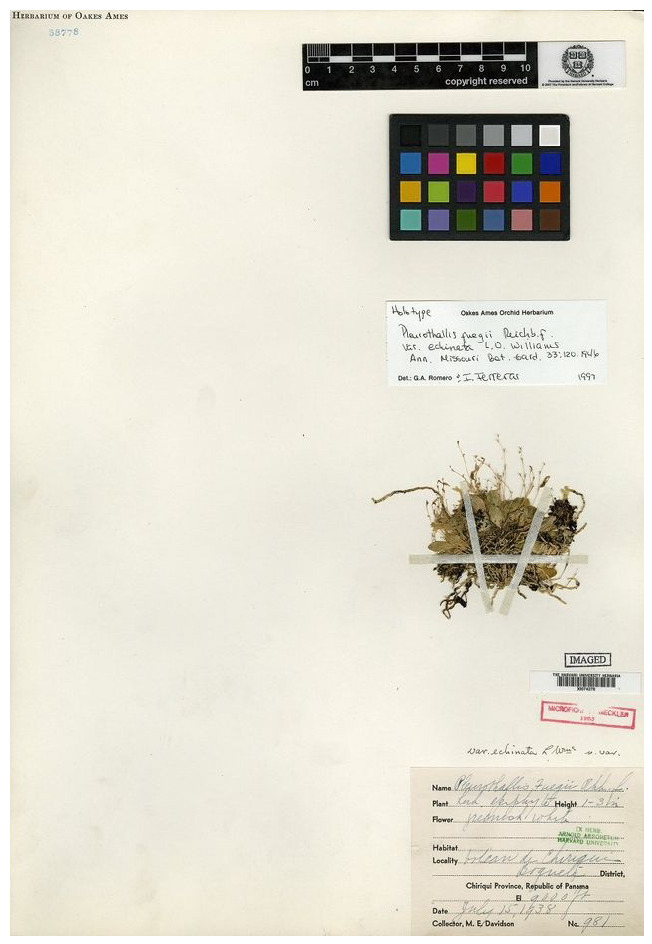
Holotype of *Pleurothallis fuegii* var. *echinata*, *M.E. Davidson 981* (AMES-74278), courtesy of The Orchid Herbarium of Oakes Ames of Harvard University.

Basionym: *Pleurothallis fuegii* var. *echinata* L.O.Williams, Ann. Missouri Bot. Gard., 33 (1): 120. 1946. TYPE: PANAMA. Chiriquí: Boquete District, Volcán de Chiriquí, about 2720 m, 15 July 1938, *M. Davidson 981* (holotype: AMES-74278! (Figure 48); isotype: AMES-74279! [includes an analytic drawing of the flower]).

Etymology: From the Latin *equinatus*, “echinate”, referring to the echinate surface of their ovaries.

**Figure 49:**
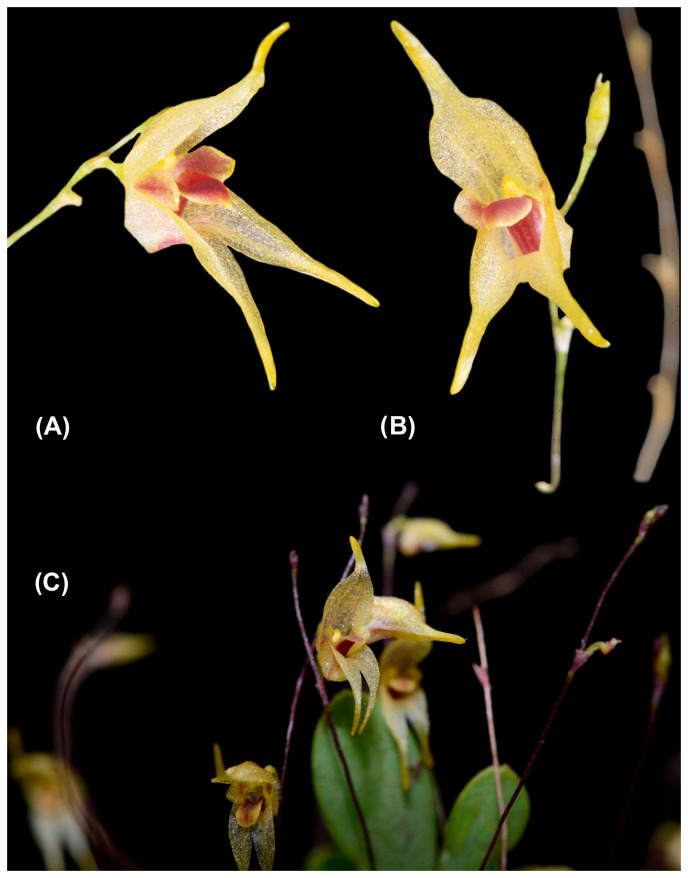
Photographs of *Specklinia echinata*. (**A**,**B**) Specimen cultivated in Mexico City. Photographs by Rodolfo Solano, based on *M.A. Soto Arenas & R. Solano 7309a* (AMO). (**C**) Specimen in its habitat, Alta Verapaz, Guatemala. Photograph by José Monzón and Edgar Mó.

**Figure 50:**
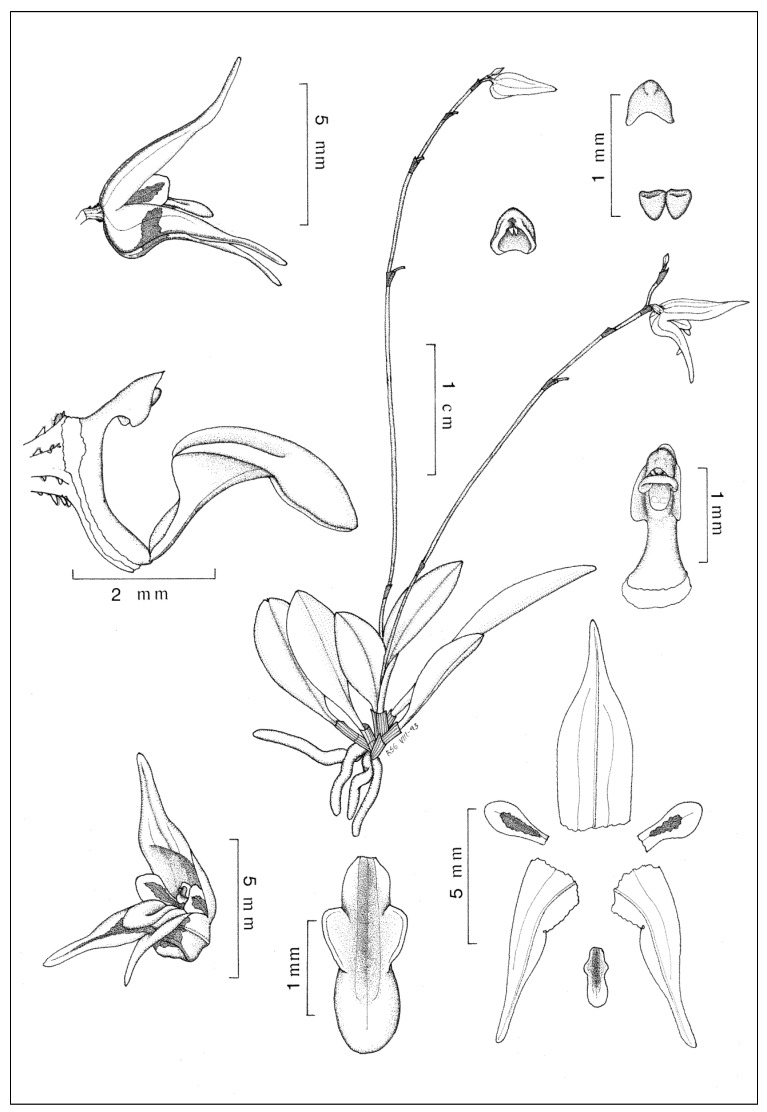
Illustration of *Specklinia echinata*. Drawing by Rodolfo Solano, based on *M.A. Soto Arenas 7309a & R. Solano* (AMO).

**Herb** cespitose, erect, up to 3.0 cm tall without the inflorescence. **Roots** 0.3–0.7 mm gross. **Rhizome** 0.5–1.2 mm long between adjacent stems. **Stem** 1.8–8.4 mm long, 0.2–0.4 mm gross, sheaths 1.4–5.0 mm long. **Leaf** 6.3–15.0 mm × 3.4–6.4 mm, elliptic–obovate to elliptic–lanceolate, obtuse, succulent, flattened, glabrous, the apical mucro 0.3–0.5 mm long, light green, abruptly attenuated into a petiole 2.0–7.0 mm long, 0.3–0.7 mm gross. **Inflorescence** 2.0–6.7 cm long, glabrous, flexuous, lax; spathaceous bract 0.6–0.9 mm long; peduncle longer than the leaf, 1.5–3.6 cm long, 0.1–0.3 mm gross, with 2–3 bracts 0.6–0.8 mm long, glabrous, membranaceous, hyaline; rachis 0.5–3.1 cm long, with 2–10, successive, secund flowers, spaced from one another by 3.0–11.5 mm, one open at a time. **Floral bracts** 0.8–1.8 mm long, obliquely tubular-infundibuliform, obtuse, shortly apiculate, membranaceous, glabrous, hyaline suffused with purple. **Flowers** 5.0–7.8 mm × 2.6–4.4 mm; sepals and petals yellowish-white, purple along their veins; lip dark purple and yellow at its apex; column and foot greenish-yellow, suffused with purple; anther yellow. **Sepals** concave, axially carinate, glabrous, caudate, the margins cellular–papillose, recurved in the apex; dorsal sepal 5.2–7.8 mm × 1.2–2.9 mm, oblong–ovate to oblong–lanceolate, 3-veined, cauda 2.0–2.5 mm long, 0.2–0.5 mm gross; lateral sepals 5.0–7.3 mm × 1.4–2.4 mm, connate by the basal ⅓, divergent towards the apex, each one oblong–lanceolate, 2-veined, gibbose in the base, caudae 1.5–2.2 mm long, 0.2–0.4 mm gross. **Petals** 1.5–2.7 mm × 0.6–1.3 mm, oblong–obovate to obovate, obtuse, 1-veined. **Lip** 2.2–3.0 mm × 1.5–2.2 mm, fleshy, 3-lobulate, 3-veined, minutely papillar-glandulous; lateral lobes obliquely elliptic–obovate, erect, the margins recurved, channeled between them; mid lobe obovate, obtuse, recurved. **Column** 1.3–1.8 mm long, 0.3–0.6 mm width; wings obliquely triangular, obtuse, retrorse, entire, 0.4–0.5 mm × 0.3–0.4 mm; clinandrium obtuse, entire; foot 1.6–2.0 mm long, 0.3–0.5 mm width, sub-terete, obtuse. **Stigmatic cavity** 0.25 mm × 0.3 mm, elliptic–oblong; rostellum laminar, oblong. **Anther** 0.4–0.5 mm long, 0.3–0.4 mm width, elliptic–ovoid. **Pollinia** 0.2–0.3 mm long and width, obovoid. **Ovary** 0.5–1.0 mm long, 0.3–0.6 mm gross, obconical, strongly recurved, verrucose–papillose, yellowish-green suffused with purple; pedicel 0.8–2.4 mm long, 0.2–0.3 mm gross, glabrous. **Capsule** 3.0–4.0 mm long, 1.6–2.0 mm gross, elliptic–obovoid, verrucose, yellowish-green suffused with purple (Figure 49 and Figure 50).

Distribution: Mexico, Guatemala, Honduras, Nicaragua, Costa Rica, Panama, and probably in El Salvador. In Mexico, the species is distributed in the states of Chiapas and Veracruz. It occurs in the Floristic Provinces of Costa del Golfo de México and Serranías Transístmicas.

**Figure 51:**
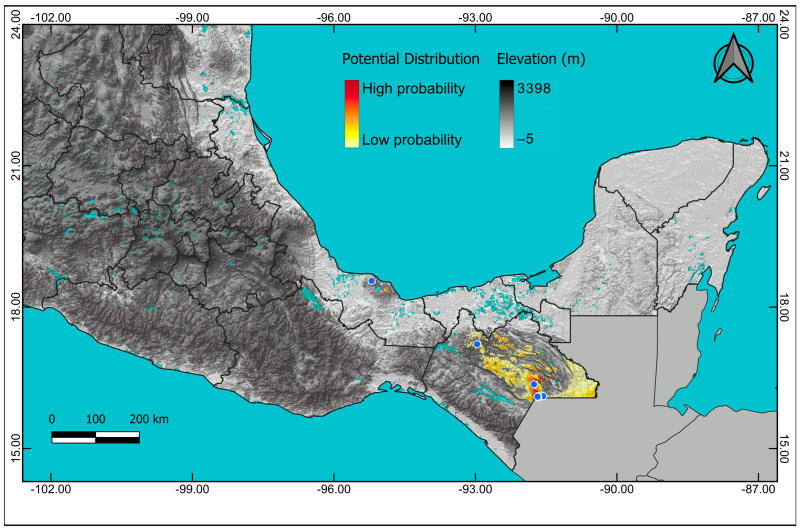
Potential distribution of *Specklinia echinata* in Mexico and the location of currently known occurrence records (blue dots). Map by Ethian Licona.

The accepted potential distribution model for *S. echinata* yields an AUC = 0.979, where the most influential environmental variables were temperature seasonality (53.1%), the Digital Elevation Model of Mexico (17.6%) and precipitation of the wettest quarter (16.5%). The potential distribution area was estimated at 9973.05 km^2^, representing approximately 0.51% of Mexico’s total territory (Figure 51).

Habitat: Epiphyte on *Alsophila* sp., *Clusia* sp., *Cyathea* sp., *Liquidambar styraciflua* and *Quercus* sp. The species grows in the evergreen tropical forest, tropical mountain cloud forest and *Quercus* forest, at elevations between 1100 and 1900 m.

Phenology: Flowering occurs from January to September. Mature fruits have been observed from April to November.

Conservation status: In the assessment of its conservation status following the MER criteria [78], a score of 1.61 was obtained, which suggests that this species should be considered as a species Under Special Protection (Pr). In the evaluation under IUCN criterion B, it achieved an EOO of 6044.997 km^2^ and an AOO of 32.00 km^2^, suggesting that this species should be classified as Vulnerable (VU).

Additional commentaries: *Specklinia echinata* can be recognized by its elliptic–obovate, obtuse leaves; flexuous, lax raceme, with a peduncle longer than the leaf; secund flowers spaced from each other, yellowish-white with purple on the veins; dorsal sepal oblong–ovate; lateral sepals connate in the basal ⅓, oblong–lanceolate; petals oblong–obovate, obtuse; lip 3-lobed, dark purple with the apex yellow; lateral lobes obliquely elliptic–obovate, erect; mid lobe obovate, obtuse; column with a body shorter than the foot; and ovary verrucose. The plants from Mexico exhibit a verrucose ovary rather than the echinate ovary observed in specimens from southern Central America.

Soto Arenas [84] cited *Specklinia montebellensis* as an unpublished name applied to specimens from Mexico. Subsequent examination of those specimens demonstrated that they corresponded to *S. echinata* [57], which at that time resulted in a new record for the Mexican Flora.

The morphological differences between *S. echinata* and the other Mexican species of *Specklinia* subg. *Sylphia* are summarized in Table 4.

Examined specimens: MEXICO. Chiapas: La Trinitaria, 1 km al N de Tziscao por el camino a Bonampak, 08.07.1985, *M.A. Soto Arenas 1318 & F. Maldonado* (AMO!); en el margen del lago Tziscao, cerca del puente de piedra, 04.02.1989, *M.A. Soto Arenas 4867, M. Hernández & E. Yañez* (AMO!); en el margen del lago Tziscao, cerca del puente de piedra, 04.02.1989, *M.A. Soto Arenas 4868, M. Hernández & E. Yañez* (AMO!); en el margen del lago Tziscao, cerca del puente de piedra, 04.02.1989, *M.A. Soto Arenas 4869, M. Hernández & E. Yañez* (AMO!); en el margen del lago Tziscao, cerca del puente de piedra, 04.02.1989, *M.A. Soto Arenas 4870, M. Hernández & E. Yañez* (AMO!); en el margen del lago Tziscao, cerca del puente de piedra, 04.02.1989, *M.A. Soto Arenas 4873, M. Hernández & E. Yañez* (AMO!); 20.4 km del camino Montebello-Amparo Agua Tinta, 10.08.1992, *M.A. Soto Arenas 7099 & R. Solano* (AMO!); km 20 del camino Montebello-Marqués de Comillas, 10.08.1992, *M.A. Soto Arenas 7100 & R. Solano* (AMO!); Parque Nacional Lagunas de Montebello, Cinco Lagos, 09.08.1992, *M.A. Soto Arenas 7309A & R. Solano* (AMO!). Las Margaritas, cerca de Nuevo Momón, 41.28 km al E de Las Margaritas, por el camino a Cruz del Rosario, 09.08.1992, *M.A. Soto Arenas 7357 & R. Solano* (AMO!). Rayón, mirador de Pueblo Nuevo, camino a Rayón, 11.04.1989, *E. Martínez 24177 & M.A. Soto Arenas* (MEXU!). Veracruz: San Andrés Tuxtla, cima del volcán San Martín Tuxtla, 14.08.2005, *T. Kromer 2522 & A. Acebey* (MEXU!).

Additional records: GUATEMALA. Baja Verapaz: Purulhá, 4 km al S de Purulhá, camino Guatemala-Cobán, cerca del Biotopo del Quetzal, 15.06.1985, *E. Martínez 13114 & O. Tellez* (MEXU!); 4 km al S de Purulhá, camino Guatemala-Cobán, cerca del Biotopo del Quetzal, 15.06.1985, *E. Martínez 13166 & O. Tellez* (MEXU!); Biotopo del Quetzal, 05.09.1988, *E. Martínez 23534, W. Stevens, A. Díaz & H. Droege* (MEXU!). MEXICO. Chiapas: La Trinitaria, en las cercanías de Cinco Lagos, Parque Nacional Lagunas de Montebello, 12.08.2017, *M. Egger s/n* (Photograph).

**13. *Specklinia fuegi*** (Rchb.f.) Solano & Soto Arenas, Icon. Orchid., 5–6: x-xi. 2003.

**Figure 52:**
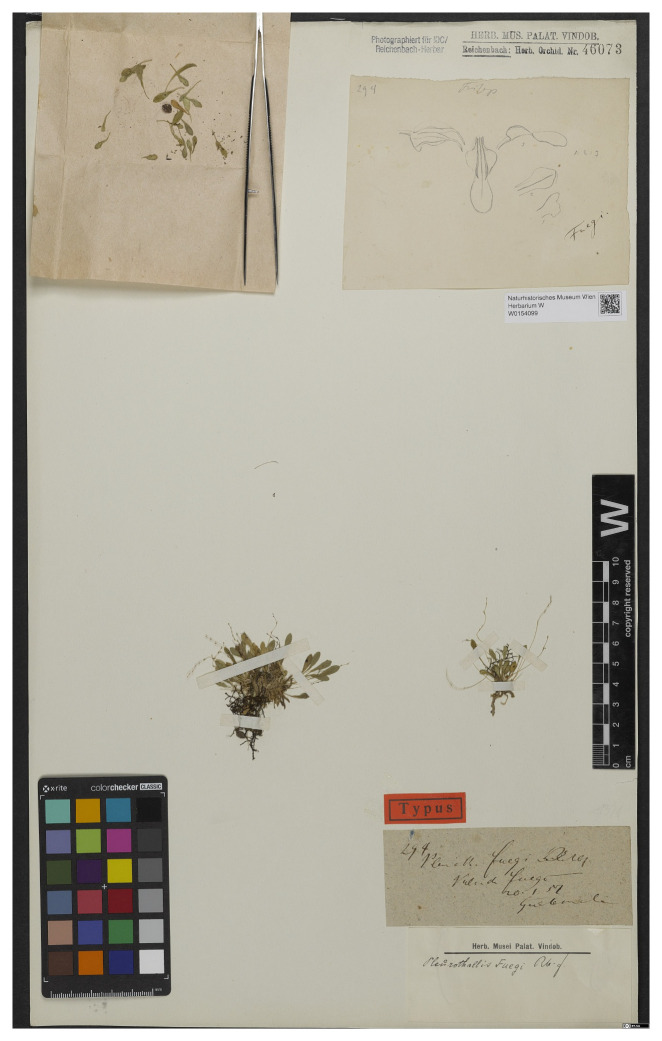
Lectotype of *Pleurothallis fuegi*, *H. Wendland 294* (W-154099), courtesy of the Herbarium of Naturhistorisches Museum Wien.

Basionym: *Pleurothallis fuegii* Rchb.f., Beitr. Orchid. K. C. Amer. 97–98. t. 10, Figure 11, Figure 12, Figure 13, Figure 14 and Figure 15. 1866. TYPE: GUATEMALA: Volcán de Fuego, 20 January 1857, *H. Wendland 294* (lectotype (designated by Dowe et al., [89]): W-154099 [includes an analytic drawing of the flower]! (Figure 52), isolectotype: GOET-8637!, GOET-8638!; drawing based on the type: AMES-74277!).

Homotypic synonyms: *Humboltia fuegii* (Rchb.f.) Kuntze, Revis. Gen. Pl., 2: 667. 1891; *Anathallis fuegi* (Rchb.f.) Pridgeon & M.W.Chase, Lindleyana, 16 (4): 248. 2001; *Specklinia fuegii* (Rchb.f.) Luer, Monogr. Syst. Bot. Missouri Bot. Gard., 95: 260. 2004, *nom. superfl.*; *Sylphia fuegi* (Rchb.f.) Luer, Monogr. Syst. Bot. Missouri Bot. Gard., 105: 228–229. 2006.

Etymology: named after the Volcán de Fuego in Guatemala, where the type was collected.

**Figure 53:**
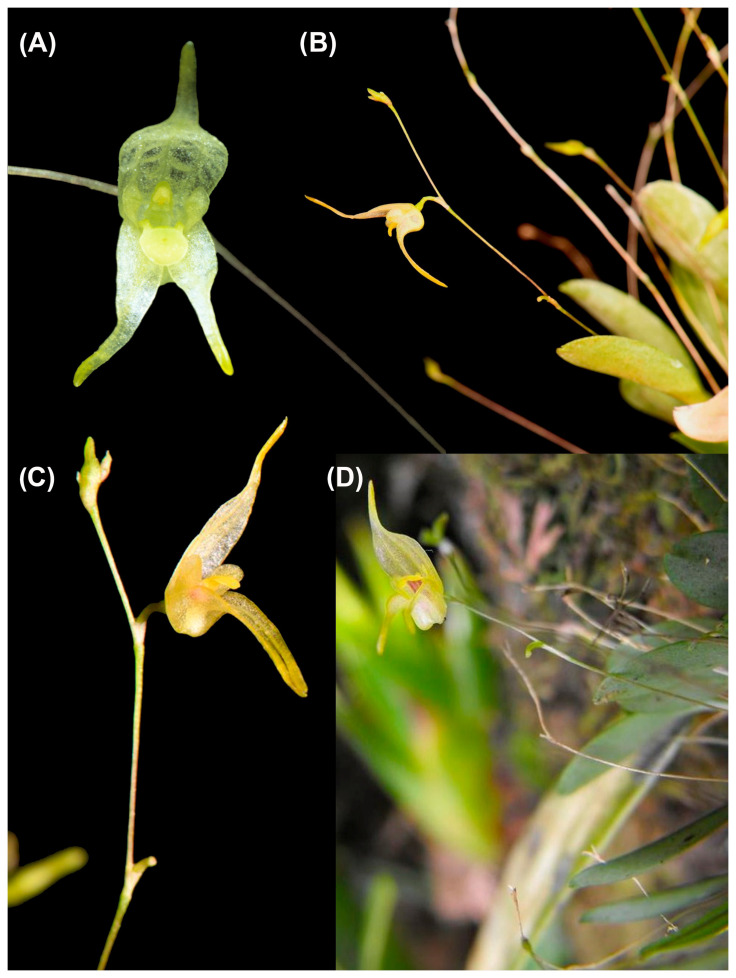
Photographs of *Specklinia fuegi*. (**A**) Specimen cultivated in Mexico City. Photograph by Rodolfo Solano, based on *M.A. Soto Arenas 10121* (AMO). (**B**,**C**) Specimen cultivated in Mexico City. Photographs by Ethian Licona and Gerardo Salazar, respectively, based on *G. Salazar 8407* (AMO). (**D**) Specimen in its habitat, Villaflores, Chiapas. Photograph by Eduardo Martínez Ovando.

**Figure 54:**
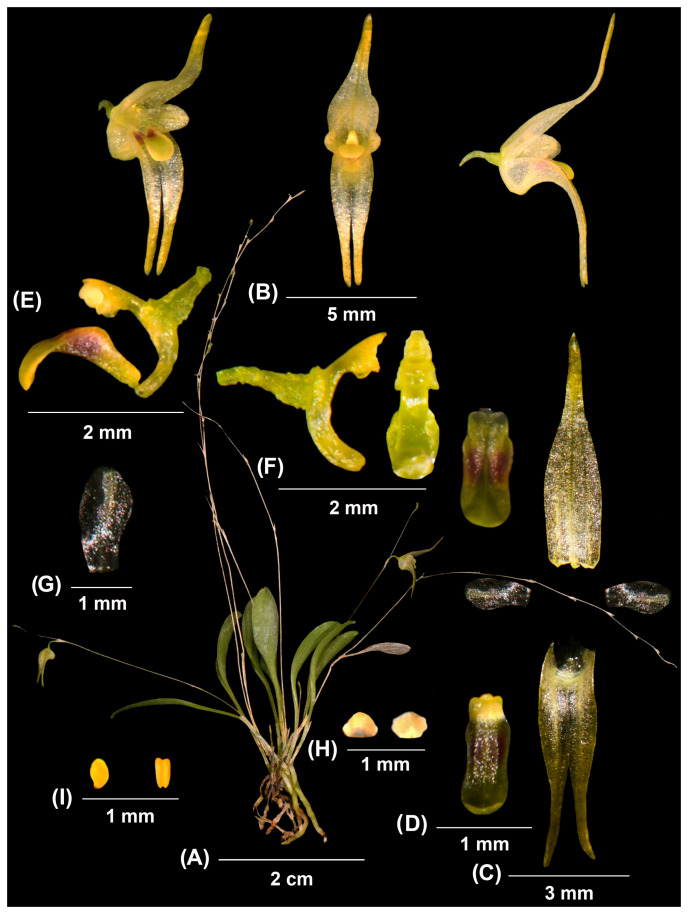
LCDP of *Specklinia fuegi*. (**A**) Habit. (**B**) Flower, ¾, frontal and lateral views. (**C**) Dissected flower. (**D**) Lip, dorsal and ventral views. (**E**) Column, lip and pedicelate ovary in lateral view. (**F**) Column, lateral and ventral views. (**G**) Petal. (**H**) Anther, dorsal and ventral views. (**I**) Pollinia, lateral and frontal views. Plate by Ethian Licona, based on *G. Salazar 8407* (AMO).

**Herb** cespitose, erect, up to 2.8 cm tall without the inflorescence. **Roots** 0.3–0.6 mm gross. **Rhizome** 0.5–2.0 mm long between adjacent stems. **Stem** 1.8–8.0 mm long, 0.3–0.5 mm gross, sheaths 1.0–5.3 mm long. **Leaf** 5.0–12.0 mm × 2.0–5.2 mm, lanceolate to elliptic–lanceolate, acute, succulent, flattened, glabrous, the apical mucro 0.2–0.3 mm long, light green, abruptly attenuated into a petiole 1.4–8.0 mm long, 0.3–0.6 mm gross. **Inflorescence** 1.5–7.6 cm long, flexuous, congested, glabrous; spathaceus bract 0.4–0.8 mm long; peduncle as long as the leaf, 1.0–2.3 cm long, 0.1–0.3 mm gross, with 2 bracts 0.7–1.0 mm long, glabrous, membranaceous, hyaline; rachis 0.5–4.3 cm long, with 4–18, successive, secund flowers, spaced from one another by 1.4–7.0 mm, one open at a time. **Floral bracts** 0.8–1.8 mm long, obliquely tubular-infundibuliform, obtuse, shortly apiculate, membranaceous, glabrous, hyaline suffused with purple. **Flowers** 3.3–6.5 mm × 1.4–3.0 mm; sepals and petals yellowish-white; lip greenish-yellow, with lateral lobes purple and apex yellow; column and foot greenish-yellow; anther yellowish-white. **Sepals** concave, axially carinate, glabrous, caudate, with margins cellular–papillose, recurved in the apex; dorsal sepal 4.0–6.5 mm × 1.0–2.3 mm, oblanceolate to oblong–obovate, 3-veined, cauda 1.3–1.8 mm long, 0.2–0.4 mm gross; lateral sepals 3.5–5.9 mm × 0.8–1.4 mm, connate in ½ of their length, sub-parallel towards the apex, each one narrowly lanceolate, 2-veined, gibbose in the base, caudae 1.0–2.2 mm long, 0.2–0.4 mm gross. **Petals** 1.5–2.1 mm × 0.5–1.2 mm, obliquely obovate, obtuse, 1-veined. **Lip** 1.7–2.5 mm × 0.8–1.8 mm, fleshy, 3-lobulate, 3-veined, minutely papillar-glandulous; lateral lobes narrowly elliptic, erect, the margins recurved, channeled between them; mid lobe oblong–obovate to oblong–elliptic, obtuse, recurved. **Column** 1.0–1.5 mm long, 0.5–0.6 mm width; wings obliquely triangular, obtuse, entire, retrorse, 0.4–0.5 mm × 0.2–0.3 mm; clinandrium obtuse, entire; foot 1.0–1.4 mm long, 0.2–0.4 mm width, sub-terete, obtuse. **Stigmatic cavity** 0.2 mm × 0.2 mm, elliptic–orbicular; rostellum laminar, oblong. **Anther** 0.4–0.5 mm long, 0.3–0.4 mm width, elliptic–ovoid. **Pollinia** 0.3–0.4 mm × 0.2–0.3 mm, obovoid. **Ovary** 0.5–1.0 mm long, 0.3–0.6 mm gross, obconical, strongly recurved, verrucose–papillose, yellowish-green suffused with purple; pedicel 0.8–2.3 mm long, 0.2–0.3 mm gross, glabrous. **Capsule** not seen (Figure 53 and Figure 54).

Distribution: Mexico, Guatemala, El Salvador, Honduras, Nicaragua, and probably in Costa Rica. In Mexico, the species is distributed in the states of Chiapas, Oaxaca and Veracruz. It occurs in the Floristic Provinces of Costa del Golfo de México, Costa Pacífica, Serranías Transístmicas and El Soconusco.

**Figure 55:**
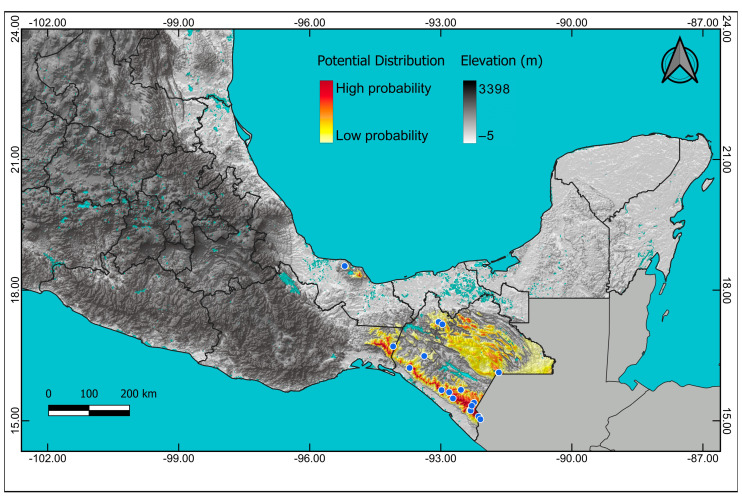
Potential distribution of *Specklinia fuegi* in Mexico and the location of currently known occurrence records (blue dots). Map by Ethian Licona.

The accepted potential distribution model for *S. fuegi* yields an AUC = 0.969, where the most influential environmental variables were the Digital Elevation Model of Mexico (37.6%), temperature seasonality (26.3%) and temperature annual range (19.0%). The potential distribution area was estimated at 22,429.48 km^2^, representing approximately 1.14% of Mexico’s total territory (Figure 55).

Habitat: Epiphyte on *Alsophila* sp., *Clusia* sp., *Cyathea* sp., *Liquidambar styraciflua* and *Quercus* sp., rarely as a lithophyte. The species grows in the evergreen tropical forest, *Quercus* forest, *Pinus-Quercus* forest and tropical mountain cloud forest, at elevations between 960 and 2380 m.

Phenology: Flowering occurs from January to September. Mature fruits have not been observed in Mexico.

Conservation status: In the assessment of its conservation status following the MER criteria [78], a score of 0.86 was obtained, therefore it is not necessary to include it in any risk category. In the evaluation under IUCN criterion B, it achieved an EOO of 46,334.072 km^2^ and an AOO of 80.00 km^2^, suggesting that this species should be classified as Least Concerned (LC).

Additional commentaries: *Specklinia fuegi* can be recognized by its lanceolate, acute leaves; flexuose, lax raceme, with a peduncle as long as the leaf; secund flowers closely spaced, yellowish-white, concolorous; dorsal sepal oblanceolate; lateral sepals connate for half their length, narrowly lanceolate; petals obovate, obtuse; lip 3-lobulate, greenish-yellow; lateral lobes purple, narrowly elliptic, erect; mid lobe oblong–obovate, obtuse, recurved; foot as long as the column body; and an ovary verrucose.

For a long time in Mexico, *Specklinia fuegi* was treated under a broad taxonomic circumscription that include the other two Mexican species here recognized in subgenus *Sylphia*, owing to their morphological similarities. Nevertheless, a consistent set of characteristics allows reliable identification of each taxon. These differences are summarized in Table 4.

Examined specimens: MEXICO. Chiapas: Ángel Albino Corzo, al NW de la Reserva El Triunfo, en el Cerro del Filo, 11.05.1982, *J. Calzada 8779, G. Cortés & G. Juárez* (MEXU!, XAL!); Reserva de la Biosfera el Triunfo, cerca al Campamento El Triunfo, 05.03.1993, *E. Hágsater s/n* (AMO!). Cacahoatán, 2 km al NW de Milán, 24.11.2007, *R. Jiménez 100* (ECO-TAH!); Ejido Benito Juárez El Plan, Reserva de la Biósfera Volcán Tacaná, 03.05.2008, *L. Jiménez 273* (ECO-TA-H!). El Porvenir, 5 km NE de Motozintla, 08.08.1986, *R. Fernández 3533* (MEXU!). La Concordia, Reserva El Triunfo, Polígono 1 Campo-Cipresal, 22.11.1990, *M. Heath 1355 & A. Long* (AMO!). La Trinitaria, Parque Nacional Lagunas de Montebello, Cinco Lagos, 09.08.1992, *M.A. Soto Arenas 7304 & R. Solano* (AMO!); N side of river-crossing beyond L. Pulido’s house, Montebello, 35 milles SE of Comitán, 17.01.1952, *M. Carlson 2285* (SEL!). Mapastepec, en los alrededores del ejido Ampliación Lagunas, 09.10.2007, *J. Martínez 1890* (HEM!). Motozintla, Boquerón-Buena Vista, 01.03.2008, *A. Damon s/n* (ECO-TA-H!); ejido Boquerón, en los alrededores de la brecha que lleva a la cascada, 15.06.2015, *M. Martínez 6044, S. Hernández & V. García* (HEM!); 13 km al O de Motozintla, camino a El Porvenir, 27.02.1988, *E. Martínez 22487, M. Sousa & A. Reyes-García* (MEXU!). Pantepec, km 105 de la carretera Tuxtla Gutiérrez-Villahermosa, aprox. 7 km arriba de Rayón, 11.09.1985, *E. Martínez s/n & M.A. Soto Arenas* (AMO!). Rayón, mirador de Pueblo Nuevo, camino a Rayón, 11.04.1989, *E. Martínez 24177 & M.A. Soto Arenas* (MEXU!). Villaflores, cerro Chumpipe, 28.05.1995, *J. Castillo 681* (CHIP!). Oaxaca: San Miguel Chimalapa, Cerro de La División, ca. 5 km al E de Benito Juárez, 39 km en línea recta al NNE de San Pedro Tapanatepec, al SW de la cima del cerro, 26.12.1984, *S. Maya 1133* (CHAPA!). Veracruz: San Andrés Tuxtla, Estación Biológica Tropical Los Tuxtlas, Volcán San Martín Tuxtla, undated, *G. Salazar 8407* (AMO!); cima del volcán San Martín Tuxtla, 14.08.2005, *T. Kromer 2522 & A. Acebey* (MEXU!, SEL!, XAL!).

Additional records: MEXICO. Chiapas: La Trinitaria, en las cercanías de Cinco Lagos, Parque Nacional Lagunas de Montebello, 12.08.2017, *M. Egger s/n* (Photograph). Unión Juárez, Orquideario Santo Domingo, undated, *A. Damon s/n* (Photograph). Villaflores, Cerro Brujo, undated, *E. Martínez-Ovando s/n* (Photograph).

**14. *Specklinia hagsateri*** (Luer) E.Licona, Solano & Soto-Trejo, *comb. nov.*

**Figure 56:**
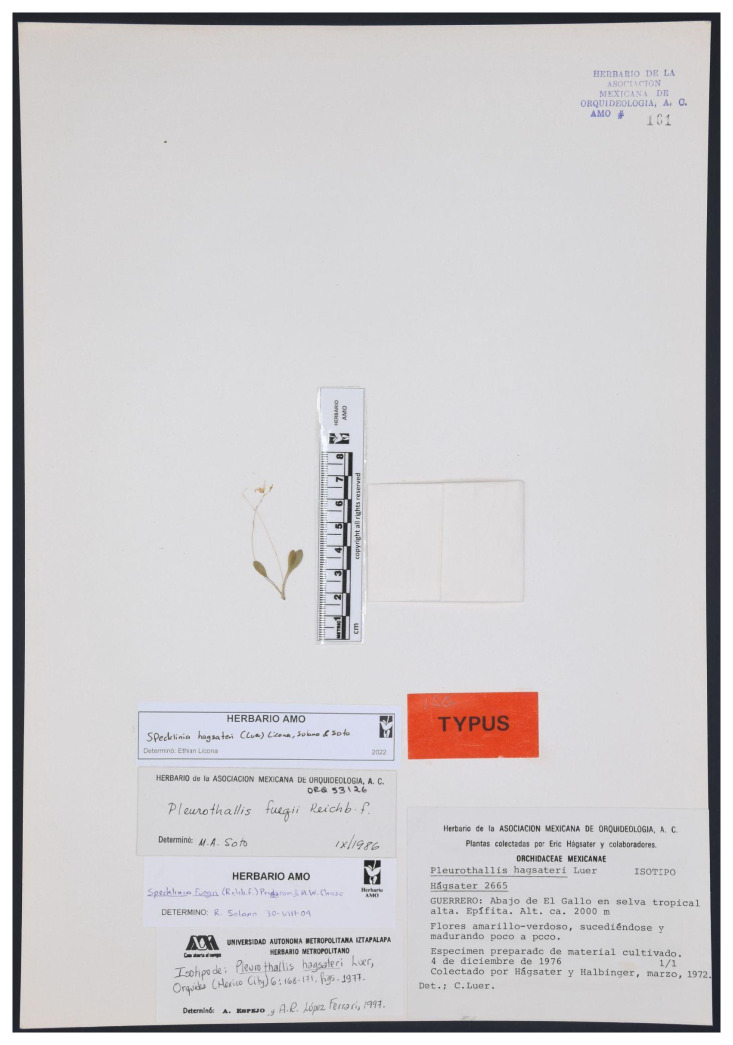
Isotype of *Pleurothallis hagsateri*, *E. Hágsater 2665 & F. Halbinger* (AMO-161), courtesy of the Eric Hágsater AMO Herbarium.

Basionym: *Pleurothallis hagsateri* Luer, Orquídea (Mexico City) n.s., 6 (6): 168–171. 1977. TYPE: MEXICO. Guerrero: [Atoyac de Álvarez] Epiphyte in tropical forest below El Gallo, ca. 2000 m, 01 March 1972. *E. Hágsater 2665 & F. Halbinger* (holotype: SEL, not seen; isotype: AMO-161! (Figure 56)).

Etymology: named after Eric Hágsater, world specialist of the genus *Epidendrum* and director of the AMO Herbarium.

**Figure 57:**
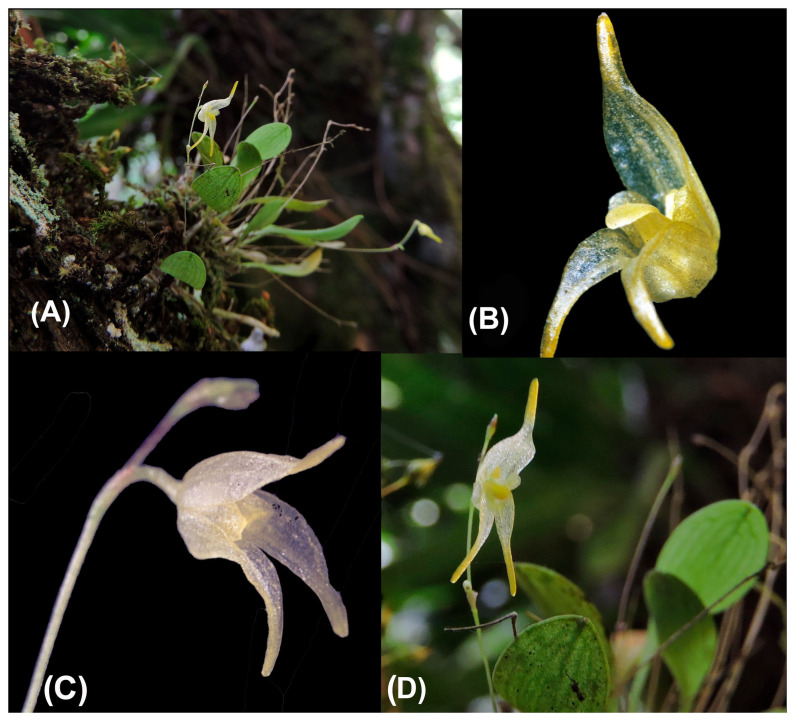
Photographs of *Specklinia hagsateri*. (**A**,**D**) Specimen in its habitat, Chilpancingo, Guerrero. Photographs by Ángel Almazán. (**B**,**C**) Specimen cultivated in Mexico City. Photographs by Eric Hágsater, based on the type (AMO).

**Figure 58:**
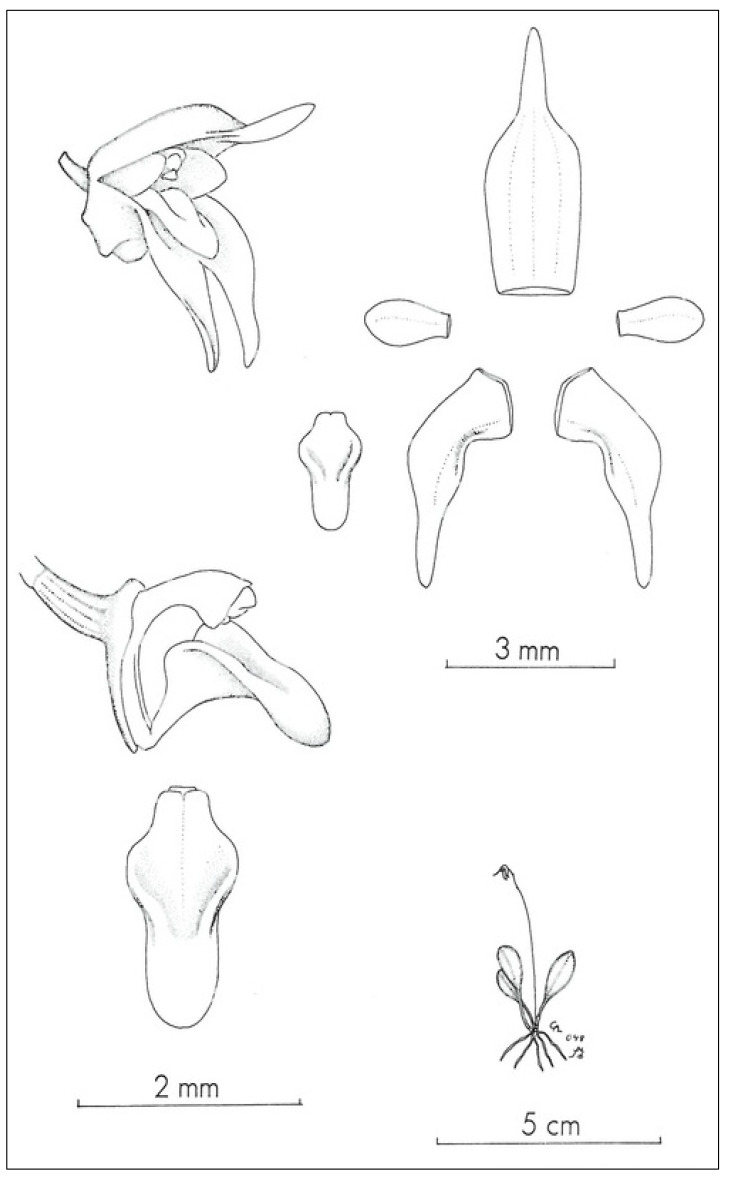
Illustration of *Specklinia hagsateri*. Drawing by Carlyle Luer, based on the type specimen. Courtesy Marie Selby Botanical Gardens.

**Figure 59:**
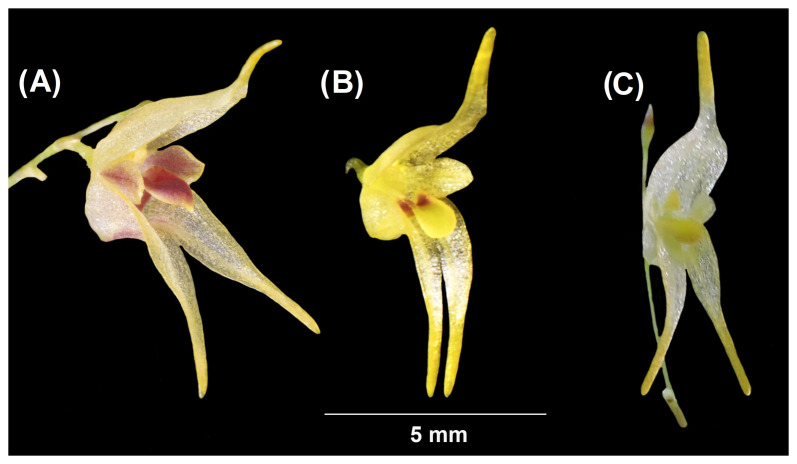
Comparison between the three Mexican species of the subgenus *Sylphia*. (**A**) *S. echinata*, based on *M.A. Soto Arenas & R. Solano 7309a* (AMO). (**B**) *S. fuegi*, based on *G. Salazar 8407* (AMO). (**C**) *S. hagsateri*, based on *A. Almazán & T. Fuentes 122* (UAGro). (**A**) Photograph by Rodolfo Solano. (**B**) Photograph by Ethian Licona. (**C**) Photograph by Ángel Almazán.

**Herb** cespitose, erect, up to 2.6 cm tall without the inflorescence. **Roots** 0.4–0.8 mm gross. **Rhizome** 1.0–1.3 mm long between adjacent stems. **Stem** 2.7–6.5 mm long, 0.3–0.4 mm gross, sheaths 1.7–4.0 mm long. **Leaf** 5.0–12.5 mm × 3.0–5.0 mm, obovate to elliptic–obovate, obtuse, succulent, flattened, glabrous, the apical mucro 0.2–0.3 mm long, light green, abruptly attenuated into a petiole 3.0–7.0 mm long, 0.3–0.7 mm gross. **Inflorescence** 2.4–6.2 cm long, flexuous, lax, glabrous; spathaceous bract 0.5–0.8 mm long; peduncle longer than the leaf, 1.4–3.9 cm long, 0.2–0.3 mm gross, with 2 bracts 0.5–0.9 mm long, glabrous, membranaceous, hyaline; rachis 1.0–2.3 cm long, with 3–8, successive, secund flowers, spaced from one another by 2.0–8.4 mm, one open at a time. **Floral bracts** 0.8–1.8 mm long, obliquely tubular-infundibuliform, obtuse, shortly apiculate, membranaceous, glabrous, hyaline suffused with purple. **Flowers** 3.4–5.0 mm × 1.6–2.8 mm; sepals and petals yellowish-white; lip greenish-yellow, the apex yellow; column and foot greenish-yellow; anther yellowish-white. **Sepals** concave, axially carinate, glabrous, the margins cellular–papillose, recurved in the apex; dorsal sepal 4.5–5.6 mm × 1.4–1.9 mm, oblanceolate to oblong–obovate, 3-veined, cauda 1.0–1.5 mm long, 0.2–0.4 mm gross; lateral sepals 3.5–5.0 mm × 0.9–1.5 mm, connate in ½ of their length, divergent towards the apex, each one obliquely oblong–lanceolate, 2-veined, gibbose in the base, caudae 1.1–1.6 mm long 0.2–0.4 mm gross. **Petals** 1.5–1.9 mm × 0.8–1.0 mm, obovate, obtuse, 1-veined. **Lip** 2.0–2.7 mm × 1.0–1.3 mm, fleshy, 3-lobulate, 3-veined, minutely papillar-glandulous; lateral lobes narrowly elliptic, erect, the margins recurved, channeled between them; mid lobe oblong to oblong–ligulate, obtuse, recurved. **Column** 0.7–1.4 mm long, 0.4–0.5 mm width, wings obliquely triangular, obtuse, entire, retrorse, 0.3–0.4 mm × 0.2–0.3 mm; clinandrium obtuse, entire; foot 1.0–1.6 mm long, 0.2–0.4 mm width, sub-terete, obtuse. **Stigmatic cavity** 0.2 mm × 0.2 mm, elliptic–orbicular; rostellum laminar, oblong. **Anther** 0.3–0.4 mm long and width, elliptic–ovoid. **Pollinia** 0.2–0.3 mm × 0.2–0.3 mm, obovoid. **Ovary** 0.5–0.8 mm long, 0.3–0.5 mm gross, obconical, recurved, verrucose–papillose, yellowish-green suffused with purple; pedicel 0.8–2.0 mm long, 0.2–0.3 mm gross, glabrous. **Capsule** not seen (Figure 57, Figure 58 and Figure 59).

**Figure 60:**
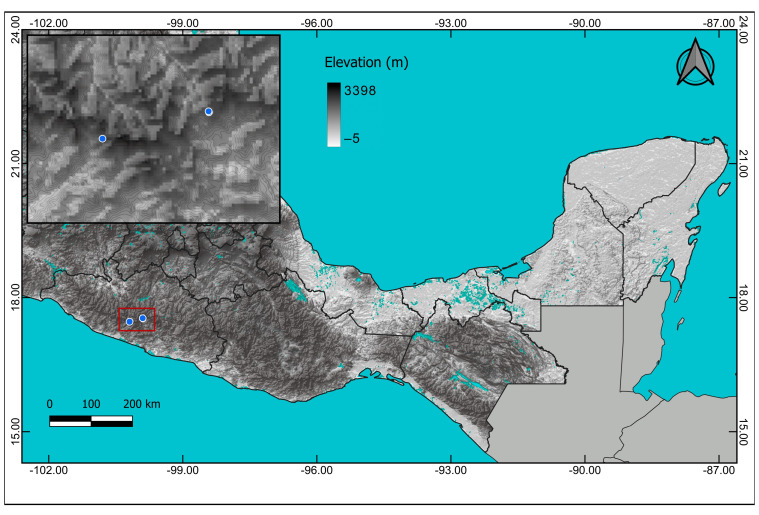
Distribution map of *Specklinia hagsateri* in Mexico (blue dots). Map by Ethian Licona.

Distribution: Endemic. Currently this species is only known from a few localities occurring in the Floristic Province of Serranías Meridionales, in the vicinity of Cerro Teotepec, in central Guerrero, a region recognized as an important center of endemism in Mexico (Figure 60).

Habitat: Epiphyte on *Quercus* sp. The species grows in the tropical mountain cloud forest, semi-deciduous tropical forest and *Pinus-Quercus* forest, at elevations between 1750 and 2010 m.

Phenology: Flowering occurs from December to August. Specimens bearing mature fruits have not yet been observed.

Conservation status: In the assessment of its conservation status following the MER criteria [78], a score of 2.08 was obtained, which suggests that this species should be considered as Endangered (P). In the evaluation under IUCN criterion B, it achieved an EOO of 21.865 km^2^ and an AOO of 12.00 km^2^, suggesting that this species should be classified as Critically Endangered (CR).

Additional commentaries: *Specklinia hagsateri* can be recognized by its obovate, obtuse leaves; flexuous, lax raceme, with a peduncle longer than the leaf; secund flowers spaced from each other, yellowish-white; dorsal sepal oblanceolate; lateral sepals connate by the half of its length, obliquely oblong–lanceolate; petals obovate, obtuse; lip 3-lobulate, greenish-yellow; lateral lobes narrowly elliptic, erect; mid lobe oblong, obtuse, recurved; foot longer than the column body; and an ovary verrucose.

*Specklinia hagsateri* has long been confused with *S. fuegi* and was previously treated as its synonym. Nevertheless, a consistent set of morphological characters and ecological preferences clearly distinguishes the two species; therefore, *S. hagsateri* is recognized as a distinct taxon. The morphological differences among the Mexican species within *Specklinia* subg. *Sylphia* are summarized in Table 4.

Examined specimens: MEXICO. Guerrero: Atoyac de Álvarez, cerca de Puerto Gallo, 01.01.2015, *A. Almazán s/n* (Photograph). Chilpancingo de los Bravo, Jaleaca de Catalán, barranca El Espantarrín, al S de Cruz de Ocote, 03.01.2015, *A. Almazán 122 & T. Fuentes* (UAGro!).

***Specklinia* subgen. *Specklinia***.

**15. *Specklinia blancoi*** (Pupulin) Soto Arenas & Solano, Icon. Orchid., 5–6: t. 669. 2003.

**Figure 61:**
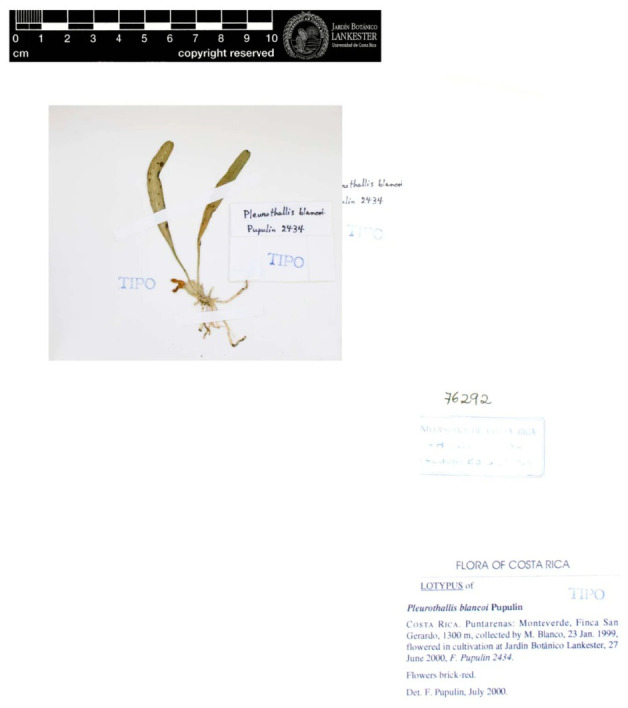
Holotype of *Pleurothallis blancoi*, *F. Pupulin 2434* (USJ-76292), courtesy of the Herbarium of the Universidad de Costa Rica.

Basionym: *Pleurothallis blancoi* Pupulin. Caesiana, 15: 1–4. 2000. TYPE: COSTA RICA: Puntarenas, Monteverde, Finca San Gerardo, 1300 m, collected on 23 January 1999 by Mario Blanco; flower in cultivation at Lankester botanical garden, 27 June 2000, *F. Pupulin 2434* (holotype: USJ-76292! (Figure 61); isotype: SEL, not seen).

Homotypic synonym: *Tribulago blancoi* (Pupulin) Luer, Monogr. Syst. Bot. Missouri Bot. Gard., 105: 230. 2006.

Etymology: Named after Mario Blanco, collector of the type specimen and researcher of Universidad de San José in Costa Rica.

**Figure 62:**
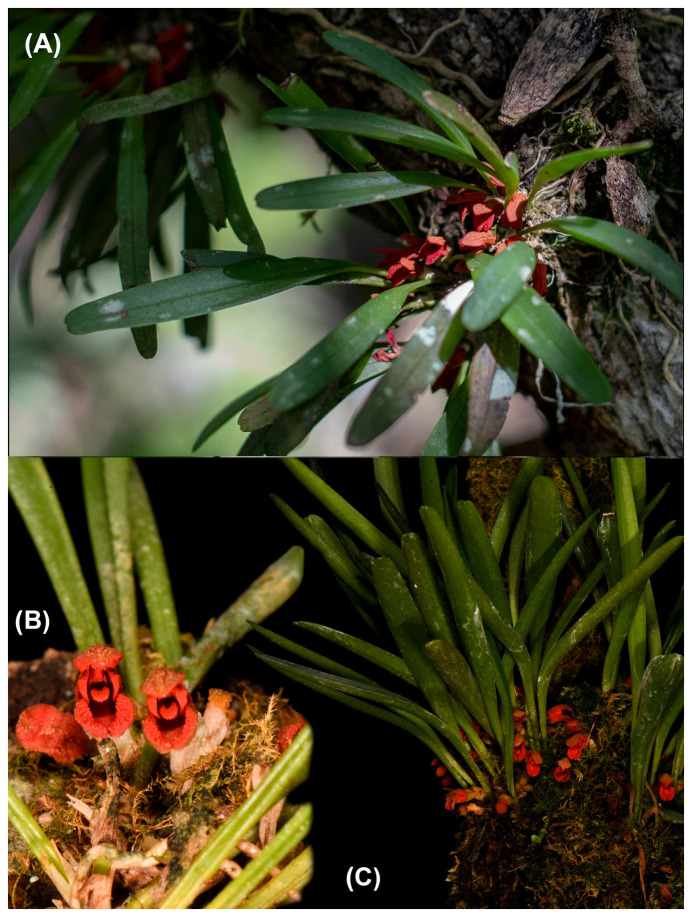
Photographs of *Specklinia blancoi*. (**A**) Specimen in its habitat, Berriozábal, Chiapas. Photograph by Jhoseline Ruiz. (**B**) Specimen cultivated in the State of Mexico, from Pichucalco, Chiapas. Photograph by Ethian Licona. (**C**) Specimen cultivated in Mexico City. Photograph by Ethian Licona, based on *M.A. Soto-Arenas 2976* (AMO).

**Figure 63:**
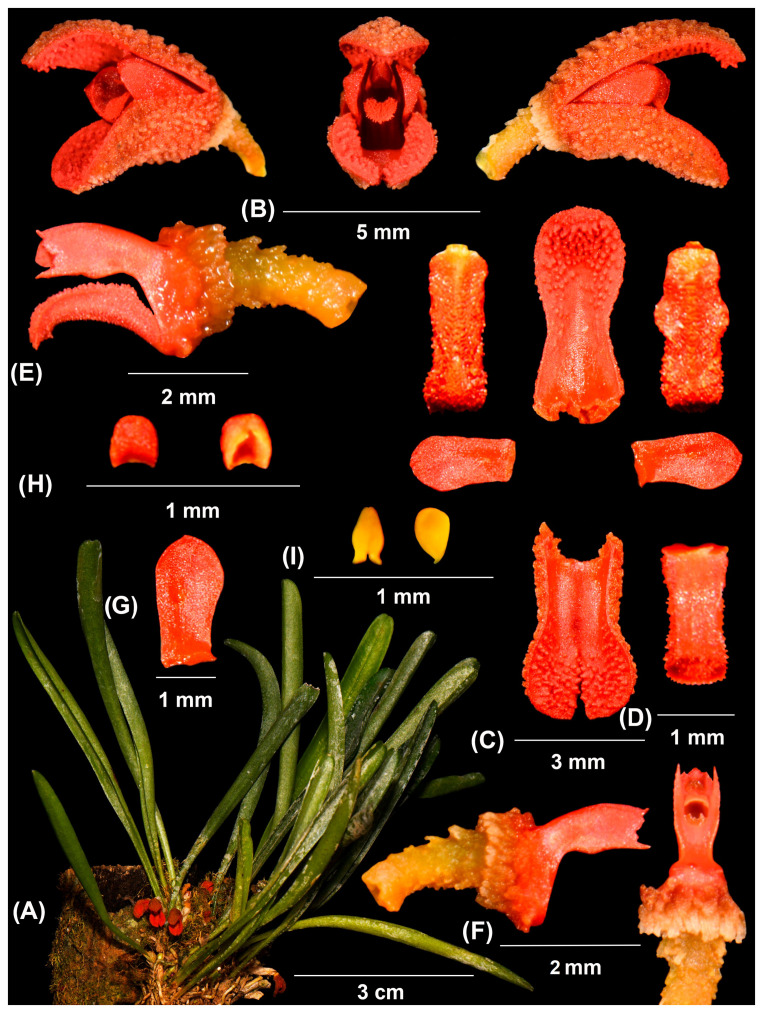
LCDP of *Specklinia blancoi*. (**A**) Habit. (**B**) Flower, ¾, frontal and lateral views. (**C**) Dissected flower. (**D**) Lip, dorsal and ventral views. (**E**) Column, lip and pedicelate ovary in lateral view. (**F**) Column, lateral and ventral views. (**G**) Petal. (**H**) Anther, dorsal and ventral views. (**I**) Pollinia, lateral and frontal views. Plate by Ethian Licona, based on *E. Licona s.n.*, from Pichucalco, Chiapas.

**Herb** cespitose, erect, up to 9.0 cm tall. **Roots** 0.4–0.8 mm gross. **Rhizome** 1.2–4.0 mm long between adjacent stems. **Stem** 3.0–14.0 mm long, 0.4–0.7 mm gross, sheaths 1.9–7.2 mm long. **Leaf** 18.0–56.0 mm × 2.0–7.5 mm, linear to narrowly linear–obovate, obtuse to truncate, coriaceous, slightly triquetrous, glabrous, the apical mucro 0.2–0.3 mm long, light green, gradually attenuated into a petiole 5.0–20.0 mm long, 0.5–0.8 mm gross. **Inflorescence** 6.0–12.5 mm long, congested, erect, glabrous, concealed by the bracts; spathaceous bract 0.7–1.0 mm long; peduncle abbreviated, 3.0–5.0 mm long, 0.4–0.5 mm gross, with 1–2 bracts 2.0–3.5 mm long, papyraceous, striated to minutely verrucose, whitish; rachis 3.0–7.5 mm long, with 2–5, successive flowers, one or two open at a time. **Floral bracts** 1.5–5.0 mm long, obliquely tubular-infundibuliform, acute to acuminate, shortly apiculate, papyraceous, striated to minutely verrucose, whitish. **Flowers** 4.0–6.8 mm × 1.3–3.0 mm; sepals, petals, lip, column, foot and anther orange. **Sepals** concave, axially carinate, densely papillose–verrucose on both surfaces; the apices free; dorsal sepal 4.0–6.8 mm × 1.3–2.6 mm, oblong–obovate, pandurate, obtuse–rounded, incurved, 3-veined; lateral sepals 3.7–6.5 mm × 1.5–3.5 mm, connate in the basal ⅔ into an oblong–obovate, obtuse, pandurate, deeply bifid synsepal, 6-veined, recurved, gibbose in the base. **Petals** 2.3–3.2 mm × 0.8–1.5 mm, obliquely oblong–obovate, obtuse, fleshy, slightly concave, minutely apiculate, 2-veined. Lip 2.2–3.0 mm × 0.8–1.0 mm, fleshy, minutely 3-lobulate, 3-veined, papillose–verrucose, marginally erose–denticulate, with a pair of verrucose submarginal calli, channeled between them; lateral lobes elliptic, erect, verrucose; mid lobe oblong–obovate, obtuse to truncate, recurved. **Column** 1.7–2.8 mm long, 0.6–1.0 mm width, wings subulate, entire, straight, 0.5–0.7 mm × 0.2–0.3 mm; clinandrium obtuse, dentate; foot 1.0–1.6 mm long, 0.3–0.6 mm width, obtuse. **Stigmatic cavity** 0.3–0.4 mm × 0.3–0.4 mm, elliptic–orbicular; rostellum laminar, oblong. **Anther** 0.6–0.9 mm long, 0.5–0.7 mm width, elliptic–ovoid, verrucose at the apex. **Pollinia** 0.3–0.4 mm × 0.2–0.3 mm, obovoid. **Ovary** 0.7–1.4 mm long, 1.1–1.6 mm gross, cylindrical, straight, echinate-verrucose, orange; pedicel 1.5–2.5 mm long, 0.6–1.0 mm gross, verrucose. **Capsule** 3.8–6.5 mm long, 2.8–3.5 mm gross, elliptic to globose, echinate–verrucose, yellowish-green (Figure 62 and Figure 63).

Distribution: Mexico, Guatemala, Honduras, Nicaragua, Costa Rica and Panama. In Mexico, the species is distributed in the states of Chiapas, Tabasco and Veracruz, probably also in Oaxaca. It occurs in the Floristic Provinces of Costa del Golfo de México, Costa Pacífica, Serranías Transístmicas, Serranías Meridionales and Valle de Tehuacán.

**Figure 64:**
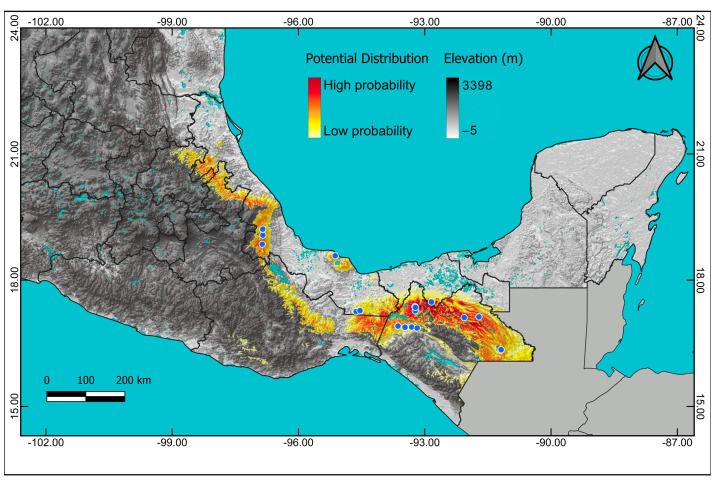
Potential distribution of *Specklinia blancoi* in Mexico and the location of currently known occurrence records (blue dots). Map by Ethian Licona.

The accepted potential distribution model for *S. blancoi* yields an AUC = 0.931, where the most influential environmental variables were the Digital Elevation Model of Mexico (27.8%), precipitation of the driest quarter (27.4%) and precipitation seasonality (15.0%). The potential distribution area was estimated at 57,180.94 km^2^, representing approximately 2.91% of Mexico’s total territory (Figure 64).

Habitat: Epiphyte on *Cordia megalantha*, *Dussia mexicana*, *Quercus* sp., *Tabebuia rosea*, *Zanthoxylum kellermanii*, and occasionally on *Mangifera indica*. The species grows in the evergreen tropical forest, semi-deciduous tropical forest, riparian vegetation and secondary vegetation derived from these forest types, at elevations between 110 and 1150 m.

Phenology: Flowering occurs from December to August. Mature fruits have been observed from February to November. The flowers emit a diurnal scent reminiscent of fermented fruit.

Conservation status: In the assessment of its conservation status following the MER criteria [78], a score of 0.64 was obtained, therefore it is not necessary to include it in any risk category. In the evaluation under IUCN criterion B, it achieved an EOO of 53,657.492 km^2^ and an AOO of 68.00 km^2^, suggesting that this species should be classified as Least Concerned (LC).

Additional commentaries: *Specklinia blancoi* can be recognized by its cespitose habit; linear, truncate, succulent leaves; congested raceme, shorter than the leaf, bearing successive, orange flowers; sepals obtuse, papillose–verrucose, free in the apices; dorsal sepal oblong–obovate; synsepal oblong–obovate, deeply bifid; petals obliquely oblong–obovate, minutely apiculate; lip minutely 3-lobulate, papillose–verrucose; lateral lobes minute, elliptic, erect; mid lobe oblong–obovate, obtuse–truncate, recurved, erose–denticulate.

This species is similar to *S. tribuloides*, from which it differs by its spiral-ascending habit (vs. cespitose); obovate, obtuse leaves (vs. linear, truncate); flowers up to 9.5 mm long (vs. up to 6.8 mm long); sepals acuminate, adherent at their apices and leaving two lateral windows (vs. obtuse, free up to their apices); dorsal sepal oblong–lanceolate (vs. oblong–obovate); synsepal oblong–oblanceolate (vs. oblong–obovate); and petals lanceolate, acuminate (vs. obovate, obtuse).

Examined specimens: MEXICO. Chiapas: Ocosingo, ca. km 33 del camino Chancalá-Monte Líbano, ca. del Rancho El Diamante, 13.16.1986, *M.A. Soto Arenas 2976* (AMO!); 2 km al SE de la colonia Benito Juárez Miramar, 21.08.1993, *A. Reyes-García 2098 & M. Sousa* (MEXU!). Ocozocoautla, Laguna Bélgica, al NE del Ocote, 30 km al NW de Ocozocoautla, 01.06.1950, *F. Miranda 6349* (MEXU!); Parque Educativo Laguna Bélgica, 16.08.2007, *G. López 820* (CHIP!); Emilio Rabasa, al N de Ocozocoautla, ca. 40 km rumbo a la Cima de las Cotorras, Selva El Ocote, 25.05.2009, *J. Borraz 32* (HEM!). Veracruz: San Andrés Tuxtla, cerro Lázaro Cárdenas, Estación Biológica Tropical Los Tuxtlas, 26.08.1986, *S. Sinaca 887* (MEXU!). Totutla, Zacuapan, 01.01.1917, *C. Purpus 7835* (GH!, UC!); Zacuapan, 01.01.1917, *C. Purpus s/n* (NYBG!). Uxpanapa, 800 mts del campamento Hnos. Cedillo hacia la escuadra, 26.07.1974, *P. Valdivia 1128* (HUAP!, XAL!); 6 km del campamento Hnos. Cedillo hacia la laguna, 12.08.1974, *P. Valdivia 1442* (XAL!).

Additional records: MEXICO. Chiapas: Berriozábal, 1.6 km al NW de Efraín A. Gutiérrez, al SE de la cueva El Amate, 10.07.2022, *J. Ruíz s/n* (https://www.inaturalist.org/observations/127173134. Accessed 25 Abril 2025). Chilón, Ramosil, 14.01.2014, *M. Hernández s/n* (Photograph). Francisco León, 2.4 km al SW de Vicente Guerrero, 01.07.2021, *D. Molina s/n* (Photograph). Ocozocoautla, Parque Educativo Laguna Bélgica, 31.07.2024, *C. Rodríguez s/n* (https://www.inaturalist.org/observations/232989556. Accessed 25 Abril 2025). Pichucalco, en la cima del Volcán Chichonal, 26.06.2021, *Fridalí s/n* (https://www.inaturalist.org/observations/191814943. Accessed 25 Abril 2025); camino al Volcán Chichonal, 01.01.2022, *M. González s/n* (Photograph). San Fernando, al SE de San Fernando, 01.06.2020, *O. Pérez-Cota s/n* (Photograph). Tabasco: Teapa, cerca de la frontera entre Chiapas y Tabasco, 15.02.2022, *M. González s/n* (Photograph). Veracruz: Amatlán de los Reyes, Manuel León, Colegio de Postgraduados, Campus Córdoba, 26.12.2020, *V. de la Cruz s/n* (https://www.inaturalist.org/observations/67123426. Accessed 25 Abril 2025). Tepetlaxco, Tepetlaxco, 21.04.2023, *V. de la Cruz s/n* (https://www.inaturalist.org/observations/156040569. Accessed 25 Abril 2025).

**16. *Specklinia corniculata*** (Sw.) Mutel, Mém. Soc. Roy. Centr. Agric. Dépt. N. 1835–1836: 84, 1837.

**Figure 65:**
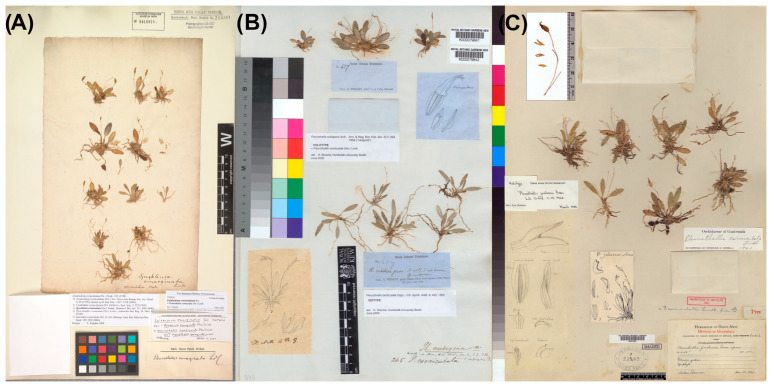
(**A**) Lectotype of *Epidendrum corniculatum*, *O. Swartz s/n* (W-16978), courtesy of the Herbarium of Naturhistorisches Museum Wien. (**B**) Holotype of *Pleurothallis nubigena*, *C. Wright 657* (K-79841, http://specimens.kew.org/herbarium/K000079841. Accessed 26 Abril 2025), from © The Board of Trustees-RBG, Kew. (**C**) Holotype of *Pleurothallis jocolensis*, *H. Johnson 1048* (AMES-74364), courtesy of The Orchid Herbarium of Oakes Ames of Harvard University.

Basionym: *Epidendrum corniculatum* Sw., Prodr. 123. 1788. TYPE: JAMAICA. Unknown locality, “*Incolit truncos vetustos arborum Jamaicae interioris*”, *O. Swartz s/n* (lectotype (designated by Kolanowska [90]): W-16978! (Figure 65A); isolectotypes: BM, not seen; LD-1732006!; S-R-1935!; UPS, not seen).

Homotypic synonyms: *Dendrobium corniculatum* (Sw.) Sw., Nova Acta Regiae Soc. Sci. Upsal. vi. 83. 1799; *Cymbidium corniculatum* (Sw.) Spreng., Syst. Veg., ed. 16 [Sprengel] 3: 722. 1826; *Specklinia emarginata* Lindl., Gen. Spec. Orchid. Pl., 8–9. 1830; *Specklinia corniculata* (Sw.) Steud., Nomencl. Bot., (ed. 2) 1: 489. 1840, *nom. superfl*.; *Pleurothallis corniculata* (Sw.) Lindl., Edwards’s Bot. Reg., 28: Misc. 83. 1842; *Pleurothallis emarginata* (Lindl.) Lindl., Folia Orchidacea, Pleurothallis. 9: 25. 1859, *nom. illeg*., non Lindl. 1830 (*=Stelis emarginata* (Lindl.) Soto Arenas & Solano); *Humboltia corniculata* (Sw.) Kuntze, Revis. Gen. Pl. 2: 667. 1891; *Sarcinula corniculata* (Sw.) Luer, Monogr. Syst. Bot. Missouri Bot. Gard., 105: 209–2011. 2006.

Etymology: From the Latin *corniculatum*, “with a little horn”, not explained in the protologue but probably referring to the column wings or the uniflorous inflorescences; and from the Latin *emarginatus*, “emarginate or without margin”, probably referring to the emarginate apex of the leaf or the synsepal.

Heterotypic synonyms: *Pleurothallis nubigena* Lindl., Ann. Mag. Nat. Hist., ser. 3. 1 (5): 326. 1858. TYPE: CUBA. [Guantánamo] in Cuba Orientali, July 1856, *C. Wright 657* (holotype: K-79841! (Figure 65B); isotypes: AMES-104098!, BR-6585716!).

Etymology: From the Latin *nubigena*, “born from a cloud”, referring to its habitat in an always cloudy environment.

*Pleurothallis jocolensis* Ames, Sched. Orch., 2: 19–21. 1923. TYPE: GUATEMALA. Izabal: Jocoló, Río Perdonalis, 25 December 1920, *H. Johnson 1048* (holotype: AMES-74364! (Figure 65C)).

Etymology: Named after Jocoló, a place in Izabal, Guatemala, where the type was collected.

**Figure 66:**
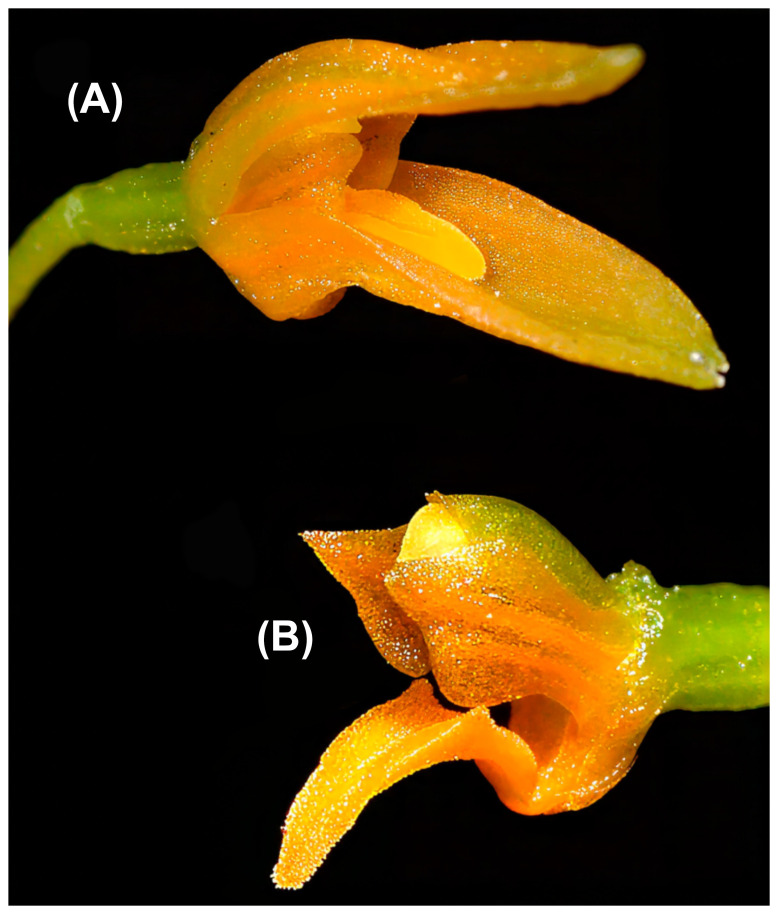
*Specklinia corniculata*, specimen cultivated in Alta Verapaz, Guatemala; (**A**) Flower in ¾ view. (**B**) Column, lip, petals and ovary in ¾ view. Photographs by José Monzón & Edgar Mó.

**Figure 67:**
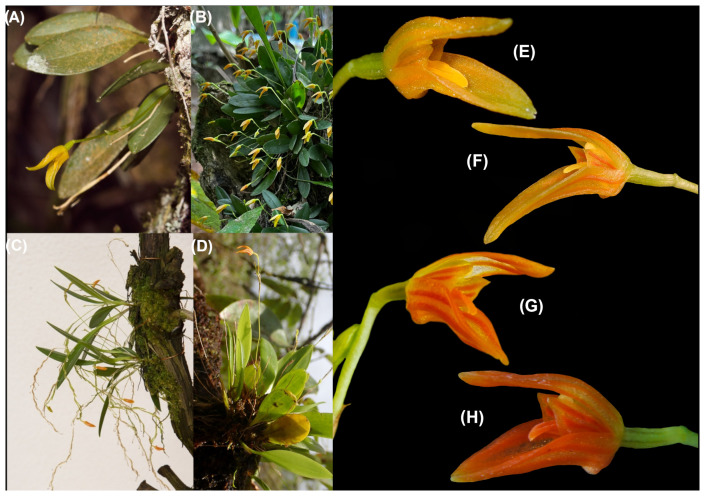
Comparison among *Specklinia corniculata* and morphologically similar species. (**A**) Habit of *S. corniculata* from Alajuela, Costa Rica. Photograph by Jessie Aguilar. (**B**) Habit of *S. barboselloides* from Puntarenas, Costa Rica. Photograph by Spicyhands (iNaturalist). (**C**) Habit of *S. displosa* from Panama. Anonymous photographer. (**D**) Habit of *S. striata* from Roura, French Guiana. Photograph by Elendil Cocchi. (**E**) Flower of *S. corniculata* from Izabal, Guatemala. Photograph by José Monzón & Edgar Mó. (**F**) Flower of *S. barboselloides* from Puntarenas, Costa Rica. Photograph by Ricardo Marín. (**G**) Flower of *S. displosa* from Panama. Anonymous photographer. (**H**) Flower of *S. striata* from Suriname. Photograph by Wiel Driessen.

**Figure 68:**
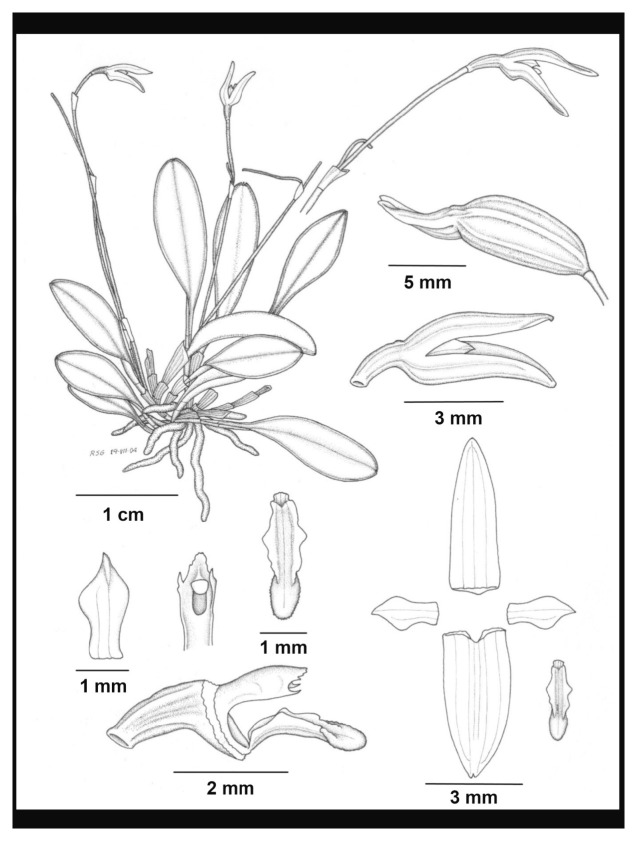
Illustration of *Specklinia corniculata*. Drawing by Rodolfo Solano, based on *E. Martínez et al. 25035* (MEXU).

**Herb** cespitose, erect, up to 4.5 cm tall without the inflorescence. **Roots** 0.5–0.8 mm gross. **Rhizome** 0.6–1.4 mm long between adjacent stems. **Stem** 2.5–10.0 mm long, 0.3–0.5 mm gross, sheaths 3.0–7.0 mm long. **Leaf** 10.0–25.0 mm × 3.0–9.0 mm, elliptic–lanceolate to elliptic–oblanceolate, obtuse, coriaceous, flattened, marginate, glabrous, the apical mucro 0.4–0.6 mm long, light green, abruptly attenuated into a petiole 2.5–10.0 mm long, 0.4–0.9 mm gross. **Inflorescence** 2.6–5.3 cm long, congested, glabrous; spathaceous bract 0.6–1.0 mm long; peduncle as long as the leaf, 1.8–3.5 cm long, 0.2–0.3 mm gross, with 1–2 bracts 0.7–1.5 mm long, membranaceous, glabrous, hyaline; rachis 0.8–1.8 cm long, with 2 flowers, but only one functional. **Floral bracts** 1.3–4.0 mm long, obliquely tubular-infundibuliform, obtuse, shortly apiculate, membranaceous, glabrous, hyaline. **Flowers** 4.6–6.0 mm × 1.8–2.4 mm; usually cleistogamous; sepals, petals and lip bright yellow to greenish-yellow; column greenish with a foot yellow; anther yellowish-white. **Sepals** concave, axially carinate, glabrous, marginally cellular–papillose; dorsal sepal 4.5–6.0 mm × 1.5–2.3 mm, oblong–lanceolate to lanceolate, obtuse, 3-veined; lateral sepals 4.2–5.7 mm × 3.0–3.5 mm, completely connate into an elliptic–lanceolate to elliptic–ovate, obtuse, emarginate synsepal, 4-veined, gibbose in the base. **Petals** 1.6–2.3 mm × 0.8–1.2 mm, oblong–subrhombic, acute and distally acuminate, slightly thickened at the apex, 2-veined, the base attenuated. **Lip** 2.2–2.5 mm × 0.7–1.2 mm, fleshy, 3-lobulate, 3-veined, minutely papillar-glandulous; lateral lobes narrowly triangular, erect, marginally erose, with a pair of papillose submarginal calli, channeled between them; mid lobe elliptic to oblong–elliptic, rounded, marginally erose–denticulate. **Column** 1.6–2.4 mm long, 0.5–0.9 mm width, wings obliquely triangular to subulate, entire, 1.5–1.7 mm × 0.2–0.3 mm; clinandrium obtuse, dentate; foot 1.0–1.9 mm long, 0.4–0.9 mm width, obtuse. **Stigmatic cavity** 0.2 mm × 0.3 mm, elliptic–oblong; rostellum laminar, oblong. **Anther** 0.4–0.5 mm long, 0.3–0.4 mm width, elliptic–ovoid. **Pollinia** 0.3–0.4 mm × 0.2–0.3 mm, obovoid. **Ovary** 1.7–4.0 mm long, 0.6–0.9 mm gross, obconical, recurved, glabrous, green; pedicel 7.0–13.0 mm long, 0.3–0.4 mm gross, glabrous. **Capsule** 4.0–9.5 mm long, 2.2–3.6 mm gross, elliptic–oblong to elliptic–obovoid, yellowish-green (Figure 66, Figure 67 and Figure 68).

Distribution: Mexico, Guatemala, Belize, Honduras, Nicaragua, Costa Rica, Cuba, Jamaica and Haiti. In Mexico, the species is distributed only in the state of Chiapas, within the Floristic Province of Costa del Golfo de México.

**Figure 69:**
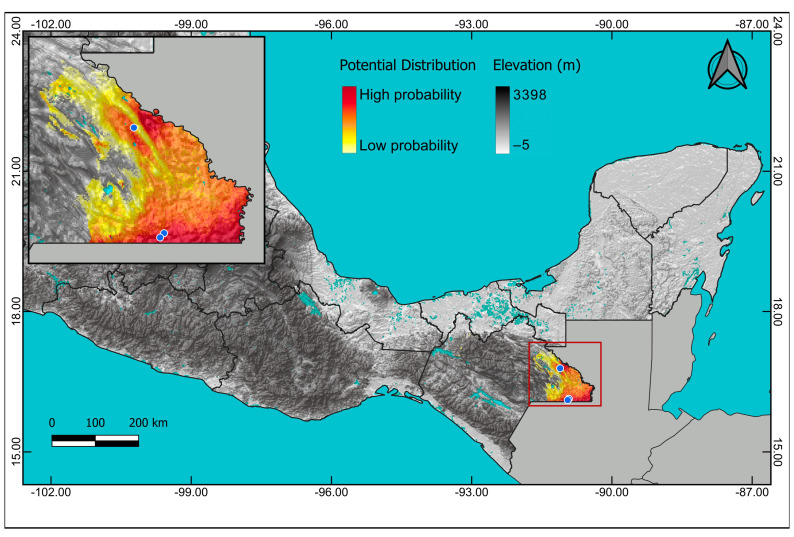
Potential distribution of *Specklinia corniculata* in Mexico and the location of currently known occurrence records (blue dots). Map by Ethian Licona.

Due to the limited number of records for this species in Mexico, reviewed specimens from Guatemala were incorporated into the modeling of its potential distribution. The resulting model was subsequently clipped to include only the Mexican distribution. The accepted potential distribution model for *S. corniculata* yields an AUC = 0.940, where the most influential environmental variables were isothermality (36.4%), temperature seasonality (32.6%) and precipitation of the wettest quarter (30.9%). The potential distribution area was estimated at 7607.09 km^2^, representing approximately 0.39% of Mexico’s total territory (Figure 69).

Habitat: Epiphyte on *Brosimum alicastrum*, *B. costaricanum*, *Crescentia* sp. and *Rhizophora mangle*. The species grows in the evergreen tropical forest, semi-deciduous tropical forest and riparian vegetation, at elevations between 100 and 450 m.

Phenology: Flowering occurs from September to July. Mature fruits have been observed from November to August. Most examined specimens bore developing capsules in the most flowers, suggesting possible cleistogamy.

Conservation status: In the assessment of its conservation status following the MER criteria [78], a score of 2.39 was obtained, which suggests that this species should be considered as Endangered (P). In the evaluation under IUCN criterion B, it achieved an EOO of 3590.523 km^2^ and an AOO of 16.00 km^2^, suggesting that this species should be classified as Endangered (EN).

Additional commentaries: *Specklinia corniculata* can be recognized by its elliptic–lanceolate, obtuse, marginate leaves; erect raceme reduced to one functional flower, with a peduncle as long as the leaf; flower bright yellow to greenish-yellow; dorsal sepal oblong–lanceolate; synsepal elliptic–lanceolate; petals oblong–subrhombic, acute and distally acuminate; lip 3-lobulate, lateral lobes narrowly triangular, erect, mid lobe elliptic, rounded, erose–denticulate.

This species belongs to a poorly studied complex that also includes *Specklinia barboselloides* (Schltr.) Pridgeon & M.W.Chase, *S. displosa* (Luer) Pridgeon & M.W.Chase and *S. striata* (H.Focke) Luer, distributed mainly in Central America and northern South America. Although *S. barboselloides* has previously been placed in synonymy with *S. corniculata*, a consistent set of morphological traits as well as ecological and distributional preferences, support their separation as distinct species. The morphological differences among this species complex are summarized in Table 5.

Examined specimens: MEXICO. Chiapas: Ocosingo, Estación Chajul, 20.07.1992, *E. Martínez 25035, C.H. Ramos, R. Lombera & R. Guerrero* (AMO!, CHAPA!, MEXU!); Estación Chajul, 08.09.1992, *E. Martínez 25275, C.H. Ramos & R. Lombera* (MEXU!); 1.16 km al SE del crucero de San Javier, 29.07.2003, *G. Aguilar 7568, A. Chambor & C. Chancayun* (MEXU!).

17. ***Specklinia juddii*** (Archila) Pupulin & Karremans, Die Orchidee (Hamburg), 64 (6): 480. 2013.

**Figure 70:**
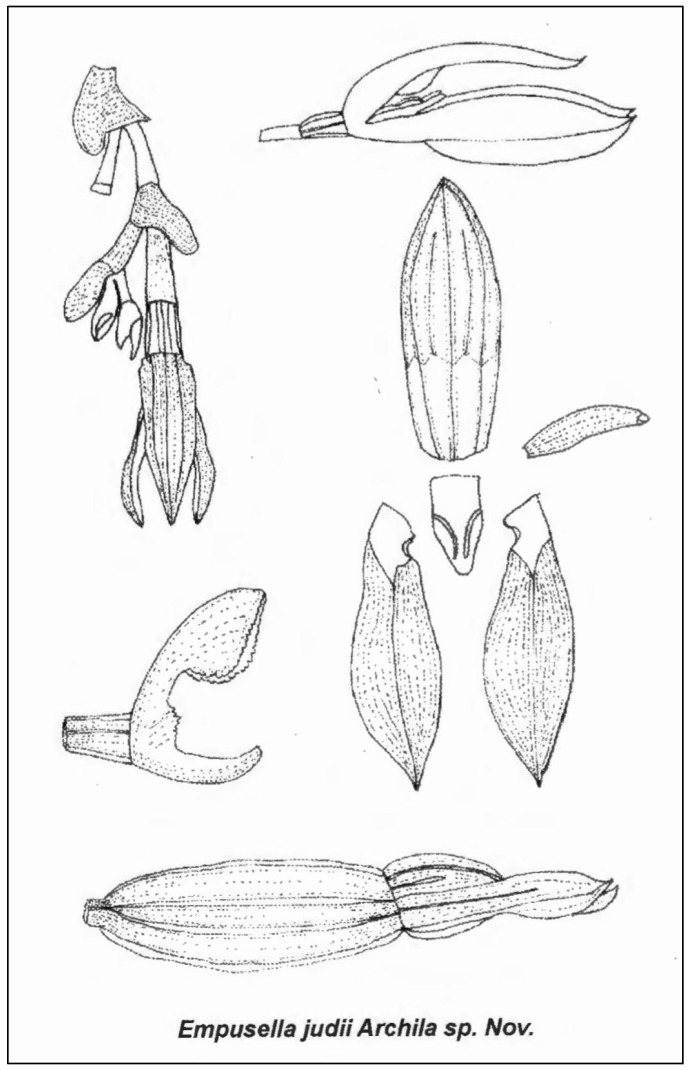
Illustration based on the type of *Empusella juddii*. Drawing by Fredy Archila, based on *F. Archila s/n* (from Archila [91]).

Basionym: *Empusella juddii* Archila, Revista Guatemalensis, 15 (1): 99. 2012. TYPE: GUATEMALA. Alta Verapaz: Cerrit Quiché, 600 m elev., sobre arbustos, April 2000, *F. Archila s/n* (holotype: BIGU, not seen. Drawing based on the type in Figure 70).

Etymology: Named after Walter Judd, American botanist, curator of the vascular plant collection in the FLAS Herbarium.

**Figure 71:**
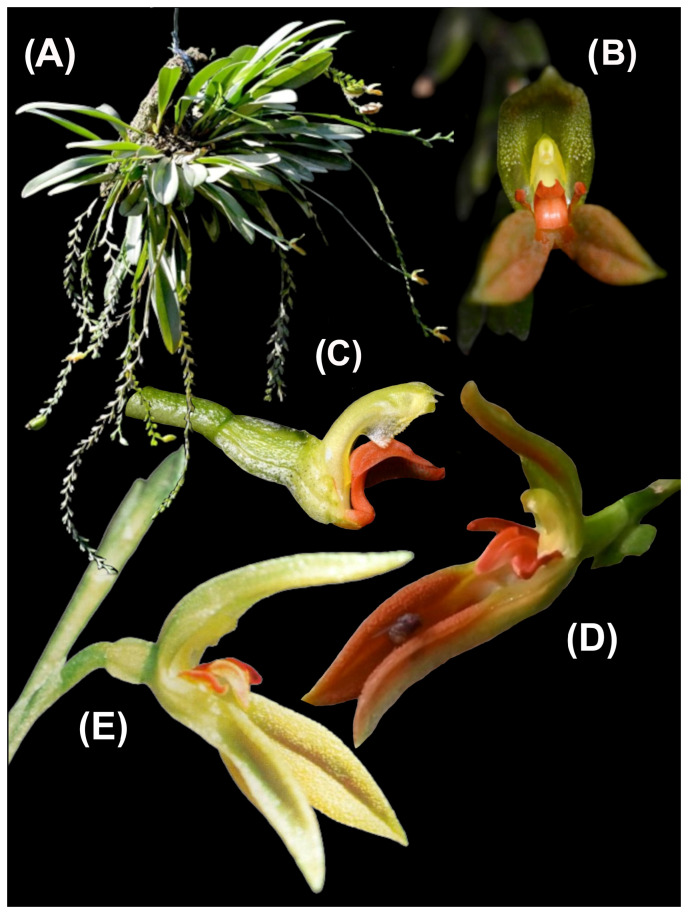
Photographs of *Specklinia juddii*. (**A**) Habit. (**B**) Flower in frontal view. (**C**) Column, petal, lip and pedicelate ovary in lateral view. Photographs by José Monzón and Edgar Mó from a specimen cultivated in Alta Verapaz, Guatemala. (**D**) Flower from a specimen cultivated in Tuxtla Gutiérrez, Chiapas. Photograph by Carlos R. Beutelspacher. (**E**) Flower from a specimen from Villa Corzo, Chiapas. Photograph by Teresa Cabrera.

**Figure 72:**
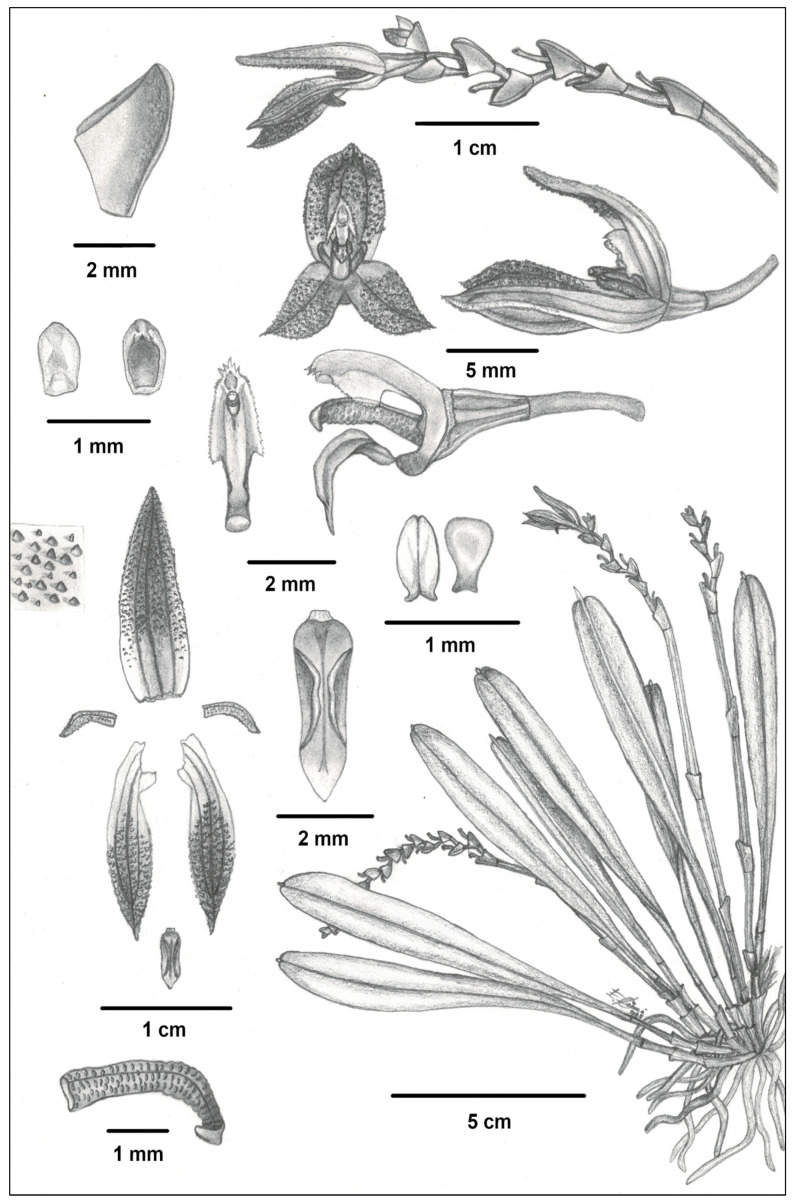
Illustration of *Specklinia juddii*. Drawing by Ethian Licona based on *A. Reyes-García 7116 et al.* (MEXU).

Herb cespitose, erect, up to 30.0 cm tall without the inflorescence. Roots 0.7–1.2 mm gross. Rhizome 2.5–7.0 mm long between adjacent stems. Stem 0.7–4.5 cm long, 1.2–2.0 mm gross, sheaths 0.4–2.1 cm long. Leaf 4.5–17.7 cm × 0.7–2.1 cm, membranous, flattened, narrowly oblanceolate, obtuse, glabrous, the margins slightly recurved, the apical mucro 0.5–1.0 mm long, light green, gradually attenuated into a petiole 1.0–7.0 cm long, 1.2–3.1 mm gross. Inflorescence 7.7–41.0 cm long, ancipitous, congested, flexuous, glabrous; spathaceous bract 2.0–4.0 mm long; peduncle as long or slightly longer than the leaf, 4.9–27.5 cm long, 2.0–2.6 mm width, with 1–2 bracts 2.0–4.5 mm long, 2.0–2.5 mm width, obliquely infundibuliform, obtuse, ancipitous, fleshy, glabrous, light green; rachis 2.8–13.5 cm long, with equidistant internodes, distant by 3.0–8.0 mm, the first internode longer, with 6–40, successive, secund flowers, one open at a time. Floral bracts 4.0–8.0 mm long, 3.0–6.5 mm width, obliquely infundibuliform, obtuse, ancipitous, fleshy, glabrous, sometimes slightly imbricate, light green. Flowers 11.5–21.3 mm × 10.0–12.5 mm; sepals adaxially orange to yellowish-orange, abaxially greenish-yellow; petals and lip reddish-orange; column yellow to greenish-yellow, foot orange; anther yellow. Sepals concave, axially keeled, adaxially verrucose, abaxially glabrous, fleshy, 3-veined; dorsal sepal 10.0–20.0 mm × 3.6–6.0 mm, elliptic–lanceolate to lanceolate, acute; lateral sepals 11.5–21.3 mm × 2.0–4.0 mm, shortly connate near the base, each one narrowly lanceolate to elliptic–lanceolate, acute, straight, sub-parallel and vertically oriented, with the adaxial surface facing each other. Petals 3.7–6.0 mm × 0.8–1.5 mm, linear–lanceolate, falcate, acute, concave, cucullate in the apex, adaxially verrucose–striated, abaxially glabrous, 2-veined. Lip 4.5–5.5 mm × 1.5–2.0 mm, fleshy, arched, oblong–lanceolate, acute, 3-veined, minutely papillar-glandulous, the margins recurved, with a pair of submarginal calli, channeled between them. Column 4.0–5.5 mm long, 1.8–3.0 mm width, wings widely trapezoidal, obtuse, dentate, 2.0–2.5 mm × 0.8–1.0 mm; clinandrium obtuse, dentate; foot 2.0–3.0 mm long, 1.0–2.0 mm width, obtuse, with a pair of pyramidal, papillose calli at the base. Stigmatic cavity 1.0 mm × 1.3 mm, elliptic–oblong; rostellum laminar, oblong. Anther 1.0–1.5 mm long, 1.5–2.0 mm width, elliptic–ovoid. Pollinia 0.6–0.8 mm × 0.4–0.6 mm, obovoid. Ovary 2.8–4.5 mm long, 0.8–1.5 mm gross, obpyramidal, triquetrous, straight, glabrous, yellowish-green; pedicel 3.0–11.0 mm long, 0.6–1.2 mm gross, glabrous. Capsule 1.8–2.0 cm long, 0.6–1.3 cm gross, elliptic–oblong to elliptic–obovoid, yellowish-green (Figure 71 and Figure 72).

Distribution: Mexico, Guatemala, El Salvador and Honduras. In Mexico, the species is confirmed only from the state of Chiapas and is likely to occur also in Oaxaca. It occurs in the Floristic Provinces of Costa Pacífica, Serranías Transístmicas and El Soconusco.

**Figure 73:**
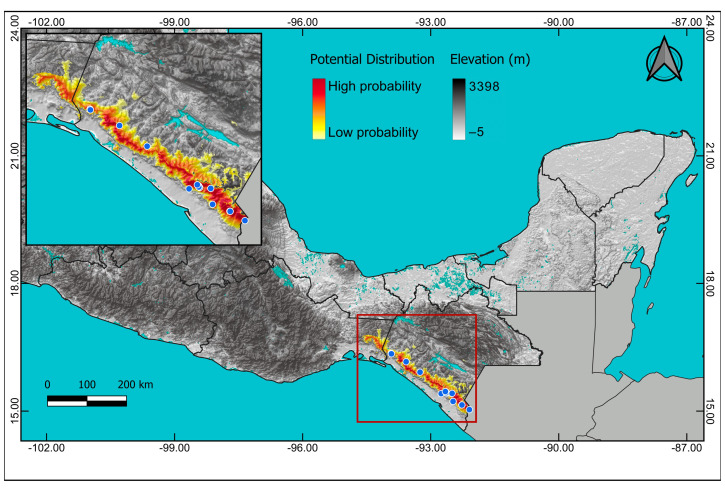
Potential distribution of *Specklinia juddii* in Mexico and the location of currently known occurrence records (blue dots). Map by Ethian Licona.

The accepted potential distribution model for *S. juddii* yields an AUC = 0.948, where the most influential environmental variables were temperature annual range (32.2%), max temperature of the warmest month (31.7%) and the Digital Elevation Model of Mexico (12.0%). The potential distribution area was estimated at 9197.76 km^2^, representing approximately 0.47% of Mexico’s total territory (Figure 73).

Habitat: Epiphyte on *Quercus* sp. The species grows in the evergreen tropical forest, semi-deciduous tropical forest and tropical mountain cloud forest, at elevations between 640 and 1850 m.

Phenology: Flowering occurs from March to November. Mature fruits have been observed from May to January. The flowers emit a diurnal scent reminiscent of fermented fruit.

Conservation status: In the assessment of its conservation status following the MER criteria [78], a score of 1.52 was obtained, which suggests that this species should be considered as a species Under Special Protection (Pr). In the evaluation under IUCN criterion B, it achieved an EOO of 2517.395 km^2^ and an AOO of 52.00 km^2^, suggesting that this species should be considered as Vulnerable (VU).

Additional commentaries: *Specklinia juddii* can be recognized by its cespitose habit; plants up to 30.0 cm in height without the inflorescence; narrowly oblanceolate, obtuse leaves; inflorescence up to 41.0 cm long, flexuous, congested, ancipitous; peduncle almost as long as the leaf, floral internodes evenly spaced along the rachis; floral bracts ancipitous; flowers up to 40, adaxially verrucose, yellowish-orange, abaxially glabrous, greenish-yellow; lateral sepals lanceolate, sub-parallel and vertically oriented, with their adaxial surfaces facing each other; petals linear–lanceolate, falcate, apically cucullate; lip oblong–lanceolate, acute, arched.

Historically, *S. juddii* and other morphologically similar species recorded in Mexico were included under *S. endotrachys* [60] or *S. spectabilis* [92], both of which were regarded as the sole representative of this complex in the country.

This species belongs to a recently recognized species complex composed by *Specklinia dunstervillei* Karremans, Pupulin & Gravend., *S. endotrachys*, *S. juddii*, *S. pfavii* (Rchb.f.) Pupulin & Karremans, *S. remotiflora* Pupulin & Karremans and *S. spectabilis*. In the present study, an additional species, *S. wendtii*, is proposed, increasing the number of taxa within this complex to seven. Of these, five are distributed in southern Central America, whereas three are distributed in the Mesoamerican region. The morphological characters useful to distinguishing the northern Mesoamerican species are summarized in Table 6.

There is a set of three specimens in the AMES herbarium bearing the collection record *O. Nagel sub Östlund 4462*. These specimens were prepared in successive years and were apparently derived from plants cultivated by Östlund in Cuernavaca, Morelos. Two of the specimens list the locality as “above Hacienda Santa María de los Arcos, East of Comitán”, whereas the third indicates “Pacific slopes of the Volcano Tacaná, near Finca El Perú, road to La Gloria”. Because no comparable specimens have been recorded from the Central Plateau of Chiapas, the locality in the Tacaná region is here considered the correct provenance for this collection number, which is referrable to *Specklinia juddii*.

Examined specimens: MEXICO. Chiapas: Acacoyagua, monte Madre Vieja, 01.06.1938, *E. Matuda 2533* (AMES!, MEXU!, MICH!); Filo del Panteón a Monte Ovando, El Triunfo, 03.05.1997, *J. Castillo 1334 & F. Bolóm* (AMO!, ECOSUR-CH!); camino ejido Las Golondrinas-Tres Cruces, 23.07.2005, *N. Martínez-Meléndez 975* (HEM!); Las Golondrinas, loma de cerro Polo, 16.10.2010, *C. Pérez 1384* (HEM!); Monte Ovando, por la vereda en el camino Las Golondrinas-Rosario Zacatonal, 14.11.2011, *J. Martínez 1563* (HEM!). Escuintla, San Juan Panamá, 23.07.1948, *E. Matuda 18152* (AMES!). Huixtla, Colonia Morelos, 07.02.1999, *A. Damon s/n* (ECO-TA-H!). Jiquipilas, cerro La Palmita, 9 km al SW de Tierra y Libertad, 06.06.2002, *O. Farrera 3261* (CHIP!); ejido Tiltepec, Rancho El Recuerdo, 06.06.2002, *A. Reyes-García 5032, C. Chavarría & E. Meléndez* (MEXU!). Tapachula, above Finca El Perú, 01.01.1935, *O. Nagel sub Oestlund 4404* (AMES!); Pacific slopes of the Volcano Tacaná, near Finca El Perú, road to La Gloria, 13.02.1935, *O. Nagel sub Oestlund 4462* (AMES!); Santa Rosalía, 03.05.2008, *A. Damon s/n, R. Solano & L. Jiménez* (ECO-TA-H!). Villa Corzo, Nueva Reforma Agraria, 26.10.2002, *J. Martínez 121* (CHIP!, HEM!, MEXU!, MO!, XAL!); ejido Sierra Morena, 12.07.2004, *A. Reyes-García 7079, D. Gómez & E. Figueroa* (MEXU!); ejido Sierra Morena, 12.07.2004, *A. Reyes-García 7116, D. Gómez & E. Figueroa* (MEXU!).

Additional records: MEXICO. Chiapas: Unión Juárez, Orquideario Santo Domingo, undated, *A. Damon s/n* (Photograph).

18. ***Specklinia lanceola*** (Sw.) Lindl., Gen. Sp. Orchid. Pl. 8. 1830.

**Figure 74:**
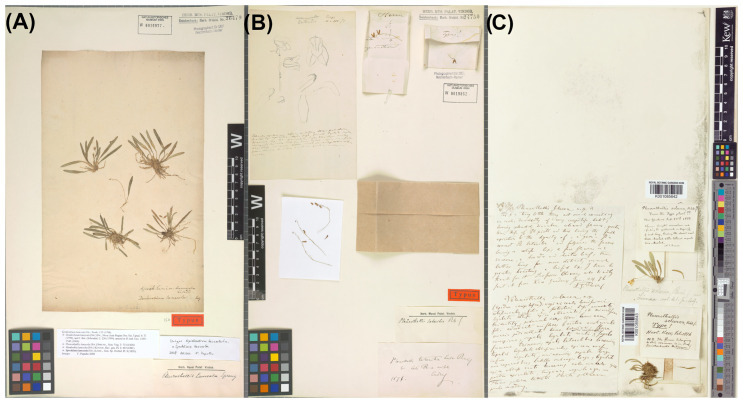
(**A**) Specimen selected as isolectotype for *Epidendrum lanceola*, *O. Swartz s/n* (W-16977), courtesy of the Herbarium of Naturhistorisches Museum Wien. (**B**) Specimen selected as lectotype for *Pleurothallis lateritia*, *A. Éndres s/n* (W-19852), courtesy of the Herbarium of Naturhistorisches Museum Wien. (**C**) Holotype of *Pleurothallis sclarea*, *Anon. s/n* (K-1085642, http://specimens.kew.org/herbarium/K001085643), from © The Board of Trustees-RBG, Kew.

Basionym: *Epidendrum lanceola* Sw., Prodr., 123. 1788. TYPE: JAMAICA. Unknown locality “*Parasiticum arborum altissimis montibus Jamaicae australis*”, *O. Swartz s/n* (lectotype here designated: S-R-1946!; isolectotypes: S-R-1947!, BM-82291!, LD-1731494!, W-16976!, W-16977! (Figure 74A)).

Homotypic synonyms: *Dendrobium lanceola* (Sw.) Sw., Nova Acta Regiae Soc. Sci. Upsal., ser. 2, 6: 83. 1799; *Cymbidium lanceolum* (Sw.) Sw., Neues J. Bot., 1: 94. 1805; *Pleurothallis lanceola* (Sw.) Spreng., Syst. Veg., ed. 16. 3: 731. 1826; *Humboltia lanceola* (Sw.) Kuntze, Revis. Gen. Pl. 2: 667. 1891; *Specklinia lanceola* (Sw.) Pridgeon & M.W.Chase, Lindleyana, 16 (4): 258. 2001. *nom. superfl.*

Etymology: From the Latin *lanceolus*, “small lance”, referring to the shape of its leaves.

Heterotypic synonyms: *Pleurothallis lateritia* Endrés ex Rchb.f., Gard. Chron., 731. 1872; *Humboltia lateritia* (Endrés ex Rchb.f.) Kuntze, Revis. Gen. Pl. 2: 667. 1891; *Specklinia lateritia* (Endrés ex Rchb.f.) Pridgeon & M.W.Chase, Lindleyana, 16 (4): 258. 2001. Type: COSTA RICA. Unknown locality “*It was sent from Costa Rica to the Hamburg Botanic Garden by Mr. Endres*”, *A. Éndres s/n* (lectotype here designated: W-19852! (Figure 74B); isolectotypes: AMES-74390 [fragments]!, AMES-74391 [Drawing based on the type]!, K-463181 [inflorescences]!).

Etymology: From the Latin *lateritius*, “brick red”, referring to the color of the flowers.

*Pleurothallis sclarea* Rchb.f., Linnaea, 41: 49. 1877 [1876]; *Specklinia sclarea* (Rchb.f.) Pridgeon & M.W.Chase, Lindleyana, 16 (4): 259. 2001. Type: Unknown locality “*culto in hortus Kewensis*”, 29 Oct 1876, *Anon. s/n* (holotype: K-1085642! (Figure 74C); isotype: K-1085643!).

Etymology: The meaning is not explained in the protologue, probably an incorrect transcription of the Latin *scarlea*, “scarlet”, referring to the color of the flowers.

**Figure 75:**
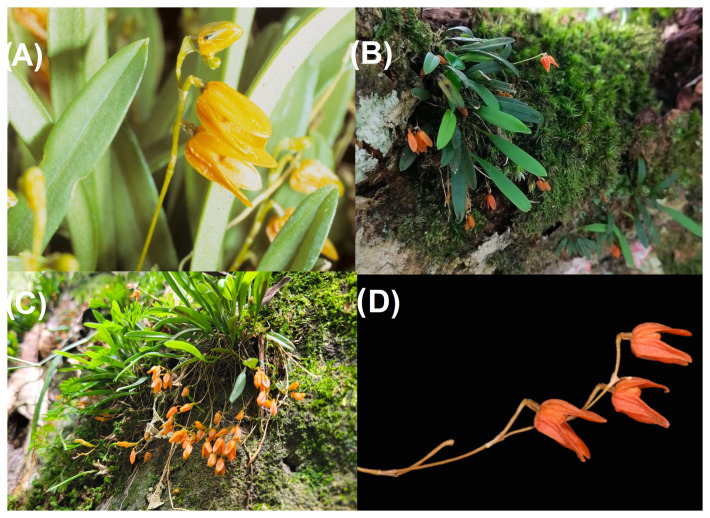
Photographs of *Specklinia lanceola*. (**A**) Specimen cultivated in Mexico city. Photograph by Rodolfo Solano, based on *M.A. Soto Arenas et al., 7905* (AMO). (**B**) Specimen in its habitat, Xico, Veracruz. Photograph by Jairo Portugal. (**C**) Specimen in its habitat, Coatepec, Veracruz. Photograph by Rodrigo Carral. (**D**) Specimen cultivated in Mexico city. Photograph by Ethian Licona, based on *M.A. Soto Arenas 9506* (AMO).

**Figure 76:**
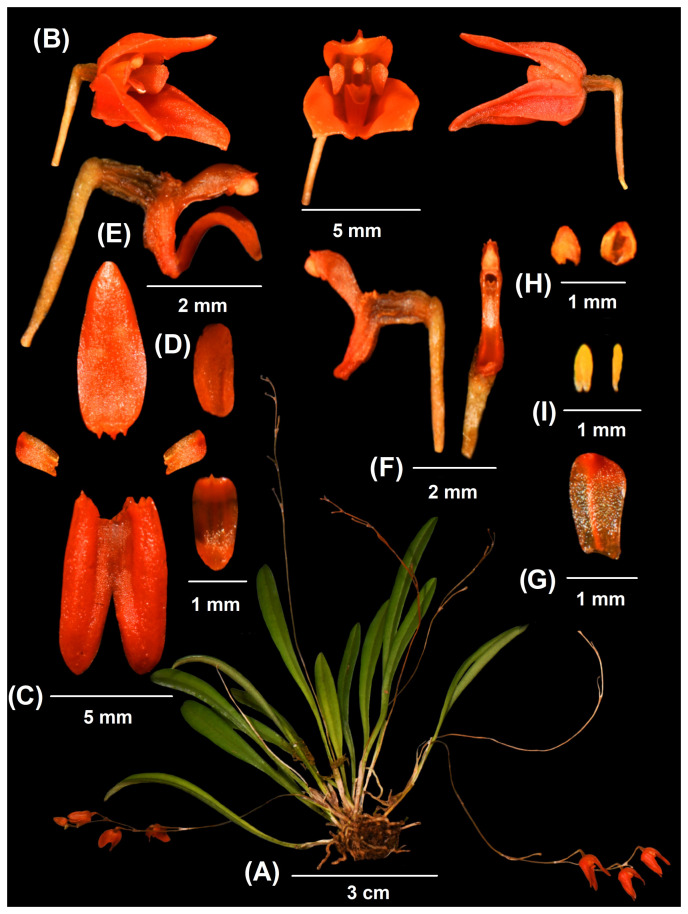
LCDP of *Specklinia lanceola*. (**A**) Habit. (**B**) Flower, ¾, frontal and lateral views. (**C**) Dissected flower. (**D**) Lip, dorsal and ventral views. (**E**) Column, lip and pedicelate ovary in lateral view. (**F**) Column, lateral and ventral views. (**G**) Petal. (**H**) Anther, dorsal and ventral views. (**I**) Pollinia, lateral and frontal views. Plate by Ethian Licona, based on *M.A. Soto-Arenas 9506* (AMO).

Herb cespitose, erect, up to 7.5 cm tall without the inflorescence. Roots 0.5–0.8 mm gross. Rhizome 0.5–2.3 mm long between adjacent stems. Stem 3.0–14.0 mm long, 0.5–0.7 mm gross, sheaths 3.0–9.1 mm long. Leaf 26.0–51.0 mm × 2.0–7.1 mm, linear–oblanceolate to linear–lanceolate, acute, coriaceous, flattened, glabrous, the apical mucro 0.5–0.9 mm long, light green, gradually attenuated into a petiole 4.0–10.0 mm long, 0.4–0.9 mm gross. Inflorescence 3.5–8.2 cm long, lax, flexuous, pendulous, glabrous; spathaceous bract 0.7–0.9 mm long; peduncle almost as long as the leaf, 2.7–5.5 cm long, 0.2–0.4 mm gross, with 3 bracts 1.5–2.0 mm long, membranaceous, glabrous, hyaline; rachis 0.8–2.7 cm long, with 2–6, simultaneous, secund flowers. Floral bracts 1.2–2.7 mm long, obliquely tubular-infundibuliform, obtuse, shortly apiculate, membranaceous, glabrous, hyaline. Flowers 3.5–7.8 mm × 3.5–4.8 mm; sepals, petals, lip, column and foot bright orange; anther yellowish-orange. Sepals concave, axially keeled, glabrous, the keels slightly dentate; dorsal sepal 3.5–7.6 mm × 1.4–3.0 mm, ovate to lanceolate, obtuse, shortly mucronate, 3-veined, the margin slightly recurved towards theapex; lateral sepals 3.8–7.8 mm × 2.0–4.0 mm, connate in the basal ⅓, each one oblong–lanceolate to elliptic–lanceolate, obtuse, shortly mucronate, 2-veined, gibbose in the base. Petals 1.7–3.4 mm × 0.7–1.6 mm, obliquely oblong–obovate, truncate to widely obtuse, thickened and shortly mucronate at the apex, 1-veined. Lip 1.8–3.4 mm × 0.7–1.2 mm, fleshy, oblong–elliptic to oblong–ligulate, obtuse to truncate, 3-veined, minutely papillar-glandulous, with a pair of submarginal calli, channeled between them. Column 2.0–2.5 mm long, 0.6–0.9 mm width, without wings; clinandrium obtuse, dentate; foot 1.4–2.0 mm long, 0.5–0.8 mm width, obtuse. Stigmatic cavity 0.4 mm × 0.5 mm, elliptic–oblong; rostellum laminar, oblong. Anther 0.6–0.8 mm long, 0.4–0.6 mm width, elliptic–ovoid. Pollinia 0.25–0.4 mm × 0.15–0.25 mm, clavate. Ovary 0.8–2.0 mm long, 0.4–0.8 mm gross, obconical, recurved, glabrous, keeled, the keels undulate, coopery orange; pedicel 1.2–7.5 mm long, 0.2–0.5 mm gross, glabrous. Capsule 5.0–6.5 mm long, 2.4–3.2 mm gross, elliptic–oblong, yellowish-orange (Figure 75 and Figure 76).

Distribution: Mexico, Guatemala, El Salvador, Honduras, Nicaragua, Costa Rica, Panama, Colombia and Jamaica. In Mexico, the species is distributed in the states of Chiapas, Oaxaca and Veracruz. It occurs in the Floristic Provinces of Costa del Golfo de México, Costa Pacífica, Serranías Transístmicas, El Soconusco, Sierra Madre Oriental and Valle de Tehuacán.

**Figure 77:**
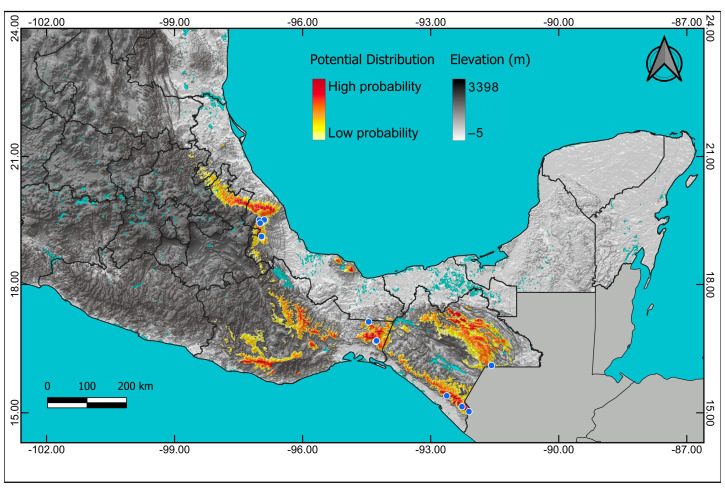
Potential distribution of *Specklinia lanceola* in Mexico and the location of currently known occurrence records (blue dots). Map by Ethian Licona.

The accepted potential distribution model for *S. lanceola* yields an AUC = 0.954, where the most influential environmental variables were temperature annual range (38.9%), max temperature of the warmest month (17.7%) and the Digital Elevation Model of Mexico (16.3%). The potential distribution area was estimated at 48,015.16 km^2^, representing approximately 2.44% of Mexico’s total territory (Figure 77).

Habitat: Epiphyte on *Quercus* sp., also recorded on *Citrus × sinensis* and *Coffea arabica*; sometimes as a lithophyte. The species grows in the evergreen tropical forest, *Quercus* forest, tropical mountain cloud forest and secondary vegetation derived from these forest types, as well as orange orchards and coffee plantations, at elevations between 850 and 1760 m.

Phenology: Flowering occurs from January to September. Mature fruits have been observed from April to November.

Conservation status: *Specklinia lanceola* is classified as a species Under Special Protection (Pr) in the list of endangered plants in Mexico [78]. After the reassessment of its conservation status following the MER criteria, a score of 1.23 was obtained, which suggests that for this species it is not necessary to include in any risk category. In the evaluation under IUCN criterion B, it achieved an EOO of 64,895.562 km^2^ and an AOO of 48.00 km^2^, suggesting that this species should be classified as Near Threatened (NT).

Additional commentaries: *Specklinia lanceola* can be recognized by its linear–oblanceolate, acute leaves; pendulous, lax, flexuous raceme with a peduncle as long as the leaf; up to 6 bright orange, secund, simultaneous flowers; dorsal sepal ovate; lateral sepals connate in the basal ⅓, each one oblong–lanceolate; petals obliquely oblong–obovate, truncate, thickened and shortly mucronate at the apex; lip oblong–elliptic, truncate.

Some specimens housed in the AMO Herbarium bear the unpublished name *Pleurothallis acevedoi*, which was presumably assigned when the species was first discovered in Mexico. Subsequently, a more detailed study demonstrated that these specimens actually belong to *S. lanceola*, leading to the abandonment of the provisional name.

This is the type species of *Specklinia* and exhibits several distinctive characters that make it easy to recognize. However, the Jamaican specimens, from which the type originates, differ morphologically from the continental populations. They can be distinguished by its shorter habit (up to 5.3 cm tall without the inflorescence vs. up to 7.5 cm tall); clearly lanceolate leaves, 13.0–34.0 × 1.5–3.2 mm (vs. linear–oblanceolate, 26.0–51.0 × 2.0–7.1 mm); inflorescence shorter than the leaf, erect and up to 4.4 cm long (vs. longer than the leaf, pendulous and up to 8.2 cm long); rachis up to 4 flowers (vs. up to 6 flowers,); and paler orange flowers, up to 5.6 mm long (vs. bright orange, up to 7.6 mm long).

On the other hand, *S. lanceola* has often been confused in Mexico with *S. vittariifolia*; however, it can be distinguished by its shorter habit (up to 6.2 cm tall without the inflorescence vs. up to 7.5 cm tall); acicular, triquetrous leaves, 10.0–45.0 × 0.5–2.6 mm (vs. linear–oblanceolate, flattened, 26.0–51.0 × 2.0–7.1 mm); inflorescence up to 7.0 cm long, congested, erect, glandulous (vs. up to 8.2 cm long, lax, pendulous, glabrous); glandulous, successive flowers (vs. glabrous, simultaneous); obovate–spathulate, apiculate, 2-veined petals (vs. oblong–obovate, truncate–mucronate, 1-veined); and 3-lobulate lip (vs. entire lip).

Examined specimens: MEXICO. Chiapas: Acacoyagua, Monte Ovando, 01.06.1937, *E. Matuda 28541* (MEXU!). La Trinitaria, km 18.9 camino Montebello-Marqués de Comillas, parte alta de la cañada del Río Veracruz (río Seco), 25.04.2000, *M.A. Soto-Arenas 9506* (AMO!). Tapachula, Santa Rosalía, cafetal abandonado cerca del río, 03.05.2008, *L. Jiménez 240* (ECO-TA-H!); Santa Rosalía, Cafetal camino al Chorro, 03.05.2008, *A. Damon s/n* (ECO-TA-H!). Unión Juárez, Santo Domingo, 01.07.1997, *F. Gálvez s/n* (ECO-TA-H!). Oaxaca: Santa María Chimalapa, Sierra de Tres Picos, ladera N, al E de Rancho Alegre y Rodríguez Clara, Veracruz, 05.04.1996, *M.A. Soto-Arenas 7905, G. Salazar, O. Rocha, E. Tenorio & T. Wendt* (AMO!); Campamento Perdiz, ca. 2.5 km al W de Díaz Ordáz, 16.03.1997, *S. Salas 1375, E. Torres, J. Rivera & R. García* (SERBO!). San Miguel Chimalapa, El peñasco, 3–4 km al W del paraje palmero El Gringo, al N del cerro Tres Picos, 26.08.1986, T. *Wendt 5415, M. Ishiki & S. Maya* (CHAPA!); paraje palmero El Venado, cerca del parteaguas continental, en la región del cerro El Retén, 28.07.1986, *S. Maya 3668* (CHAPA!). Veracruz: Coatepec, Planta comprada en Coatepec a Carmen González, aparentemente del Cofre de Perote, cerca de Coatepec, 01.09.1997, *M.A. Soto-Arenas 7299* (AMO!). Huatusco, forest near Huatusco, 01.09.1954, *F. Johnson 954* (AMES!, SEL!). Xalapa, Reserva Natural El Tejar, 01.01.1894, *C. Smith 1899* (SEL!).

Additional records: MEXICO. Veracruz: Coatepec, al N de Cinco Palos, sobre el camino al Zapotal, 15.01.2024, *R. Carral s/n & Y. Amano* (Photograph). Xico, parcelas detrás del panteón de Xico, 03.09.2021, *J. Portugal s/n* (Photograph).

19. ***Specklinia spectabilis*** (Ames & C.Schweinf.) Pupulin & Karremans, Phytotaxa, 63: 15–18. 2012.

**Figure 78:**
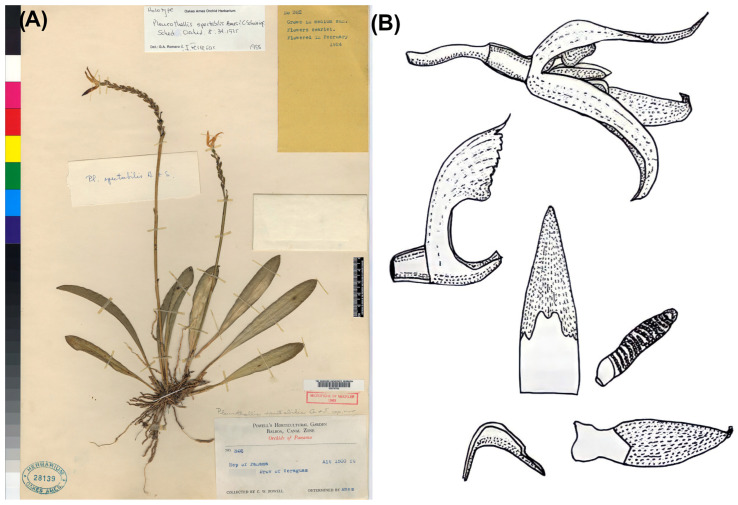
(**A**) Holotype of *Pleurothallis spectabilis*, *C.W. Powell 382* (AMES-74765), courtesy of The Orchid Herbarium of Oakes Ames of Harvard University. (**B**) Illustration based on the type of *Specklinia daviesii*, drawing by Fredy Archila based on *F. Archila s/n* (from Archila et al., 2015).

Basionym: *Pleurothallis spectabilis* Ames & C.Schweinf., Schedul. Orchid., 8: 34–35. 1925. TYPE: PANAMA. Veraguas: Santa Fé, 1500 ft., Feb 1924, *C. Powell 382* (holotype: AMES-74765! (Figure 78A)).

Etymology: From the Latin *spectabilis*, “remarkable, admirable”, referring to the striking and colorful nature of its flowers.

Heterotypic synonym: *Specklinia daviesii* Archila, Jiménez-Rodr. & Véliz, Moscosoa, 19: 18–19. 2015. TYPE: GUATEMALA. Alta Verapaz: Tactic, 1200 m, Aug 1998. *F. Archila s/n* (holotype: BIGU, not seen (Drawing based on the type Figure 78B)).

Etymology: Named after Kevin Davies, English orchideologist, specialist in vegetal anatomy.

**Figure 79:**
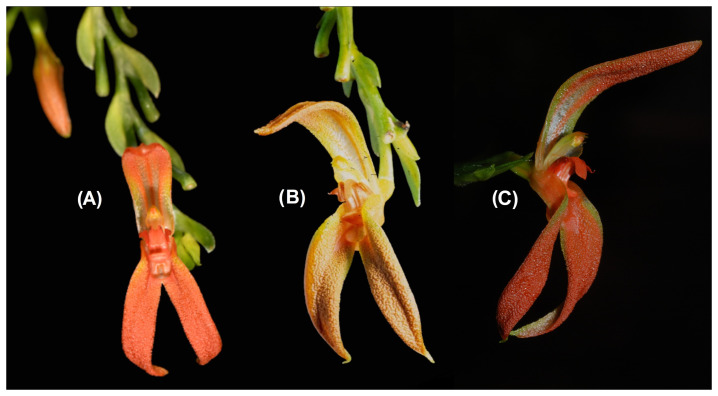
Photographs of *Specklinia spectabilis*. (**A**) Specimen cultivated in Mexico City. Photograph by Rodolfo Solano, based on *G. Salazar et al., 4550* (AMO). (**B**) Specimen cultivated in Mexico City. Photograph by Rodolfo Solano, based on *M.A. Soto Arenas et al., 9484* (AMO). (**C**) Specimen cultivated in Mexico City. Photograph by Gerardo Salazar, based on *M.A. Soto Arenas et al., 9495* (AMO).

**Figure 80:**
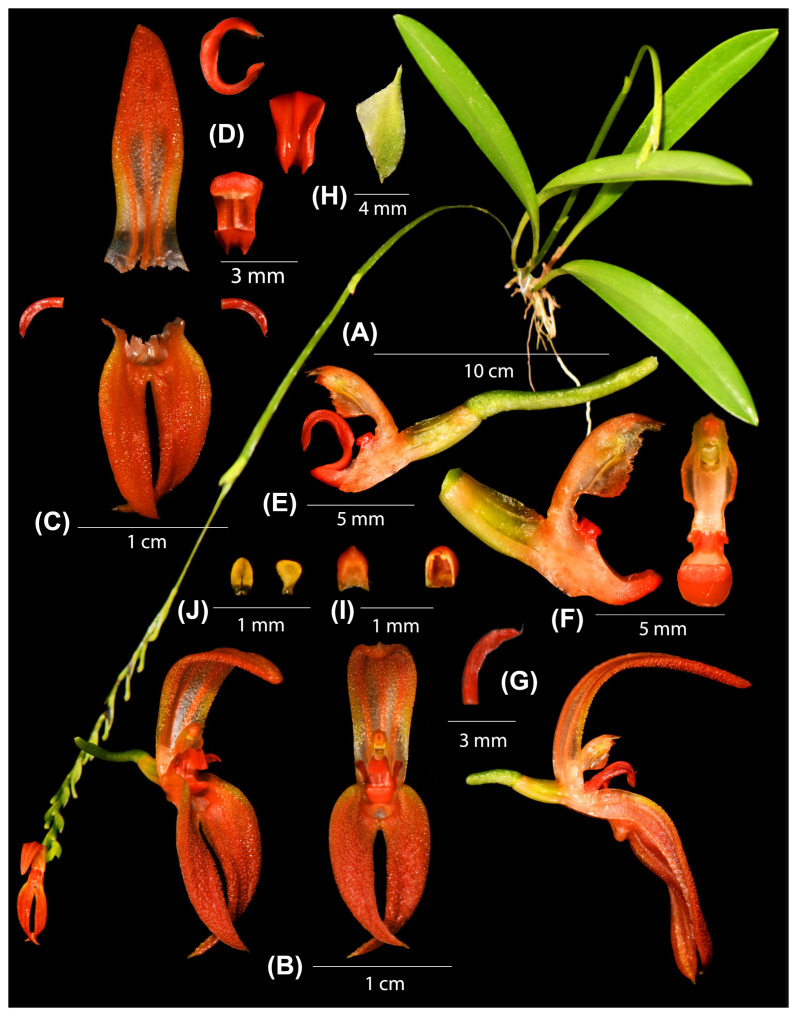
LCDP of *Specklinia spectabilis*. (**A**) Habit. (**B**) Flower, ¾, frontal and lateral views. (**C**) Dissected flower. (**D**) Lip, dorsal and ventral views. (**E**) Column, lip and pedicelate ovary in lateral view. (**F**) Column, lateral and ventral views. (**G**) Petal. (**H**) Floral bract. (**I**) Anther, dorsal and ventral views. (**J**) Pollinia, lateral and frontal views. Plate by Ethian Licona, based on *M.A. Soto Arenas et al., 9484* (AMO).

Herb cespitose, erect, up to 24.6 cm tall without the inflorescence. Roots 0.7–1.4 mm gross. Rhizome 3.0–9.0 mm long between adjacent stems. Stem 0.5–2.9 cm long, 0.8–1.5 mm gross, sheaths 0.5–1.5 cm long. Leaf 4.1–15.7 cm × 0.7–1.5 cm, membranous, flattened, narrowly oblanceolate, acute, glabrous, the margins slightly recurved, the apical mucro 0.4–0.7 mm long, light green, gradually attenuated into a petiole 1.0–6.0 cm long, 1.0–1.9 mm gross. Inflorescence 23.6–63.6 cm long, congested, ancipitous, flexuous, glabrous; spathaceous bract 3.0–4.0 mm long; peduncle longer than the leaf, 14.6–26.7 cm long, 1.3–2.4 mm width, with 2–3 bracts 4.5–6.0 mm long, 1.5–2.8 mm width, obliquely infundibuliform, acuminate, ancipitous, fleshy, glabrous, light green; rachis 4.0–36.9 cm long, with equidistant internodes, distant by 5.0–15.0 mm long, the first internode longer, with 8–40, successive, secund flowers, one open at a time. Floral bracts 5.0–11.0 mm long, 3.0–8.0 mm width, obliquely infundibuliform, acute, apiculate, ancipitous, fleshy, glabrous, imbricate, light green. Flowers 17.0–24.0 mm × 6.0–10.5 mm; sepals bright orange, with margins bright yellow; petals and lip orange to reddish-orange; column orange, ventrally yellow, foot reddish-orange; anther orange. Sepals concave, axially keeled, adaxially verrucose, abaxially glabrous, with margins slightly recurved apically, 3-veined; dorsal sepal 18.0–24.0 mm × 4.0–6.0 mm, slightly pandurate, narrowly lanceolate to oblong–lanceolate, acuminate; lateral sepals 16.0–22.0 mm × 3.5–4.5 mm, connate near to the base, each one narrowly lanceolate to oblong–lanceolate, acuminate, falcate, reflex, divergent, sometimes overlapping on the apices. Petals 4.0–5.1 mm × 0.7–1.0 mm, linear–lanceolate, acute, sub-terete, falcate, with a terete, filamentous apical projection, adaxially papillose–striated, abaxially glabrous, 2-veined. Lip 5.5–7.5 mm × 2.2–2.8 mm, fleshy, strongly arched, oblong–subpandurate, rounded to truncate, apically mucronate, 3-veined, minutely papillar-glandulous, the margins recurved, with a pair of submarginal calli, channeled between them. Column 5.9–6.5 mm long, 2.0–3.2 mm width; wings widely triangular, oblique, obtuse, erose to dentate, 2.5–3.0 mm × 1.0–1.4 mm; clinandrium acute, mucronate, dentate; foot 4.5–5.7 mm long, 1.2–2.1 mm width, obtuse, with a pair of columnar calli at the base, apically broadened. Stigmatic cavity 0.7 mm × 1.2 mm, oblong–subsquare; rostellum laminar, oblong. Anther 1.0–1.6 mm long, 0.5–1.3 mm width, elliptic–ovoid, mucronate. Pollinia 0.5–0.9 mm × 0.3–0.6 mm, obovoid. Ovary 3.5–5.0 mm long, 1.0–1.8 mm gross, obpyramidal, triquetrous, straight, glabrous, greenish-orange; pedicel 6.0–14.0 mm long, 0.7–1.3 mm gross, glabrous. Capsule 2.0–2.4 cm long, 0.8–1.5 cm gross, elliptic–oblong to elliptic–obovoid, yellowish-green (Figure 79 and Figure 80).

Distribution: Mexico, Guatemala, Belice, El Salvador, Honduras, Nicaragua, Costa Rica and Panama. In Mexico, the species is known only from the state of Chiapas, occurring in the Floristic Province of Serranías Transístmicas.

**Figure 81:**
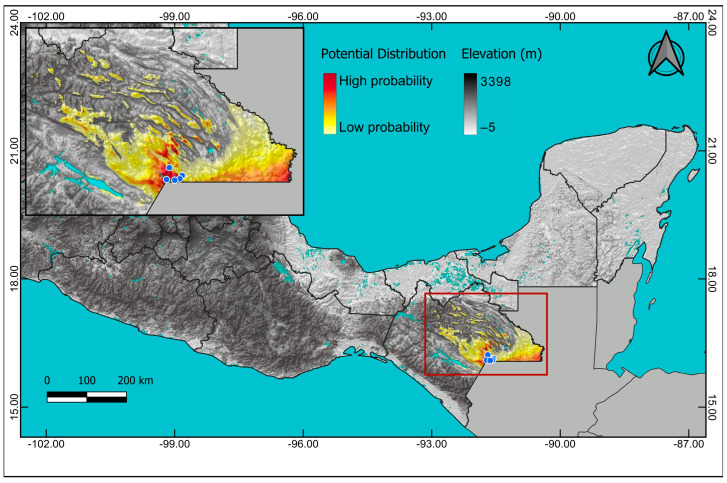
Potential distribution of *Specklinia spectabilis* in Mexico and the location of currently known occurrence records (blue dots). Map by Ethian Licona.

The accepted potential distribution model for *S. spectabilis* yields an AUC = 0.968, where the most influential environmental variables were temperature seasonality (62.3%), precipitation of the wettest quarter (31.3%) and the Digital Elevation Model of Mexico (5.0%). The potential distribution area was estimated at 7681.38 km^2^, representing approximately 0.39% of Mexico’s total territory (Figure 81).

Habitat: Epiphyte on *Quercus* sp. The species grows in the evergreen tropical forest and tropical mountain cloud forest, at elevations between 850 and 1560 m.

Phenology: Flowering occurs throughout the year, predominantly from August to May. Mature fruits have been observed from October to July. The flowers emit a diurnal scent reminiscent of fermented fruit.

Conservation status: In the past, this species and other morphologically similar taxa present in Mexico were treated as *S. endotrachys* [60] or *S. spectabilis* [91]. Under either name they were included in the list of threatened Mexican plants [78] in the category of Under Special Protection (Pr). However, in the reassessment of its conservation status following the MER criteria [78], a score of 2.14 was obtained, indicating that this species should be classified independently as Endangered (P). In the evaluation under IUCN criterion B, it achieved an EOO of 223.842 km^2^ and an AOO of 24.00 km^2^, suggesting that this species should be classified as Endangered (EN).

Additional commentaries: *Specklinia spectabilis* can be recognized by its cespitose habit; with relatively tall plants, reaching up to 25.0 cm in height, without the inflorescence; narrowly oblanceolate, acute leaves; inflorescence up to 63.6 cm long, flexuous, congested, ancipitous, with a peduncle flattened, longer than the leaf; floral internodes evenly spaced along the rachis; floral bracts ancipitous; bearing up to 40, bright orange flowers; lateral sepals lanceolate, reflex, falcate, sometimes overlapping in the apices; petals linear–lanceolate, with a filamentous apical projection; lip oblong–subpandurate, truncate, strongly arched.

This species belongs to the *Specklinia endotrachys* complex. The morphological differences among the Mesoamerican species of this complex are summarized in Table 6.

Examined specimens: MEXICO. Chiapas: La Independencia, 21–23 km along the road from Lake Tziscao to Santa Elena, 15.05.1988, *D.E. Breedlove 67924 & M. Bourell* (CAS!). La Trinitaria, 10 km WNW from Dos Lagos, above Santa Elena, 14.10.1981, *D.E. Breedlove 53566* (CAS!); 10 km ENE from Dos Lagos, above Santa Elena, 19.01.1982, *D.E. Breedlove 57495 & F. Almeda* (CAS!); Parque Nacional Lagunas de Montebello, Dos Lagos, 01.09.1984, *D. Mally 24* (CAS!); km. 18.9 del camino Montebello-Marqués de Comillas, parte alta de la cañada del Río Veracruz (Río Seco), 25.04.2000, *M.A. Soto Arenas 9484, S. Maldonado & L. López* (AMO!); km. 18.9 del camino Montebello-Marqués de Comillas, parte alta de la cañada del Río Veracruz (Río Seco), 25.04.2000, *M.A. Soto Arenas 9485, S. Maldonado & L. López* (AMO!).

Additional records: GUATEMALA. Alta Verapaz: Cobán, lomas al N de Cobán, hacía el río Sachichá, 11.02.1990, *G. Salazar 4550, M.A. Soto Arenas, E. Hágsater, R.L. Dressler, M.A. Dix, M.W. Dix & O. Mittelstaedt* (AMO!); Orquigonia, 15.03.2024, *M. León s/n* (https://www.inaturalist.org/observations/202544874. Accessed 30 April 2025).

20. ***Specklinia tribuloides*** (Sw.) Pridgeon & M.W.Chase, Lindleyana, 16 (4): 259. 2001.

**Figure 82:**
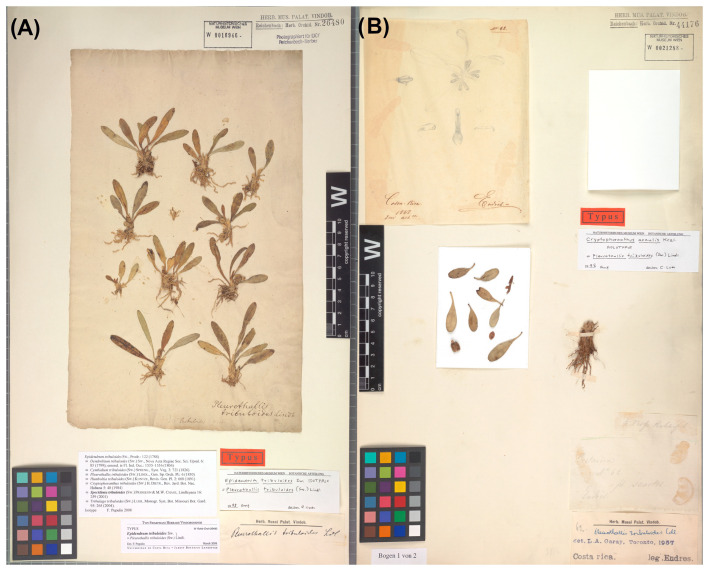
(**A**) Specimen selected as isolectotype for *Epidendrum tribuloides*, *O. Swartz s/n* (W-16946), courtesy of the Herbarium of Naturhistorisches Museum Wien. (**B**) Holotype of *Cryptophoranthus acaulis*, *A. Éndres 52* (W-21288), courtesy of the Herbarium of Naturhistorisches Museum Wien.

Basionym: *Epidendrum tribuloides* Sw., Prodr. 123. 1788. TYPE: JAMAICA. Unknown locality “*Habitat in sylvis interioribus Jamaicae; parasiticum arborum Crescentiae*”, undated, *O. Swartz s/n* (lectotype here designated: S-R-1975!; isolectotypes: BM-82281!, C (not seen), LD-1744870!, S-R-1974!, UPS (not seen), W-16946! (Figure 82A)).

Homotypic synonyms: *Dendrobium tribuloides* (Sw.) Sw., Nova Acta Regiae Soc. Sci. Upsal., ser. 2, 6: 83. 1799; *Cymbidium tribuloides* (Sw.) Spreng., Syst. Veg., ed. 16. 3: 721. 1826; *Pleurothallis tribuloides* (Sw.) Lindl., Gen. Sp. Orchid. Pl. 6. 1830; *Humboltia tribuloides* (Sw.) Kuntze, Revis. Gen. Pl. 2: 668. 1891; *Cryptophoranthus tribuloides* (Sw.) H.Dietr., Revista Jard. Bot. Nac. Univ. Habana, 5: 48. 1984; *Tribulago tribuloides* (Sw.) Luer, Monogr. Syst. Bot. Missouri Bot. Gard., 95: 265. 2004.

Etymology: From the Latin *tribulus*, “caltrop”, ancient war artifact with spines; referring to the similarity with the fruits of the genus *Tribulus* (Zygophyllaceae).

Heterotypic synonyms: *Pleurothallis spathulata* A.Rich. & Galeotti, Ann. Sci. Nat., Bot. sér. 3, 3: 17. 1845. TYPE: MEXICO. Veracruz: Xalapa, above Xalapa, 4000 ft., *H. Galeotti 5181* (apparently in W, not seen). Etymology: From the Latin *spathulatus*, “spathulate”, referring to the shapes of its leaves.

*Pleurothallis fallax* Rchb.f., Bonplandia, 3 (15): 224. 1855. LECTOTYPE (designated by Mora de Retana & Atwood [93]): MEXICO. Unknown locality. *P.F. Siebold s.n.* (W, not seen; sintype: Costa Rica. “*In Monte Irasu*”, *A.S. Ørsted s.n.* (W, not seen). Etymology: From the Latin *fallax*, “deceptive”, referring to its similarity with *Pleurothallis tribuloides*.

*Cryptophoranthus acaulis* Kraenzl., Repert. Spec. Nov. Regni Veg. Beih. 34: 232. 1925. TYPE: COSTA RICA. [Puntarenas] Mina Iglesias, Río Barranca. *A.R. Éndres 62* (Holotype: W-21288! (Figure 82B); drawings based on the type: AMES-41409!, W-21280!). Etymology: From the Latin *acaulis*, “without stem”, referring to its abbreviated stems.

**Figure 83:**
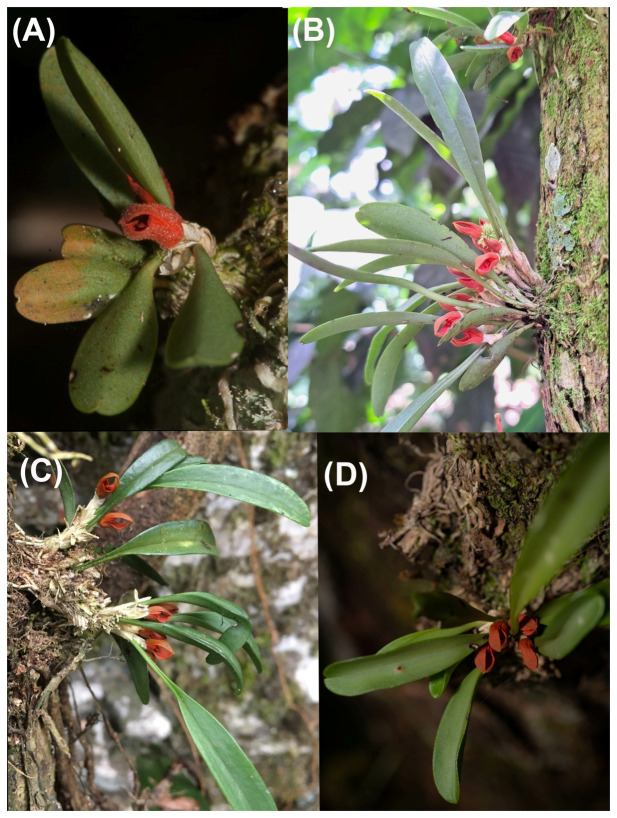
Photographs of *Specklinia tribuloides* in its habitat. (**A**) Maravilla Tenejapa, Chiapas. Photograph by Jesús Moreno. (**B**) Coatepec, Veracruz. Photograph by María Chávez. (**C**) Xilitla, San Luis Potosí. Photograph by Adríana García. (**D**) Villa Corzo, Chiapas. Photograph by Obet Sarmiento.

**Figure 84:**
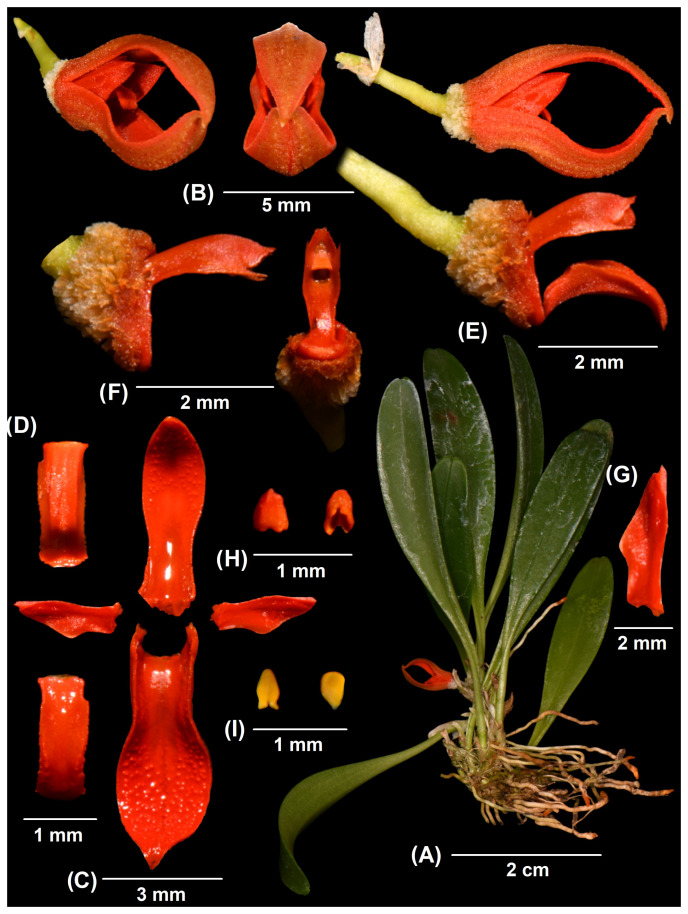
LCDP of *Specklinia tribuloides*. (**A**) Habit. (**B**) Flower, ¾, frontal and lateral views. (**C**) Dissected flower. (**D**) Lip, dorsal and ventral views. (**E**) Column, lip and pedicelate ovary in lateral view. (**F**) Column lateral and ventral views. (**G**) Petal. (**H**) Anther, dorsal and ventral views. (**I**) Pollinia, lateral and frontal views. Plate by Ethian Licona, based on *M.A. Soto Arenas et al. 9682* (AMO).

Herb spiral-ascending, erect, up to 9.8 cm tall. Roots 0.4–0.8 mm gross. Rhizome 1.6–6.0 mm long between adjacent stems. Stem 3.2–18.0 mm long, 0.6–1.0 mm gross, sheaths 1.7–8.0 mm long. Leaf 10.0–60.0 mm × 3.0–12.0 mm, narrowly oblong–obovate to obovate, obtuse to rounded, coriaceous, flattened, glabrous, the apical mucro 0.3–0.6 mm long, light green, gradually attenuated into a petiole 3.7–20.0 mm long, 0.6–1.2 mm gross. Inflorescence 3.0–11.0 mm long, shorter than the leaf, congested, erect, glabrous, concealed by the bracts; spathaceous bract 0.5–1.0 mm long; peduncle abbreviated, 1.0–4.0 mm long, 0.4–0.5 mm gross, with 1–2 bracts 2.8–6.4 mm long, papyraceous, striated to minutely verrucose, whitish; rachis 2.0–7.0 mm long, with 1–4 successive flowers, one or two open at a time. Floral bracts 1.2–5.0 mm long, obliquely tubular-infundibuliform, acute to acuminate, shortly apiculate, papyraceous, striated to minutely verrucose, whitish. Flowers 4.7–9.5 mm × 1.6–3.5 mm; sepals, petals, lip, column and anther orange. Sepals concave, axially carinate, densely papillose–verrucose on both surfaces, adherent at their apices, leaving a pair of lateral windows; dorsal sepal 4.8–8.6 mm × 1.5–3.0 mm, oblong–lanceolate, pandurate, acuminate, 3-veined; lateral sepals 4.7–9.5 mm × 2.0–3.8 mm, totally connate into an oblong–oblanceolate, pandurate, acuminate synsepal, 6-veined, gibbose in the base. Petals 2.0–4.5 mm × 0.6–1.8 mm, obliquely oblong–lanceolate, acuminate, fleshy, slightly concave, minutely apiculate, 2-veined. Lip 2.1–3.0 mm × 0.5–1.5 mm, fleshy, minutely 3-lobulate, 3-veined, papillar–verrucose, marginally erose–denticulate, with a pair of verrucose submarginal calli, channeled between them; lateral lobes elliptic to triangular, erect; mid lobe oblong–obovate, obtuse to truncate, recurved. Column 2.1–2.8 mm long, 0.5–1.0 mm width; wings subulate, acuminate, straight, entire, 0.5–0.7 mm × 0.1–0.2 mm; clinandrium obtuse, dentate; foot 0.9–1.8 mm long, 0.3–0.7 mm width, obtuse. Stigmatic cavity 0.3–0.4 mm × 0.3–0.4 mm, elliptic–orbicular; rostellum laminar, oblong. Anther 0.5–0.7 mm long, 0.4–0.5 mm width, elliptic–ovoid, apically verrucose. Pollinia 0.3–0.4 mm × 0.2–0.4 mm, obovoid. Ovary 0.6–1.4 mm long, 1.0–2.6 mm gross, obconical, straight, equinate- verrucose, orange; pedicel 1.5–4.5 mm long, 0.6–1.2 mm gross, verrucose. Capsule 4.0–7.5 mm long, 2.6–5.5 mm gross, ellipsoid to globose, equinate-verrucose, yellowish-green (Figure 83 and Figure 84).

Distribution: Mexico, Guatemala, Belize, El Salvador, Honduras, Nicaragua, Costa Rica, Panama, Cuba, Haiti and Jamaica. In Mexico, the species is distributed in the states of Chiapas, Guerrero, Oaxaca, Puebla, Querétaro, San Luis Potosí, Tabasco, Veracruz and it is probably present in Hidalgo. It occurs in the Floristic Provinces of Costa del Golfo de México, Costa Pacífica, Depresión del Balsas, Serranías Meridionales, Serranías Transístmicas, El Soconusco, Sierra Madre Oriental and Valle de Tehuacán. Although there are historical records from the Balsas Depression region in Guerrero (*H. Moore 59*, 1950, AMES) and Puebla (*H. Bravo 557*, 1950, MEXU), this species has not been observed nor collected in this province in recent times.

**Figure 85:**
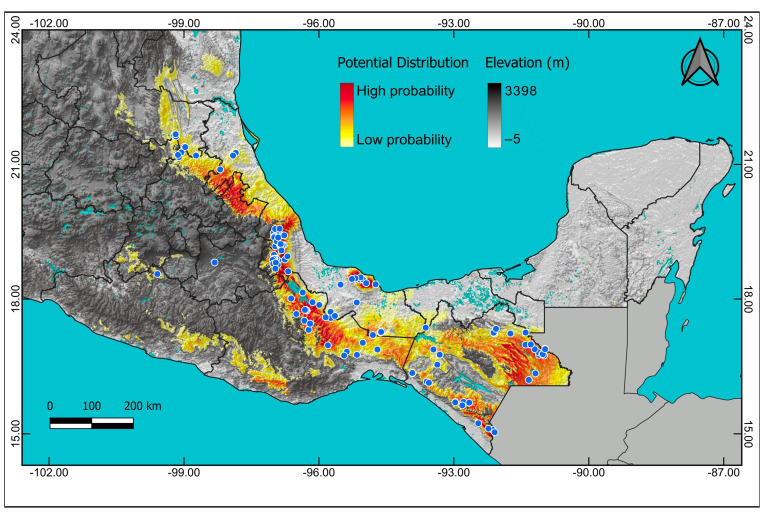
Potential distribution of *Specklinia tribuloides* in Mexico and the location of currently known occurrence records (blue dots). Map by Ethian Licona.

The accepted potential distribution model for *S. tribuloides* yields an AUC = 0.927, where the most influential environmental variables were the Digital Elevation Model of Mexico (17.5%), annual precipitation (15.7%), and isothermality (11.5%). The potential distribution area was estimated at 144,649.89 km^2^, representing approximately 7.36% of Mexico’s total territory (Figure 85).

Habitat: Epiphyte on *Albizia purpusii* Britton & Rose, *Brosimum alicastrum*, *Ceiba pentandra*, *C. aesculifolia* (Kunth) Britten & Baker f., *Cojoba arborea*, *Crescentia* sp., *Ficus* sp., *Manilkara zapota*, *Platymiscium* sp., *Plumeria rubra*, *Pseudobombax ellipticum*, *Quercus* sp., *Spondias mombin*, *Tabebuia rosea*, *Vachellia pennatula* (Schltdl. & Cham.) Seigler & Ebinger and *Yucca* sp., also recorded on *Citrus x sinensis*, *Coffea arabica*, *Mangifera indica* and *Tamarindus indica*, occasionally also as a lithophyte. The species grows in the evergreen tropical forest, semi-deciduous tropical forest, tropical deciduous forest, thorn forest, rupicolous xerophytic scrub, *Quercus* forest, riparian vegetation, secondary vegetation derived from these forest types, as well as in orange, mango and tamarind orchards, coffee plantations and urban parks, at elevations between 5 and 2370 m.

Phenology: Flowering occurs from April to February. Mature fruits have been observed from June to April. The flowers emit a diurnal scent reminiscent of fermented fruit.

Conservation status: In the assessment of its conservation status following the MER criteria [78], a score of 0.16 was obtained, therefore it is not necessary to include it in any risk category. In the evaluation under IUCN criterion B, it achieved an EOO of 290,034.292 km^2^ and an AOO of 468.00 km^2^, suggesting that this species should be classified as Least Concerned (LC).

Additional commentaries: *Specklinia tribuloides* can be recognized by its spiral-ascending habit; oblong–obovate, obtuse to rounded leaves; abbreviated, congested raceme; bright orange flowers; sepals acuminate, papillose–verrucose, adherent in their apices, leaving two lateral windows; dorsal sepal oblong–lanceolate; synsepal oblong–oblanceolate; petals obliquely oblong–lanceolate; lip minutely 3-lobulate, lateral lobes elliptic to triangular, erect; mid lobe oblong–obovate, obtuse–truncate, recurved, erose–denticulate.

There are records of specimens within this species exhibiting a flava color variant (https://www.facebook.com/photo/?fbid=1479305782604895&set=a.162416664293820. Accessed 28 April 2025).

This species is similar to *S. blancoi*, which differs by its cespitose habit (vs. spiral-ascending); linear, truncate leaves (vs. obovate, obtuse); flowers up to 6.8 mm long (vs. up to 9.5 mm long); obtuse sepals with the apices free (vs. acuminate, adherent at their apices); dorsal sepal oblong–obovate (vs. oblong–lanceolate); synsepal oblong–obovate (vs. oblong–oblanceolate); petals obovate, obtuse (vs. lanceolate, acuminate).

Examined specimens: MEXICO. Unknown locality, 15.03.1962, *R. Spencer s/n* (F!). Chiapas: Ángel Albino Corzo, Reserva de la Biósfera El Triunfo, undated, *J. Castillo s/n* (CHIP!). Berriozábal, 1 km al NW del entronque Aeropuerto-Ocozocoautla, sobre la carretera 190, 03.03.1988, *A. Reyes-García 396, M. Sousa & E. Martínez* (MEXU!, MO!). Cacahoatán, Benito Juárez El Plan, 01.10.2008, *A. Damon s/n* (ECO-TA-H!). Chilón, ca. km 17 del camino Chancalá-Monte Líbano, 01.04.2000, *M.A. Soto Arenas 9682, L. López & P. Schluetter* (AMO!); 60 km al SW de Palenque, por la carretera 199, cerca de Tzinteel, 31.08.1986, *P.M. Catling 16.19 & V.R. Brownell* (AMO!); arroyo Agua Azul, 15.08.1998, *G. Carnevali s/n* (CICY!); Cascadas de Agua Azul, 06.08.1985, *T. Chehaibar 121, A. Espejo & M. Palacios* (MEXU!). Huixtla, Colonia Morelos, 07.02.1999, *A. Damon s/n* (ECO-TA-H!). Jiquipilas, ejido Tiltepec, Rancho El Recuerdo, 06.06.2002, *A. Reyes-García 5030, C. Chavarría & E. Meléndez* (HEM!, MEXU!); Cerro La Palmita, 9 km al SW de Tierra y Libertad, 01.03.2003, *O. Farrera 3262* (MEXU!). La Concordia, 5 km de la Finca Cuxtepec, 10.05.1988, *T. Cabrera 71* (AMO!); camino Finca Cuxtepeques-Finca Álamos, polígono zona de amortiguamiento El Triángulo, 15.06.2006, *J. Martínez 1484* (HEM!); canyon NE of the slopes of Cerro Venado, above Finca Cuxtepeque, 10.05.1988, *D. Breedlove 67554 & M. Bourell* (MEXU!). Mapastepec, Reserva El Triunfo, Polígono 1, Cañada honda-El Tomatal, 23.04.1990, *M. Heath 885 & A. Long* (CHAPA!, MEXU!). Ocosingo, 4 km al W de Crucero Corozal, camino Palenque-Boca Lacantum, 10.08.1984, *E. Martínez 6895* (MEXU!, MO!); 4 km al W de Crucero Corozal, camino Palenque-Boca Lacantum, 19.09.1984, *E. Martínez 7636* (MEXU!); 2 km al S de Crucero Corozal, camino Palenque-Boca Lacantún, 21.09.1984, *E. Martínez 7693* (MEXU!); 8 km antes de llegar a Lacanjá, saliendo de Palenque, 29.07.1984, *A. Espejo 1066, M. Martínez, S. Hernández & L. Pacheco* (MEXU!); Lacanjá-Chanzayab, camino Palenque-Boca Lacantum, 07.11.1985, *E. Martínez 15076* (MEXU!); Crucero Nueva Palestina, 14 km al SE de Nuevo Guerrero, camino a Boca Lacantún, 07.01.1986, *E. Martínez 15554* (MEXU!); 2 km al SE de la colonia Benito Juárez Miramar, 21.08.1993, *A. Reyes-García 2098 & M. Sousa* (MEXU!); Yaxchilán, porción N de la Omega, 25.08.1998, *A. Rincón 1030* (MEXU!); 0.7 km al NW de Nuevo Guerrero, arroyo La Poza, 10.11.2002, *D. Álvarez 2407 & G. Aguilar* (MEXU!); 4.2 km al S de Santo Domingo, 21.11.2003, *D. Álvarez 7216 & C. Jiménez* (MEXU!). Ocozocoautla, Parque Educativo Laguna Bélgica, 17.08.2007, *I. Moreno s/n & G. López* (CHIP!). Salto del Agua, camino a La Boquilla-Cascada de Agua Azul, 25.10.2011, *D. Torres s/n* (CHIP!). Tapachula, Finca San Carlos, 01.05.2008, *A. Damon s/n* (ECO-TA-H!). Villa Corzo, Ejido Sierra Morena, cerca de los límites con Tonalá, 30.05.2002, *A. Reyes-García 4775, D. Gómez & C. Chavarría* (MEXU!); camino a Ejido Sierra Morena, 12.07.2004, *A. Reyes-García 7136, D. Gómez & E. Figueroa* (MEXU!). Guerrero: Taxco, on hills above Taxco, 04.04.1950, *H. Moore 59* (AMES!). Oaxaca: El Barrio de la Soledad, Torre de microondas Palma Sola, 10 km al W de Almoloya, 24.09.1984, *R. Torres 6174 & E. Cabrera* (MEXU!). Guevea de Humboldt, Cerro Picacho, 3 km al E de la cumbre, 27.06.1991, *A. Campos 3723* (MEXU!). San José Chiltepec, 12.02.1968, *G. Martínez 1631* (MEXU!, MO!). San Juan Bautista Tuxtepec, en predio La Joya del Obispo (La Joya de Sta. Ma. Jacatepec), rancho El Tochero, aprox. 10 km al E de San Agustín, 20.10.1990, *C.H. Ramos 446 & E. Martínez* (AMO!). San Juan Bautista Valle Nacional, km 160 de la carretera Oaxaca-Puerto Ángel, saliendo de Oaxaca, 28.12.1986, *E. Greenwood 1333 & O. Suárez* (AMO!); Ranchería Monte Bello, 5.5 km al SE de Santa Fé, 07.07.2009, *K. Velásco 3863, L. Vásquez, C. González, A. Hernández, J. Hernández & R. Pérez* (MEXU!); filo de la colindancia entre Yetla y Santa Fé, 08.07.2009, *K. Velásco 3949, L. Vásquez, C. González, & R. Cardoza* (MEXU!); cerro de La Loma, 3.3 km al E de Santa Fé, 05.09.2009, *K. Velásco 4272, L. Vásquez & E.D. Enríquez* (MEXU!). San Juan Guichicovi, carretera Matías Romero-Sarabia, 5.2 km de Sarabia, La Esmeralda, 21.04.1987, *I. Aguirre 1175 & N. Pozos* (AMO!). San Juan Lalana, 10 km al S de Xochiapa, sobre la carretera 147, 02.09.1986, *P.M. Catling 19.1 & V.R. Brownell* (AMES!, AMO!). San Miguel Soyaltepec, torre 38 de la L.T. Temascal II-Oaxaca Potencia, 10.11.2004, *G. Juárez 826 & A. Martínez-Feria* (MEXU!). San Pedro Teutila, Piedra Ancha.

Torre 120 de la L.T. Temascal II-Oaxaca Potencia, 1 km al S de Piedra Ancha, 07.04.2005, *G. Juárez 1651 & C. Cruz* (MEXU!). Santa María Chimalapa, 4–6 km al SW de Sta. María, por la vereda al Paso Cuycucjo, 13.06.1985, *H. Hernández 1266* (MEXU!, MO!). Puebla: San Diego La Mesa Tochimiltzingo, 01.08.1950, *H. Bravo 557* (MEXU!). Querétaro: Jalpan de Serra, 2–3 km al E de La Boquilla, S.L.P., sobre el río Santa María, 10.03.1993, *E. Carranza 4553* (IEB!, QMEX!); al NE de Tanchanaquito, por el cañón de La Vuelta de la Peña, 18.03.1997, *L. López 524* (IEB!); 7–8 km al N de Tanchanaquito, punto La Barranca Grande, 12.04.1997, *L. López 557* (AMO!, IEB!, MEXU!, QMEX!); al N de Tanchanaquito, punto La Barranca Grande, 15.07.1998, *L. López 679* (IEB!, MEXU!, QMEX!). Landa de Matamoros, camino entre Agua Zarca y El Río Moctezuma, 25.05.1990, *S. Zamudio 7865* (IEB!, MEXU!); Cerro de La Yesca, 14.03.2013, *L. Hernández-Sandoval 6520 & O. Guillemín* (QMEX!). San Luis Potosí: Tamazunchale, along Río Amajác, 01.07.1937, *C. Lundell 7201 & A. Lundell* (AMES!, MICH!, NYBG!). Xilitla, on the road to Xilitla, 01.01.1954, *F. Johnson 453–11* (SEL!). Tabasco: Huimanguillo, Villa de Guadalupe, undated, *M. González 23* (UJAT!). Tenosique, ejido Niños Héroes de Chapultepec, 30.01.2013, *J. Hernández 113* (UJAT!). Veracruz: Amatlán de los Reyes, rancho Chilpanapa, 15.05.1990, *C. Huerta 25* (AMO!). Catemaco, Coyame, 15.09.1966, *M. Sousa 2788* (MEXU!); Fraccionamiento Totonicapan, 3 km al N de Catemaco, 08.09.1983, *R. Cedillo 2496* (IEB!, MEXU!); Parque de la Flora y Fauna Silvestre Tropical, planicie del Cerro Pipiapan, 21.06.1994, *G. Carmona 148* (AMO!); Dos Amates, cerro de Chochobí, 20.12.2006, *B. Senterre 4376 & M. Parvais* (XAL!). Coatepec, entre Coatepec y Coatepec Viejo, cerca de la cascada de la Granada, 24.08.1986, *M. Cházaro 3985, P. Hernández y J. Camarillo* (XAL!); Cerro de La Campana, ca. 2 km al W del camino viejo entre Coatepec y Xalapa, cerca de La Pitahaya, 02.04.1993, *P. Hietz 841 & U. Seifert* (XAL!). Coetzala, Coetzala, 31.03.1976, *P. Valdivia 2195* (WIS!, XAL!). Córdoba, entrada a La Trinidad Chica, 04.06.1994, *O. Tejeda 5* (XAL!). Emiliano Zapata, Chavarrillo, 1.5 km adelante de la estación de Chavarrillo, 18.06.1994, *G. Castillo-Campos 12454* (IEB!, MEXU!, XAL!). Fortín, near Fortín, along the road Fortín-Córdoba, ca. 1 mile E of Fortín, on the road, N side, 28.06.1954, *G.B. Newcomb 133c* (UC!); en la Barranca del Metlác, 16.07.1983, *S. Zamudio 963a & A. Guadarrama* (UJAT!). Ixtaczoquitlán, Barranca del Metlac, 14.06.1992 *M. López 135* (AMO!). Paso del Macho, near Paso del Macho, 09.07.1932, *O. Nagel 2649* (AMES!). Playa Vicente, 6 km de la carretera El Nigromante-Santa Teresa, 03.10.1971, *J. Chavelas 4185* (MEXU!). Puente Nacional, Angostillo, carretera Totutla-Puente Nacional, 8 km al E de La Hacienda El Mirador, 17.05.1994, *A.R. López-Ferrari 1944, A. Espejo, J. García & L. Sánchez* (AMO!, IEB!). San Andrés Tuxtla, Laguna Encantada, Cráter volcánico, 3 km al E de S.A.T., 15.08.1953, *R. Dressler 78 & Q. Jones* (GH!); 2 km al W de San Andrés, Puerta Nueva Xoteapan, 12.05.1965, *M. Sousa 2415* (MEXU!); Laguna Encantada, 2 km al N de San Andrés, 06.08.1987, *R. Cedillo 3864* (MEXU!). San Juan Evangelista, Río San Juan, 116 km al SE de Cd. Alemán, 16.07.1983, *S. Zamudio 963* (IEB!). Santiago Sochiapam, 10 km S of Xochiapa, on the side of highway 147, 02.09.1986, *P. Catling 19 & V. Brownell* (AMES!). Soteapan, camino a Bastonal, 12.01.1974, *M. Sousa 4316* (MEXU!). Tatahuicapan, La Valentina, 2 km a partir del pueblo de Benigno Mendoza, faldas del volcán San Martín Pajapan, 07.09.1992, *A. Flores-Palacios 496* (XAL!). Teocelo, El Salto de Texolo, ladera arriba de la cascada, hacía el lado de Teocelo, 13.12.1988, *G.A. Salazar 4152* (AMO!). Tezonapa, Monte Almalonga, NE of Tezonapa, 14.06.1934, *O. Nagel 3598* (AMES!). Tlacotepec, cerro de Los Agustinos, El Moro, 17.05.1994, *A.R. López-Ferrari 1944, A. Espejo, J. García & L. Sánchez* (IEB!); 7 km W of Palmilla, along the highway to Huatusco, 10.08.1971, *W. Stevens 1411* (MO!). Totutla, Zacuapan, 01.07.1906, *C. Purpus 2124* (AMES!, GH!, UC!); Zacuapan, 10.08.1933, *C. Purpus 2997* (MEXU!); Zacuapan, 15.09.1933, *C. Purpus 2942* (AMES!); Zacuapan, 17.05.1994, *L. Sánchez 293, A.R. López-Ferrari. A. Espejo & C. García* (AMO!); Barranca de Zacuapan, 17.05.1994, *J. García-Cruz 679, L. Sánchez, A. Espejo & A.R. López-Ferrari* (AMO!); Barranca de Zacuapan, 13.06.1993, *S. Avendaño 3228 & T. Platas* (XAL!). Uxpanapa, 4 km del campamento Hermanos Cedillo y 200 m del lado N, hacía La Laguna, 28.05.1974, *P. Valdivia 781* (MEXU!, XAL!); 1.5 km del campamento Hermanos Cedillo, hacia La Escuadra, 24.06.1974, *P. Valdivia 976* (XAL!); 800 m del campamento Hnos. Cedillo hacia la escuadra, 24.07.1974, *P. Valdivia 1066* (XAL!); 800 m del campamento Hnos. Cedillo hacia la escuadra, 25.07.1974, *P. Valdivia 1101* (MEXU!, XAL!); 800 m del campamento Hnos. Cedillo hacia la escuadra, 26.07.1974, *P. Valdivia 1128* (HUAP!, IEB!, MEXU!); 1 km del campamento Hermanos Cedillo, hacia La Escuadra, 29.07.1974, *P. Valdivia 1182* (XAL!); río Soloxuchil, entre Hermanos Cedillo y La Escuadra, 30.07.1974, *M. Vásquez 904* (XAL!); 7 km pasando Río Alegre, 26.01.1975, *P. Valdivia 1886* (XAL!).

Additional records: MEXICO. Chiapas: Maravilla Tenejapa, Mirador Las Nubes, cerca de Gallo Giro, 02.08.2012, *J. Moreno s/n* (https://www.inaturalist.org/observations/2880841. Accessed 26 April 2025). Montecristo de Guerrero, 30.06.2014, *A. Jiménez s/n* (Photograph). Ocosingo, Yaxchilán, en la Omega, 19.07.2021, *S. Chan s/n* (https://www.inaturalist.org/observations/87764041. Accessed 26 April 2025); al NW de Nuevo Guerrero, 07.09.2021, *S. Miguel s/n* (https://www.inaturalist.org/observations/97399019. Accessed 26 April 2025); Área de Protección de Flora y Fauna Metzabok, 06.11.2024, *S. Enriquez s/n* (https://www.inaturalist.org/observations/253805700. Accessed 26 April 2025). Ocozocoautla, Parque Educativo Laguna Bélgica, 01.09.2018, *N. Ramírez s/n* (https://www.inaturalist.org/observations/16187605. Accessed 26 April 2025); Cerro Brujo, 03.05.2019, *E. Martínez-Ovando s/n* (Photograph). Tonalá, al NW de Sierra Morena, 29.12.2024, *C. Velasco-Macías s/n* (https://www.inaturalist.org/observations/257500669. Accessed 26 April 2025). Unión Juárez, Orquideario Santo Domingo, undated, *A. Damon s/n* (Photograph). Villa Corzo, Ejido Sierra Morena, 17.10.2019, *O. Sarmiento s/n* (https://www.inaturalist.org/observations/250740357. Accessed 26 April 2025). Oaxaca: Ixtlán de Juárez, San Miguel Tiltepec, 03.06.2023, *C. Tomás-López s/n* (Photograph). San Juan Lalana, Montenegro Lalana, 01.06.2022, *M. Correa s/n* (Photograph). San Juan Tabaá, 30.07.2022, *C. Tomás-López s/n* (Photograph). San Miguel Quetzaltepec, al N de Santa Cruz Quetzaltepec, 23.07.2023, *J. Mendoza s/n* (https://www.inaturalist.org/observations/175245314. Accessed 26 April 2025). Santa María Guienagati, 2.7 km al SW de Algodón, 03.10.2023, *H. Cruz-Luna s/n* (https://www.inaturalist.org/observations/186107936. Accessed 26 April 2025). Santiago Camotlán, al NW de Santiago Camotlán, 12.12.2023, *O. Renteria s/n* (https://www.inaturalist.org/observations/195776030. Accessed 26 April 2025). Querétaro: Jalpan de Serra, al W de Puerto de Tantzotzob, 24.07.2016, *U. Torres s/n* (https://www.inaturalist.org/observations/6357107. Accessed 26 April 2025). Landa de Matamoros, Neblinas, 26.12.2021, *J. Garay s/n* (https://www.inaturalist.org/observations/104370472. Accessed 26 April 2025); Neblinas, 05.01.2022, *J. Garay s/n* (https://www.inaturalist.org/observations/104370472. Accessed 26 April 2025). San Luis Potosí: Aquismón, Sótano de las Golondrinas, 31.12.2024, *K. Posadas s/n* (https://www.inaturalist.org/observations/343360692. Accessed 26 April 2025). Xilitla, al NW de La Conchita, 11.05.2019, *R. Llanos s/n* (https://www.inaturalist.org/observations/70708800. Accessed 26 April 2025); Jardín Surrealista de Edward James, 19.04.2021, *A. García s/n* (https://www.inaturalist.org/observations/74533207. Accessed 26 April 2025); Jardín Surrealista de Edward James, 19.04.2021, 08.03.2025, *E. Maqueda s/n* (https://www.inaturalist.org/observations/267388738. Accessed 26 April 2025); al NE de la Cascada El General, 02.06.2025, *C. Mendoza s/n* (https://www.inaturalist.org/observations/286298910. Accessed 26 April 2025). Veracruz: Amatlán de los Reyes, Facultad de Ciencias Biológicas y Agropecuarias Peñuela, Universidad Veracruzana, 24.02.2023, *I. Dávila s/n* (https://www.inaturalist.org/observations/150653007. Accessed 26 April 2025); Colegio de Postgraduados, Campus Córdoba, 11.07.2019, *V. de la Cruz s/n* (https://www.inaturalist.org/observations/28634084. Accessed 26 April 2025). Atoyac, Caballo Blanco, al S de Atoyác, 10.07.2023, *S. Space s/n* (https://www.inaturalist.org/observations/172202212. Accessed 26 April 2025). Chocamán, al S de San José Neria, 03.02.2023, *V. de la Cruz s/n* (https://www.inaturalist.org/observations/149536902. Accessed 26 April 2025). Coatepec, al E de Consolapan, 08.09.2022, *A. Rockefeller s/n* (https://www.inaturalist.org/observations/134936215¿. Accessed 26 April 2025); al NW de Hacienda Los Cafetales, 02.08.2024, *M. Chávez s/n* (https://www.inaturalist.org/observations/234008033. Accessed 26 April 2025); al E de Tixtla, 11.09.2025, *R. Carral s/n* (https://www.inaturalist.org/observations/313251471. Accessed 26 April 2025). Córdoba, Francisco Mórosini, junto al Río Jamapa, 09.11.2017, *S. Quijano s/n* (https://www.inaturalist.org/observations/8743308. Accessed 26 April 2025); Laguna El Porvenir, 30.10.2020, *G. León s/n* (https://www.inaturalist.org/observations/64358807. Accessed 26 April 2025); Paso Coyol, 22.12.2020, *V. de la Cruz s/n* (https://www.inaturalist.org/observations/66951259. Accessed 26 April 2025); Cervantes y Lozada, 18.07.2024, *G. Ríos s/n* (https://www.inaturalist.org/observations/230520236. Accessed 26 April 2025). Emiliano Zapata, al E de Chavarrillo, 11.03.2021, *L. Orea s/n* (https://www.inaturalist.org/observations/71590385. Accessed 26 April 2025). Fortín, al NE de Fortín de las Flores, 24.05.2020, *V. de la Cruz s/n* (https://www.inaturalist.org/observations/47138263. Accessed 26 April 2025); Fortín Viejo, Barranca del Metlác, 22.05.2021, *V. de la Cruz s/n* (https://www.inaturalist.org/observations/79970007. Accessed 26 April 2025); Fortín Viejo, Barranca del Metlác, 06.06.2021, *V. de la Cruz s/n* (https://www.inaturalist.org/observations/82172804. Accessed 26 April 2025). Huatusco, al W de Tepetzingo, 14.07.2021, *G. Torres s/n* (https://www.inaturalist.org/observations/87230455. Accessed 26 April 2025). Ixtaczoquitlán, Cuautlapan, 15.06.2021, *A.G.P. s/n* (https://www.inaturalist.org/observations/83141827. Accessed 26 April 2025); Tuxpanguillo, 27.06.2021, *G. Torres s/n* (https://www.inaturalist.org/observations/84893129. Accessed 26 April 2025); Zapoapan, 23.07.2023, *A. Díaz s/n* (https://www.inaturalist.org/observations/174963233. Accessed 26 April 2025); Cerro Chicahuaxtla 26.08.2023, *C. Vieyra-Rojas s/n* (https://www.inaturalist.org/observations/180458315. Accessed 26 April 2025); Cañón del Río Blanco, 25.06.2024, *O. Cerón-Jiménez s/n* (https://www.inaturalist.org/observations/225186955. Accessed 26 April 2025). Naranjal, al N de Naranjal, 10.09.2022, *P. Aguilar s/n* (https://www.inaturalist.org/observations/134574371. Accessed 26 April 2025). Sochiapa, al W de La Raya, 10.06.2023, *S. Romo s/n* (https://www.inaturalist.org/observations/166629935. Accessed 26 April 2025). Teocelo, al E de Teocelo, 30.05.2018, *J. Rojas s/n* (https://www.inaturalist.org/observations/13065760. Accessed 26 April 2025); al N de Tejería, 11.02.2024, *S. Díaz s/n* (https://www.inaturalist.org/observations/199136221. Accessed 26 April 2025). Tlalnelhuayocan, al SE de Tlalnelhuayocan, 06.03.2021, *R. Carral s/n* (https://www.inaturalist.org/observations/71884566. Accessed 26 April 2025). Tlaltetela, al S de Pinillo, 18.06.2020, *S. Díaz s/n* (https://www.inaturalist.org/observations/51734169. Accessed 26 April 2025). Xalapa, Xoloxtla, 28.08.2025, *T. Sánchez s/n* (https://www.inaturalist.org/observations/310653830. Accessed 26 April 2025). Xico, al N de Xico, 15.06.2019, *T. Colotl s/n* (https://www.inaturalist.org/observations/27060593. Accessed 26 April 2025). Zentla, al NE de Tepatlaxco, 03.06.2020, *R. Carral s/n* (https://www.inaturalist.org/observations/48412127. Accessed 26 April 2025). Zongolica, Xonamanca, 29.05.2022, *M. Cuevas s/n* (Photograph).

21. ***Specklinia vittariifolia*** (Schltr.) Pridgeon & M.W. Chase, Lindleyana, 16: 259. 2001.

**Figure 86:**
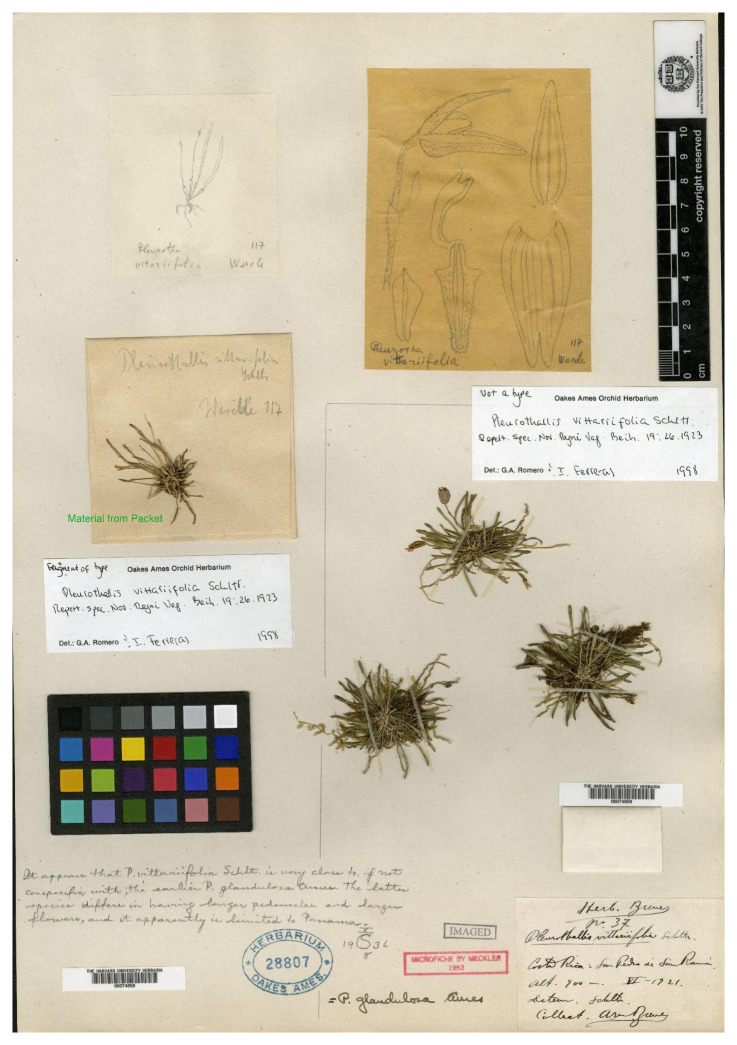
Lectotype of *Pleurothallis vittariifolia*, *K. Wercklé 117* (AMES-74858), courtesy of The Orchid Herbarium of Oakes Ames of Harvard University.

Basionym: *Pleurothallis vittariifolia* Schltr., Repert. Spec. Nov. Regni Veg. Beih. 19: 26. 1923. TYPE: COSTA RICA. [San José: Moravia] San Jerónimo, 1350 m, “*blühend im Juni 1921*”. *C. Wercklé 117* (holotype: B (destroyed); lectotype (designated by Pupulin [94]): *K. Wercklé 117*, AMES-74858! [with copies of drawings by Schlechter based on the holotype] (Figure 86)).

Etymology: From the Latin *vittariifolius*, “with leaves like *Vittaria*”, referring to the similarity with the leaves of the fern genus *Vittaria*.

**Figure 87:**
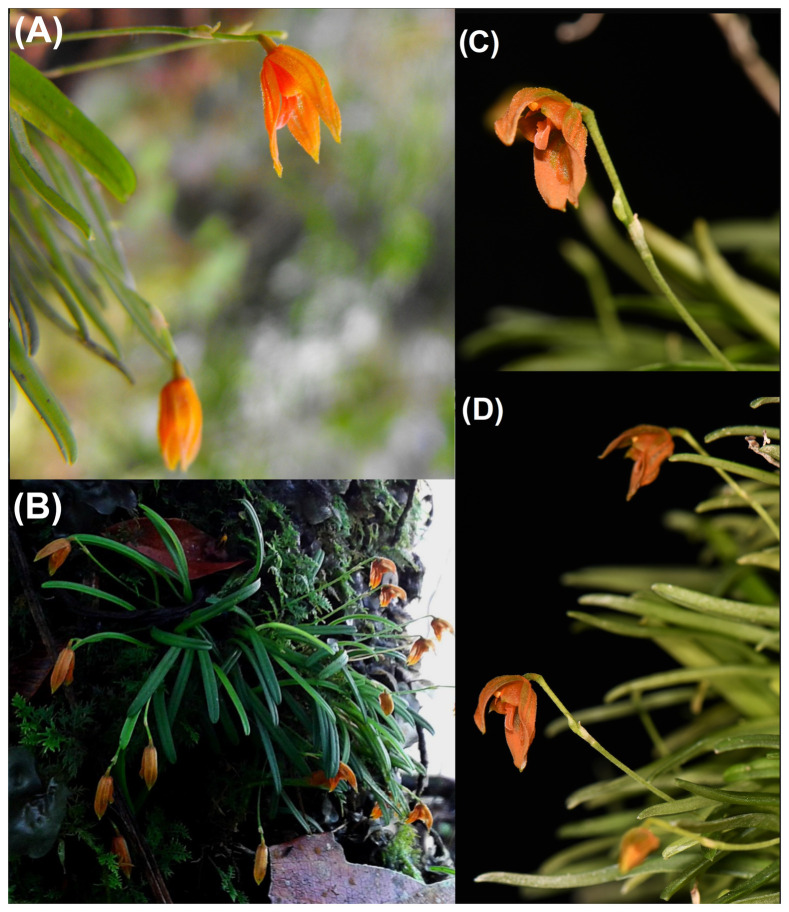
Photographs of *Specklinia vittariifolia*. (**A**) Specimen in its habitat, Tapachula, Chiapas. Photograph by Eduardo Martínez Ovando. (**B**) Specimen in its habitat, Montecristo de Guerrero, Chiapas. Photograph by Alexander Jiménez. (**C**,**D**) Specimen cultivated in Mexico City. Photographs by Ethian Licona, based on *O. Suárez 2372* (AMO).

**Figure 88:**
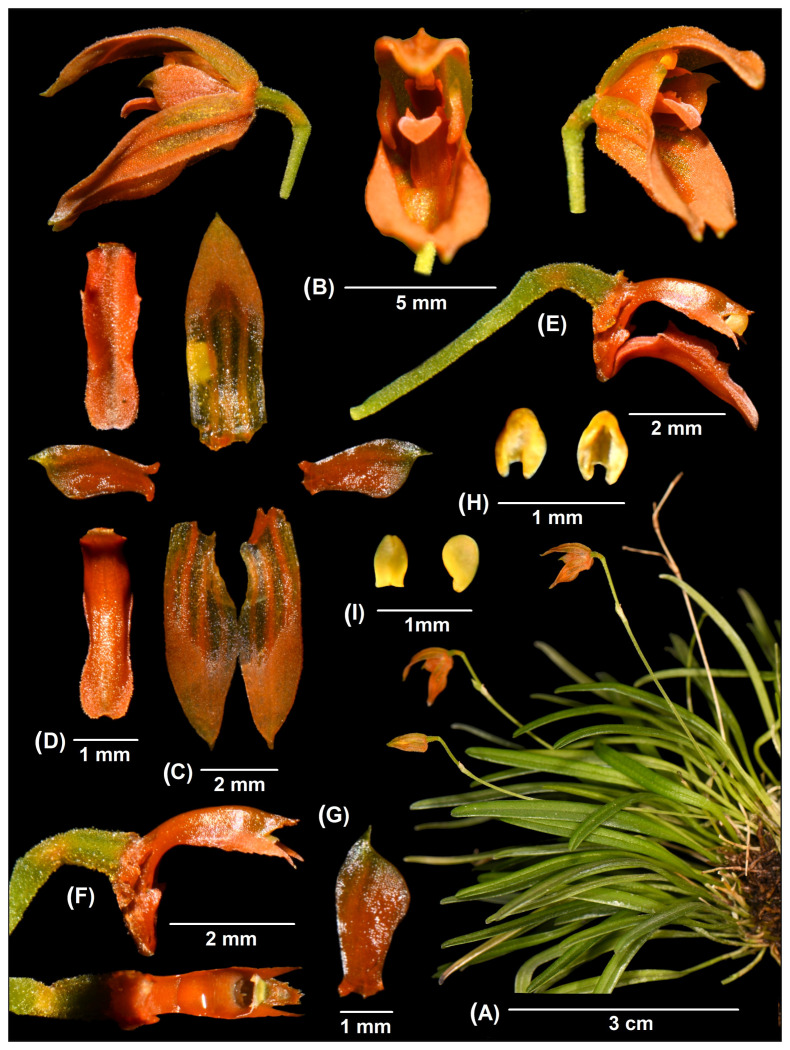
LCDP of *Specklinia vittariifolia*. (**A**) Habit. (**B**) Flower, ¾, frontal and lateral views. (**C**) Dissected flower. (**D**) Lip, dorsal and ventral views. (E) Column, lip and pedicelate ovary in lateral view. (**F**) Column, lateral and ventral views. (**G**) Petal. (**H**) Anther, dorsal and ventral views. (**I**) Pollinia, lateral and frontal views. Plate by Ethian Licona, based on *O. Suárez 2372* (AMO).

Herb cespitose, erect, up to 6.2 cm tall without the inflorescence. Roots 0.4–0.8 mm gross. Rhizome 0.5–1.5 mm long between adjacent stems. Stem 2.5–8.7 mm long, 0.3–0.7 mm gross, sheaths 1.2–5.0 mm long. Leaves 10.0–45.0 mm × 0.5–2.6 mm, acicular, obtuse to truncate, succulent, triquetrous, glabrous, the apical mucro 0.3–0.5 mm long, light green, gradually attenuated into a petiole 2.5–8.0 mm long, 0.5–1.0 mm gross. Inflorescence 1.7–7.0 cm long, congested, erect, glandulous; spathaceous bract 1.0–1.5 mm long; peduncle as long as the leaf, 1.5–4.3 cm long, 0.3–0.5 mm gross, with 2 bracts 1.5–2.1 mm long, membranaceous, glandulous, hyaline; rachis 0.2–2.8 cm long, with 1–7, distichous, successive flowers, 1–2 open at a time. Floral bracts 1.0–3.0 mm long, obliquely tubular-infundibuliform, obtuse, shortly apiculate, membranaceous, glandulous, hyaline. Flowers 4.8–9.0 mm × 1.9–3.0 mm; sepals, petals, lip and column bright orange, the sepals somewhat yellowish-translucent; anther yellow. Sepals concave, axially carinate, shortly apiculate, adaxially glabrous, abaxially glandulous, the margins cellular-glandulous; dorsal sepal 4.5–7.8 mm × 1.5–2.6 mm, oblong–lanceolate to oblong–ovate, obtuse, 3-veined, the apex slightly recurved; lateral sepals 4.8–9.0 mm × 2.0–4.0 mm, connate by ½ of its length into an oblong–lanceolate to oblong–oblanceolate, obtuse, deeply bifid synsepal, 6-veined, gibbose in the base. Petals 1.7–3.8 mm × 0.7–1.7 mm, obliquely obovate–spathulate, obtuse, apiculate, slightly falcate, 2-veined. Lip 2.5–4.5 mm × 0.7–1.5 mm, fleshy, shortly 3-lobulate, 3-veined, papillose–verrucose; lateral lobes erect, subpandurate-trapezoidal, obtuse, apiculate; mid lobe oblong–obovate, rounded, slightly emarginate, recurved, marginally erose–dentate, with a pair of verrucose submarginal calli, channeled between them. Column 2.6–3.2 mm long, 0.6–0.9 mm width; wings obliquely triangular to subulate, dentate to erose, 1.5 × 0.5 mm; clinandrium obtuse, dentate; foot 1.0–2.0 mm long, 0.5–0.7 mm width, obtuse, with a pair of obliquely conical, papillose calli at the base. Stigmatic cavity 0.4 mm × 0.5 mm, elliptic–oblong; rostellum laminar, oblong. Anther 0.5–0.6 mm long, 0.4–0.5 mm width, elliptic–ovoid. Pollinia 0.3–0.4 mm × 0.25–0.3 mm, obovoid. Ovary 0.5–2.0 mm long, 0.3–0.8 mm gross, obconical, arched, glandulous, green to greenish-orange; pedicel 1.5–8.0 mm long, 0.2–0.5 mm gross, glandulous. Capsule 5.0–8.0 mm long, 1.8–3.0 mm gross, elliptic–obovoid, glandulous, greenish-yellow to greenish-orange (Figure 87 and Figure 88).

Distribution: Mexico, Guatemala, El Salvador, Honduras, Nicaragua, Costa Rica and Panama. In Mexico, the species is distributed only in the state of Chiapas, within the Floristic Provinces of Costa Pacífica, Serranías Transístmicas and El Soconusco.

**Figure 89:**
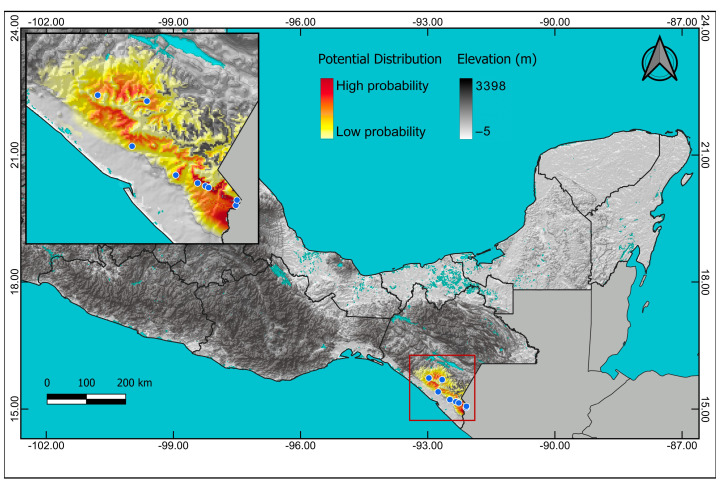
Potential distribution of *Specklinia vittariifolia* in Mexico and the location of currently known occurrence records (blue dots). Map by Ethian Licona.

The accepted potential distribution model for *S. vittariifolia* yields an AUC = 0.989, where the most influential environmental variables were precipitation of wettest quarter (55.0%), precipitation of warmest quarter (34.3%), and the Digital Elevation Model of Mexico (7.7%). The potential distribution area was estimated at 6725.44 km^2^, representing approximately 0.34% of Mexico’s total territory (Figure 89).

Habitat: Epiphyte on *Brosimum costaricanum*, *Liquidambar styraciflua*, *Quercus* sp. and *Terminalia oblonga*, also recorded on *Coffea arabica*. The species grows in the evergreen tropical forest, *Quercus* forest, tropical mountain cloud forest, riparian vegetation, secondary vegetation derived from these forest types and coffee plantations, at elevations between 620 and 1210 m.

Phenology: Flowering occurs from June to January. Mature fruits have been observed from October to March.

Conservation status: This species was previously classified in Mexico as Under Special Protection under the name *S. glandulosa* (Ames) Pridgeon & M.W.Chase, but this species is not distributed in the country. However, following a reassessment of its conservation status following the MER criteria and recognizing it as a different species [78], a score of 1.70 was obtained, indicating that this species should be considered as Threatened (A). In the under IUCN criterion B, it achieved an EOO of 2011.055 km^2^ and an AOO of 32.00 km^2^, suggesting that this species should be classified as Endangered (EN).

Additional commentaries: *Specklinia vittariifolia* can be recognized by its acicular, triquetrous leaves; erect, glandulous raceme; peduncle as long as the leaf, bearing up to seven distichous, congested, successive, glandulous, bright orange flowers with hyaline-yellow lines; synsepal oblong–lanceolate, apiculate, deeply bifid; petals obliquely obovate–spathulate, apiculate; lip shortly 3-lobulate, papillose–verrucose, bright orange; lateral lobes subpandurate-trapezoidal, erect and mid lobe oblong–obovate, rounded, slightly emarginate, erose–dentate.

Until recently, *S. vittariifolia* was treated as a synonym of *S. glandulosa*, the name under which the species was known in Mexico, and which was regarded as a morphologically variable taxon with a wide distribution from Mexico to Central America. However, Karremans et al. [65] demonstrated that this name represents a species complex comprising *Specklinia alajuelensis* Karremans & Pupulin, *S. glandulosa*, *S. pertenuis* (C.Schweinf.) Karremans & Gravend. and *S. vittariifolia*. Most members of this complex are distributed in southern Central America, where they may occur sympatrically, whereas *S. vittariifolia* is the only representative occurring in Mexico and northern Central America.

*Specklinia glandulosa* differs from *S. vittariifolia* by its smaller habit (plants up to 3.0 cm tall without the inflorescence vs. up to 6.2 cm tall); leaves linear–lanceolate, up to 2.1 cm long (vs. acicular, up to 4.5 cm long); inflorescence up to 4.0 cm long with a peduncle longer than the leaf (vs. up to 7.0 cm long with a peduncle as long as the leaf); bearing up to four dull orange flowers, up to 7.5 mm long (vs. up to seven bright orange flowers, up to 9.0 mm long); dorsal sepal narrowly lanceolate, up to 1.5 mm width (vs. oblong–lanceolate, up to 2.6 mm width); synsepal narrowly lanceolate, up to 2.5 mm width (vs. oblong–lanceolate, up to 4.0 mm width); and the lip up to 3.5 mm × 1.0 mm, with triangular lateral lobes and an oblong mid lobe (vs. up to 4.5 mm × 1.5 mm, with subpandurate-trapezoidal lateral lobes, and an obovate mid lobe).

In Mexico, this species has been confused with *S. lanceola*. However, it differs by its longer habit (up to 7.5 cm tall without the inflorescence vs. up to 6.2 cm tall); linear–lanceolate, flattened leaves, 26.0–51.0 mm × 2.0–7.1 mm (vs. acicular, triquetrous, 10.0–45.0 mm × 0.5–2.6 mm); inflorescence up to 8.2 cm long, lax, flexuous, glabrous (vs. up to 7.0 cm long, congested, erect, glandulous); glabrous, simultaneous flowers (vs. glandulous, successive flowers); petals oblong–obovate, truncate to widely obtuse, 1-veined (vs. obovate–spathulate, obtuse, 2-veined); lip entire, oblong–elliptic, truncate (vs. shortly 3-lobulate, slightly emarginate).

*Specklinia acicularis* is another morphologically similar species. However, it differs by its glabrous raceme (vs. glandulous); glabrous floral bracts (vs. glandulous); dark purple flowers, up to 9.5 mm long, adaxially spiculate, abaxially glabrous (vs. bright orange, up to 7.0 mm long, adaxially glabrous, abaxially glandulous); lateral sepals connate up to near their apices (vs. connate by ½ of its length); lip yellow with the apex magenta, with narrowly triangular lateral lobes (vs. bright orange, with subpandurate-trapezoidal lateral lobes); column with erose clinandrium (vs. dentate clinandrium).

Examined specimens: MEXICO. Chiapas: Acacoyagua, Monte Madre Vieja, 01.06.1938, *E. Matuda 2532* (AMES!, MEXU!, MICH!, SEL!). Huixtla, Finca Belén, undated, *A. Damon s/n* (ECO-TA-H!). La Concordia, Finca Custepec, por el camino NW hacía la finca, 12.07.1990, *R. Hampshire 1244 & A. Reyes-García* (MEXU!). Tapachula, Finca Irlanda, 01.01.1913, *C. Purpus 7711* (UC!); Santa Rosalía, 03.05.2008, *A. Damon s/n, R. Solano & L. Jiménez* (ECO-TA-H!). Unión Juárez, Volcán Tacaná, 01.11.2005, *O. Suárez 2372* (AMO!).

Additional records: MEXICO. Chiapas: Montecristo de Guerrero, en las cercanías de Montecristo 30.06.2014, *A. Jiménez s/n* (Photograph). Tapachula, Finca Guadalupe Zajú, 18.07.2012, *E. Martínez-Ovando s/n* (Photograph). Unión Juárez, Orquideario Santo Domingo, undated, *A. Damon s/n* (Photograph).

22. ***Specklinia wendtii*** E.Licona & Solano, *sp. nov.*

**Figure 90:**
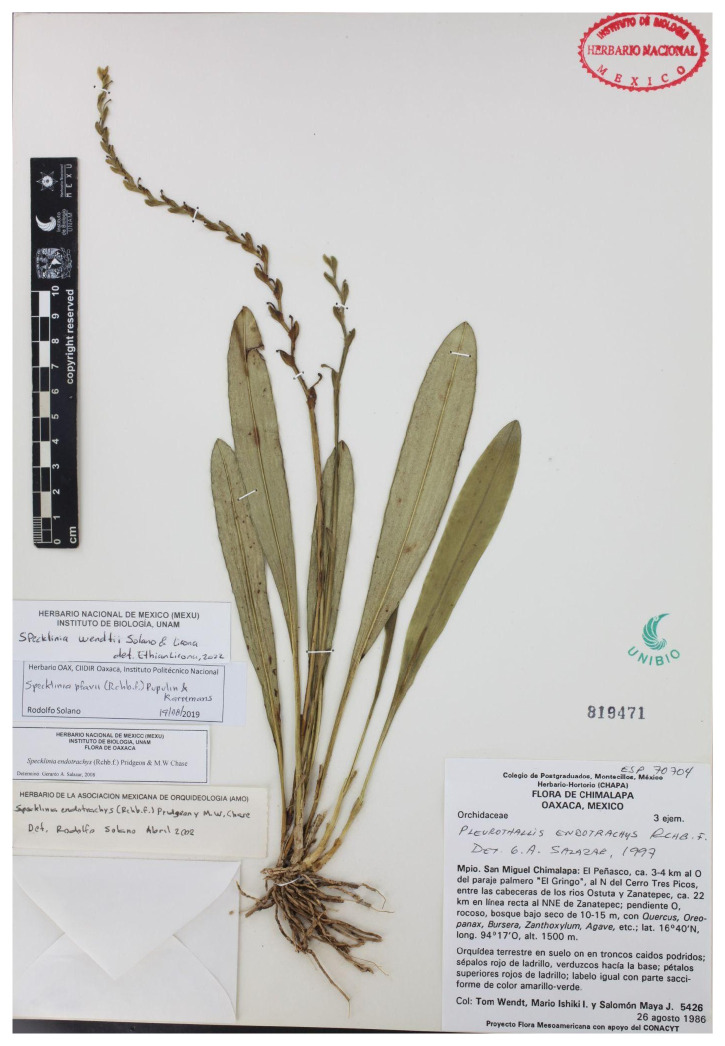
Holotype of *Specklinia wendtii, T. Wendt et al. 5426* (MEXU-819471), courtesy of the National Herbarium of the Universidad Nacional Autonoma de Mexico.

HOLOTYPE: MEXICO. Oaxaca: San Miguel Chimalapa, El Peñasco, 3–4 km al W del paraje palmero El Gringo, al N del cerro Tres Picos, entre las cabeceras de los ríos Ostuta y Zanatepec, ca. 22 km en línea recta al NNE de Zanatepec, ladera W, 1500 m, 26 August 1986, *T. Wendt 5426, M. Ishiki & S. Maya* (MEXU-819471! (Figure 90)).

Etymology: Named after Thomas Leighton Wendt, American pteridologist, collector of the type specimen and researcher of the Flora from Los Chimalapas-Uxpanapa, Mexico.

*Diagnosis: Similar to Specklinia remotiflora, from which it differs by its shorter habit, (up to 29.0 cm tall without the inflorescence vs. up to 43.0 cm tall), shorter rhizome (up to 15.0 mm long between adjacent stems vs. up to 25.0 mm long), rachis with internodes gradually shortening towards the apex (vs. internodes evenly spaced along the rachis); up to 35 flowers, 11.0–16.0 mm long (vs. up to 12 flowers, 15.0–20.0 mm long); petals geniculate at the apex (vs. concave at the apex); lip oblanceolate to obovate, acute (vs. elliptic–lanceolate, obtuse).*

**Figure 91:**
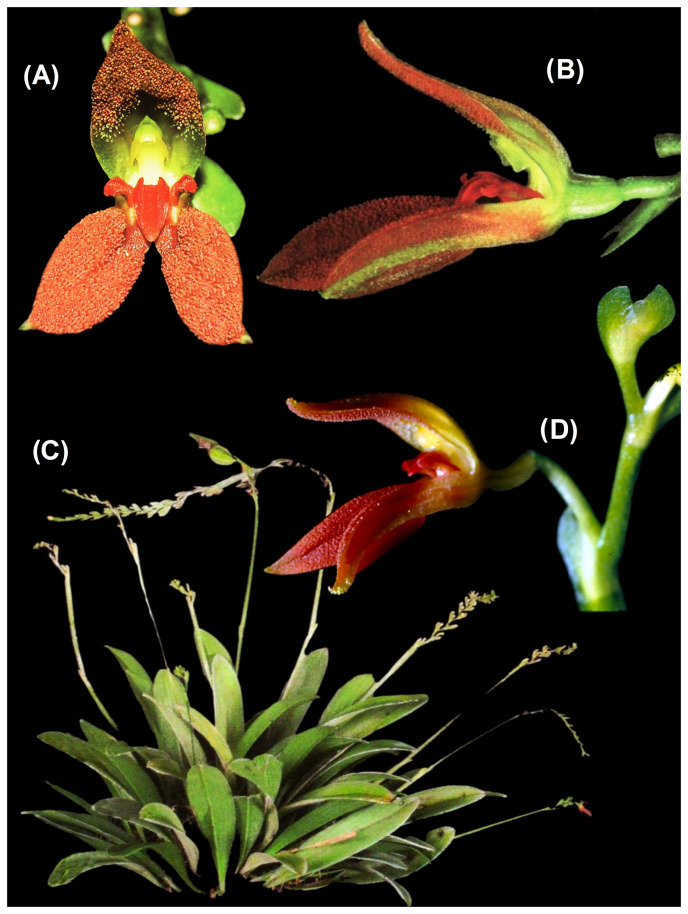
Photographs of *Specklinia wendtii*. (**A**–**C**) Specimen cultivated in San Andrés Huayapam, Oaxaca. Photographs by Octavio Suárez. (**D**) Specimen cultivated in Mexico City. Photograph by Eric Hágsater, based on *E. Hágsater 1261* (AMO).

**Figure 92:**
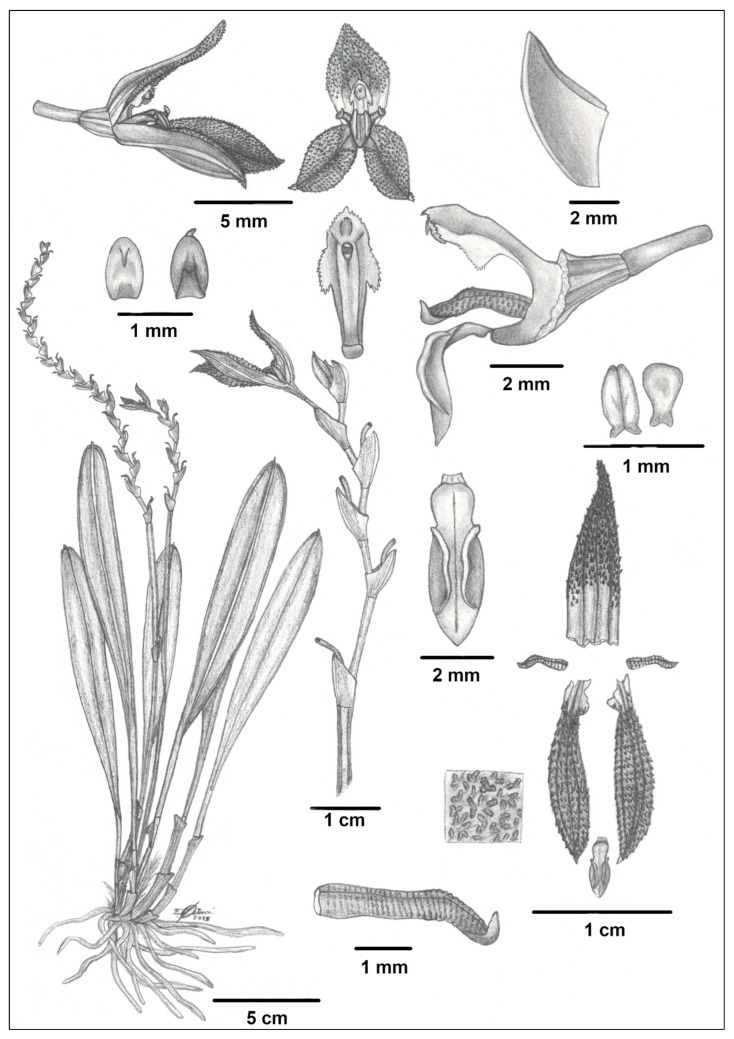
Illustration of *Specklinia wendtii*. Drawing by Ethian Licona based on *T. Wendt et al. 5426* (MEXU).

**Figure 93:**
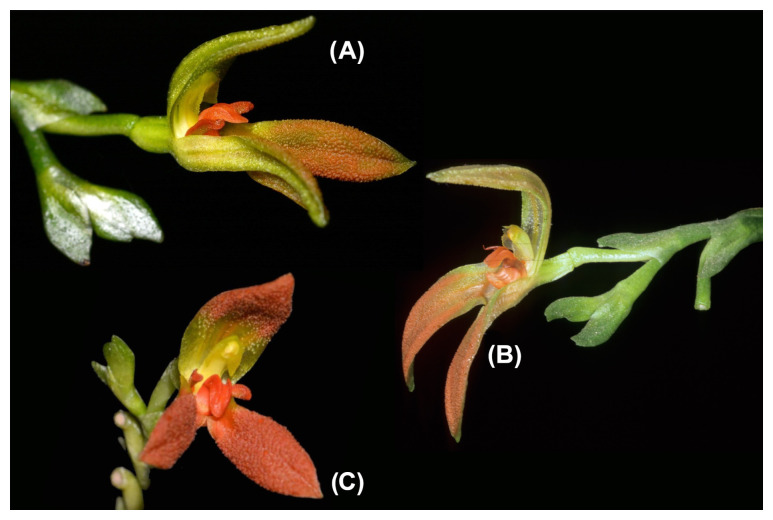
Comparison of flowers among the Mexican species of the *Specklinia endotrachys* complex. (**A**) *Specklinia juddii* from Alta Verapaz, Guatemala. Photograph by José Monzón & Edgar Mó. (**B**) *Specklinia spectabilis*. Photograph by Rodolfo Solano, based on *M.A. Soto-Arenas et al. 9484* (AMO). (**C**) *Specklinia wendtii*. Photograph and culture by Octavio Suárez, based on *O. Suárez s/n*.

Herb shortly rhizomatous, erect, up to 29.0 cm tall without the inflorescence. Roots 0.8–1.6 mm gross. Rhizome 2.5–15.0 mm long between adjacent stems. Stem 0.9–4.0 cm long, 1.5–2.3 mm gross, sheaths 0.7–3.1 cm long. Leaf 6.5–19.0 cm × 1.0–2.8 cm, membranous, narrowly obovate, obtuse, glabrous, the margins slightly recurved, the apical mucro 0.6–1.2 mm long, light green, gradually attenuated into a petiole 1.0–6.0 cm long, 1.4–2.2 mm gross. Inflorescence 11.4–43.5 cm long, ancipitous, congested, flexuous, glabrous; spathaceous bract 3.0–4.6 mm long; peduncle as long or slightly longer than the leaf, 7.3–21.0 cm long, 1.5–2.6 mm width, with 2–3 bracts 2.5–5.0 mm long, 2.7–3.0 mm width, obliquely infundibuliform, obtuse, ancipitous, fleshy, glabrous, light green; rachis 4.1–22.5 cm long, internodes spaced at the base, shortening towards the apex, distant by 4.0–10.0 mm long, the first internode longer, with 9–35, secund, successive flowers, one open at a time. Floral bracts 4.0–10.0 mm long, 3.5–6.5 mm width, obliquely infundibuliform, obtuse, ancipitous, fleshy, glabrous, non-overlapping, light green. Flowers 11.0–16.0 mm × 5.0–8.5 mm; sepals bright orange, basally yellow; petals and lip orange to reddish-orange; column and foot yellow; anther yellow. Sepals concave, axially keeled, adaxially verrucose, abaxially glabrous, the margins slightly recurved, 3-veined; dorsal sepal 11.0–15.5 mm × 3.0–6.0 mm, lanceolate, acute; lateral sepals 11.0–16.0 mm × 2.0–4.0 mm, connate near to the base, each one lanceolate to elliptic–lanceolate, acute, slightly falcate, sub-parallel, slightly reflex towards the apex. Petals 3.0–5.0 mm × 0.7–1.4 mm, linear–lanceolate, acute, concave, falcate, reflex, geniculate at the apex, adaxially striated–verrucose, abaxially glabrous, 2-veined. Lip 4.0–5.5 mm × 1.0–1.8 mm, fleshy, arched, oblanceolate, acute, slightly pandurate, 3-veined, minutely papillar-glandulous, the margins slightly recurved, with a pair of submarginal calli, channeled between them. Column 4.0–5.2 mm long, 1.3–2.5 mm width; wings widely triangular, oblique, obtuse, dentate, 1.6–2.2 mm × 0.8–1.0 mm; clinandrium obtuse, dentate; foot 2.0–3.0 mm long, 0.7–1.0 mm width, obtuse. Stigmatic cavity 0.4 mm × 0.6 mm, oblong; rostellum laminar, oblong. Anther 0.8–1.0 mm long, 0.4–0.7 mm width, elliptic–ovoid, apically apiculate. Pollinia 0.5–0.7 mm × 0.3–0.4 mm, obovoid. Ovary 3.0–6.0 mm long, 1.3–2.0 mm gross, obpyramidal, triquetrous, straight, glabrous, greenish-yellow; pedicel 3.0–12.0 mm long, 0.5–0.8 mm gross, glabrous. Capsule 1.3–1.8 cm long, 0.5–0.8 cm gross, elliptic–obovoid, glabrous, greenish-yellow (Figure 91, Figure 92 and Figure 93).

Distribution: Endemic. Up to now, it is only known from the states of Chiapas and Oaxaca, within the Floristic Provinces of Costa Pacífica, Serranías Transístmicas and El Soconusco.

**Figure 94:**
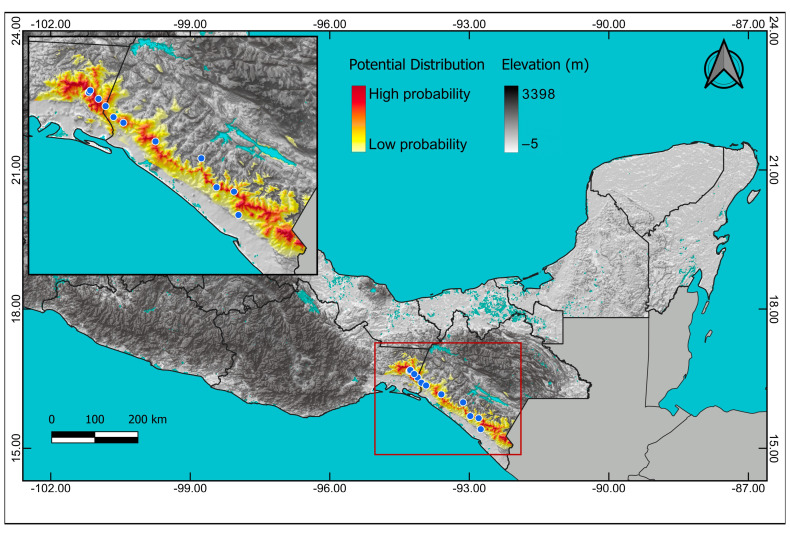
Potential distribution of *Specklinia wendtii* in Mexico and the location of currently known occurrence records (blue dots). Map by Ethian Licona.

The accepted potential distribution model for *S. wendtii* yields an AUC = 0.973, where the most influential environmental variables were temperature annual range (43.0%), the Digital Elevation Model of Mexico (21.6%) and isothermality (17.8%). The potential distribution area was estimated at 10,916.70 km^2^, representing approximately 0.56% of Mexico’s total territory (Figure 94).

Habitat: Epiphyte on *Quercus* sp., rarely terrestrial on humus and accumulated leaf litter. The species grows in the semi-deciduous tropical forest, *Pinus-Quercus* forest, *Quercus* forest and tropical mountain cloud forest at elevations between 840 and 2050 m.

Phenology: Flowering occurs from July to April. Mature fruits have been observed from October to July. The flowers emit a diurnal scent reminiscent of fermented fruit.

Conservation status: In the assessment of its conservation status following the MER criteria [78], a score of 1.74 was obtained, which suggests that this species should be considered as Threatened (A). In the evaluation under IUCN criterion B, it achieved an EOO of 2248.844 km^2^ and an AOO of 36.00 km^2^, suggesting that this species should be classified as Endangered (EN).

Additional commentaries: *Specklinia wendtii* can be recognized by its habit shortly rhizomatous; plants up to 30.0 cm tall; narrowly obovate, obtuse leaves; large, ancipitous inflorescence, up to 40.0 cm long, with a peduncle as long or slightly longer than the leaf, rachis flexuous, congested, with internodes shortening towards the apex; floral bracts ancipitous; bright orange flowers with yellow base; lateral sepals lanceolate, sub-parallel, slightly reflex towards the apex; petals linear–lanceolate, falcate, geniculate at the apex, adaxially striated–verrucose; lip oblanceolate, acute, arched.

Historically, this species and other morphologically similar taxa present in Mexico were considered as part of *S. endotrachys* [60] or *S. spectabilis* [91], both of which were regarded as the sole representative of this complex in the country.

This new species is a member of the *S. endotrachys* complex, which, with the inclusion of *S. wendtii*, now comprises seven taxa, three of which are distributed in Mexico and northern Central America. The morphological differences among these three Mesoamerican species are summarized in Table 6.

Examined specimens: MEXICO. Chiapas: Cintalapa, SE of Cerro Baúl, 16 km NW to Rizo de Oro, along the road to Colonia Figueroa, 21.04.1972, *D. Breedlove 24758* (MO!); Estación de Microondas La Mina, 13.11.1983, *D. Breedlove 60179 & F. Almeda* (CAS!). Jiquipilas, La Sepultura, Cerro La Palmita, 07.11.1994, *J. Castillo 404* (AMO!). La Concordia, camino a Cerro Venado, El Triunfo, 14.03.1998, *J. Castillo 1701 & F. Bolóm* (AMO!, CHIP!). Mapastepec, Reserva El Triunfo, 01.02.1990, *M. Heath 800 & A. Long* (CHIP!). Villa Corzo, Cerro La Angostura, ejido Nuevo Edén, 9 km al S de la colonia Plan de Ayala, 25.05.2002, *A. Farrera 2812* (HEM!); Cerro La Peña, al W del ejido Sierra Morena, 19.10.2002, *L. Alvarado 614 & A. Reyes* (MEXU!). Oaxaca: San Miguel Chimalapa, El Peñasco, 3–4 km al W del paraje palmero El Gringo, al N del cerro Tres Picos, 06.05.1986, *S. Maya 3300* (MEXU!); El Peñasco, 3–4 km al W del paraje palmero El Gringo, al N del cerro Tres Picos, sobre el arroyo del lado E, 13.09.1986, *S. Maya 3884* (CHAPA!, MEXU!); paraje palmero El Venado, cerca del parteaguas continental, en la región del Cerro El Retén, 28.07.1986, *S. Maya 3671* (CHAPA!); zona de Cerro Baúl, 01.03.1966, *R. Galley 1–377-52* (MEXU!).

Additional records: MEXICO. Oaxaca: San Andrés Huayapam, cultivated plant by Octavio Suárez, in Orchidarium La Encantada, undated, *O. Suárez s/n* (Photograph). San Miguel Chimalapa, Cerro Baúl, undated, *E. Hágsater 1261* (Photograph).

### 3.4. Excluded Species

*Specklinia endotrachys* (Rchb.f.) Pridgeon & M.W.Chase, Lindleyana, 16 (4): 257. 2001. TYPE: COSTA RICA. Alajuela: San Ramón, along the Barranca River. *A.R. Endrés 92* (lectotype (designated by Pupulin et al. [64]): W-21581!; isolectotypes: AMES-118500!, W-20331!, W-20150!).

Formerly, this taxon was considered as the sole species in the genus *Empusella*, with a distribution range from Mexico south to Venezuela. However, Pupulin et al. [64] demonstrated that it represents a species complex, and that *S. endotrachys* is endemic from Costa Rica. In Mexico, this complex is represented by three distinct but morphologically similar species: *S. juddii*, *S. spectabilis* and *S. wendtii*.

*Specklinia glandulosa* (Ames) Pridgeon & M.W.Chase, Lindleyana, 16 (4): 257. 2001. TYPE: PANAMA. Juan Grande range. Sea-level. September 1923. *C.W. Powell 306* (holotype: AMES-74295!; isotypes: AMES-74296!, MO-956087!).

Formerly regarded as a widely distributed and morphologically variable species, ranging from Mexico to Panama. *S. glandulosa* was shown by Karremans et al. [65] to be part of a species complex, with *S. glandulosa* restricted to Costa Rica and Panama. Mexican specimens previously identified under this name correspond to *S. vittariifolia*.

*Specklinia grobyi* (Bateman ex Lindl.) F.Barros, Hoehnea, 10: 110. 1983 [1984]. TYPE: GUYANA. Demerara. January 1834. *J. Bateman s/n* (holotype: (*sensu* Karremans) K-79839! [combined with *Specklinia grisebachiana*]; isotype: M-226535! (original watercolor realized by Lindley)).

Previously treated as a widely distributed and morphologically variable species, ranging from Mexico to southern Brazil and the Antilles. *Specklinia grobyi* has been demonstrated by several authors [2,26,87] to represent a species complex requiring further and deeper study. *Specklinia grobyi sensu stricto* is likely restricted to northwestern Amazonia and northern South America. Mexican specimens formerly assigned to this name correspond to five distinct but morphologically similar species: *S. choconiana*, *S. marginata*, *S. orbiculata*, *S. solanoi* and *S. xoconochcana*.

*Specklinia pfavii* (Rchb.f.) Pupulin & Karremans, Phytotaxa, 63: 8–10. 2012. TYPE: PANAMA. Chiriquí. *R. Pfau s/n* (Holotype: W-100935186).

Another representative of the *S. endotrachys* complex, this species was recognized as distinct despite its morphological similarity to related taxa, based on the study by Pupulin et al. [64]. *Specklinia pfavii* is restricted to Costa Rica and Panama. Mexican specimens previously identified under this name correspond to *S. juddii* and *S. wendtii*.

*Specklinia picta* (Lindl.) Pridgeon & M.W.Chase, Lindleyana, 16 (4): 259. 2001. TYPE: GUYANA. Demerara. 1834. *G. Loddiges s/n* (Holotype: K-79831! [includes a reproduction of the watercolor realized by Lindley]).

Formerly considered as a widely distributed and morphologically variable species, ranging from Mexico to southern Brazil. *Specklinia picta* has likewise been shown to represent a species complex [2,26,87], requiring further and deeper study. *Specklinia picta sensu stricto* is probably restricted to northwestern Amazonia and northern South America. Mexican specimens previously assigned to this name correspond to *S. choconiana* and *S. marginata*.

## 4. Discussion

After the designation of a lectotype for *Specklinia* by Garay & Sweet [40], the study of this group of miniature orchids entered a period of renewed activity. However, it was the work of Luer [39,41,43], that significantly accelerated the almost uninterrupted process of discovering new species and stimulated greater interest in understanding their interrelationships. This momentum culminated in the phylogenetic study by Pridgeon et al. [7], in which *Specklinia* was definitively re-established and segregated from *Pleurothallis*. Subsequently, the phylogenetic analysis conducted by Karremans et al. [18], corroborated the monophyly of *Specklinia* and clarified its relationships with other genera within the *Specklinia* alliance.

The study of Pridgeon et al. [7] also laid the foundation for further research on *Specklinia* in Mexico. After almost three centuries of botanical exploration in the country, several species had been recorded, and, with the additions made by Soto Arenas [84] and Soto Arenas & Solano [55,56,57,58,59,60,61], a total of 16 species were reported from Mexico at the beginning of the 21th century [62]. Following the generic delimitation proposed by Karremans et al. [18], 15 species of *Specklinia* were recognized [6], while four taxa previously assigned to this genus were transferred to *Muscarella*, (currently *Andreettaea* Luer) [18]: *M. marginata* (Rich.) Luer, *M. fimbriata* (Ames & C.Schweinf.) Luer, *M. samacensis* (Ames) Luer, and *M. segregatifolia* (Ames & C.Schweinf.) Karremans.

In the present study 22 species of *Specklinia* are recognized in Mexico, making the country the fifth worldwide in terms of species richness for the genus, surpassed only by Costa Rica, Panama, Colombia and Ecuador. Of these, five are described as new species, two represent new records for the country and four taxa previously treated as synonyms of morphologically similar species are here recognized as valid. Additionally, five species are endemic to Mexico: *S. digitale*, *S. hagsateri*, *S. microphylla*, *S. perez-garciae* and *S. wendtii*.

Through detailed examination of herbarium specimens, five species previously reported for Mexico were excluded from the national flora, as they are morphologically similar to Mexican taxa but do not actually occur in the country.

Two species deserve special attention due to their microendemic status and extremely restricted distribution. *Specklinia perez-garciae* is the most aberrant species in the genus, inhabiting a rocky outcrop within xerophytic scrub on the Pacific slope of the Isthmus of Tehuantepec, a habitat not shared by any other Mexican species of *Specklinia*. In contrast, *S. hagsateri* is restricted to the high mountains near Cerro Teotepec, the highest peak in the state of Guerrero, and is geographically isolated from all other species of the genus.

Of the 22 Mexican *Specklinia* species, only four were previously included in the Mexican list of at risk plant species [78]. Following reassessment of their conservation status using the MER criteria, seven additional species are proposed for inclusion as protected orchids in Mexico. Five under the category of Special Protection (*S. digitalis*, *S. echinata*, *S. juddii*, *S. microphylla* and *S. xoconochcana*); two as Threatened (*S. vittariifolia* and *S. wendtii*); and four as Endangered (*S. corniculata*, *S. hagsateri*, *S. perez-garciae* and *S. spectabilis*). In this context, the category ‘Under Special Protection’ in Mexico indicates that, although the species is not currently threatened, it is close to becoming so; therefore, it is legally protected against illegal trade and considered important for conservation

Meanwhile, for the IUCN Red List categories, the inclusion of 14 Mexican species is proposed: two as Near Threatened (*S. lanceola* and *S. pisinna*), four as Vulnerable (*S. echinata*, *S. juddi*, *S. microphylla*, and *S. orbiculata*), six as Endangered (*S. corniculata*, *S. digitale*, *S. spectabilis*, *S. vittariifolia*, *S. wendtii*, and *S. xoconochcana*), and two as Critically Endangered (*S. hagsateri* and *S. perez-garciae*).

Fortunately, these species are not currently subject to significant collection pressure for illegal trade. Nevertheless, interest in miniature orchids has increased in recent years in Mexico, a trend that could carry future risks. At present, the main threats faced by these species include illegal logging, land-use change, increasingly frequent forest fires and the impacts of urbanization and climate change. Due to their small size, these orchids are highly dependent on stable microenvironmental conditions, the presence of suitable phorophyte species (many of which are timber trees) and relatively undisturbed habitats, as only a few species are capable of persisting in secondary vegetation.

At present, almost no conservation efforts are being undertaken to protect these threatened species, and no governmental programs exist to ensure their conservation. Only one institutional initiative focuses on the reproduction of an endemic species, *S. digitale* [95]. Some species are cultivated and propagated vegetatively for conservation purposes at several institutions: the Orquideario Miguel Ángel Soto (*S. perez-garciae*) and the Eric Hágsater AMO Herbarium (*S. lanceola*, *S. perez-garciae*, *S. solanoi*, *S. spectabilis*, *S. wendtii*, and *S. xoconochcana*), both in Mexico City; the Jardín Botánico Clavijero in Xalapa, Veracruz (*S. digitale*, *S. lanceola*, and *S. microphylla*); the Jardín Etnobiológico de las Selvas del Soconusco (*S. juddii* and *S. xoconochcana*); and the Orquideario Santo Domingo in the Tacaná region, both in Chiapas (*S. juddii*, *S. lanceola*, and *S. vittariifolia*).

## 5. Conclusions

This monographic revision provides an updated taxonomic treatment of *Specklinia* in Mexico and significantly expands the documented diversity of the genus in the country. Based on the examination of herbarium material, cultivated specimens, the literature, and online databases, 22 species of *Specklinia* are recognized for Mexico, including five new species, two new country records, two new combinations and four taxa reinstated as valid. Five species are endemic to Mexico. For each species, morphological descriptions, photographs, illustrations, synonymy, potential distribution models and data on distribution, habitat, phenology, conservation status and taxonomic affinities are provided. This study establishes a robust taxonomic framework for *Specklinia* in Mexico and highlights the need for continued systematic and conservation research for this charismatic and fascinating miniature orchid genus, particularly in light of the proposed inclusion of 11 species under NOM-059-SEMARNAT-2010, and 14 species under the IUCN Red List of endangered species. It is necessary to consider integrative strategies to prevent the loss of these marvelous orchids in Mexico.

## Figures and Tables

**Table 1:** Conservation status for Mexican species of *Specklinia* according to the Extinction Risk Assessment Method [78] and according to the IUCN Criterion B. Pr: Under Special Protection. A: Threatened. P: Endangered. EOO: Extent of occurrence. AOO: Area of occupancy. LC: Least Concern. NT: Near Threatened. VU: Vulnerable. EN: Endangered. CR: Critically Endangered. The status for *S. acicularis* could not be assessed due to insufficient information for Mexican populations. The numbers of the MER assessment represent the average value obtained for each criterion.

	MER						IUCN		
Species	Criterion A: Geographic Distribution	Criterion B: Habitat Status	Criterion C: Biological Vulnerability	Criterion D: Anthropogenic Impact	Total	Status	EEO	AOO	Status
*S. blancoi*	0.36	0.11	0.17	0.0	0.64	Without risk	53,657.492 km^2^	68.000 km^2^	LC
*S. brighamii*	0.36	0.44	0.17	0.0	0.97	Without risk	123,943.998 km^2^	184.000 km^2^	LC
*S. choconiana*	0.18	0.22	0.17	0.0	0.57	Without risk	181,895.063 km^2^	224.000 km^2^	LC
*S. corniculata*	0.91	0.89	0.39	0.2	2.39	P	3590.523 km^2^	16.000 km^2^	EN
*S. digitale*	0.72	0.33	0.17	0.3	1.52	Pr	1141.913 km^2^	96.000 km^2^	EN
*S. echinata*	0.63	0.56	0.22	0.2	1.61	Pr	6044.997 km^2^	32.000 km^2^	VU
*S. fuegi*	0.36	0.33	0.17	0.0	0.86	Without risk	46,334.072 km^2^	80.000 km^2^	LC
*S. hagsateri*	0.91	0.67	0.3	0.2	2.08	P	21.865 km^2^	12.000 km^2^	CR
*S. juddii*	0.72	0.33	0.17	0.3	1.52	Pr	2517.395 km^2^	52.000 km^2^	VU
*S. lanceola*	0.54	0.33	0.26	0.1	1.23	Without risk	64,895.562 km^2^	48.000 km^2^	NT
*S. marginata*	0.18	0.11	0.17	0.0	0.46	Without risk	285,290.042 km^2^	392.000 km^2^	LC
*S. microphylla*	0.72	0.44	0.17	0.2	1.53	Pr	19,015.418 km^2^	60.000 km^2^	VU
*S. orbiculata*	0.54	0.56	0.17	0.2	1.47	Without risk	47,955.140 km^2^	40.000 km^2^	VU
*S. perez-garciae*	1.0	0.89	0.39	0.2	2.48	P	—	2.000 km^2^	CR
*S. pisinna*	0.54	0.44	0.17	0.1	1.25	Without risk	23,634.983 km^2^	60.000 km^2^	NT
*S. solanoi*	0.36	0.44	0.17	0.2	1.17	Without risk	113,342.097 km^2^	124.000 km^2^	LC
*S. spectabilis*	0.91	0.78	0.35	0.1	2.14	P	223.842 km^2^	24.000 km^2^	EN
*S. tribuloides*	0.09	0.0	0.17	−0.1	0.16	Without risk	290,034.292 km^2^	468.000 km^2^	LC
*S. vittariifolia*	0.72	0.56	0.22	0.2	1.7	A	2011.055 km^2^	32.000 km^2^	EN
*S. wendtii*	0.81	0.56	0.17	0.2	1.74	A	2248.844 km^2^	36.000 km^2^	EN
*S. xoconochcana*	0.72	0.56	0.17	0.2	1.65	Pr	3783.727 km^2^	44.000 km^2^	EN

**Table 2:** Morphological comparison among *Specklinia grobyi* and related Mexican species.

	*S. choconiana*	*S. grobyi*	*S. marginata*	*S. orbiculata*	*S. solanoi*	*S. xoconochcana*
Height	Up to 3.0 cm without the inflorescence	Up to 4.0 cm without the inflorescence	Up to 3.0 cm without the inflorescence	Up to 1.8 cm without the inflorescence	Up to 4.0 cm without the inflorescence	Up to 4.6 cm without the inflorescence
Habit	Cespitose, ascending to prostrate	Cespitose, ascending to prostrate	Shortly rhizomatous, prostrate	Shortly rhizomatous, prostrate	Cespitose, ascending to prostrate	Shortly rhizomatous, erect
Leaves	Obovate to oblanceolate, obtuse, 5.0–20.0 × 3.0–7.6 mm	Obovate to spathulate, rounded, 7.4–22.0 × 3.6–8.4 mm	Elliptic to elliptic–oblanceolate, acute, 5.0–18.0 × 3.0–7.6 mm	Orbicular–elliptic to elliptic–obovate, rounded, 5.0–9.0 × 3.0–6.5 mm	Obovate to elliptic–oblanceolate, obtuse, 6.0–25.0 × 3.4–9.2 mm	Oblanceolate to elliptic– oblanceolate, obtuse, 8.2–30.0 × 4.0–9.0 mm
Raceme	Up to 11.8 cm long, peduncle longer than the leaf	Up to 9.6 cm long, peduncle longer than the leaf	Up to 12.8 cm long, peduncle longer than the leaf	Up to 8.2 cm long, peduncle longer than the leaf	Up to 17.6 cm long, peduncle longer than the leaf	Up to 9.9 cm long, peduncle as long as the leaf
Flower	Yellow with red lines, distichous, sub-parallel to the rachis	Yellow with red lines, distichous, perpendicular to the rachis	White to yellowish with pink lines, secund, perpendicular to the rachis	Yellow with red lines, distichous, sub-parallel to the rachis	Yellowish, concolorous, distichous, perpendicular to the rachis	White to yellowish, concolorous, secund, perpendicular to the rachis
Dorsal sepal	Lanceolate to narrowly ovate, acuminate, 3.0–7.0 × 1.0–2.9 mm	Ovate, acute, 3.0–5.4 × 1.2–2.0 mm	Lanceolate to narrowly ovate, acute, 3.0–4.8 × 1.0–2.0 mm	Elliptic–lanceolate to elliptic–ovate, acute, 2.8–4.7 × 1.0–1.8 mm	Oblong–ovate to oblong–lanceolate, acuminate, 2.9–5.4 × 1.2–2.2 mm	Lanceolate to ovate, acuminate, 3.0–5.8 × 1.2–2.5 mm
Synsepal	Lanceolate to oblong–lanceolate, acuminate, 3.4–7.9 × 1.5–3.4 mm	Oblong–lanceolate to ovate, obtuse, 3.2–5.8 × 1.4–2.8 mm	Oblong–elliptic to oblong– lanceolate, acute, 3.2–5.5 × 1.6–2.7 mm	Elliptic–lanceolate to oblong–lanceolate, acute, 3.5–5.0 × 1.6–2.5 mm	Oblong–lanceolate, acuminate, 3.3–6.0 × 1.5–2.6 mm	Oblong–oblanceolate to elliptic–oblanceolate, obtuse, 3.0–6.4 × 1.3–2.9 mm
Petals	Oblong–lanceolate, acuminate, 1.6–2.6 × 0.4–1.0 mm	Oblong–lanceolate, acuminate, 1.4–2.0 × 0.6–0.8 mm	Obliquely obovate, acuminate, 1.6–2.2 × 0.5–0.7 mm	Obliquely oblong–obovate, obtuse, 1.4–2.1 × 0.4–0.6 mm	Obliquely oblong–obovate, obtuse, 1.4–2.2 × 0.4–0.8 mm	Obliquely lanceolate, acute, 1.8–2.9 × 0.5–0.7 mm
Lip	Oblong–ligulate, 1.8–3.0 × 0.6–1.3 mm	Oblong–ligulate, 1.6–2.5 × 0.5–1.2 mm	Oblong–ligulate, 1.7–2.1 × 0.5–0.9 mm	Oblong–ligulate, 1.6–2.4 × 0.5–0.8 mm	Oblong–obovate, 1.5–2.4 × 0.6–1.1 mm	Oblong–obovate, 1.8–2.9 × 0.5–1.2 mm

**Table 3:** Morphological comparison among species of the *Specklinia digitale* complex.

	*S. digitale*	*S. lugduno-batavae*	*S. microphylla*	*S. perez-garciae*	*S. pisinna*
Height	Up to 2.8 cm without the inflorescence	Up to 1.0 cm without the inflorescence	Up to 1.9 cm without the inflorescence	Up to 2.5 cm without the inflorescence	Up to 2.3 cm without the inflorescence
Leaves	Obovate to elliptic–obovate, 5.0–19.0 × 3.4–7.0 mm	Elliptic–orbicular, 4.0–8.0 × 3.0–6.0 mm	Elliptic–orbicular to oblong–elliptic, 4.0–10.8 × 2.5–5.4 mm	Elliptic–oblanceolate, 6.7–20.0 × 3.0–10.0 mm	Elliptic–obovate to oblong–obovate, 4.0–14.0 × 3.0–6.5 mm
Raceme	Up to 10.8 cm long, peduncle longer than the leaf	Up to 2.0 cm long, peduncle as long as the leaf	Up to 2.7 cm long, peduncle as long as the leaf	Up to 4.5 cm long, peduncle shorter than the leaf	Up to 3.6 cm long, peduncle longer than the leaf
Flowers	Cream, up to 10 flowers	White, up to 6 flowers	White, up to 6 flowers	Yellowish, up to 10 flowers	White with magenta lines, up to 6 flowers
Dorsal sepal	Oblong–elliptic to oblong–ovate, 4.0–5.5 × 1.9–2.5 mm, calyptra 0.5–0.7 × 0.5–0.8 mm	Elliptic–lanceolate, 4.0–4.5 × 2.1–2.3 mm, without a conspicuous calyptra	Lanceolate to oblong–ovate, 2.5–4.0 × 1.3–1.6 mm, calyptra 0.4–0.5 × 0.3–0.4 mm	Oblong–ovate to elliptic, 3.3–5.1 × 1.4–2.2 mm, calyptra 1.0–1.4 × 0.6–0.8 mm	Oblong–elliptic to oblong–ovate, 2.8–4.5 × 1.4–2.2 mm, calyptra 0.5–0.7 × 0.5–0.8 mm
Synsepal	Ovate to oblong–elliptic, 4.1–5.9 × 2.0–3.3 mm	Elliptic to elliptic–ovate, 4.0–4.5 × 2.8–3.0 mm	Ovate to elliptic, 2.7–4.3 × 1.4–2.5 mm	Elliptic–oblong, 3.6–5.5 × 2.3–3.2 mm	Elliptic to ovate, 3.2–4.7 × 1.9–3.0 mm
Petals	Obliquely obovate, obtuse, 2.0–2.4 × 0.8–1.1 mm	Elliptic–oblong, obtuse, 2.1–2.2 × 0.9–1.0 mm	Obliquely oblong–obovate, obtuse, 1.4–2.3 × 0.4–0.6 mm	Obliquely oblanceolate, obtuse, 2.1–2.5 × 0.6–1.0 mm	Lanceolate, acute, 1.5–2.8 × 0.5–0.9 mm
Lip	Oblong–ligulate, obtuse, yellowish, 1.7–2.2 × 0.6–1.1 mm	Oblong–ligulate, obtuse, yellowish, 1.5–1.8 × 0.7–0.8 mm	Oblong–obovate, rounded, yellowish, 1.5–1.8 × 0.5–0.6 mm	Oblong–ligulate, obtuse, yellowish, 1.8–2.4 × 0.8–1.3 mm	Elliptic–oblong, rounded, magenta, 1.8–2.5 × 0.7–1.1 mm

**Table 4:** Morphological comparison among Mexican species of *Specklinia* subg. *Sylphia*.

	*S. echinata*	*S. fuegi*	*S. hagsateri*
Heigh	Up to 3.0 cm without the inflorescence	Up to 2.8 cm without the inflorescence	Up to 2.6 cm without the inflorescence
Leaves	Elliptic–obovate to elliptic–lanceolate, obtuse, 5.0–19.0 × 3.4–7.0 mm	Lanceolate to elliptic–lanceolate, acute, 5.0–12.0 × 2.0–5.2 mm	Obovate to elliptic–obovate, obtuse, 5.0–12.5 × 3.0–5.0 mm
Raceme	Up to 6.7 cm long, peduncle longer than the leaf, flowers spaced each other by 3.0–11.5 mm	Up to 7.6 cm long, peduncle as long as the leaf, flowers spaced each other by 1.4–7.0 mm	Up to 6.2 cm long, peduncle longer than the leaf, flowers spaced each other by 2.0–8.4 mm
Flowers	Yellowish-white suffused with purple, up to 10 flowers, 5.0–7.8 × 2.6–4.4 mm	Yellowish-white, concolorous, up to 18 flowers, 3.3–6.5 × 1.4–3.0 mm	Yellowish-white, concolorous, up to 8 flowers, 3.4–5.0 × 1.6–2.8 mm
Dorsal sepal	Oblong–ovate to oblong–lanceolate, 5.2–7.8 × 1.2–2.9 mm, cauda 2.0–2.5 × 0.2–0.5 mm	Oblanceolate to oblong–obovate, 4.0–6.5 × 1.0–2.3 mm, cauda 1.3–1.8 × 0.2–0.4 mm	Oblanceolate to oblong–obovate, 4.5–5.6 × 1.4–1.9 mm, cauda 1.0–1.5 × 0.2–0.4 mm
Lateral sepals	Oblong–lanceolate, 5.0–7.3 × 1.4–2.4 mm, caudae 1.5–2.2 × 0.2–0.4 mm	Lanceolate, 3.5–5.9 × 0.8–1.4 mm, caudae 1.0–2.2 × 0.2–0.4 mm	Oblong–lanceolate, 3.5–5.0 × 0.9–1.5 mm, caudae 1.1–1.6 × 0.2–0.4 mm
Petals	Oblong–obovate, 1.5–2.7 × 0.6–1.3 mm	Obovate, 1.5–2.1 × 0.5–1.2 mm	Obovate, 1.5–1.9 × 0.8–1.0 mm
Lip	Purple with the apex yellow, lateral lobes elliptic–obovate, mid lobe obovate, 2.2–3.0 × 1.5–2.2 mm	Greenish-yellow with purple, lateral lobes elliptic, mid lobe oblong–obovate, 1.7–2.5 × 0.8–1.8 mm	Greenish-yellow, lateral lobes elliptic, mid lobe oblong, 2.0–2.7 × 1.0–1.3 mm

**Table 5:** Morphological comparison among species of the *Specklinia corniculata* complex.

	*S. barboselloides*	*S. corniculate*	*S. displosa*	*S. striata*
Heigh	Up to 8.0 cm without the inflorescence	Up to 4.5 cm without the inflorescence	Up to 8.0 cm without the inflorescence	Up to 7.0 cm without the inflorescence
Leaves	Oblong–oblanceolate, obtuse, 10.0–46.0 × 4.0–12.0 mm	Elliptic–lanceolate, obtuse, 10.0–25.0 × 3.0–9.0 mm	Narrowly oblanceolate, acute, 40.0–60.0 × 6.0–12.0 mm	Lanceolate, acute, 10.0–45.0 × 3.0–9.0 mm
Raceme	Up to 8.0 cm long, peduncle longer than the leaf, up to 3 flowers	Up to 5.3 cm long, peduncle as long as the leaf, with one functional flower	Up to 12.0 cm long, peduncle longer than the leaf, up to 25 flowers	Up to 7.2 cm long, peduncle as long as the leaf, up to 8 flowers
Flowers	Yellowish-orange, 5.0–10.0 × 2.0–3.7 mm, pedicel 8.0–15.0 mm	Bright yellow to greenish-yellow, 4.5–6.0 × 1.8–2.8 mm, pedicel 7.0–13.0 mm	Orange, 4.0–8.0 × 1.6–3.0 mm, pedicel 3.0–5.0 mm	Orange, 3.0–6.0 × 1.4–2.2 mm, pedicel 3.0–8.0 mm
Dorsal sepal	Oblong–lanceolate 5.0–10.0 × 1.5–3.0 mm	Oblong–lanceolate, 4.5–6.0 × 1.5–2.3 mm	Ovate, 3.8–7.0 × 1.4–2.9 mm	Ovate, 3.4–5.5 × 1.0–2.0 mm
Synsepal	Elliptic–lanceolate, obtuse, 5.0–10.0 × 2.0–4.0 mm	Elliptic–lanceolate, obtuse, 4.5–6.0 × 2.5–3.5 mm	Oblong–ovate, acute, 4.0–8.0 × 1.9–3.5 mm	Oblong–ovate, acute, 3.8–6.0 × 1.6–2.5 mm
Petals	Oblong–subrhombic, acuminate, 2.0–3.0 × 0.8–1.8 mm	Oblong–subrhombic, acute and distally acuminate, 1.6–2.3 × 0.8–1.2 mm	Obliquely obovate, acute, 1.6–2.5 × 0.8–1.5 mm	Oblong–subrhombic, acute, 1.2–1.8 × 0.6–1.0 mm
Lip	Yellowish-orange, lateral lobes minutely elliptic, mid lobe narrowly lanceolate, 2.5–3.0 × 0.6–1.5 mm	Yellow, lateral lobes widely triangular, mid lobe obovate, 2.2–2.5 × 0.7–1.2 mm	Orange, lateral lobes minutely triangular, mid lobe oblong–lanceolate, 2.0–2.7 × 0.5–1.2 mm	Orange, lateral lobes minutely triangular, mid lobe oblong, 1.8–2.2 × 0.8–1.5 mm
Distribution	Pacific slope of Costa Rica and Panama	Caribbean basin of Central America and Greater Antilles	Endemic of Panama	Panama, Trinidad and northern South America

**Table 6:** Morphological comparison among Mexican species of the *Specklinia endotrachys* complex.

	*S. juddii*	*S. spectabilis*	*S. wendtii*
Height	Up to 30.0 cm without the inflorescence	Up to 25.0 cm without the inflorescence	Up 30.0 cm without the inflorescence
Habit	Cespitose	Cespitose	Shortly rhizomatous
Leaves	Narrowly oblanceolate, obtuse, 4.5–17.7 × 0.7–2.1 cm	Narrowly oblanceolate, acute, 4.1–15.7 × 0.7–1.5 cm	Narrowly obovate, obtuse, 6.5–19.0 × 1.0–2.8 mm
Raceme	Up to 41.0 cm long, peduncle as long as the leaf, floral internodes 3.0–8.0 mm long, flowers evenly spaced, floral bracts slightly imbricate	Up to 64.0 cm long, peduncle longer than the leaf, floral internodes 5.0–15.0 mm long, flowers evenly spaced, floral bracts imbricate	Up to 44.0 cm long, peduncle as long as the leaf, floral internodes 4.0–10.0 mm long, flowers gradually becoming congested towards the apex, floral bracts no imbricate
Flowers	11.5–21.3 × 10.0–12.5 mm	17.0–24.0 × 6.0–10.5 mm	11.0–16.0 × 5.0–8.5 mm
Sepals	Adaxially yellowish-orange, abaxially greenish-yellow	Bight orange with yellow margins	Bright orange with yellow base
Dorsal sepal	Elliptic–lanceolate, acute, 10.0–20.0 × 3.6–6.0 mm	Narrowly lanceolate, acuminate, 18.0–24.0 × 4.0–6.0 mm	Narrowly lanceolate, acute, 11.0–15.5 × 3.0–6.0 mm
Lateral sepals	Narrowly lanceolate, acute, 11.5–21.3 × 2.0–4.0 mm, sub-parallel, vertically oriented with their adaxial surface facing each other	Narrowly lanceolate, acuminate, 16.0–22.0 × 3.5–4.5 mm, divergent, horizontally oriented, not facing each other	Narrowly lanceolate, acute, 11.0–16.0 × 2.0–4.0 mm, sub-parallel, horizontally oriented, not facing each other
Petals	Linear–lanceolate, acute, apex cucullate, 3.7–6.0 × 0.8–1.5 mm	Linear–lanceolade, acute, apex with a filamentous appendage, 4.0–5.1 × 0.7–1.0 mm	Linear–lanceolate, sub-rounded, apex geniculate, 3.0–5.0 × 0.7–1.4 mm
Lip	Oblong–lanceolate, acute, 4.5–5.5 × 1.5–2.0 mm	Oblong–subpandurate, truncate, 5.5–7.5 × 2.2–2.8 mm	Oblanceolate, acute, 4.0–5.5 × 1.0–1.8 mm

## Data Availability

The authors assure that, by mutual agreement, we have shared data and information included in this study for its potential publication.
